# Transcriptome analysis of a nematode resistant and susceptible upland cotton line at two critical stages of *Meloidogyne incognita* infection and development

**DOI:** 10.1371/journal.pone.0221328

**Published:** 2019-09-10

**Authors:** Pawan Kumar, Sameer Khanal, Mychele Da Silva, Rippy Singh, Richard F. Davis, Robert L. Nichols, Peng W. Chee

**Affiliations:** 1 Dept. of Crop and Soil Sciences and Institute of Plant Breeding, Genetics, and Genomics, University of Georgia, Tifton, GA, United States of America; 2 Department of Plant Pathology, University of Georgia, Tifton, GA, United States of America; 3 USDA-ARS, Crop Protection and Management Research Unit, Tifton, GA, United States of America; 4 Cotton Incorporated, Cary, NC, United States of America; CSIRO, AUSTRALIA

## Abstract

Host plant resistance is the most practical approach to control the Southern root-knot nematode (*Meloidogyne incognita*; RKN), which has emerged as one of the most serious economic pests of Upland cotton (*Gossypium hirsutum* L.). Previous QTL analyses have identified a resistance locus on chromosome 11 (*qMi-C11*) affecting galling and another locus on chromosome-14 (*qMi-C14*) affecting egg production. Although these two QTL regions were fine mapped and candidate genes identified, expression profiling of genes would assist in further narrowing the list of candidate genes in the QTL regions. We applied the comparative transcriptomic approach to compare expression profiles of genes between RKN susceptible and resistance genotypes at an early stage of RKN development that coincides with the establishment of a feeding site and at the late stage of RKN development that coincides with RKN egg production. Sequencing of cDNA libraries produced over 315 million reads of which 240 million reads (76%) were mapped on to the *Gossypium hirsutum* genome. A total of 3,789 differentially expressed genes (DEGs) were identified which were further grouped into four clusters based on their expression profiles. A large number of DEGs were found to be down regulated in the susceptible genotype at the late stage of RKN development whereas several genes were up regulated in the resistant genotype. Key enriched categories included transcription factor activity, defense response, response to phyto-hormones, cell wall organization, and protein serine/threonine kinase activity. Our results also show that the DEGs in the resistant genotype at *qMi-C11* and *qMi-C14* loci displayed higher expression of defense response, detoxification and callose deposition genes, than the DEGs in the susceptible genotype.

## Introduction

The genus *Meloidogyne* includes nearly 100 species, but four species–*M*. *incognita*, *M*. *arenaria*, *M*. *javanica* and *M*. *hapla*–account for approximately 95% of the total crop area infested by this genus. The Southern root-knot nematode (*Meloidogyne incognita*, RKN) is a sedentary endoparasitic nematode that penetrates plant roots as second-stage juveniles (J2s) and transforms multiple host cells into highly metabolically active ‘giant cells’ on which they feed to complete their life cycle. Formation of giant cells are initiated by the delivery of RKN ‘effectors’ into the host cells where they modulate plant signal transduction pathways resulting in multiple cycles of DNA replication followed by nuclear division without cytokinesis. Root tissues surrounding these giant cells undergo swelling resulting in gall formation, which can severely obstruct water and nutrient uptake in the infected plants leading to wilting, stunted growth, and reduced yields. Host plants respond to invading RKNs through a series of defense mechanisms by coordinating different signaling pathways. Host plants recognize pathogen-associated molecular patterns (PAMPs) derived from the pathogen by utilizing pattern recognition receptors (PRRs) that induce pattern-triggered immunity (PTI) or effector-triggered immunity (ETI) [1]. Expression of large numbers of downstream genes is triggered to regulate resistance-related pathways, including hormone signaling, transduction, and transcription factors (TFs) constituting a highly controlled regulatory network.

Cotton (*Gossypium* spp.) is an economically important crop providing natural fibers for the textile industry and cotton seed oil for the production of food and biodiesel fuel. Several parasitic nematode species are known to infect cotton but the Southern root-knot nematode (*Meloidogyne incognita*, *RKN*) is the most important species because it is endemic to wherever cotton is grown, has a wide host range and is capable of inflicting economic losses through direct damage to the plant root system and indirectly through increasing severity of other root diseases such as Fusarium Wilt caused by *Fusarium oxysporum* f. sp. *vasinfectum*. Yield loss due to RKN has dramatically increased in the U.S. from 1% in 1987 to 5.5% in 2006, resulting in losses of more than 136 million kilograms (4.4%) of cotton valued over $235 million (Cotton Disease Loss Estimate Committee Report 2012). Recommended management practices to control RKN include crop rotation and nematicide application. The broad host range of RKN leaves cotton growers with few profitable options to adopt crop rotation as a means of nematode management. Fields with threshold or greater levels of RKN require application of nematicides to minimize yield loss to RKN. Although nematicides are effective in controlling RKN, they do not provide season-long protection and their future availability is uncertain due to environmental concerns.

Host plant resistance, the ability of a plant to suppress nematode reproduction, is the most economical, practical, and environmentally sound method to provide a season long crop protection against RKN. A near immunity level of RKN resistance is available in the cotton germplasm ‘Auburn 623 RNR’, which originated from transgressive segregation in a cross between two moderately resistant parents, Clevewilt 6 and Wild Mexican Jack Jones [2]. The resistance was subsequently transferred to several agronomically adapted cultivars through backcrossing, resulting in the release of the M-line series including genotypes such as ‘M-120 RNR’, ‘M-315 RNR’, and ‘M-155 RNR’, with greatly improved agronomic qualities while retaining the almost-immune level of resistance of Auburn 623 RNR. By using the genotype M-315 RNR, Jenkins et al. [3] showed that resistance to RKN was due to a two-stage post-penetration interference with the first stage occurred soon (10–12 days) after infection which prevented feeding site development and the second stage occurred much later (25–30 days) after infection resulting in a significantly lowered egg production compared to a susceptible genotype. Genetic linkage mapping studies revealed that the resistance in this resistance source is largely controlled by two major QTLs, a locus on the long arm of chromosome 11 and another on the short arm of chromosome 14 [4–6]. Further studies have revealed that the locus on chromosome 11 affects root gall production while the locus on chromosome 14 largely affected RKN egg production with minimal effect on galling [5,7].

The objectives of this study were a) to explore global changes in gene expression at early (12 Days after inoculation; DAI) and late (30 DAI) stages of RKN infection in a RKN resistant and a susceptible genotype using the next generation sequencing based comparative transcriptomic approach, and b) to identify differentially expressing genes (DEGs) in the genomic regions of chromosome 11 and chromosome 14 that have previously been implicated to contain the resistant genes.

## Materials and methods

### Plant material

Two Upland cotton germplasm lines, M-120 RNR (M120) and Coker 201 (C201) with contrasting responses to *M*. *incognita* were used in the experiment. M120 displays a high level of resistance to both galling and RKN egg production while the susceptible genotype C201 is the recurrent parent of the resistant line [2]. Delinted cotton seeds of both genotypes were surface sterilized, germinated in vermiculite and grown for two weeks. Seedlings of similar size from both genotypes were then individually transplanted into a 10.6 cm x 10.6 cm x 12.4 cm pot filled with approximately 500 ml of steam-pasteurized soil (Tifton loamy sand). At transplant, seedlings were infected with 4,000 *M*. *incognita* J2 per plant. Inoculum was distributed into two holes about 2.5 cm deep in soil on opposite sides of the seedling and the holes were covered with soil after inoculation. Healthy seedlings of each genotype without inoculation served as treatment controls. Whole intact root system from inoculated and un-inoculated M120 and C201 plants were carefully removed from the potting-mix at 12 and 30 DAI, washed with de-ionized water, dried with sterilized paper towels and immediately frozen in liquid nitrogen. Frozen roots of individual plants were stored at -80°C until RNA extraction.

### Library construction and sequencing

Frozen root samples of individual plants from M120 and C201 collected from 12 and 30 DAI along with un-inoculated control of both genotypes collected at 12 and 30 DAI were individually crushed with a mortar and pestle in liquid nitrogen, and total RNA was extracted using Spectrum^™^ Plant Total RNA extraction kit (Sigma-Aldrich) following the manufacturer’s protocol. Quantity and quality of the extracted RNA was assessed by NanoDrop (Thermo Scientific). Sample from the infected and control plants of both the resistant and susceptible genotypes with a 260/280 and 260/230 ratios around 2.0 were selected for library construction. Equal concentration of RNA from two individual plants from M120 and C201 at 12 and 30 DAI were utilized for cDNA library constructions. For both genotypes, the cDNA libraries constructed from plants at 12 DAI were designated as early sampling date (E), at 30 DAI designated as late sampling date (L). Similarly, for both genotypes, a cDNA library designated as control (C) was also developed from pooling equal concentration of RNA from three un-inoculated plants each sampled at 12 and 30 DAI. All libraries were constructed using the NEBNext Ultra RNA library Prep Kit for Illumina (New England BioLabs Inc.) following the manufacturer’s protocol. The NEBNext Multiplex Oligos for Illumina (Index Primers Set 2, New England BioLabs Inc.) containing adaptors and primers were used to label libraries facilitating pooling. Libraries were submitted to the Georgia Genomic Facility, University of Georgia for sequencing using 150 bp paired-end Illumina NextSeq 300 High Output flow cell platform.

### Transcriptome assembly, and differential expression

The qualities of the raw reads were checked using FastQC program [8]. Low-quality bases and adapter sequences from paired reads were trimmed using the Trimmomatic v0.30 program [9]. Trimmed reads were mapped to the *G*. *hirsutum* reference genome [10] downloaded from the cotton functional genomics database (https://cottonfgd.org) using HISAT2 v2.1.0 (https://ccb.jhu.edu/software/hisat2/). Sequence alignment files were input into the software Stringtie v1.3.3 (http://ccb.jhu.edu/software/stringtie/) to assemble potential transcripts [11]. A python script was used to obtain gene-level raw counts from each library for differential gene expression analysis using the R package DESeq2 [12]. All calculated *P*-values were adjusted for multiple testing with Benjamini and Hochberg's approach using a false discovery rate (FDR) of 5% and genes were determined to be differentially expressing with FDR < 0.05.

cDNA libraries from RKN infected M120 and C201 plants at early and late sampling dates were compared to their respective controls to identify up and down regulated genes. In addition, the libraries of infected M120 and C201 plants at early and late sampling dates were compared to each other to identify genotype specific DEGs and estimate the log_2_ Fold Change (FC). Genes were considered to have a significant difference in gene expression when their relative expression levels showed at 4-FC (log_2_ FC >2.0 or < -2.0) difference between infected and control samples, with *P*-value<0.05. Clustering of the significant DEGs with similar expression patterns was performed by the Multiexperiment Viewer v4.9.0 (http://mev.tm4.org/) using K-means clustering and Pearson’s correlation coefficient.

### Real-time qPCR

Primers for target genes in the QTL regions and an endogenous control gene were designed with Primer3Plus [13]—available at https://primer3plus.com/cgi-bin/dev/primer3plus.cgi - and synthesized by Eurofins Genomics US. Reverse transcription (RT) for cDNA synthesis was done using 4 μL iScript Reverse Transcription Supermix for RT-qPCR (bio-rad), ~1 μg total plant RNA, and nuclease-free water to constitute 20 μL final reaction volume. Amplification conditions were: 5 min at 25°C for priming, 20 min at 46°C for RT, and 1 min at 95°C for RT inactivation. Two biological replicates of samples corresponding to early and late infection time-points were used in 10 μl qPCR reactions containing: 5 ml of SsoAdvanced^™^ Universal Inhibitor-Tolerant SYBR^®^ Green Supermix (bio-rad), 0.25 μM of each primer, 2 μl of diluted cDNA (corresponding to 20 ng of total RNA), and 2.5 μl nuclease-free water. Three technical replicates were used in qPCR reactions. StepOnePlus Real-Time PCR System (ThermoFisher Scientific) was run with the following amplification conditions: hot start of 3 min at 98°C followed by 40 cycles of 15 s at 98°C and 60 s at 58–60°C when the raw fluorescence data was recorded. After 40 cycles, melting curves were estimated from 60°C to 95°C, with a plate reading step at 0.3°C increments. Raw fluorescence data was exported to comma separated files (.csv). The fractional cycle number at threshold (CT) values and primer efficiencies were estimated using Miner [14] v4.0 (http://miner.ewindup.info/). Relative gene expression and their significance were estimated through 2,000 iterations of CT, using REST 2009 [15] v.1 (http://rest.gene-quantification.info/).

### Gene enrichment analysis

Gene list enrichment analysis is done by PANTHER Overrepresentation Test (http://geneontology.org/, Released 20171205) using Fisher's Exact with FDR multiple test correction (FDR<0.05). Kyoto Encyclopedia of Genes and Genomes (KEGG) pathway enrichment analysis of the DEGs was performed using the Database for Annotation, Visualization and Integrated Discovery (DAVID) v6.8 (https://david.ncifcrf.gov) with Uniprot IDs associated with these genes obtained from the cotton functional genomics database (https://cottonfgd.org). GO terms with P < 0.05 were considered significantly enriched.

## Results

### Transcriptome sequencing

**Fig 1:**
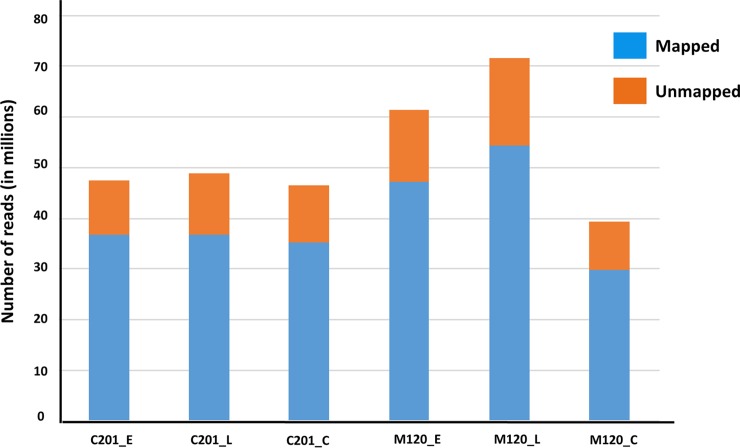
Average number of mapped and unmapped sequences at early (E), late (L) and control (C) sampling time point.

To gain insight into the transcriptomic changes in Upland cotton roots as the nematode infection progresses, cDNA libraries of resistant M120 and susceptible C201 plants were sequenced at early (12 DAI) and late (30 DAI) stages of nematode infection. After trimming adapters and low quality reads, the number of reads generated ranged from 39.3 million reads in M120-C library to 47.2 million reads in M120-E library. Of the 315 million total reads obtained (157.5 million pair ends), 240.54 million or 76.3% were mapped to the *G*. *hirsutum* reference genome from cotton functional genomics database (Fig 1).

### Differential gene expression and functional analysis

Differential expression of genes in M120 and C201 (FDR <0.05) were evaluated using DESeq2 package that performs variance estimation and differential expression of the raw read count between infected and control libraries at each sampling point.

**Fig 2:**
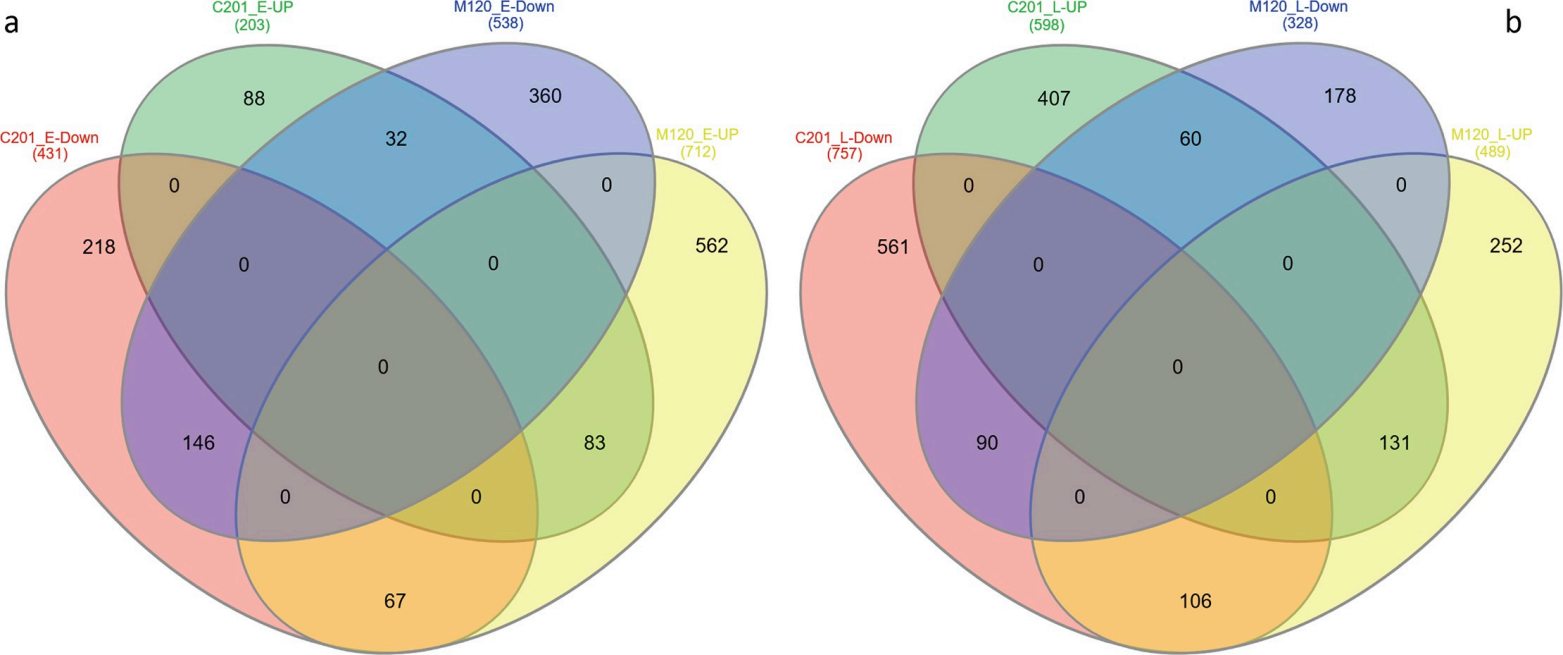
Venn diagram illustrating differentially expressed genes in C201 and M120 at (a) early (b) late stage of RKN infection.

A total of 3,789 genes were found to be differentially expressed at the set threshold of *P*-value<0.05. Comparisons of the up and down regulated genes at early and late sampling stage are presented in a Venn diagram (Fig 2). In total there were 1,884 (49.7%) differentially expressed genes at the early stage of infection and a total of 2,172 (57.3%) at the later stage of RKN infection. The resistant genotype, M120, had greater number (1,250) of differentially expressing genes at early stage of RKN development whereas the susceptible genotype C201 had greater number (1,355) of differentially expressing genes at later stage of RKN development (Fig 2) suggesting that the host plant response to RKN infection and development is different between the two genotypes.

**Fig 3:**
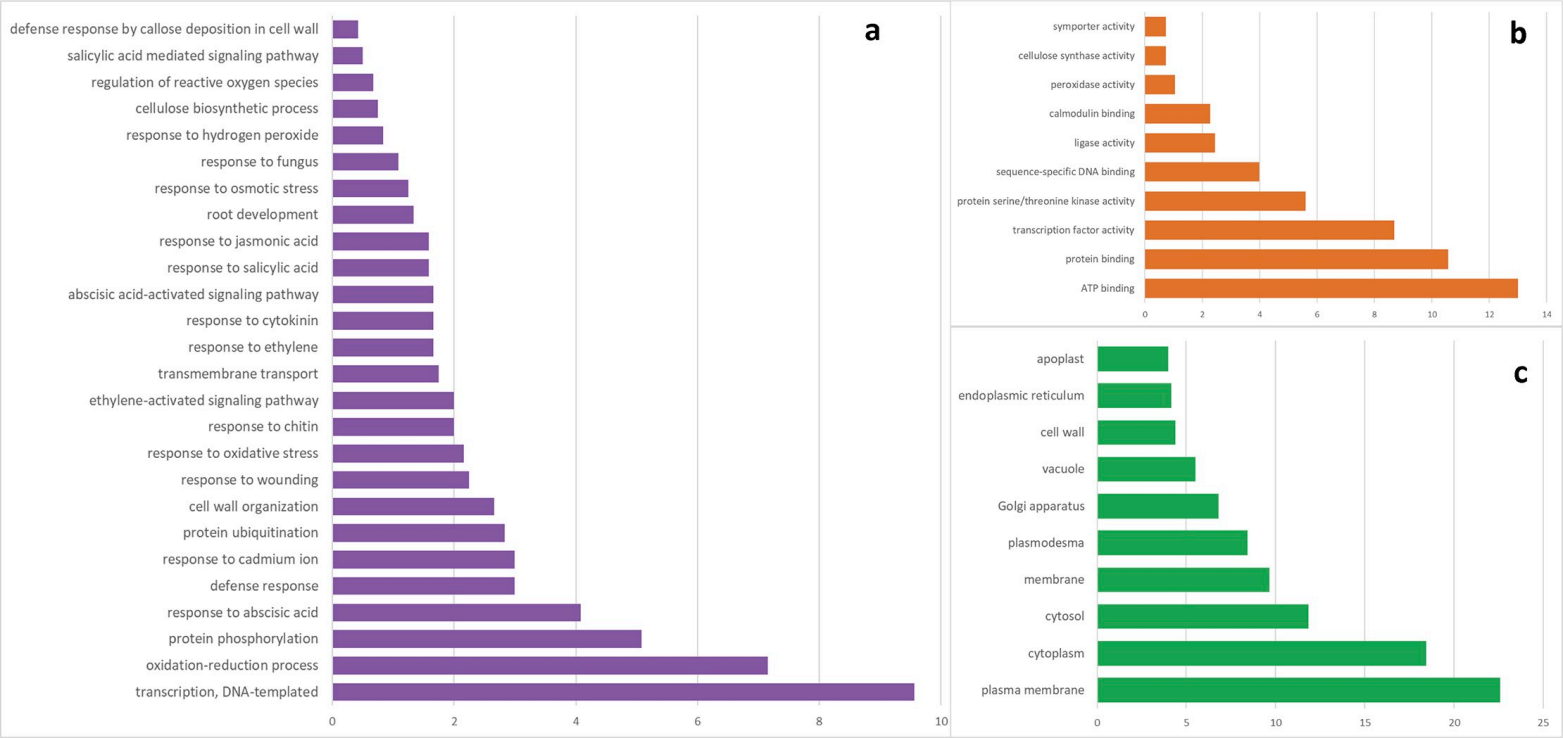
GO enrichment terms. (a) biological process, (b) molecular function, (c) Cellular components.

GO enrichment analysis of the annotated DEGs revealed several enriched biological processes, molecular functions and cellular components (Fig 3). Functional analysis of DEGs identified at the early sampling stage revealed that the key enriched biological processes include protein ubiquitination (GO:0016567), defense response (GO:0006952), response to phyto-hormones such as salicylic acid (GO:0009751), abscisic acid (GO:0009737), auxin (GO:0009733), ethylene (GO:0009873), response to chitin (GO:0010200) and cell wall organization (GO:0071555). The key molecular functions enriched included transcription factor activity (GO:0003700), protein serine/threonine kinase activity (GO:0004674), kinase activity (GO:0016301), and calmodulin binding (GO:0005516) (Fig 3). The most enriched cellular components group included plasma membrane (GO:0005886), plasmodesma (GO:0009506), and cell wall (GO:0005618). KEGG pathway analysis showed that plant-pathogen interaction (ath04626), plant hormone signal transduction (ath04075), ubiquinone and other terpenoid-quinone biosynthesis (ath00130) were also enriched.

### Differential gene expression between resistance and susceptible genotypes

To estimate on the number of genes expressing differentially between M120 and C201 at early and late stages of nematode infection, we performed a comparative analysis using the DESeq2 package at the preset threshold of fold change (FC) >2.0 or < -2.0 and false discovery rate (FDR) <0.05. Comparative analysis of libraries at early stage of nematode infection (M120-E vs C201-E) identified 1,313 DEGs where a positive log_2_FC indicates higher relative expression of genes in M120 while negative log_2_FC indicates relatively higher expression of genes in C201. A total of 454 (36%) DEGs had higher expression in C201 while 859 (64%) had higher expression in M120. Similarly, a total of 1,555 DEGs were identified at the late stage of nematode infection (C201-L Vs M120-L), and of these 658 (42%) had a greater relative expression in C201 (negative log_2_FC) while 897 (58%) DEGs had a greater relative expression level in M120 (positive log_2_FC).

### Cluster analysis

**Fig 4:**
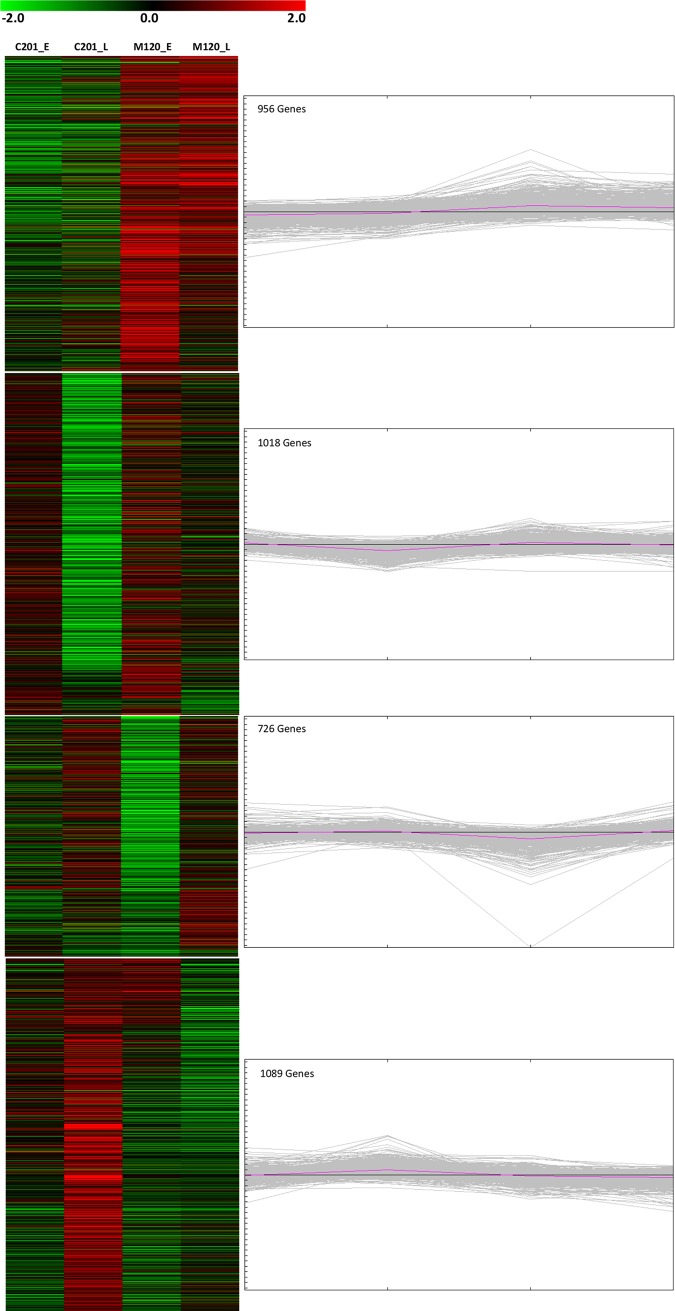
Cluster analysis of the identified differentially expressing genes.

All the DEGs between treated and untreated libraries were grouped into four clusters based on the expression profiles using k-mean algorithm (S1 Table; Fig 4). The first cluster included 956 genes predominately up regulated at early stages of RKN infection in M120 (S1 Table). Expression levels of several of these genes were up regulated in M120-L but were down regulated in C201-E and C201-L. This cluster included over 60 genes involved in host disease response such as MYB transcription factors, genes in auxin (TIFY 9) and ethylene-activated signaling pathway (ERF13), genes activated in response to salicylic acid (syntaxin and callose synthase), and calmodulin binding genes (S1 Table). The second cluster consisted of 1,018 RKN responsive genes that were up regulated at early stages of RKN development in C201 and M120 but were down regulated in C201-L (Fig 4). This cluster included over 50 disease responsive genes, genes associated with host plant hypersensitive response such as endochitinase (EP3) flavin-containing monooxygenase (FMO1), genes involved in salicylic acid mediated signaling pathway and systemic acquired resistance (SAR) (S1 Table). The third cluster consisted of 726 RKN responsive genes up regulated at later stages of RKN infection in both C201 and M120 (S1 Table). The expression level of the majority of these genes were down regulated at early stages of RKN infection in C201. A majority of the up regulated DEGs were associated with auxin-activated signaling pathway and DNA-template transcription for controlling vegetative and floral developmental stages. The fourth cluster consisted of 1,089 RKN responsive genes that were up regulated in C201-L. This cluster included several cell wall organization genes like cellulose synthase, endoglucanase, galacturonosyltransferase, and xylose synthase along with genes involved in the lignin biosynthetic process such as laccase, caffeoylshikimate esterase. Other genes in this cluster were associated with biological processes such as response to water deprivation, response to osmotic stress, unidimensional cell growth and auxin or ethylene activated signaling pathways.

### Transcription factors analysis

**Fig 5:**
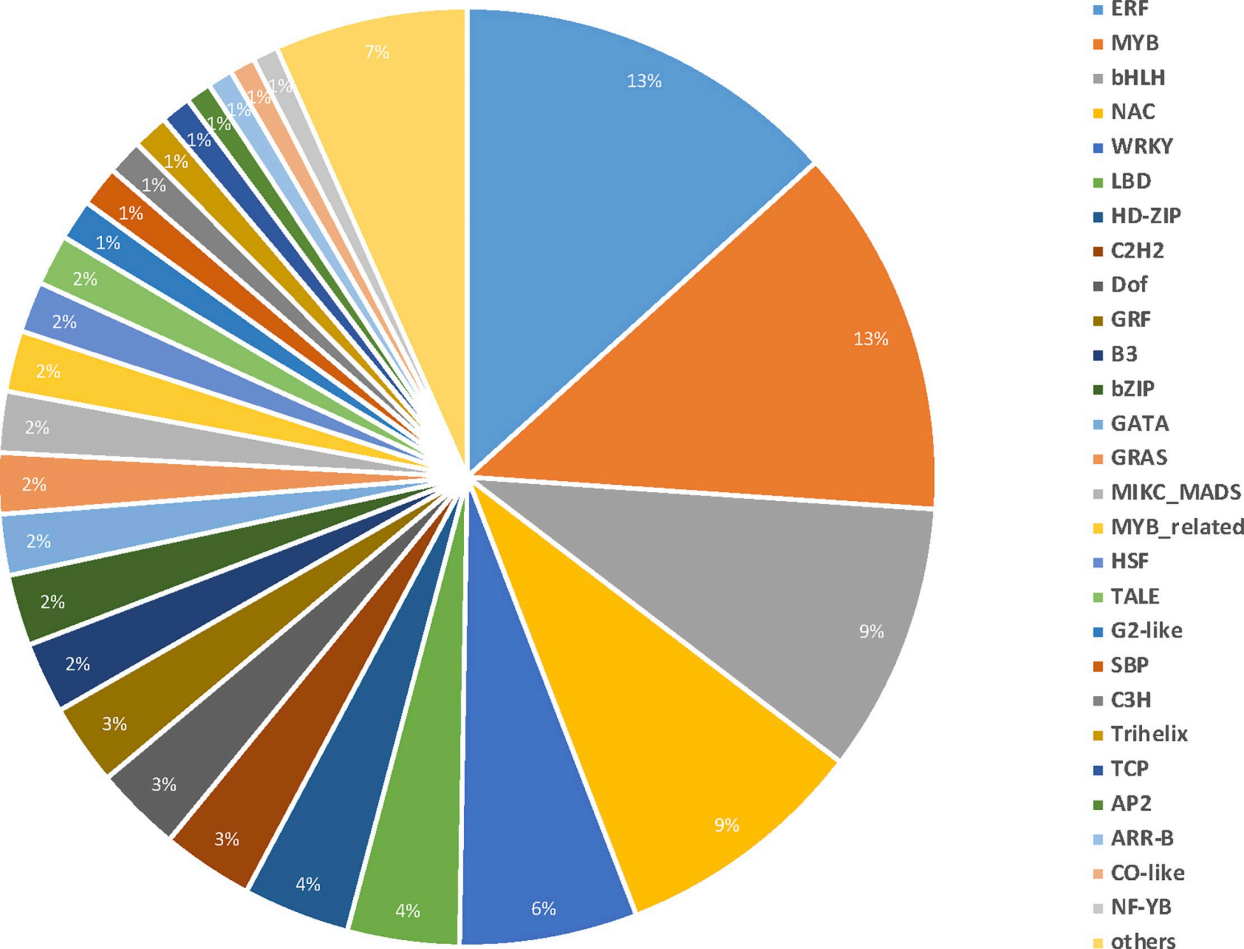
Distribution of *G*. *hirsutum* transcription factors families during RKN infection and development.

Transcription factors (TFs) are vital components of the host molecular response to invading pathogens as they control gene transcriptions by binding to *cis*-acting elements in the promoters of their target genes. TFs can positively and negatively regulate downstream defense responsive genes in response to biotic and abiotic stresses. The Plant Transcription Factor Database (planttfdb.cbi.pku.edu.cn) hosts over 5,000 *G*. *hirsutum* transcription factors that have been classified into 58 families. In this study, 571 TF’s belonging to 42 families displayed differential expression in response to RKN infection (Fig 5). The most abundantly expressing TF families were ERF (13%), MYB (13%), bHLH (9%), NAC (9%), WRKY (6%), and C2H2 (3%). These TFs are integral parts of the signaling networks that modulate many biological processes including host response to pathogens, regulation of pathogen resistance, and cell death. Enhanced activity of these TFs suggests possible role in regulating genes in multiple pathways through cis-acting sequences in response to RKN infection.

### Differentially expressing genes at the QTL loci regions

The physical location of the QTL regions on chromosome 11 and 14 were identified by BLAST searching the *G*. *hirsutum* genome sequence using clone sequences from which the flanking SSR markers CIR316 and CIR069 demarcating region on chromosome 11 and markers BNL3644 and NAU5467 demarcating region on chromosome 14 were developed. We included chromosome D11, homeologs of chromosome 11 (A11) and chromosome A02, homeolog of chromosome 14 (D02) in searching for DEGs. A total of 36 DEGs were identified between C201-E and M120-E libraries while a total of 39 DEGS were identified between the C201-L and M120-L libraries (Table 1). Eighteen DEGs were common between the two stages of sampling, which included genes encoding for Glutathione S-transferase (Gh_A02G0247, Gh_A02G0260), Callose synthase (Gh_A02G0303, Gh_D02G0367), Putative disease resistance protein (Gh_A11G2835), LRR receptor-like protein kinase (Gh_A02G0272), Wall-associated receptor kinase (Gh_D02G0201), and heat shock protein (Gh_D02G0378), emphasizing the role of these QTL loci in detoxification, stress response, disease resistance, and cell wall modification in response to RKN infection and development.

**Table 1:** Differentially expressing genes in the QTL region between M120 and C201 at early and late stages of RKN infection.

Stage	Genes	Log2FC	Gene Name	Chr	Start	Strand	Length (bp)
Early	Gh_A02G0103	1.13	Zinc finger BED domain-containing	A02	935,425	-	9,193
			protein RICESLEEPER 2				
Early	Gh_A02G0172	2.01	Cannabidiolic acid synthase-like 1	A02	1,919,267	-	1,614
Early	Gh_A02G0194	1.06	Receptor-like protein 12	A02	2,168,659	+	1,496
Early	Gh_A02G0224	-2.98	Cytochrome P450 CYP749A22	A02	2,685,528	+	1,391
Early	Gh_A02G0247	3.38	Glutathione S-transferase U7	A02	3,054,421	-	1,987
Early	Gh_A02G0260	1.01	Glutathione transferase GST 23	A02	3,129,950	-	24,845
Early	Gh_A02G0262	1.09	Glutathione transferase GST 23	A02	3,167,292	-	3,890
Early	Gh_A02G0272	-7.79	Leucine-rich repeat receptor-like protein	A02	3,245,923	-	1,560
			kinase At2g33170				
Early	Gh_A02G0301	2.26	Anthocyanidin 3-O-glucosyltransferase 5	A02	3,591,480	+	1,437
Early	Gh_A02G0303	1.58	Callose synthase 12	A02	3,607,023	+	5,141
Early	Gh_A11G2761	-2.39	UPF0481 protein At3g47200	A11	90,312,119	-	2,202
Early	Gh_A11G2782	-1.09	AP2-like ethylene-responsive	A11	90,835,335	-	5,639
			transcription factor PLT2				
Early	Gh_A11G2835	6.58	Putative disease resistance protein RGA3	A11	91,808,765	+	2,439
Early	Gh_A11G2836	-2.36	Putative disease resistance RPP13-like	A11	91,814,126	-	2,221
			protein 1				
Early	Gh_A11G3073	1.30	bZIP transcription factor 53	A11	40,812	+	429
Early	Gh_A11G3090	2.21	U-box domain-containing protein 21	A11	2,392	+	1,323
Early	Gh_A11G3216	4.04	Cytochrome P450 CYP73A100	A11	28,401	-	1,080
Early	Gh_A11G3286	1.92	Pentatricopeptide repeat-containing	A11	7,299	-	1,251
			protein At3g14580				
Early	Gh_D02G0201	3.82	Wall-associated receptor kinase-like 1	D02	2,253,878	-	4,364
Early	Gh_D02G0214	1.67	Cannabidiolic acid synthase-like 1	D02	2,443,413	-	6,549
Early	Gh_D02G0215	1.47	Cannabidiolic acid synthase-like 1	D02	2,457,745	-	1,608
Early	Gh_D02G0257	1.25	Receptor-like protein 12	D02	2,942,631	+	3,456
Early	Gh_D02G0259	1.42	Receptor-like protein 12	D02	2,979,373	+	3,918
Early	Gh_D02G0264	1.25	Probable inorganic phosphate transporter	D02	3,084,374	+	1,590
			1–5				
Early	Gh_D02G0287	1.10	Cytochrome P450 CYP749A22	D02	3,726,880	+	74,034
Early	Gh_D02G0367	1.56	Callose synthase 12	D02	4,871,474	+	5,142
Early	Gh_D02G0378	1.01	22.7 kDa class IV heat shock protein	D02	4,975,599	+	564
Early	Gh_D11G3141	-1.81	Remorin	D11	63,905,019	+	2,572
Early	Gh_D11G3171	-1.18	Dirigent protein 21	D11	64,342,794	+	453
Early	Gh_D11G3192	-1.80	Putative disease resistance protein RGA4	D11	64,700,262	+	3,630
Early	Gh_D11G3321	-1.31	UPF0503 protein At3g09070,	D11	66,015,529	+	603
			chloroplastic				
Early	Gh_D11G3369	1.28	TMV resistance protein N	D11	6,267	+	5,104
Early	Gh_D11G3379	-3.38	Dirigent protein 15	D11	2,301	-	585
Early	Gh_D11G3383	2.46	LRR receptor-like serine/threonine-	D11	715	-	2,904
			protein kinase GSO1				
Early	Gh_D11G3412	-1.40	NAD-dependent malic enzyme 2,	D11	79,319	-	643
			mitochondrial				
Early	Gh_D11G3471	5.84	Probable prolyl 4-hydroxylase 7	D11	10	-	1,402
Late	Gh_A02G0172	3.84	Cannabidiolic acid synthase-like 1	A02	1,919,267	-	1,614
Late	Gh_A02G0247	-1.01	Glutathione S-transferase U7	A02	3,054,421	-	1,987
Late	Gh_A02G0259	-1.51	Glutathione S-transferase U7	A02	3,123,568	-	985
Late	Gh_A02G0260	2.49	Glutathione transferase GST 23	A02	3,129,950	-	24,845
Late	Gh_A02G0272	-1.03	Leucine-rich repeat receptor-like protein	A02	3,245,923	-	1,560
			kinase At2g33170				
Late	Gh_A02G0303	2.52	Callose synthase 12	A02	3,607,023	+	5,141
Late	Gh_A02G0339	-1.15	CBL-interacting serine/threonine-protein	A02	4,015,866	+	1,389
			kinase 7				
Late	Gh_A11G2820	-1.93	Probable E3 ubiquitin-protein ligase	A11	91,539,168	-	3,612
			BAH1-like				
Late	Gh_A11G2835	2.01	Putative disease resistance protein RGA3	A11	91,808,765	+	2,439
Late	Gh_A11G3073	1.12	bZIP transcription factor 53	A11	40,812	+	429
Late	Gh_A11G3090	1.80	U-box domain-containing protein 21	A11	2,392	+	1,323
Late	Gh_A11G3117	-1.06	Probable trehalose-phosphate	A11	403,630	+	1,774
			phosphatase J				
Late	Gh_A11G3216	2.22	Cytochrome P450 CYP73A100	A11	28,401	-	1,080
Late	Gh_A11G3289	2.27	Probable receptor-like protein kinase	A11	54,944	+	3,811
			At1g67000				
Late	Gh_D02G0196	-1.07	Wall-associated receptor kinase-like 8	D02	2,184,616	-	2,577
Late	Gh_D02G0201	1.45	Wall-associated receptor kinase-like 1	D02	2,253,878	-	4,364
Late	Gh_D02G0213	1.20	Tetrahydrocannabinolic acid synthase	D02	2,424,564	-	1,614
Late	Gh_D02G0216	2.10	Cannabidiolic acid synthase-like 1	D02	2,506,487	-	1,623
Late	Gh_D02G0217	1.14	Cannabidiolic acid synthase-like 1	D02	2,580,689	-	1,623
Late	Gh_D02G0218	1.08	Not available	D02	2,612,216	-	1,440
Late	Gh_D02G0225	1.47	Crocetin glucosyltransferase,	D02	2,650,442	-	1,410
			chloroplastic				
Late	Gh_D02G0227	2.56	Crocetin glucosyltransferase,	D02	2,674,235	+	1,410
			chloroplastic				
Late	Gh_D02G0229	3.34	Crocetin glucosyltransferase,	D02	2,690,560	+	1,410
			chloroplastic				
Late	Gh_D02G0256	1.22	Probable magnesium transporter NIPA2	D02	2,926,827	+	7,957
Late	Gh_D02G0257	3.77	Receptor-like protein 12	D02	2,942,631	+	3,456
Late	Gh_D02G0259	1.62	Receptor-like protein 12	D02	2,979,373	+	3,918
Late	Gh_D02G0264	3.21	Probable inorganic phosphate transporter	D02	3,084,374	+	1,590
			1–5				
Late	Gh_D02G0265	-1.36	Probable inorganic phosphate transporter	D02	3,092,123	+	1,596
			1–5				
Late	Gh_D02G0292	-2.40	Not available	D02	4,031,391	-	2,031
Late	Gh_D02G0297	-1.24	Not available	D02	4,064,414	+	3,707
Late	Gh_D02G0367	1.71	Callose synthase 12	D02	4,871,474	+	5,142
Late	Gh_D02G0378	-1.62	22.7 kDa class IV heat shock protein	D02	4,975,599	+	564
Late	Gh_D02G0387	-1.39	Homocysteine S-methyltransferase 3	D02	5,016,407	-	3,693
Late	Gh_D11G3175	-2.14	Probable E3 ubiquitin-protein ligase	D11	64,358,944	-	3,822
			BAH1-like				
Late	Gh_D11G3379	4.02	Dirigent protein 15	D11	2,301	-	585
Late	Gh_D11G3381	-1.31	Not available	D11	31,491	-	2,243
Late	Gh_D11G3383	4.87	LRR receptor-like serine/threonine-	D11	715	-	2,904
			protein kinase GSO1				
Late	Gh_D11G3412	3.85	NAD-dependent malic enzyme 2,	D11	79,319	-	643
			mitochondrial				
Late	Gh_D11G3494	1.01	Putative disease resistance protein RGA1	D11	93,132	+	4,071

### qPCR of candidate genes at the QTL regions

**Fig 6:**
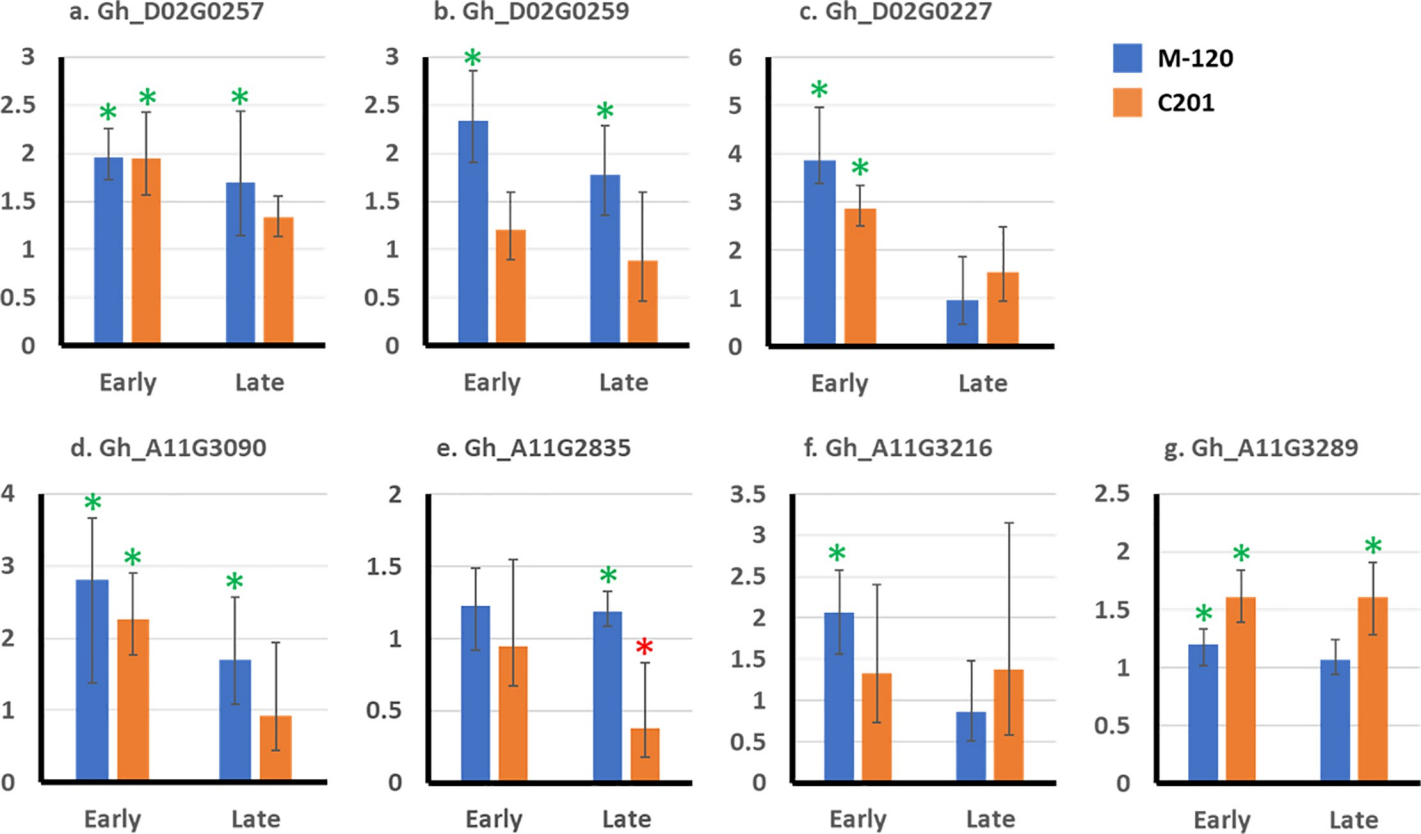
Quantitative real-time PCR (qRT-PCR) of candidate root-knot nematode (RKN) resistance genes at early and late infection time-points. Charts show mean fold-change in expression after RKN infection with standard error bars. *Green* asterisks mark significant upregulation and *red* asterisks mark significant down regulation in expression in inoculated samples compared to non-inoculated plants and as determined by t-test of ΔCt values (P ≤ 0.05) using two biological replicates.

A total of 12 DEGs primarily involved in defense-related functions, six from each of the two QTL regions on chromosomes 11 (with an exception of a homoeologous TMV resistance gene Gh_D11G3369) and 14 were selected for further qPCR analysis. Detailed information on the target genes, endogenous control, and PCR primers are provided in S2 Table. Relative gene expression (normalized against endogenous *GhACT4*) between nematode treated vs. control samples at early and late time-points showed that 11 out of 12 targets were significantly overexpressed in at least one treatment-genotype combination (Fig 6, S1 Fig, and S3 Table). For example, five of the six target genes on chromosome 14 such as Gh_D02G0257 and Gh_D02G0259 were significantly overexpressed in response to nematode infection, with Gh_D02G0259, a receptor kinase gene, showing significant overexpression only in the resistant M120 genotype at both stages of nematodes development (Fig 6B). Similarly, five of the six target genes chromosome 11 and a TMV resistant gene mapped to homoeologous region in chromosome 21 were significantly overexpressed in response to nematode infection. It is noteworthy that two colocalized putative disease resistance genes viz. Gh_A11G2835 and Gh_A11G2836 showed different directions of expression in response to nematode infection (Fig 6 and S1 Fig), which is collaborate with the expression results from RNA-seq (Table 1 and S1 Table). Specifically, RNA-seq showed significantly overexpression of Gh_A11G2835 in the resistant genotype at both stages of nematodes development, while qPCR showed late-stage specific overexpression in M120 with significant down expression in C201. However, Gh_A11G2836 was significantly overexpressed in the susceptible genotype C201 at the early stage in RNA-seq and at both stages in qPCR, making the colocalized pair the most likely candidate genes conditioning different disease reaction in the two genotypes.

## Discussion

The Auburn 623 RNR germplasm represents the most important source of resistance to RKN in cotton. Histopathology studies have shown a two-stage mechanism in conferring RKN resistance in this germplasm source [3] and QTL analyses collaborated with this observation with the identification of two resistance loci, one on chromosome 11 which had a strong effect on root gall suppression while the second locus on chromosome 14 had a strong effect on impeding egg production but little effect on galling [4–6]. These results not only lend credence to the hypothesis that resistance in Auburn 623 is conferred by two genes with different defense response strategy against nematode attack but also suggest that the mechanism of resistance for the *qMi-C11* locus occurs in the early defense reaction (~8–12 days post penetration) preventing juvenile nematodes from developing functional feeding sites to form giant cells and the *qMi-C14* locus involves in the later defense mechanism (~25–30 days post penetration) whereby impeding the development of nematodes into eggs laying adult females. Therefore, analyzing the transcriptome level difference between M120 and C201 to identify unique alterations in gene expression at early and late stages of RKN development will narrow the lists of candidate genes for q*Mi-C11* and q*Mi-C14* to further facilitate in elucidating the mechanisms of resistance.

### Transcriptomic changes at early stage of infection

At early stages, expression of defense response genes was up regulated in both genotypes, however the magnitude of upregulation was several-fold greater in M120. Expression levels of genes that trigger early plant defenses and hypersensitive responses such as the endochitinase EP3, disease resistance protein RPM1, and protein EDS1L were higher in M120 compared to C201. Many defense-responsive genes were specific to M120 (grouped in cluster-1) and were up regulated at both early and late stages suggesting their role in constitutive M120 specific resistance. For example, ethylene-responsive genes, ERF12, ERF13, ERF1B and transcription factors such as MYB108, and TIFY9 were expressed at higher levels in M120-E and M120-L. Genes belonging to ethylene-responsive transcription factor superfamily have played a pivotal role in adaption to biotic stresses by activating pathogenesis-related (PR) genes [16]. Together these results suggest that the durable host plant resistance in M120 may not only be due to activation of M120-specific defense response genes but also by multifold increase in expression of defense genes common between resistant and susceptible genotypes.

Plant hormones play a pivotal role in regulating plant growth and development, and coordinate host plant response to biotic stresses. Genes activated in response to plant hormones like auxins and cytokinins were up regulated in C201. Auxin responsive genes (IAA) such as IAA20, Indole-3-acetic acid-amido synthetase (GH3.6), and Dof zinc finger protein (DOF3.4) had up to 6-fold higher expression in C201 at the early stage of infection. Our findings are in genotype with previous reports that the increased activity of auxin responsive genes during the early stage of infection in susceptible plants have a role in nematode feeding site development [17]. *In silico* analyses of nematode-responsive genes have shown that accumulation of auxin in response to nematode infection may also result in activation of genes in other pathways critical for nematode feeding site development [17, 18]. Genes involved in cytokinin metabolic processes were also up regulated in the susceptible genotype. We detected increased activity of cytokinin dehydrogenase genes (CKX6, and CKX7) in C201, which agrees with the fact that the nematodes activate cell division by manipulating production and signaling of cytokinins [19]. Expressions of cytokinin dehydrogenase genes were found down regulated in M120, which is in contrast to findings in Arabidopsis where a transgenic line over expressing cytokinin oxidase 2 (*35S*:*CKX2*) gene produced fewer galls and eggs per plant [20]. The precise role of cytokinins and cytokinin-oxidase genes in plant-nematode interaction remains obscure.

### Transcriptomic changes at later stage of infection

At the late stage of nematode development, expression levels of the genes related to plant cell wall organization and lignin modification were up regulated in C201 (cluster-4). For example, genes like cellulose synthase, xyloglucan endotransglycosylase, endoglucanase, galacturonosyltransferase, xylose synthase, laccase, and caffeoylshikimate esterase had multifold increased activity in C201. Studies have shown that nematodes enlarge their feeding site by up-regulating plant genes encoding for proteins that promote cell wall loosening like endoglucanases, pectinases, and expansins [21–23]. Further expansion of cells is achieved thru structural changes in cell wall xyloglucans which are the major components of hemicellulose and play crucial roles in cell wall flexibility [24]. The Xyloglucan endo-transglycosylase/hydrolase (XTH) genes in plants are primarily responsible for cell wall reinforcement after cell expansion is complete [25]. In the susceptible C201, xyloglucan endo-transglycosylase/hydrolase (XTH9) and expansin (EXPA5, EXPA17, EXLA1) gene expression levels were twice that in M120, suggesting rapid modification and restructuring of cell walls in susceptible genotypes. Higher expression of genes for callose deposition (CALS12) and phenylalanine ammonia-lyase (PAL), an enzyme in the lignin biosynthetic pathway were also detected in the resistant M120. Lignin and callose deposition in roots strengthen the plant resistance to invading nematodes [26, 27] by limiting the accessibility of cell wall-degrading enzymes secreted by nematodes during penetration and migration in plant roots [28].

Increased activity of detoxification genes was observed at the later stage. While the expression of glutathione S-transferase (GSTU7, GSTU8, GSTU17, GSTL3) genes involved in the conjugation of reduced glutathione to a wide number of exogenous and endogenous hydrophobic electrophiles was up regulated in both genotypes, the expression of laccase genes (LAC7, LAC14), which are involved in lignin catabolism and detoxification, had elevated expression only in the resistant genotype. Laccase genes along with peroxidase genes catalyze polymerization of monolignols to yield lignin, which plays an integral role in the defense response in several crops like cabbage [29], wheat [30], and maize [31]. In soybean a set of five laccase genes were found at *rhg1* locus that provided resistance against soybean cyst nematode (SCN; *Heterodera glycines*) and it was suggested that *LAC* genes may interact with other detoxifying genes and defense related genes to provide enhanced SCN resistance [32, 33].

### Transcriptomic changes at QTL loci

Genes differentially expressed at the QTL interval of q*Mi-C11* and q*Mi-C14* are of special interest in this study because of their physical position in the genome. In a previous study, we have reported the candidate genes for both QTL regions including 20 LRR genes in which SNPs were detected between a resistant and a susceptible genotype [7]. The 75 DGES found in the QTL regions primarily included genes involved in disease resistance, cell wall modification, production/metabolism of secondary metabolites and detoxification pathways.

Plant resistance gene analogs (RGAs), have conserved domains and motifs that play specific roles in host plant resistance [34]. Three resistance gene analogs (RGA1, RGA3, RGA4) at the QTL loci were overexpressed in response to RKN infection. Expression of RGA1 was confined to the late infection stage in M120 whereas RGA4 was induced in C201 at the early stage, however RGA3 was overexpressed in M120 at both early and late stages. At early stage of infection, expression levels of the gene for putative disease resistance protein (RGA3; Gh_A11G2835) was more than 6 log_2_FC in M120 with sustained expression till later stage of infection at relatively lower levels (log_2_FC = 2.0). Although quantitatively overexpressed at both stages of infection, qPCR validated significance of late stage-specific overexpression of RGA3 in M120 with concurrent down expression in C201, making the target the most probable candidate gene in chromosome 11 QTL region. Receptor like proteins (RLP) are one of the most abundant RGAs in plants that are involved in disease resistance and in plant development [33]. Two RLP12 genes (Gh_D02G0257, Gh_D02G0259) at the QTL loci on chromosome 14 were identified as candidate genes in our previous research [7] and their overexpression in response to RKN infection in M120 was evident in both RNA-seq and qPCR studies, which strongly suggest their role as plant receptors in sensing nematode parasitism and activating signaling pathways to trigger innate immune response.

A set of callose synthase genes at the QTL region on chromosome 14 and its homeolog A02 were highly up regulated in the RKN resistant M120, particularly during late infection. Similar results were reported in rice, where overexpression of three callose deposition genes along with lignin biosynthesis genes was observed in the roots of RKN resistant plants post nematode infection [28]. These results suggest that lignin and callose deposition likely interfere with some aspect of nematode development. Similarly, enhanced expression of Glutathione S-transferase genes on chromosome A02 suggest that this gene family may act as antioxidant and may be instrumental in regulating/signaling of cell death during hypersensitive response. Elevated expression of GSTs along with glutathione peroxidase (GPX) and GSH were observed in resistant tobacco when challenged with TMV [35] and in resistant sunflower infected with downy mildew [36], however, the precise role of GSTs during RKN infection in cotton needs further validation.

Finally, two genes have been reported to play a role in RKN resistance in cotton. The MIC-3 gene family that encodes cotton-specific pathogenesis-related proteins was shown to suppress RKN egg production when overexpressed in an RKN-susceptible genotype [37]. However, no MIC genes were found in the *qMi-C14* region, precluding them as candidates for this QTL. Interestingly, the gene Gh_D02G0276 encoding for domesticated transposase from hAT-superfamily (RICESLEEPER 3), which we previously reported to be a candidate [7] has recently been proposed as the causal gene *qMi-C14* [38]. This gene belongs to the SLEEPER gene family and while it has been known to be essential for normal growth and development [39], its role in disease resistance is largely unknown. However, the homeolog of this gene (Gh_A02G0103) was differentially up regulated in M120 at early stage of infection and suppression of its expression by virus induced gene silencing in a resistant line resulted in a phenotype that was similar to a RKN susceptible genotype. Our qPCR results did not show significant up-regulation of Gh_D02G0275 (data not shown) in response to RKN infection.

In conclusion, transcriptome analysis of M120 and C201 at early and late stages of RKN infection indicates that the host response to RKN parasitism is complex and greatly different between the resistant and susceptible genotypes. Resistant plants induced expression of early defense response genes, detoxification genes, and callose and lignin deposition genes to resist invasion and restrict movement of RKN inside the roots, whereas the susceptible plants expressed genes that facilitated establishment of feeding sites and development of female RKN. We found that the resistance in M120 might not only be due to induction of genotype-specific genes but also to greater magnitude in expression of defense response genes that are common to both genotypes. Overall, the expression data shows that the two QTL loci may have only partial contribution to RKN resistance in M120 with a greater part of resistance due to complex interactions of genes in the M120 genome. Systematic gene co-expression network analysis along with exhaustive histopathological studies are required to identify prominent pathways and/or groups of genes responsible for near immunity to RKN in the Auburn 623 RNR source.

## Supporting information

S1 Fig Quantitative real-time PCR (qRT-PCR) of candidate root-knot nematode (RKN) resistance genes at early and late infection time-points. Charts show mean fold-change in expression after RKN infection with standard error bars. *Green* asterisks mark significant upregulation and *red* asterisks mark significant down regulation in expression in inoculated samples compared to non-inoculated plants and as determined by t-test of ΔCt values (P ≤ 0.05) using two biological replicates.
(TIF)

S1 Table Cluster analysis of differentially expressing genes between M120 and C201 at early (E) and late (L) stages of nematode infection. (DOCX)

S2 Table Target genes, endogenous control, and PCR primers used in the qPCR analysis. (DOCX)

S3 Table Relative gene expression in an early and late stage in M120 and C201 genotypes in response to root-knot infection. (DOCX)
