# Supplementary figures and images for: Transcriptome analysis of a nematode resistant and susceptible upland cotton line at two critical stages of Meloidogyne incognita infection and development
Source: PLoS One. 2019 Sep 10;14(9):e0221328. doi: 10.1371/journal.pone.0221328 (PMC6736245; doi:10.1371/journal.pone.0221328)

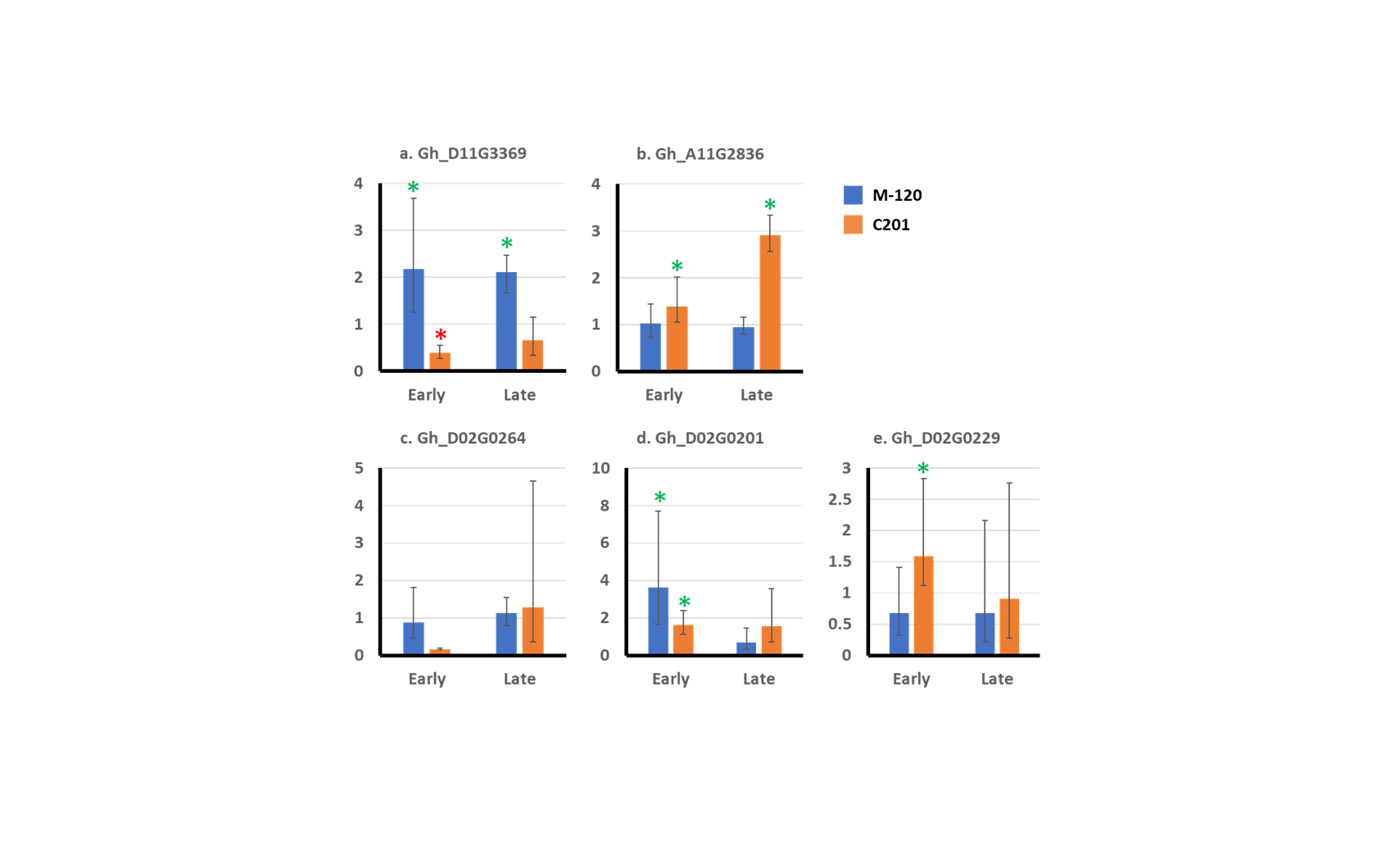

Supplement: S1 Fig — Charts show mean fold-change in expression after RKN infection with standard error bars. Green asterisks mark significant upregulation and red asterisks mark significant down regulation in expression in inoculated samples compared to non-inoculated plants and as determined by t-test of ΔCt values (P ≤ 0.05) using two biological replicates. (TIF) [file pone.0221328.s001.tif]
