# Supplementary material for: Transcriptome analysis of a nematode resistant and susceptible upland cotton line at two critical stages of Meloidogyne incognita infection and development
Source: PLoS One. 2019 Sep 10;14(9):e0221328. doi: 10.1371/journal.pone.0221328 (PMC6736245; doi:10.1371/journal.pone.0221328)
Supplement: S1 Table — (DOCX) [file pone.0221328.s002.docx]

| **Cluster** | **Gene ID** | **Gene Name** | **Description** | **Log2 Fold Change Compare to Controls** | | | |
| --- | --- | --- | --- | --- | --- | --- | --- |
|  |  |  |  | C201_E | C201_L | M120_E | M120_L |
| C_1 | Gh_A01G0009 | AC101 | Actin-101 | 0.1541 | 0.2525 | 1.2985 | -0.0536 |
| C_1 | Gh_A01G0065 | Zadh2 | Zinc-binding alcohol dehydrogenase domain-containing | 0.0417 | -0.1244 | 2.1210 | -0.0619 |
|  |  |  | protein 2 |  |  |  |  |
| C_1 | Gh_A01G0110 | TFT6 | 14-3-3 protein 6 | 0.3197 | -0.2288 | 4.5787 | -0.1603 |
| C_1 | Gh_A01G0125 | At5g63020 | Probable disease resistance protein | -0.2206 | -0.0482 | 1.2893 | 0.0278 |
| C_1 | Gh_A01G0153 | TIFY9 | Protein TIFY 9 | 0.2554 | -0.5164 | 2.2972 | 0.4852 |
| C_1 | Gh_A01G0248 | At1g56140 | Probable LRR receptor-like serine/threonine-protein | -0.3048 | -0.4116 | 0.7064 | -0.0042 |
|  |  |  | kinase |  |  |  |  |
| C_1 | Gh_A01G0383 | RLP12 | Receptor-like protein 12 | 0.3950 | 1.0811 | 1.7698 | 1.3882 |
| C_1 | Gh_A01G0432 | NA | NA | 0.0978 | 0.7901 | 0.6221 | 0.6390 |
| C_1 | Gh_A01G0475 | N | TMV resistance protein N | -1.4532 | -0.1155 | -0.3859 | 0.5493 |
| C_1 | Gh_A01G0489 | NCS2 | S-norcoclaurine synthase 2 | -2.8639 | 0.0926 | -0.0834 | 0.1072 |
| C_1 | Gh_A01G0498 | NA | NA | 0.1827 | -1.4955 | 2.0869 | 1.8087 |
| C_1 | Gh_A01G0540 | SLSG | S-locus-specific glycoprotein S13 | -1.0601 | -1.5987 | 2.2457 | 1.4733 |
| C_1 | Gh_A01G0553 | OCT4 | Organic cation/carnitine transporter 4 | 0.0973 | -0.3819 | 0.6392 | 0.5839 |
| C_1 | Gh_A01G0568 | GAUT12 | Probable galacturonosyltransferase 12 | -0.6429 | -0.9122 | 0.5445 | 0.8926 |
| C_1 | Gh_A01G0575 | UBQ10 | Polyubiquitin 10 | -0.0730 | -0.0577 | 0.2968 | -0.0689 |
| C_1 | Gh_A01G0632 | NA | NA | -0.9892 | -1.0587 | 2.0048 | 0.6933 |
| C_1 | Gh_A01G0637 | NPK1 | Mitogen-activated protein kinase kinase kinase NPK1 | 0.6097 | 0.9120 | 1.8200 | 1.3201 |
| C_1 | Gh_A01G0653 | At4g26790 | GDSL esterase/lipase | -3.7844 | 1.0381 | 1.6598 | -1.3366 |
| C_1 | Gh_A01G0762 | XERICO | Probable E3 ubiquitin-protein ligase XERICO | -0.2884 | -0.8220 | 0.8837 | 0.5553 |
| C_1 | Gh_A01G0763 | XERICO | Probable E3 ubiquitin-protein ligase XERICO | -0.3628 | -1.0218 | 0.7309 | 0.3876 |
| C_1 | Gh_A01G0840 | Tango2 | Transport and Golgi organization 2 homolog | -0.8939 | -0.1934 | 4.8707 | 2.5761 |
| C_1 | Gh_A01G1018 | NA | Squalene monooxygenase | -0.3206 | 0.5608 | 1.3865 | 0.9956 |
| C_1 | Gh_A01G1263 | PUB11 | U-box domain-containing protein 11 | 0.3723 | 0.8749 | 0.8611 | 0.7339 |
| C_1 | Gh_A01G1375 | LIMYB | L10-interacting MYB domain-containing protein | 0.3255 | -0.2266 | 0.3129 | 2.0646 |
| C_1 | Gh_A01G1418 | IREG2 | Solute carrier family 40 member 2 | -0.6082 | -0.8439 | 0.1553 | 0.1641 |
| C_1 | Gh_A01G1529 | NA | NA | 0.3502 | 0.3604 | 1.7958 | 1.1073 |
| C_1 | Gh_A01G1675 | DTX42 | Protein DETOXIFICATION 42 | -5.0455 | -2.5299 | -0.6565 | -0.2859 |
| C_1 | Gh_A01G1739 | NA | Secoisolariciresinol dehydrogenase | -0.0755 | -0.9894 | 0.5248 | 0.8833 |
| C_1 | Gh_A01G1800 | NA | NA | -1.0140 | 1.2089 | 1.0546 | 1.4547 |
| C_1 | Gh_A01G1839 | PAL | Phenylalanine ammonia-lyase | -0.2952 | -0.3709 | -0.0174 | 0.6622 |
| C_1 | Gh_A01G1870 | EXO70A1 | Exocyst complex component EXO70A1 | -2.1485 | -1.9853 | 0.8069 | 0.7512 |
| C_1 | Gh_A01G2011 | APUM7 | Putative pumilio homolog 7, chloroplastic | 0.2764 | -0.2565 | 1.7861 | 1.4216 |
| C_1 | Gh_A01G2138 | CCR4-1 | Carbon catabolite repressor protein 4 homolog 1 | -0.1541 | 0.2305 | 0.5333 | 0.3521 |
| C_1 | Gh_A01G2149 | GSTL3 | Glutathione S-transferase L3 | -0.2851 | -0.0301 | 0.8760 | 1.1461 |
| C_1 | Gh_A02G0013 | At2g38610 | KH domain-containing protein | -0.5052 | 0.2702 | 0.1112 | 0.1099 |
| C_1 | Gh_A02G0086 | NA | NA | -3.0002 | -1.6470 | 0.7316 | -0.6621 |
| C_1 | Gh_A02G0172 | CBDAS2 | Cannabidiolic acid synthase-like 1 | -1.9023 | -3.3014 | 0.1114 | 0.5368 |
| C_1 | Gh_A02G0247 | GSTU7 | Glutathione S-transferase U7 | -1.2805 | -0.4747 | 2.0967 | -1.4820 |
| C_1 | Gh_A02G0301 | GT5 | Anthocyanidin 3-O-glucosyltransferase 5 | -3.3047 | -0.0293 | -1.0491 | 0.2748 |
| C_1 | Gh_A02G0303 | CALS12 | Callose synthase 12 | -1.8111 | -2.5403 | -0.2320 | -0.0174 |
| C_1 | Gh_A02G0353 | NA | NA | 0.3862 | -0.6142 | 1.6105 | 2.7424 |
| C_1 | Gh_A02G0503 | NA | Major allergen Pru ar 1 | -0.6035 | -0.9198 | 1.4468 | -0.5791 |
| C_1 | Gh_A02G0509 | NA | Major allergen Pru ar 1 | -0.7469 | -1.1188 | 1.0788 | -0.8511 |
| C_1 | Gh_A02G0686 | ADH1 | Alcohol dehydrogenase 1 | 1.4918 | 0.9750 | 6.9088 | 5.0344 |
| C_1 | Gh_A02G0687 | ADH | Alcohol dehydrogenase | 0.5907 | 0.3450 | 5.9466 | 2.9895 |
| C_1 | Gh_A02G0727 | WAK2 | Wall-associated receptor kinase 2 | 0.0844 | -0.3579 | 1.8905 | 0.9306 |
| C_1 | Gh_A02G0749 | NAC002 | NAC domain-containing protein 2 | -0.7077 | -0.9875 | 0.1839 | 0.3667 |
| C_1 | Gh_A02G0878 | PFP-ALPHA | Pyrophosphate--fructose 6-phosphate 1- | 0.0476 | 0.6869 | 0.4924 | 0.5982 |
|  |  |  | phosphotransferase subunit alpha |  |  |  |  |
| C_1 | Gh_A02G0912 | At3g11710 | Lysine--tRNA ligase, cytoplasmic | -0.9291 | -0.4482 | -0.0337 | -0.1410 |
| C_1 | Gh_A02G0920 | CML42 | Calcium-binding protein CML42 | 0.2805 | -0.0119 | 1.7179 | 1.0504 |
| C_1 | Gh_A02G0963 | WNK8 | Serine/threonine-protein kinase WNK8 | -0.5742 | -0.8264 | 0.1079 | -0.0880 |
| C_1 | Gh_A02G1112 | CDI | Protein CDI | -0.0187 | 0.8669 | 0.8457 | 1.0247 |
| C_1 | Gh_A02G1216 | NA | NA | -2.4835 | -0.9765 | -0.2061 | -1.1975 |
| C_1 | Gh_A02G1315 | NA | NA | -0.8482 | -1.5966 | 2.7688 | 1.8816 |
| C_1 | Gh_A02G1337 | NA | NA | -0.8263 | -0.2860 | 0.2281 | 1.6417 |
| C_1 | Gh_A02G1420 | NA | NA | -1.1154 | -1.3204 | 1.1231 | -0.0386 |
| C_1 | Gh_A02G1428 | HAT22 | Homeobox-leucine zipper protein HAT22 | -0.7267 | -0.3100 | 0.6741 | 0.4807 |
| C_1 | Gh_A02G1455 | APK1 | Adenylyl-sulfate kinase 1, chloroplastic | -0.3826 | 0.4140 | 0.1399 | 0.3118 |
| C_1 | Gh_A02G1465 | NA | NA | -0.1074 | -0.6835 | 0.9942 | 0.4990 |
| C_1 | Gh_A02G1500 | mkkA | Mitogen-activated protein kinase kinase kinase A | -0.9342 | -0.7388 | 1.6675 | 0.6365 |
| C_1 | Gh_A02G1515 | NA | NA | -0.4601 | 0.0199 | 0.3221 | -0.0427 |
| C_1 | Gh_A02G1539 | SUVR5 | Histone-lysine N-methyltransferase SUVR5 | -1.5461 | 0.2702 | -0.1915 | 0.7728 |
| C_1 | Gh_A02G1547 | At2g23790 | Calcium uniporter protein 2, mitochondrial | -0.6470 | -0.7695 | 0.0367 | -0.3518 |
| C_1 | Gh_A02G1548 | At2g23790 | Calcium uniporter protein 2, mitochondrial | -0.6608 | -0.9301 | 0.0757 | -0.1257 |
| C_1 | Gh_A02G1734 | At1g06620 | 1-aminocyclopropane-1-carboxylate oxidase homolog 1 | -3.9331 | -2.5798 | -0.6498 | -1.0043 |
| C_1 | Gh_A02G1760 | NA | Fructose-1,6-bisphosphatase, cytosolic | -4.8285 | 0.5720 | -0.2311 | 0.5926 |
| C_1 | Gh_A02G1788 | S-ACP-DES6 | Stearoyl-[acyl-carrier-protein] 9-desaturase 6, | 0.1534 | 1.1765 | 1.1611 | 1.7484 |
|  |  |  | chloroplastic |  |  |  |  |
| C_1 | Gh_A03G0142 | MPA1 | Puromycin-sensitive aminopeptidase | -0.3044 | 0.1677 | 0.2814 | 0.0973 |
| C_1 | Gh_A03G0169 | POPTRDRAF | Biotin carboxylase 1, chloroplastic | -0.5366 | -0.2773 | -0.2173 | 0.3475 |
|  |  | T_831870 |  |  |  |  |  |
| C_1 | Gh_A03G0209 | FBL3 | F-box/LRR-repeat protein 3 | -1.9391 | -1.3230 | -0.2007 | -0.4524 |
| C_1 | Gh_A03G0256 | NRT2.3 | High affinity nitrate transporter 2.3 | 0.4385 | -3.4511 | 4.8234 | 5.6344 |
| C_1 | Gh_A03G0257 | NRT2.1 | High-affinity nitrate transporter 2.1 | -2.3713 | -4.5108 | -0.1941 | 0.5400 |
| C_1 | Gh_A03G0266 | CNGC15 | Putative cyclic nucleotide-gated ion channel 15 | 0.3574 | 0.6188 | 0.8589 | 0.5685 |
| C_1 | Gh_A03G0330 | ZAT12 | Zinc finger protein ZAT12 | -0.3567 | -1.6618 | 1.8348 | 1.6156 |
| C_1 | Gh_A03G0428 | NA | NA | 0.1119 | 0.0224 | 1.2015 | 1.0030 |
| C_1 | Gh_A03G0441 | NA | NA | -0.5337 | -0.4206 | -0.2119 | 0.4876 |
| C_1 | Gh_A03G0594 | MYB34 | Transcription factor MYB34 | -0.9522 | -2.0407 | 4.0547 | 1.8025 |
| C_1 | Gh_A03G0595 | dnajc10 | DnaJ homolog subfamily C member 10 | -0.4546 | 0.3185 | 0.4883 | 0.5441 |
| C_1 | Gh_A03G0791 | NUDT4 | Nudix hydrolase 4 | -0.2947 | 0.3720 | 1.0671 | 1.3597 |
| C_1 | Gh_A03G0895 | NA | NA | 0.3261 | 0.9130 | 0.9914 | 1.0599 |
| C_1 | Gh_A03G1007 | NA | NA | -1.3661 | -2.2371 | 0.3301 | -0.4259 |
| C_1 | Gh_A03G1125 | NA | NA | -0.4568 | -0.3533 | 0.9812 | 0.7948 |
| C_1 | Gh_A03G1170 | NA | NA | -0.0002 | -0.1248 | 0.7095 | 0.4526 |
| C_1 | Gh_A03G1185 | DTX29 | Protein DETOXIFICATION 29 | -1.4455 | -1.4092 | -0.8252 | -0.4131 |
| C_1 | Gh_A03G1186 | DTX29 | Protein DETOXIFICATION 29 | 0.0689 | -0.0492 | 0.1392 | 0.3185 |
| C_1 | Gh_A03G1209 | MENB | 1,4-dihydroxy-2-naphthoyl-CoA synthase, peroxisomal | -1.0344 | -1.8274 | 0.6250 | 1.1040 |
| C_1 | Gh_A03G1262 | FH8 | Formin-like protein 8 | -0.3326 | 0.4072 | 1.0570 | 0.4606 |
| C_1 | Gh_A03G1281 | NA | NA | -0.1419 | -0.3577 | 0.3582 | 0.2627 |
| C_1 | Gh_A03G1315 | NA | NA | -1.7471 | -2.7030 | 3.4230 | 2.6244 |
| C_1 | Gh_A03G1341 | TIFY9 | Protein TIFY 9 | -0.4014 | -0.1984 | 1.8479 | 0.7400 |
| C_1 | Gh_A03G1352 | CYP51G1 | Sterol 14-demethylase | 0.4716 | 0.8135 | 0.9849 | 0.7408 |
| C_1 | Gh_A03G1373 | RTNLB18 | Reticulon-like protein B18 | -0.2392 | -0.0005 | 0.5857 | 0.5138 |
| C_1 | Gh_A03G1384 | NA | NA | 0.8292 | 1.7423 | 2.3215 | 0.7093 |
| C_1 | Gh_A03G1453 | RPL44 | 60S ribosomal protein L44 | -2.5587 | -1.2063 | 1.5516 | 1.1613 |
| C_1 | Gh_A03G1611 | ZAT10 | Zinc finger protein ZAT10 | -0.3557 | 0.0005 | 1.4343 | 1.6871 |
| C_1 | Gh_A03G1634 | NA | Limonoid UDP-glucosyltransferase | -0.8617 | 0.4916 | 0.5876 | 0.7175 |
| C_1 | Gh_A03G1801 | plcA | 1-phosphatidylinositol phosphodiesterase | 0.0374 | -0.0810 | 0.2372 | 0.4566 |
| C_1 | Gh_A03G1897 | NA | NA | -0.3075 | 0.3989 | 3.1061 | 2.3547 |
| C_1 | Gh_A03G1917 | RABA2A | Ras-related protein RABA2a | 0.0674 | 0.0317 | 0.3919 | 0.5849 |
| C_1 | Gh_A03G1923 | CAT1 | Cationic amino acid transporter 1 | -0.1418 | 0.7738 | 0.4319 | 0.9649 |
| C_1 | Gh_A03G1960 | At1g32060 | Phosphoribulokinase, chloroplastic | -1.5975 | -1.1791 | 1.4827 | 1.7276 |
| C_1 | Gh_A03G1962 | 4CLL6 | 4-coumarate--CoA ligase-like 6 | -1.1460 | -2.2710 | 0.9831 | -0.1814 |
| C_1 | Gh_A03G2028 | IQD14 | Protein IQ-DOMAIN 14 | -0.2675 | 0.3578 | 0.2607 | 0.6017 |
| C_1 | Gh_A03G2049 | GT6 | UDP-glucose flavonoid 3-O-glucosyltransferase 6 | -0.4028 | 0.0770 | 2.2484 | 4.1183 |
| C_1 | Gh_A03G2115 | PUB21 | U-box domain-containing protein 21 | -0.2567 | -0.1967 | 1.6521 | 0.8969 |
| C_1 | Gh_A03G2174 | KCS4 | 3-ketoacyl-CoA synthase 4 | -0.7103 | 0.0888 | 0.0397 | 0.0838 |
| C_1 | Gh_A04G0022 | ATJ11 | Chaperone protein dnaJ 11, chloroplastic | 0.7609 | 0.7193 | 1.5605 | 0.8536 |
| C_1 | Gh_A04G0157 | At3g47570 | Probable LRR receptor-like serine/threonine-protein | -1.4336 | -1.6846 | -0.5933 | -0.1787 |
|  |  |  | kinase |  |  |  |  |
| C_1 | Gh_A04G0184 | NA | NA | -0.1209 | 0.6446 | 3.2140 | 3.5612 |
| C_1 | Gh_A04G0232 | menG | Demethylmenaquinone methyltransferase | 0.3418 | 0.7483 | 1.2310 | 0.7959 |
| C_1 | Gh_A04G0264 | NA | NA | 1.1763 | 0.3809 | 2.6193 | 2.0319 |
| C_1 | Gh_A04G0649 | IQD1 | Protein IQ-DOMAIN 1 | -0.1462 | -0.1369 | 0.0946 | 0.0551 |
| C_1 | Gh_A04G0700 | NA | NA | -1.0623 | -0.7085 | -0.2414 | 0.0252 |
| C_1 | Gh_A04G0970 | AOMI | Ubiquinol oxidase, mitochondrial | -0.0370 | -0.6723 | 1.4332 | 0.3882 |
| C_1 | Gh_A04G0993 | YLR126C | Putative glutamine amidotransferase | -0.1502 | 0.4242 | 1.1297 | 0.3766 |
| C_1 | Gh_A04G1075 | At2g17140 | Pentatricopeptide repeat-containing protein | -0.5342 | 0.4210 | 1.0303 | 0.2936 |
| C_1 | Gh_A04G1106 | XLG1 | Extra-large guanine nucleotide-binding protein 1 | 0.1071 | 0.1244 | 1.1231 | 0.7624 |
| C_1 | Gh_A04G1360 | SCPL29 | Serine carboxypeptidase-like 29 | -0.0329 | 0.1604 | 0.4845 | -0.0690 |
| C_1 | Gh_A04G1411 | tmem53 | Transmembrane protein 53 | 0.4099 | 0.7190 | 1.1969 | 1.0011 |
| C_1 | Gh_A04G1469 | hpxO | FAD-dependent urate hydroxylase | -3.4400 | -1.1576 | -1.8906 | 1.7328 |
| C_1 | Gh_A04G1470 | xlnD | 3-hydroxybenzoate 6-hydroxylase 1 | -1.6086 | -1.5308 | 0.5658 | 0.1049 |
| C_1 | Gh_A04G1471 | nicC | 6-hydroxynicotinate 3-monooxygenase | -1.4753 | -1.9384 | 0.2381 | -0.1231 |
| C_1 | Gh_A05G0057 | NA | NA | 0.4871 | 0.7657 | 0.7143 | 1.1221 |
| C_1 | Gh_A05G0111 | NA | Early nodulin-93 | -0.0407 | 1.0016 | 2.2664 | 1.6147 |
| C_1 | Gh_A05G0112 | NA | Early nodulin-93 | -0.5751 | 1.5219 | 0.9526 | 0.9599 |
| C_1 | Gh_A05G0200 | At4g29190 | Zinc finger CCCH domain-containing protein 49 | -0.8680 | -0.4274 | -0.4053 | 0.2496 |
| C_1 | Gh_A05G0225 | IQM1 | IQ domain-containing protein IQM1 | 0.1297 | -0.4784 | 1.4287 | 1.0221 |
| C_1 | Gh_A05G0531 | NA | NA | -0.5048 | 0.1178 | 1.0709 | 0.8120 |
| C_1 | Gh_A05G0622 | NA | NA | -0.6600 | 0.4071 | 1.5287 | 0.8255 |
| C_1 | Gh_A05G0623 | MLO6 | MLO-like protein 6 | -0.6422 | -1.3740 | 0.5392 | -0.0062 |
| C_1 | Gh_A05G0718 | STOP1 | Protein SENSITIVE TO PROTON RHIZOTOXICITY 1 | -2.7197 | -1.7528 | -0.5076 | -0.5651 |
| C_1 | Gh_A05G0727 | GA2OX8 | Gibberellin 2-beta-dioxygenase 8 | -1.0527 | -0.6249 | -0.0842 | 0.3924 |
| C_1 | Gh_A05G0803 | TRP5 | Telomere repeat-binding protein 5 | -0.2063 | -0.1145 | -0.0668 | 0.1249 |
| C_1 | Gh_A05G0855 | PHOT1 | Phototropin-1 | 0.5293 | 1.1104 | 3.6525 | 3.1047 |
| C_1 | Gh_A05G1145 | NA | NA | 0.3642 | 0.6230 | 0.7021 | 0.7071 |
| C_1 | Gh_A05G1171 | GLIP1 | GDSL esterase/lipase 1 | -0.0370 | -0.4311 | 0.5283 | 0.9811 |
| C_1 | Gh_A05G1191 | RNF217 | Probable E3 ubiquitin-protein ligase | -1.3552 | -1.9500 | 1.2406 | 1.0115 |
| C_1 | Gh_A05G1331 | ERF091 | Ethylene-responsive transcription factor | 0.7145 | -0.3273 | 1.0983 | 2.1227 |
| C_1 | Gh_A05G1417 | NA | NA | 0.7198 | 1.9954 | 2.2251 | 2.3985 |
| C_1 | Gh_A05G1448 | NA | NA | 0.4844 | 0.4651 | 0.7915 | 0.8292 |
| C_1 | Gh_A05G1468 | At4g22030 | Probable F-box protein | -0.9608 | -4.0449 | 4.9501 | 4.0365 |
| C_1 | Gh_A05G1617 | SLAH1 | S-type anion channel SLAH1 | -0.8037 | -0.9644 | -0.5113 | -0.1211 |
| C_1 | Gh_A05G1690 | GATL1 | Probable galacturonosyltransferase-like 1 | 0.2574 | 0.1238 | 1.3888 | 1.4285 |
| C_1 | Gh_A05G1692 | LYK4 | LysM domain receptor-like kinase 4 | -1.7185 | -1.8354 | -0.5559 | 0.3021 |
| C_1 | Gh_A05G1771 | SCE1 | SUMO-conjugating enzyme SCE1 | -1.4461 | -0.7673 | 1.6451 | 1.5974 |
| C_1 | Gh_A05G1772 | FRA1 | Kinesin-like protein FRA1 | 0.0517 | 0.3243 | 0.4548 | 0.8083 |
| C_1 | Gh_A05G1815 | ZAT10 | Zinc finger protein ZAT10 | -0.1153 | 0.7651 | 0.4263 | 0.6805 |
| C_1 | Gh_A05G1837 | NA | NA | -0.2837 | 1.0159 | 2.3788 | 2.0075 |
| C_1 | Gh_A05G1887 | PGI1 | Glucose-6-phosphate isomerase 1, chloroplastic | 0.2416 | 0.2066 | 0.6369 | 0.5944 |
| C_1 | Gh_A05G1915 | NA | Histone H3.3 | 0.0599 | 0.1904 | 0.3540 | 0.3398 |
| C_1 | Gh_A05G1921 | PCO1 | Plant cysteine oxidase 1 | 0.6157 | 0.8573 | 1.2728 | 1.6141 |
| C_1 | Gh_A05G1978 | NA | NA | -0.3632 | -1.8755 | 1.9860 | 1.5288 |
| C_1 | Gh_A05G2069 | ERF114 | Ethylene-responsive transcription factor | -1.2209 | 0.0771 | 1.1697 | 0.4310 |
| C_1 | Gh_A05G2125 | PDIL5-2 | Protein disulfide-isomerase 5-2 | -0.4499 | -0.6288 | 0.3804 | -0.1430 |
| C_1 | Gh_A05G2146 | ERF020 | Ethylene-responsive transcription factor | 0.9862 | 0.1690 | 1.2478 | 2.9089 |
| C_1 | Gh_A05G2171 | At1g76070 | Uncharacterized protein | -2.3820 | -1.5920 | -0.6877 | 0.5314 |
| C_1 | Gh_A05G2246 | CYP81E8 | Cytochrome P450 81E8 | -0.3996 | -0.3663 | 0.4435 | 0.3993 |
| C_1 | Gh_A05G2281 | QUA2 | Probable pectin methyltransferase QUA2 | -0.2030 | 0.8577 | 0.6232 | 1.4215 |
| C_1 | Gh_A05G2403 | NA | NA | 0.2618 | 2.0411 | 3.0497 | 2.2529 |
| C_1 | Gh_A05G2519 | NA | NA | 0.5072 | 0.8258 | 1.5227 | 1.1921 |
| C_1 | Gh_A05G2695 | ERMP1 | Endoplasmic reticulum metallopeptidase 1 | -1.0215 | -0.1653 | 0.4715 | 0.3763 |
| C_1 | Gh_A05G2721 | CRK2 | Cysteine-rich receptor-like protein kinase 2 | -0.4910 | -0.1459 | 0.6326 | 0.0321 |
| C_1 | Gh_A05G2753 | At3g47570 | Probable LRR receptor-like serine/threonine-protein | -0.2197 | -0.5338 | 1.3507 | 0.3336 |
|  |  |  | kinase |  |  |  |  |
| C_1 | Gh_A05G2878 | ANS | Leucoanthocyanidin dioxygenase | -1.2998 | 0.6396 | 0.8424 | 1.3183 |
| C_1 | Gh_A05G3183 | VTI13 | Vesicle transport v-SNARE 13 | -0.4017 | -0.3233 | -0.0496 | -0.5489 |
| C_1 | Gh_A05G3255 | HSFA4B | Heat stress transcription factor A-4b | -0.6402 | -0.1622 | 1.3869 | 0.7778 |
| C_1 | Gh_A05G3396 | At1g65240 | Aspartic proteinase-like protein 2 | 0.0848 | 0.0611 | 0.7266 | 0.3380 |
| C_1 | Gh_A05G3422 | ALA1 | Phospholipid-transporting ATPase 1 | -0.0339 | -0.5221 | 1.0719 | 0.2419 |
| C_1 | Gh_A05G3462 | SIB1 | Sigma factor binding protein 1, chloroplastic | -0.3077 | -1.0708 | 0.5888 | 0.3967 |
| C_1 | Gh_A05G3485 | NA | NA | -0.1870 | -1.1494 | 1.3569 | 1.4071 |
| C_1 | Gh_A05G3547 | PIRL6 | Plant intracellular Ras-group-related LRR protein 6 | 0.3209 | -0.9487 | 3.5828 | 3.7870 |
| C_1 | Gh_A05G3668 | At5g57670 | Probable receptor-like serine/threonine-protein kinase | 0.0092 | -0.2288 | 0.2005 | 0.3184 |
| C_1 | Gh_A05G3751 | ERF012 | Ethylene-responsive transcription factor | -0.4932 | 0.6771 | 1.1242 | 2.0035 |
| C_1 | Gh_A05G3909 | MLO3 | MLO-like protein 3 | -2.4185 | -0.6066 | -0.1104 | -0.1142 |
| C_1 | Gh_A05G3932 | FMO1 | Probable flavin-containing monooxygenase 1 | -1.4746 | -1.9486 | 2.4627 | 1.3025 |
| C_1 | Gh_A06G0001 | Trmt61a | tRNA (adenine(58)-N(1)-methyltransferase catalytic | 0.1283 | 0.0605 | 1.9872 | 0.5627 |
|  |  |  | subunit |  |  |  |  |
| C_1 | Gh_A06G0033 | NA | Thaumatin-like protein 1 | -0.8791 | 0.5303 | 0.4098 | 0.8839 |
| C_1 | Gh_A06G0107 | RGA3 | Putative disease resistance protein RGA3 | -0.2640 | 0.2154 | 0.2476 | 0.2425 |
| C_1 | Gh_A06G0136 | MYB44 | Transcription factor MYB44 | -0.9375 | -1.0078 | -0.2127 | 0.0609 |
| C_1 | Gh_A06G0157 | CPK34 | Calcium-dependent protein kinase 34 | -3.2712 | 0.1502 | 0.0155 | 0.1192 |
| C_1 | Gh_A06G0201 | NA | NA | -0.6910 | -0.9401 | 1.4068 | 0.5822 |
| C_1 | Gh_A06G0260 | At4g40080 | Putative clathrin assembly protein | -0.6068 | -0.6197 | 1.4725 | 1.0503 |
| C_1 | Gh_A06G0345 | ORP1C | Oxysterol-binding protein-related protein 1C | 0.2215 | 0.3123 | 0.4878 | 0.2251 |
| C_1 | Gh_A06G0420 | NA | NA | -1.0550 | 0.0230 | 1.1526 | 0.9278 |
| C_1 | Gh_A06G0547 | ROPGEF9 | Rop guanine nucleotide exchange factor 9 | -1.2467 | 2.1239 | 3.0607 | 3.1791 |
| C_1 | Gh_A06G0558 | XTH26 | Probable xyloglucan endotransglucosylase/hydrolase protein 26 | 0.1040 | 0.4963 | 1.2841 | 1.0495 |
| C_1 | Gh_A06G0604 | PFK3 | ATP-dependent 6-phosphofructokinase 3 | 0.2282 | 0.3016 | 0.6450 | 0.3551 |
| C_1 | Gh_A06G0772 | NA | Eukaryotic initiation factor 4A-8 | -0.1329 | 0.5911 | 0.3651 | 0.6832 |
| C_1 | Gh_A06G0995 | DDB_G02890 | IST1-like protein | -3.1249 | -0.2090 | 0.3283 | -0.6103 |
|  |  | 29 |  |  |  |  |  |
| C_1 | Gh_A06G1074 | CRK15 | Cysteine-rich receptor-like protein kinase 15 | -0.5269 | -1.3901 | 0.1499 | 0.5919 |
| C_1 | Gh_A06G1341 | ACO | 1-aminocyclopropane-1-carboxylate oxidase | 0.2577 | 0.5162 | 0.6679 | 1.9722 |
| C_1 | Gh_A06G1381 | NUDT2 | Nudix hydrolase 2 | -0.9895 | -1.8510 | 0.7550 | 0.8459 |
| C_1 | Gh_A06G1433 | At2g40140 | Zinc finger CCCH domain-containing protein 29 | -0.4997 | -0.0490 | 0.2058 | 1.2818 |
| C_1 | Gh_A06G1585 | WRKY70 | Probable WRKY transcription factor 70 | 0.4865 | -0.7501 | 2.5281 | 2.0272 |
| C_1 | Gh_A06G1728 | OPT5 | Oligopeptide transporter 5 | -1.3999 | -1.1860 | 0.1371 | -0.9010 |
| C_1 | Gh_A06G1784 | CRK35 | Putative cysteine-rich receptor-like protein kinase 35 | -0.3158 | 0.1576 | 1.2370 | 0.1937 |
| C_1 | Gh_A06G1879 | NA | NA | -0.5579 | -0.4017 | 0.5527 | -0.0253 |
| C_1 | Gh_A06G1884 | AHL17 | AT-hook motif nuclear-localized protein 17 | -0.8446 | 0.1898 | 1.1631 | 2.4065 |
| C_1 | Gh_A06G1945 | NA | NA | -0.5973 | 0.1595 | 0.2430 | -0.3369 |
| C_1 | Gh_A06G1961 | NA | Cysteine proteinase inhibitor 1 | 0.0627 | 1.9916 | 2.3867 | 2.9119 |
| C_1 | Gh_A06G2067 | RKF1 | Probable LRR receptor-like serine/threonine-protein | -0.4501 | -0.8543 | 0.7799 | 0.4102 |
|  |  |  | kinase RFK1 |  |  |  |  |
| C_1 | Gh_A07G0020 | At5g20050 | Probable receptor-like protein kinase | -1.2056 | -1.6504 | 0.9366 | 0.7638 |
| C_1 | Gh_A07G0066 | LRX4 | Leucine-rich repeat extensin-like protein 4 | -0.3267 | 0.7712 | 0.5322 | 0.6900 |
| C_1 | Gh_A07G0209 | GAPN | NADP-dependent glyceraldehyde-3-phosphate | -2.6223 | -0.4691 | -0.9477 | -0.7081 |
|  |  |  | dehydrogenase |  |  |  |  |
| C_1 | Gh_A07G0252 | NA | NA | 0.0313 | 0.7457 | 1.4436 | 0.8575 |
| C_1 | Gh_A07G0261 | WRKY18 | WRKY transcription factor 18 | 1.6572 | 2.8436 | 4.6785 | 2.5688 |
| C_1 | Gh_A07G0409 | CHLM | Magnesium protoporphyrin IX methyltransferase, | -1.6749 | 0.5881 | 0.5675 | 0.8083 |
|  |  |  | chloroplastic |  |  |  |  |
| C_1 | Gh_A07G0427 | SAUR71 | Auxin-responsive protein SAUR71 | -0.8792 | -1.1677 | -0.7794 | -0.2078 |
| C_1 | Gh_A07G0609 | UPTG2 | Alpha-1,4-glucan-protein synthase [UDP-forming] 2 | 0.2110 | 0.1153 | 0.2041 | 0.3938 |
| C_1 | Gh_A07G0614 | NA | NA | -1.0647 | -0.5627 | -0.2229 | 4.7887 |
| C_1 | Gh_A07G0673 | DTX8 | Protein DETOXIFICATION 8 | -1.7778 | -0.3948 | 0.3056 | 0.0610 |
| C_1 | Gh_A07G0677 | NA | NA | -2.9667 | -1.1776 | -0.9218 | -1.3990 |
| C_1 | Gh_A07G0758 | NA | NA | 0.1968 | 0.4417 | 0.7845 | 0.4959 |
| C_1 | Gh_A07G0860 | ECA4 | Calcium-transporting ATPase 4, endoplasmic reticulum- | -0.1951 | -0.4125 | -0.0702 | 0.1003 |
|  |  |  | type |  |  |  |  |
| C_1 | Gh_A07G0909 | At3g19950 | E3 ubiquitin-protein ligase RING1-like | -0.0081 | 0.3523 | 1.6745 | 2.1830 |
| C_1 | Gh_A07G0932 | CRCK1 | Calmodulin-binding receptor-like cytoplasmic kinase 1 | 0.1185 | 0.4707 | 0.8232 | 0.9985 |
| C_1 | Gh_A07G1009 | NAC090 | NAC domain-containing protein 90 | -0.9216 | -0.3603 | 2.3264 | 1.8608 |
| C_1 | Gh_A07G1167 | NA | NA | 0.3961 | 0.0788 | 1.9320 | 1.1965 |
| C_1 | Gh_A07G1171 | BZIP53 | bZIP transcription factor 53 | -0.1337 | 0.5105 | 0.6727 | 0.3426 |
| C_1 | Gh_A07G1217 | At1g02270 | Uncharacterized calcium-binding protein | 0.2932 | 1.3685 | 1.3845 | 1.4573 |
| C_1 | Gh_A07G1274 | WRKY46 | Probable WRKY transcription factor 46 | -0.4514 | -0.3355 | 1.5617 | 0.9986 |
| C_1 | Gh_A07G1357 | GAE3 | UDP-glucuronate 4-epimerase 3 | 0.1487 | 0.9588 | 1.1679 | 0.9337 |
| C_1 | Gh_A07G1396 | SETH3 | Probable arabinose 5-phosphate isomerase | 0.2579 | 0.3114 | 0.5259 | 0.3140 |
| C_1 | Gh_A07G1614 | ywbO | Uncharacterized protein YwbO | -0.2782 | -0.3327 | -0.1075 | -0.1586 |
| C_1 | Gh_A07G1629 | FER | Receptor-like protein kinase FERONIA | -1.3459 | -3.2551 | 2.9430 | 2.1458 |
| C_1 | Gh_A07G1657 | UGT82A1 | UDP-glycosyltransferase 82A1 | -1.8472 | 0.9586 | 1.3669 | 0.8494 |
| C_1 | Gh_A07G1738 | NFYB3 | Nuclear transcription factor Y subunit B-3 | -1.1693 | 0.4627 | 1.2636 | -0.3254 |
| C_1 | Gh_A07G1779 | GLR1.3 | Glutamate receptor 1.3 | -0.2221 | -0.8115 | 1.6501 | 0.6618 |
| C_1 | Gh_A07G1802 | A6 | Probable glucan endo-1,3-beta-glucosidase A6 | 0.1345 | 0.5268 | 0.5406 | 0.5501 |
| C_1 | Gh_A07G1871 | CESA4 | Cellulose synthase A catalytic subunit 4 [UDP-forming] | 0.0304 | -0.0723 | 0.3161 | 1.1527 |
| C_1 | Gh_A07G2010 | SIP1-1 | Aquaporin SIP1-1 | -0.6086 | 0.1708 | 1.4006 | 1.7300 |
| C_1 | Gh_A07G2059 | HT1 | Serine/threonine-protein kinase HT1 | 0.4203 | 0.2734 | 1.6376 | 1.1862 |
| C_1 | Gh_A07G2219 | OXI1 | Serine/threonine-protein kinase OXI1 | -3.8823 | -0.4895 | 0.8235 | 1.8339 |
| C_1 | Gh_A07G2274 | WRKY42 | Probable WRKY transcription factor 42 | -0.0474 | -0.3125 | 1.4415 | 0.4578 |
| C_1 | Gh_A07G2285 | BT1 | BTB/POZ and TAZ domain-containing protein 1 | 0.0758 | -0.3876 | 1.0174 | 0.3992 |
| C_1 | Gh_A07G2307 | IQM1 | IQ domain-containing protein IQM1 | 0.2595 | -0.3536 | 1.2824 | 0.9944 |
| C_1 | Gh_A08G0003 | CTIMC | Triosephosphate isomerase, cytosolic | -0.0643 | 0.9094 | 1.1172 | 0.5146 |
| C_1 | Gh_A08G0043 | CYP94C1 | Cytochrome P450 94C1 | -1.9612 | -0.8934 | 2.6219 | 1.1612 |
| C_1 | Gh_A08G0068 | CAF1-11 | Probable CCR4-associated factor 1 homolog 11 | -1.6379 | 0.0811 | 0.4695 | 1.4147 |
| C_1 | Gh_A08G0091 | NA | NA | -0.2713 | -1.2397 | 2.0193 | 1.1563 |
| C_1 | Gh_A08G0225 | NA | NA | -0.1980 | 0.6602 | 0.4325 | 1.3491 |
| C_1 | Gh_A08G0277 | NA | NA | -0.1047 | 0.1677 | 0.0933 | 0.1346 |
| C_1 | Gh_A08G0316 | AOC4 | Allene oxide cyclase 4, chloroplastic | 0.3165 | 1.0297 | 1.8421 | 0.4945 |
| C_1 | Gh_A08G0337 | UGPA | UTP--glucose-1-phosphate uridylyltransferase | 0.3163 | 0.2816 | 0.8052 | 0.2318 |
| C_1 | Gh_A08G0351 | NA | NA | 1.1213 | 2.2740 | 7.7208 | 6.9553 |
| C_1 | Gh_A08G0374 | GSO2 | LRR receptor-like serine/threonine-protein kinase GSO2 | 1.2219 | 1.3530 | 4.9303 | 0.7093 |
| C_1 | Gh_A08G0808 | PAT24 | Protein S-acyltransferase 24 | -0.0970 | -0.3281 | 0.8122 | 0.0913 |
| C_1 | Gh_A08G0872 | NA | Hevamine-A | -0.2382 | -0.3521 | 1.9276 | 2.3292 |
| C_1 | Gh_A08G1045 | At3g01520 | Universal stress protein A-like protein | 0.2229 | 1.0630 | 3.4906 | 4.1488 |
| C_1 | Gh_A08G1046 | COX5C | Cytochrome c oxidase subunit 5C | 0.0392 | -0.0557 | 0.4350 | 0.1674 |
| C_1 | Gh_A08G1059 | MYB108 | Transcription factor MYB108 | 1.0134 | 0.3512 | 3.2738 | 3.5309 |
| C_1 | Gh_A08G1183 | MPK4 | Mitogen-activated protein kinase 4 | 0.0933 | -0.0885 | 0.8305 | 0.4648 |
| C_1 | Gh_A08G1190 | NA | NA | -2.0254 | -0.7737 | 2.0846 | 1.4087 |
| C_1 | Gh_A08G1277 | SCPL40 | Serine carboxypeptidase-like 40 | -0.7267 | 0.6465 | 0.9877 | 1.0817 |
| C_1 | Gh_A08G1293 | NA | NA | -0.5603 | -0.6490 | 2.4554 | 0.4974 |
| C_1 | Gh_A08G1334 | NFD4 | Protein NUCLEAR FUSION DEFECTIVE 4 | -0.1755 | -0.0339 | 0.5809 | 0.3405 |
| C_1 | Gh_A08G1401 | MYB39 | Transcription factor MYB39 | -1.0958 | -2.2535 | 4.5281 | 4.4019 |
| C_1 | Gh_A08G1458 | NA | NA | -0.2913 | 0.2617 | 1.6222 | 0.0908 |
| C_1 | Gh_A08G1562 | At1g62810 | Primary amine oxidase | 0.2810 | 0.3107 | 0.8770 | 0.2289 |
| C_1 | Gh_A08G1585 | CYP76B6 | Geraniol 8-hydroxylase | -1.2227 | 0.0988 | 0.0151 | -0.1758 |
| C_1 | Gh_A08G1730 | RLP12 | Receptor-like protein 12 | -1.5377 | -1.4944 | 0.6322 | 1.1987 |
| C_1 | Gh_A08G1761 | PIP2-2 | Probable aquaporin PIP2-2 | -3.5183 | -3.9765 | -1.7637 | -0.7878 |
| C_1 | Gh_A08G2049 | NA | Myb-related protein Zm1 | -1.0072 | 0.3325 | 0.6249 | -0.2318 |
| C_1 | Gh_A08G2078 | DGK7 | Diacylglycerol kinase 7 | 0.3364 | 0.5850 | 0.9021 | 0.2017 |
| C_1 | Gh_A08G2164 | NA | NA | -0.0230 | 0.1971 | 0.3372 | 0.5576 |
| C_1 | Gh_A08G2191 | harbi1 | Putative nuclease HARBI1 | 0.1025 | 0.7332 | 1.9129 | 1.8304 |
| C_1 | Gh_A08G2292 | NA | NA | -0.7388 | -0.3473 | -0.1373 | 0.0389 |
| C_1 | Gh_A08G2295 | DIR4 | Dirigent protein 4 | -5.6027 | -4.3333 | -0.5591 | -1.3895 |
| C_1 | Gh_A08G2338 | PCO2 | Plant cysteine oxidase 2 | 0.0665 | 1.7042 | 1.7953 | 2.6868 |
| C_1 | Gh_A08G2389 | AAE3 | Oxalate--CoA ligase | 0.0093 | 0.1651 | 0.8444 | 0.6348 |
| C_1 | Gh_A08G2416 | NA | NA | -0.4400 | -1.8331 | 0.7474 | 1.8046 |
| C_1 | Gh_A09G0016 | ABCG10 | ABC transporter G family member 10 | -0.5314 | -0.6453 | -0.2229 | -0.2525 |
| C_1 | Gh_A09G0137 | CML45 | Probable calcium-binding protein CML45 | -1.3287 | -2.3579 | 0.1425 | 0.0918 |
| C_1 | Gh_A09G0187 | At4g08850 | Probable LRR receptor-like serine/threonine-protein | 0.3070 | 0.1188 | 2.9261 | 2.6386 |
|  |  |  | kinase |  |  |  |  |
| C_1 | Gh_A09G0199 | At4g08850 | Probable LRR receptor-like serine/threonine-protein | -0.1144 | 0.4400 | 2.6904 | 1.0264 |
|  |  |  | kinase |  |  |  |  |
| C_1 | Gh_A09G0296 | GLCAK1 | Glucuronokinase 1 | -1.2284 | 0.2135 | -0.3308 | 0.0117 |
| C_1 | Gh_A09G0341 | At3g27220 | Kelch repeat-containing protein | 0.4439 | 1.6090 | 1.7320 | 2.2260 |
| C_1 | Gh_A09G0485 | At1g18390 | Probable serine/threonine-protein kinase | -0.5216 | -0.5832 | 0.3494 | -0.0074 |
| C_1 | Gh_A09G0645 | MYB108 | Transcription factor MYB108 | -2.3261 | -2.3268 | -1.6767 | -0.2056 |
| C_1 | Gh_A09G0653 | CIPK1 | CBL-interacting serine/threonine-protein kinase 1 | -0.4537 | 0.5403 | 0.7602 | 0.0930 |
| C_1 | Gh_A09G0769 | ALA4 | Probable phospholipid-transporting ATPase 4 | -4.2961 | -4.8115 | -1.5599 | -0.6808 |
| C_1 | Gh_A09G0785 | NA | Dynein light chain LC6, flagellar outer arm | -1.8841 | 0.0155 | -0.3153 | -0.6497 |
| C_1 | Gh_A09G0798 | NA | NA | -3.8180 | -3.4265 | 1.6661 | -0.2610 |
| C_1 | Gh_A09G0835 | STOP1 | Protein SENSITIVE TO PROTON RHIZOTOXICITY 1 | -2.9186 | -3.4413 | -0.6071 | 0.5065 |
| C_1 | Gh_A09G0923 | CAF1-9 | Probable CCR4-associated factor 1 homolog 9 | 0.2766 | 0.3852 | 1.7436 | 1.5221 |
| C_1 | Gh_A09G0976 | At3g03100 | Probable NADH dehydrogenase 1 alpha subcomplex | -0.6336 | -0.8824 | -0.1169 | -0.3063 |
|  |  |  | subunit 12 |  |  |  |  |
| C_1 | Gh_A09G1038 | At5g46170 | F-box protein | -0.3450 | 0.7056 | 0.8102 | 0.3739 |
| C_1 | Gh_A09G1118 | CML35 | Probable calcium-binding protein CML35 | 0.0684 | -0.1654 | 0.3140 | 0.9103 |
| C_1 | Gh_A09G1337 | PP2A15 | F-box protein PP2-A15 | -0.1665 | 0.0968 | 0.9135 | 0.5622 |
| C_1 | Gh_A09G1446 | NA | NA | -0.5297 | 1.3749 | 1.4852 | 1.0638 |
| C_1 | Gh_A09G1617 | CYB561A | Transmembrane ascorbate ferrireductase 1 | -0.5285 | -0.1564 | 0.6222 | 0.2775 |
| C_1 | Gh_A09G1800 | At2g33170 | Probable leucine-rich repeat receptor-like protein kinase | -0.0054 | -0.3112 | 0.8747 | 0.0252 |
| C_1 | Gh_A09G1847 | Os03g0144800 | Xyloglucan galactosyltransferase KATAMARI1 homolog | -2.1764 | -2.5044 | -0.4838 | -0.0461 |
| C_1 | Gh_A09G1848 | NA | Cytochrome P450 CYP736A12 | -1.8501 | -2.6278 | -0.5702 | -0.7262 |
| C_1 | Gh_A09G1891 | NA | Cysteine synthase | -0.3277 | -0.6251 | -0.3629 | 0.1900 |
| C_1 | Gh_A09G1923 | ACA13 | Putative calcium-transporting ATPase 13, plasma | 0.4440 | 1.1862 | 1.7356 | 1.5399 |
|  |  |  | membrane-type |  |  |  |  |
| C_1 | Gh_A09G1981 | At1g06620 | 1-aminocyclopropane-1-carboxylate oxidase homolog 1 | 0.2022 | 1.2741 | 3.7638 | 0.8323 |
| C_1 | Gh_A09G2087 | ATL2 | RING-H2 finger protein ATL2 | -0.2362 | -0.6129 | 1.0462 | 1.1553 |
| C_1 | Gh_A09G2314 | BBX24 | B-box zinc finger protein 24 | 0.1240 | 0.2882 | 0.5111 | 0.2679 |
| C_1 | Gh_A09G2428 | PDC1 | Pyruvate decarboxylase 1 | 0.1721 | 0.8282 | 0.8384 | 0.4755 |
| C_1 | Gh_A10G0048 | TPS6 | Probable terpene synthase 6 | -2.8435 | -2.6672 | -0.1413 | -1.7170 |
| C_1 | Gh_A10G0115 | ANP2 | Mitogen-activated protein kinase kinase kinase 2 | 0.3386 | 0.5577 | 2.0959 | 1.1344 |
| C_1 | Gh_A10G0117 | ATG8C | Autophagy-related protein 8C | -0.7166 | -0.8391 | 0.5192 | 1.0419 |
| C_1 | Gh_A10G0161 | CRK10 | Cysteine-rich receptor-like protein kinase 10 | -0.1911 | -0.3704 | 0.2479 | 0.1083 |
| C_1 | Gh_A10G0169 | SD18 | Receptor-like serine/threonine-protein kinase SD1-8 | -2.1676 | -0.4728 | -0.3170 | 1.1718 |
| C_1 | Gh_A10G0255 | ERF4 | Ethylene-responsive transcription factor 4 | 0.2573 | 0.4007 | 0.9471 | 0.6684 |
| C_1 | Gh_A10G0348 | DOF1.5 | Dof zinc finger protein DOF1.5 | 0.0178 | -0.2001 | 1.6747 | 0.8940 |
| C_1 | Gh_A10G0371 | At4g18260 | Cytochrome b561 domain-containing protein | 0.9604 | 1.4144 | 1.2961 | 2.2539 |
| C_1 | Gh_A10G0537 | ABCC8 | ABC transporter C family member 8 | -0.8334 | -1.6206 | -0.3528 | 0.1177 |
| C_1 | Gh_A10G0563 | NA | NA | -0.0178 | -0.1166 | 0.1023 | 0.9718 |
| C_1 | Gh_A10G0601 | FAAH | Fatty acid amide hydrolase | -0.7991 | -0.4990 | 0.1739 | 0.2222 |
| C_1 | Gh_A10G0617 | PCMP-H92 | Pentatricopeptide repeat-containing protein | -2.8090 | -1.4796 | -0.1191 | 0.3028 |
| C_1 | Gh_A10G0739 | EME1B | Crossover junction endonuclease EME1B | 0.0442 | 0.1385 | 0.4675 | 0.5010 |
| C_1 | Gh_A10G0741 | ERF114 | Ethylene-responsive transcription factor | -0.2131 | -1.6221 | 2.8663 | 0.9312 |
| C_1 | Gh_A10G0817 | NA | NA | -0.3518 | -0.5983 | 1.4667 | 2.1694 |
| C_1 | Gh_A10G0818 | CYCD3-2 | Cyclin-D3-2 | -1.8033 | -0.7059 | 0.5280 | 0.0369 |
| C_1 | Gh_A10G0841 | NA | NA | 0.2140 | 1.9628 | 3.6011 | 3.9403 |
| C_1 | Gh_A10G0843 | At2g23790 | Calcium uniporter protein 2, mitochondrial | -1.7950 | 0.4544 | 1.2922 | 1.6986 |
| C_1 | Gh_A10G0878 | fosB | Metallothiol transferase FosB | -0.1409 | 0.5421 | 0.7187 | 0.8874 |
| C_1 | Gh_A10G1090 | NA | Glutathione reductase, cytosolic | -0.3203 | -0.5090 | 0.1233 | -0.1509 |
| C_1 | Gh_A10G1320 | ASP1 | Aspartate aminotransferase, mitochondrial | -0.5453 | 1.0146 | 1.8338 | 2.6921 |
| C_1 | Gh_A10G1378 | AMT3-1 | Ammonium transporter 3 member 1 | -1.8879 | -1.0785 | -1.1508 | 0.4327 |
| C_1 | Gh_A10G1447 | SLC25A19 | Mitochondrial thiamine pyrophosphate carrier | -1.1743 | 0.1128 | 0.1687 | -0.3472 |
| C_1 | Gh_A10G1520 | SAR1A | GTP-binding protein SAR1A | 0.0169 | 0.4054 | 0.5373 | 0.3540 |
| C_1 | Gh_A10G1598 | DXMT1 | 3,7-dimethylxanthine N-methyltransferase | -6.0541 | -2.0327 | 0.3605 | 0.7337 |
| C_1 | Gh_A10G1628 | NA | NA | 0.1404 | 1.3828 | 2.8754 | 2.6123 |
| C_1 | Gh_A10G1696 | ABCI17 | ABC transporter I family member 17 | -2.2445 | -1.4823 | -1.0858 | -0.5443 |
| C_1 | Gh_A10G1722 | CYP83B1 | Cytochrome P450 83B1 | -0.8706 | 0.8157 | 0.8131 | 0.8237 |
| C_1 | Gh_A10G1791 | NA | NA | -0.0937 | 0.1597 | 0.0706 | 0.1370 |
| C_1 | Gh_A10G1958 | NA | NA | -2.0439 | -2.7047 | 0.7151 | 0.4869 |
| C_1 | Gh_A10G1985 | PCS1 | Aspartic proteinase PCS1 | 0.4100 | 0.1403 | 1.8264 | 0.7941 |
| C_1 | Gh_A10G2006 | CYT1 | Mannose-1-phosphate guanylyltransferase 1 | -0.0693 | 0.2351 | 0.4640 | 0.1606 |
| C_1 | Gh_A10G2072 | CSA1 | Disease resistance-like protein CSA1 | -0.2699 | 0.4941 | 1.0054 | 1.2362 |
| C_1 | Gh_A10G2111 | NA | NA | -0.6848 | -0.9799 | 0.8830 | 0.1417 |
| C_1 | Gh_A10G2112 | UGT94E5 | Beta-D-glucosyl crocetin beta-1,6-glucosyltransferase | 1.0619 | 1.4534 | 4.3101 | 2.0177 |
| C_1 | Gh_A10G2123 | CCR3 | Putative serine/threonine-protein kinase-like protein CCR3 | -0.6662 | -0.5550 | 1.6503 | 1.5160 |
| C_1 | Gh_A10G2343 | SIB1 | Sigma factor binding protein 1, chloroplastic | -1.7883 | -1.2419 | 0.5676 | 0.7061 |
| C_1 | Gh_A11G0039 | DTX16 | Protein DETOXIFICATION 16 | -3.1905 | -1.9687 | -1.9272 | -0.8386 |
| C_1 | Gh_A11G0097 | arsB | Putative transporter arsB | -0.5370 | -0.7802 | 0.0068 | -0.2431 |
| C_1 | Gh_A11G0185 | ACR8 | ACT domain-containing protein ACR8 | 1.0595 | 1.5741 | 2.0410 | 1.4380 |
| C_1 | Gh_A11G0261 | BRG1 | BOI-related E3 ubiquitin-protein ligase 1 | -1.4051 | -1.0149 | 0.1649 | 0.0765 |
| C_1 | Gh_A11G0284 | SUD1 | Probable E3 ubiquitin ligase SUD1 | 0.0394 | -0.0629 | 1.3244 | -0.1508 |
| C_1 | Gh_A11G0297 | At1g74360 | Probable LRR receptor-like serine/threonine-protein | -0.5713 | -0.8424 | 0.8469 | 0.2509 |
|  |  |  | kinase |  |  |  |  |
| C_1 | Gh_A11G0316 | DOF5.4 | Dof zinc finger protein DOF5.4 | -0.0703 | 0.3095 | 0.8139 | 1.5595 |
| C_1 | Gh_A11G0335 | CML19 | Putative calcium-binding protein CML19 | 0.2570 | 0.3062 | 4.0587 | 2.6667 |
| C_1 | Gh_A11G0392 | NA | NA | 0.1772 | -0.4167 | 0.8990 | 0.8872 |
| C_1 | Gh_A11G0419 | NA | NA | 0.7554 | 1.2890 | 1.1594 | 1.1622 |
| C_1 | Gh_A11G0425 | PLP1 | Patatin-like protein 1 | -0.9139 | -0.7093 | 0.1730 | -1.1655 |
| C_1 | Gh_A11G0452 | VQ29 | VQ motif-containing protein 29 | -0.6407 | -0.9717 | 1.8779 | 1.1782 |
| C_1 | Gh_A11G0472 | NA | NA | -0.4876 | -0.5283 | 0.6555 | 0.1065 |
| C_1 | Gh_A11G0526 | csd | Probable cysteine desulfurase | 0.3144 | 0.2826 | 2.4241 | 2.4735 |
| C_1 | Gh_A11G0683 | NA | NA | -0.5059 | -0.0656 | 0.4059 | 1.2641 |
| C_1 | Gh_A11G0685 | ERF2 | Ethylene-responsive transcription factor 2 | -1.4161 | -2.4250 | 1.6027 | 1.0851 |
| C_1 | Gh_A11G0686 | ERF13 | Ethylene-responsive transcription factor 13 | -1.0889 | -1.0765 | 3.4381 | 0.9120 |
| C_1 | Gh_A11G0780 | At4g11680 | E3 ubiquitin-protein ligase | -1.2143 | -1.5349 | -0.1603 | 0.0737 |
| C_1 | Gh_A11G0910 | NA | NA | -4.9731 | -2.1057 | -2.1786 | -0.4911 |
| C_1 | Gh_A11G1017 | NA | NA | -3.2098 | -1.1484 | -1.4787 | -0.3390 |
| C_1 | Gh_A11G1019 | COBL7 | COBRA-like protein 7 | -0.7801 | -0.2969 | -0.3715 | 1.9709 |
| C_1 | Gh_A11G1124 | CYCP3-1 | Cyclin-P3-1 | 0.1795 | 1.2724 | 1.2981 | 1.2808 |
| C_1 | Gh_A11G1434 | CPX1 | Coproporphyrinogen-III oxidase 1, chloroplastic | -0.6101 | 0.5564 | 0.6605 | 0.6582 |
| C_1 | Gh_A11G1529 | At1g67000 | Probable receptor-like protein kinase | -0.0604 | -0.0196 | 0.9096 | 0.3274 |
| C_1 | Gh_A11G1587 | At5g10080 | Aspartic proteinase-like protein 1 | -0.5844 | -1.0145 | -0.1424 | -0.0968 |
| C_1 | Gh_A11G1684 | NA | NA | 0.6587 | 1.1192 | 1.6498 | 0.8441 |
| C_1 | Gh_A11G1729 | CML41 | Probable calcium-binding protein CML41 | -0.3254 | -1.2331 | 0.9231 | 0.6741 |
| C_1 | Gh_A11G1809 | NA | NA | -0.6972 | -0.7400 | 4.1824 | -0.2525 |
| C_1 | Gh_A11G1864 | PUMP5 | Mitochondrial uncoupling protein 5 | -0.0242 | -0.1375 | 0.6169 | 0.3383 |
| C_1 | Gh_A11G1968 | At5g67385 | BTB/POZ domain-containing protein | 0.1508 | 0.1105 | 2.0623 | 2.8320 |
| C_1 | Gh_A11G1972 | P4H9 | Probable prolyl 4-hydroxylase 9 | -0.2414 | 0.5081 | 1.3333 | 2.0837 |
| C_1 | Gh_A11G1997 | CYP81E8 | Cytochrome P450 81E8 | 0.2039 | -0.2730 | 0.5839 | 0.8448 |
| C_1 | Gh_A11G1999 | HDHD3 | Haloacid dehalogenase-like hydrolase domain-containing | 0.6140 | 0.7325 | 1.0509 | 0.7106 |
|  |  |  | protein 3 |  |  |  |  |
| C_1 | Gh_A11G2181 | NA | NA | 0.7289 | 1.4222 | 1.8775 | 2.1768 |
| C_1 | Gh_A11G2264 | MYOB1 | Myosin-binding protein 1 | -0.1710 | 0.0500 | 0.4229 | -0.0954 |
| C_1 | Gh_A11G2286 | HEV1 | Pro-hevein | -0.6196 | -0.1764 | 0.2885 | -0.3198 |
| C_1 | Gh_A11G2314 | guaD | Guanine deaminase | -0.8346 | -0.1478 | 0.4036 | -0.2414 |
| C_1 | Gh_A11G2399 | PNC1 | Peroxisomal adenine nucleotide carrier 1 | -0.3408 | 0.9069 | 1.3574 | 0.9434 |
| C_1 | Gh_A11G2401 | ANP1 | Mitogen-activated protein kinase kinase kinase ANP1 | -0.1544 | -0.0969 | 0.6659 | 0.1682 |
| C_1 | Gh_A11G2455 | PP2B15 | F-box protein PP2-B15 | -0.4788 | 0.8246 | 1.6348 | 1.6621 |
| C_1 | Gh_A11G2472 | At3g01520 | Universal stress protein A-like protein | 0.4067 | 1.6552 | 1.7662 | 1.6744 |
| C_1 | Gh_A11G2547 | NA | NA | -0.5460 | 0.2757 | 0.1864 | 0.0680 |
| C_1 | Gh_A11G2559 | At4g14610 | Probable disease resistance protein | -0.0860 | 0.0593 | 0.5847 | 0.2929 |
| C_1 | Gh_A11G2650 | NA | NA | -0.4578 | 0.1884 | 0.4055 | 0.6239 |
| C_1 | Gh_A11G2685 | DAPB2 | 4-hydroxy-tetrahydrodipicolinate reductase 2, | -0.5393 | -0.5729 | 0.0368 | -0.2375 |
|  |  |  | chloroplastic |  |  |  |  |
| C_1 | Gh_A11G2705 | NA | NA | -0.8549 | -0.1853 | 0.2884 | -0.3173 |
| C_1 | Gh_A11G2727 | NA | NA | -0.1192 | 0.1232 | 8.4119 | -0.2513 |
| C_1 | Gh_A11G2733 | CAMBP25 | Calmodulin-binding protein 25 | -0.7563 | -0.9898 | 0.0486 | 0.0038 |
| C_1 | Gh_A11G2759 | Ncapd2 | Condensin complex subunit 1 | -0.4970 | 0.1086 | 0.0763 | 0.2630 |
| C_1 | Gh_A11G2762 | At3g02645 | Putative UPF0481 protein | -0.4247 | -0.2928 | -0.2119 | -0.3288 |
| C_1 | Gh_A11G2835 | RGA3 | Putative disease resistance protein RGA3 | -2.2554 | -2.2599 | 4.3268 | -0.2526 |
| C_1 | Gh_A11G2859 | NA | NA | -0.6505 | -0.2823 | 0.9070 | -1.2818 |
| C_1 | Gh_A11G2870 | HDT1 | Histone deacetylase HDT1 | 0.1034 | 0.1869 | 0.6017 | 0.1686 |
| C_1 | Gh_A11G3068 | NA | NA | 2.0757 | 2.3629 | 6.9401 | 5.3061 |
| C_1 | Gh_A11G3073 | BZIP53 | bZIP transcription factor 53 | -0.4860 | -0.1602 | 0.8170 | 0.9641 |
| C_1 | Gh_A11G3075 | NA | NA | -0.5202 | -0.3411 | -0.2001 | 0.5692 |
| C_1 | Gh_A11G3090 | PUB21 | U-box domain-containing protein 21 | -1.0376 | -1.6188 | 1.1710 | 0.1836 |
| C_1 | Gh_A11G3216 | NA | Cytochrome P450 CYP73A100 | 0.3356 | 1.3392 | 4.3737 | 3.5629 |
| C_1 | Gh_A11G3286 | At3g14580 | Pentatricopeptide repeat-containing protein | -1.2952 | 0.7829 | 0.6229 | -0.0900 |
| C_1 | Gh_A12G0100 | AAP19-2 | AP-1 complex subunit sigma-2 | -1.8133 | -0.1630 | -0.3994 | -0.3271 |
| C_1 | Gh_A12G0109 | CPK4 | Calcium-dependent protein kinase 4 | -0.0962 | -0.5886 | 0.4269 | 0.9007 |
| C_1 | Gh_A12G0206 | At5g67130 | PI-PLC X domain-containing protein | -1.3044 | -0.6052 | -0.0957 | -0.6500 |
| C_1 | Gh_A12G0365 | NA | NA | -0.5285 | 0.2150 | 1.4600 | 0.9588 |
| C_1 | Gh_A12G0394 | At1g07650 | Probable LRR receptor-like serine/threonine-protein | -0.9895 | -0.9532 | -0.6125 | -0.4663 |
|  |  |  | kinase |  |  |  |  |
| C_1 | Gh_A12G0498 | NA | NA | -0.7510 | -1.0412 | -0.1100 | -0.5727 |
| C_1 | Gh_A12G0501 | NA | NA | -0.5375 | -0.7009 | 0.8832 | 0.8108 |
| C_1 | Gh_A12G0548 | NA | NA | 0.0249 | 0.3829 | 1.9934 | 2.2807 |
| C_1 | Gh_A12G0550 | NA | NA | -0.2858 | 0.6885 | 0.6125 | 0.3705 |
| C_1 | Gh_A12G0589 | NA | NA | 0.0637 | -0.3154 | 0.9852 | 0.2540 |
| C_1 | Gh_A12G0683 | NA | NA | -0.0665 | 0.2416 | 0.1501 | 0.7012 |
| C_1 | Gh_A12G0725 | RAX3 | Transcription factor RAX3 | -0.6579 | -0.2376 | 0.4303 | -0.6309 |
| C_1 | Gh_A12G0729 | NA | NA | -0.1879 | 0.0704 | 0.1508 | 1.6414 |
| C_1 | Gh_A12G0875 | ERF13 | Ethylene-responsive transcription factor 13 | -0.0186 | -2.0180 | 3.2680 | 1.5371 |
| C_1 | Gh_A12G0876 | ERF13 | Ethylene-responsive transcription factor 13 | -1.1861 | -3.0670 | 4.1966 | 2.7912 |
| C_1 | Gh_A12G0969 | RHA1B | E3 ubiquitin-protein ligase | -1.1130 | 0.1143 | 1.2490 | 1.3097 |
| C_1 | Gh_A12G1150 | NA | NA | -0.0853 | 0.0916 | 0.4009 | 0.4021 |
| C_1 | Gh_A12G1194 | EID1 | Phytochrome A-associated F-box protein | 0.2696 | 0.4334 | 0.8517 | 0.4021 |
| C_1 | Gh_A12G1249 | 3AT1 | Coumaroyl-CoA:anthocyanidin 3-O-glucoside-6''-O- | 0.9883 | 1.9179 | 3.5337 | 2.8901 |
|  |  |  | coumaroyltransferase 1 |  |  |  |  |
| C_1 | Gh_A12G1439 | Esyt3 | Extended synaptotagmin-3 | -0.0388 | 1.3373 | 1.2800 | 0.7737 |
| C_1 | Gh_A12G1570 | IDL1 | Protein IDA-LIKE 1 | 0.0439 | 0.1162 | 0.7491 | 0.8300 |
| C_1 | Gh_A12G1589 | NA | Dynein light chain LC6, flagellar outer arm | 0.0311 | -0.1174 | 0.4803 | 0.0778 |
| C_1 | Gh_A12G1661 | COB | Protein COBRA | 0.0732 | 0.2788 | 0.4042 | 0.2282 |
| C_1 | Gh_A12G1788 | At5g07610 | F-box protein | -1.3657 | -3.5186 | 0.9027 | 0.5640 |
| C_1 | Gh_A12G1917 | TOM2A | Tobamovirus multiplication protein 2A | -1.2225 | -0.1469 | 1.2170 | 0.4741 |
| C_1 | Gh_A12G1935 | NA | NA | -0.4740 | 0.0646 | 1.8711 | 2.3780 |
| C_1 | Gh_A12G2024 | SMO1-1 | Methylsterol monooxygenase 1-1 | -0.0782 | 0.4519 | 0.7777 | 0.3513 |
| C_1 | Gh_A12G2121 | WRKY53 | Probable WRKY transcription factor 53 | 0.4257 | -0.3481 | 2.8453 | 1.8150 |
| C_1 | Gh_A12G2188 | COMT1 | Caffeic acid 3-O-methyltransferase | -0.5331 | -1.2296 | -0.1597 | 0.4068 |
| C_1 | Gh_A12G2201 | NUP98A | Nuclear pore complex protein | 0.3017 | 0.7758 | 1.3302 | 0.6072 |
| C_1 | Gh_A12G2441 | TIFY6B | Protein TIFY 6B | 0.3030 | 0.3368 | 0.6591 | 0.5101 |
| C_1 | Gh_A12G2449 | PUB21 | U-box domain-containing protein 21 | -2.9994 | -2.5259 | 1.7088 | 2.2977 |
| C_1 | Gh_A12G2480 | SRO5 | Probable inactive poly [ADP-ribose] polymerase SRO5 | 0.4607 | 1.2024 | 1.7444 | 1.1152 |
| C_1 | Gh_A13G0037 | NA | NA | -0.5992 | -0.2071 | 0.9333 | 0.3279 |
| C_1 | Gh_A13G0056 | NA | NA | -0.5363 | -1.5564 | 1.8299 | 0.9826 |
| C_1 | Gh_A13G0095 | NA | NA | -0.0355 | 0.8251 | 1.0241 | 0.5453 |
| C_1 | Gh_A13G0154 | CYP714C2 | Cytochrome P450 714C2 | -1.2861 | -1.3773 | 1.4454 | -1.0747 |
| C_1 | Gh_A13G0196 | ZAT11 | Zinc finger protein ZAT11 | -0.5065 | -0.8203 | 0.4556 | 0.3384 |
| C_1 | Gh_A13G0228 | NA | NA | -0.4116 | -0.8590 | 2.1385 | 0.2667 |
| C_1 | Gh_A13G0301 | NA | Guanine nucleotide-binding protein subunit beta-2 | -0.3469 | -0.6705 | 0.0580 | 0.3151 |
| C_1 | Gh_A13G0350 | LAG2 | LAG1 longevity assurance homolog 2 | -0.4625 | -0.3565 | 1.3247 | 1.3507 |
| C_1 | Gh_A13G0581 | ST2 | High affinity sulfate transporter 2 | -5.9103 | -4.5571 | -2.4763 | -3.3254 |
| C_1 | Gh_A13G0735 | RPK2 | LRR receptor-like serine/threonine-protein kinase | -0.6025 | -1.0705 | 1.1590 | 1.7672 |
| C_1 | Gh_A13G0831 | KN | Syntaxin-related protein KNOLLE | 0.1799 | -0.1120 | 0.6520 | 0.3607 |
| C_1 | Gh_A13G0853 | NA | NA | -1.1189 | -1.5528 | -0.1564 | -0.6710 |
| C_1 | Gh_A13G0862 | XRN4 | 5'-3' exoribonuclease 4 | -0.0738 | 0.0342 | 0.5504 | 0.2653 |
| C_1 | Gh_A13G1043 | MYB4 | Myb-related protein Myb4 | -1.7277 | -2.0765 | 1.9732 | -0.4569 |
| C_1 | Gh_A13G1052 | NA | NA | 0.2044 | -0.1235 | 0.9682 | 1.3544 |
| C_1 | Gh_A13G1087 | COX15 | Cytochrome c oxidase assembly protein COX15 | 0.1454 | 0.3222 | 0.5657 | 0.5423 |
| C_1 | Gh_A13G1352 | At3g47200 | UPF0481 protein | -2.4060 | -1.8615 | -1.4820 | 1.3526 |
| C_1 | Gh_A13G1401 | MYB44 | Transcription factor MYB44 | -4.5919 | -3.8374 | -1.0360 | -1.2131 |
| C_1 | Gh_A13G1526 | ATL6 | E3 ubiquitin-protein ligase ATL6 | -0.3592 | -1.0885 | 1.0348 | 0.4688 |
| C_1 | Gh_A13G1612 | NA | NA | 0.7172 | 0.5857 | 1.4302 | 1.1751 |
| C_1 | Gh_A13G1659 | SLC25A44 | Solute carrier family 25 member 44 | -0.0781 | 1.2245 | 1.9468 | 3.5402 |
| C_1 | Gh_A13G1668 | COG3 | Conserved oligomeric Golgi complex subunit 3 | -0.3154 | -0.2694 | -0.1651 | 0.0932 |
| C_1 | Gh_A13G1685 | At5g01020 | Serine/threonine-protein kinase | 0.3006 | -0.0735 | 1.3237 | 0.6766 |
| C_1 | Gh_A13G1707 | XI-K | Myosin-17 | -0.0999 | -0.1500 | 0.8111 | -0.4392 |
| C_1 | Gh_A13G1727 | VPS32.2 | Vacuolar protein sorting-associated protein 32 homolog 2 | -1.4735 | -1.9734 | -1.3934 | -0.3481 |
| C_1 | Gh_A13G1741 | ABI1 | Protein phosphatase 2C 56 | -1.1689 | -1.2126 | 0.1344 | -0.2180 |
| C_1 | Gh_A13G1747 | cfxQ | Protein cfxQ homolog | -0.0733 | 0.8663 | 0.7727 | 1.1355 |
| C_1 | Gh_A13G1847 | MTP4 | Metal tolerance protein 4 | -1.1251 | -0.8881 | -0.9692 | -0.4679 |
| C_1 | Gh_A13G1903 | NFS2 | Cysteine desulfurase 1, chloroplastic | -0.1973 | -0.1424 | 0.5341 | -0.1964 |
| C_1 | Gh_A13G2033 | At2g30020 | Probable protein phosphatase 2C 25 | -0.1258 | 0.4947 | 1.2941 | 1.4787 |
| C_1 | Gh_A13G2112 | ZAT10 | Zinc finger protein ZAT10 | -0.1267 | -0.2941 | 0.7662 | 0.4957 |
| C_1 | Gh_A13G2264 | NA | NA | -0.4840 | -1.4317 | 1.4725 | 1.4009 |
| C_1 | Gh_A13G2293 | NA | NA | -0.4964 | -0.7238 | 1.1726 | 1.5234 |
| C_1 | Gh_D01G0008 | AC101 | Actin-101 | 0.1569 | 0.3957 | 1.2827 | 0.0886 |
| C_1 | Gh_D01G0012 | ANAC094 | Putative NAC domain-containing protein 94 | -1.3628 | -2.8522 | 1.7004 | 1.2850 |
| C_1 | Gh_D01G0098 | PLT5 | Polyol transporter 5 | -1.9231 | -1.3912 | -0.5443 | -0.3031 |
| C_1 | Gh_D01G0188 | PUB42 | Putative U-box domain-containing protein 42 | 0.4768 | -0.5087 | 1.7009 | 1.3221 |
| C_1 | Gh_D01G0225 | MRS2-2 | Magnesium transporter MRS2-2 | -4.6556 | -3.9597 | -0.1106 | -0.3577 |
| C_1 | Gh_D01G0228 | ECI3 | Enoyl-CoA delta isomerase 3 | -0.3541 | -0.7663 | -0.1866 | 0.4510 |
| C_1 | Gh_D01G0246 | At1g56140 | Probable LRR receptor-like serine/threonine-protein kinase | -0.0330 | -0.1732 | 1.0672 | 0.4122 |
| C_1 | Gh_D01G0294 | VQ1 | VQ motif-containing protein 1 | -2.0688 | -2.5862 | 1.3419 | 1.2922 |
| C_1 | Gh_D01G0297 | rpoB | DNA-directed RNA polymerase subunit beta | -0.5841 | 1.0072 | 0.9804 | 0.2907 |
| C_1 | Gh_D01G0322 | GDPDL2 | Glycerophosphodiester phosphodiesterase protein kinase | 0.1772 | 0.1421 | 1.6658 | 1.1962 |
|  |  |  | domain-containing |  |  |  |  |
| C_1 | Gh_D01G0331 | CYP72A15 | Cytochrome P450 72A15 | -0.8323 | -1.4772 | -0.1567 | 0.4116 |
| C_1 | Gh_D01G0439 | NA | NA | -0.1044 | 0.5633 | 0.7845 | 0.8958 |
| C_1 | Gh_D01G0502 | CBSX5 | CBS domain-containing protein CBSX5 | 0.8516 | 0.6744 | 1.5867 | 0.9423 |
| C_1 | Gh_D01G0514 | NAC072 | NAC domain-containing protein 72 | 0.0549 | 0.4627 | 1.7485 | 0.5275 |
| C_1 | Gh_D01G0551 | PBP1 | Calcium-binding protein | -0.4948 | -0.2388 | 1.2943 | 1.2679 |
| C_1 | Gh_D01G0559 | GH3.6 | Indole-3-acetic acid-amido synthetase | -1.3044 | -1.4097 | -1.1308 | -0.4385 |
| C_1 | Gh_D01G0583 | NDR1 | Protein NDR1 | 0.1328 | -0.4805 | 1.0634 | 0.8098 |
| C_1 | Gh_D01G0646 | NA | NA | -0.1659 | 0.0055 | 2.1050 | 1.2781 |
| C_1 | Gh_D01G0657 | WRKY6 | WRKY transcription factor 6 | -0.4978 | -0.6533 | 0.5673 | 0.4366 |
| C_1 | Gh_D01G0718 | SAT3 | Serine acetyltransferase 3, mitochondrial | -0.5594 | 0.1726 | 1.4800 | 0.4080 |
| C_1 | Gh_D01G0915 | PLDDELTA | Phospholipase D delta | -0.1487 | 0.3590 | 0.8027 | 0.4960 |
| C_1 | Gh_D01G1075 | NA | Squalene monooxygenase | 1.0033 | 0.7995 | 3.2717 | 1.5184 |
| C_1 | Gh_D01G1411 | At2g01630 | Glucan endo-1,3-beta-glucosidase 3 | 0.0216 | 0.0058 | 0.6224 | 0.5285 |
| C_1 | Gh_D01G1418 | PCR2 | Protein PLANT CADMIUM RESISTANCE 2 | -0.4595 | 0.0107 | 0.3657 | 0.3602 |
| C_1 | Gh_D01G1419 | PCR2 | Protein PLANT CADMIUM RESISTANCE 2 | -1.0486 | -0.8635 | 0.2573 | 0.1000 |
| C_1 | Gh_D01G1437 | CRK19 | Cysteine-rich receptor-like protein kinase 19 | -0.1022 | -0.4840 | 1.9096 | 1.5908 |
| C_1 | Gh_D01G1578 | CXE11 | Probable carboxylesterase 11 | -0.2438 | -0.6084 | 0.9162 | 0.9201 |
| C_1 | Gh_D01G1753 | IKU2 | Receptor-like protein kinase HAIKU2 | -0.0339 | 0.4612 | 0.9141 | 0.3689 |
| C_1 | Gh_D01G1852 | CML44 | Probable calcium-binding protein | -0.6958 | -0.9598 | 0.0892 | 0.1094 |
| C_1 | Gh_D01G1925 | DTX42 | Protein DETOXIFICATION 42 | -8.5226 | -4.4130 | -0.5746 | -0.7113 |
| C_1 | Gh_D01G1977 | LOG7 | Cytokinin riboside 5'-monophosphate | -1.4848 | -1.5942 | 0.4405 | 0.4862 |
|  |  |  | phosphoribohydrolase |  |  |  |  |
| C_1 | Gh_D01G2237 | RLP12 | Receptor-like protein 12 | -0.6977 | -0.9780 | 0.7199 | 1.2243 |
| C_1 | Gh_D01G2258 | FBL3 | F-box/LRR-repeat protein 3 | -0.0839 | -0.3086 | 1.4458 | -0.1598 |
| C_1 | Gh_D01G2305 | At5g49610 | F-box protein | 0.1833 | 0.0027 | 1.0511 | 0.0404 |
| C_1 | Gh_D01G2347 | NA | NA | -0.1119 | -0.3540 | 1.5394 | 1.4136 |
| C_1 | Gh_D02G0174 | NA | NA | -1.8644 | -2.2546 | -1.4990 | -0.3404 |
| C_1 | Gh_D02G0201 | WAKL1 | Wall-associated receptor kinase-like 1 | -1.7184 | -0.3651 | 2.0989 | 1.0826 |
| C_1 | Gh_D02G0214 | CBDAS2 | Cannabidiolic acid synthase-like 1 | -1.6952 | -0.3419 | -0.0265 | 0.3576 |
| C_1 | Gh_D02G0215 | CBDAS2 | Cannabidiolic acid synthase-like 1 | -1.3344 | 0.4855 | 0.1373 | 0.3099 |
| C_1 | Gh_D02G0218 | NA | NA | -2.6699 | -2.2791 | -0.2110 | -1.2025 |
| C_1 | Gh_D02G0227 | UGT75L6 | Crocetin glucosyltransferase, chloroplastic | 1.5772 | 0.7606 | 2.1190 | 3.3186 |
| C_1 | Gh_D02G0256 | At4g13800 | Probable magnesium transporter NIPA2 | 0.0938 | -0.5075 | 0.7389 | 0.7093 |
| C_1 | Gh_D02G0264 | PHT1-5 | Probable inorganic phosphate transporter 1-5 | -1.4717 | -1.2001 | -0.2229 | 2.0082 |
| C_1 | Gh_D02G0268 | Mettl13 | Methyltransferase-like protein 13 | -0.4598 | 0.0588 | 0.5394 | -0.1403 |
| C_1 | Gh_D02G0348 | TSJT1 | Stem-specific protein TSJT1 | -0.8229 | -0.7185 | -0.5926 | -0.0852 |
| C_1 | Gh_D02G0367 | CALS12 | Callose synthase 12 | -2.2128 | -2.1120 | -0.6550 | -0.3975 |
| C_1 | Gh_D02G0385 | RLP12 | Receptor-like protein 12 | -0.7610 | -0.3695 | 0.1102 | -0.0536 |
| C_1 | Gh_D02G0472 | PCMP-H87 | Pentatricopeptide repeat-containing protein | -0.2780 | 0.1696 | 0.4772 | 0.1183 |
| C_1 | Gh_D02G0510 | NA | Lipase | -0.4871 | -0.3046 | 0.1178 | -0.0215 |
| C_1 | Gh_D02G0659 | RAP | Aspartic proteinase | 1.2334 | -1.3010 | 5.4706 | 4.7292 |
| C_1 | Gh_D02G0692 | NA | NA | 0.0937 | 1.4350 | 1.3194 | 1.0768 |
| C_1 | Gh_D02G0703 | NA | NA | 0.2594 | 0.2223 | 1.3571 | 0.4807 |
| C_1 | Gh_D02G0768 | WAK2 | Wall-associated receptor kinase 2 | 0.0323 | -1.7159 | 2.3714 | 1.4803 |
| C_1 | Gh_D02G1018 | At1g65420 | Ycf20-like protein | -0.4635 | -0.8670 | 1.1732 | 0.2490 |
| C_1 | Gh_D02G1067 | FAH1 | Fatty acid 2-hydroxylase 1 | -0.6089 | 0.3325 | 0.3119 | 1.2175 |
| C_1 | Gh_D02G1179 | NA | NA | -0.8793 | -1.2154 | 2.5837 | 0.1255 |
| C_1 | Gh_D02G1555 | ABCC3 | ABC transporter C family member 3 | -1.7949 | -2.1301 | 1.1147 | -2.2908 |
| C_1 | Gh_D02G1619 | RPL27AC | 60S ribosomal protein L27a-3 | 0.1001 | 1.4534 | 9.1800 | -0.2525 |
| C_1 | Gh_D02G1728 | LBD42 | LOB domain-containing protein 42 | 0.0342 | 0.6254 | 2.5118 | 1.8542 |
| C_1 | Gh_D02G1735 | IST1 | IST1 homolog | -0.4291 | -0.2788 | -0.2230 | -0.1656 |
| C_1 | Gh_D02G1756 | NA | NA | -0.7259 | -1.5847 | 2.0585 | 0.9105 |
| C_1 | Gh_D02G1776 | TIFY9 | Protein TIFY 9 | -0.0382 | 0.0195 | 1.9032 | 0.6874 |
| C_1 | Gh_D02G1879 | MYB4 | Myb-related protein Myb4 | -0.0567 | -1.8585 | 3.7814 | 1.3531 |
| C_1 | Gh_D02G1929 | TMEM53 | Transmembrane protein 53 | -0.0386 | -0.2259 | 2.3041 | 1.7476 |
| C_1 | Gh_D02G1931 | At5g08350 | GEM-like protein 4 | 0.3881 | 0.7928 | 1.5547 | 0.3084 |
| C_1 | Gh_D02G2153 | RER1B | Protein RER1B | -0.4050 | -0.0087 | 0.7256 | -0.0107 |
| C_1 | Gh_D02G2248 | PIP2-8 | Probable aquaporin PIP2-8 | 0.3063 | 0.0944 | 1.4807 | 4.9947 |
| C_1 | Gh_D02G2286 | CHIA1 | Endochitinase 1 | 0.0551 | 0.9584 | 3.0128 | 1.2214 |
| C_1 | Gh_D02G2336 | NA | NA | 0.1258 | 1.0874 | 2.9877 | 2.2943 |
| C_1 | Gh_D02G2369 | PID | Protein kinase PINOID | -0.6230 | -0.2507 | 0.2345 | -0.0501 |
| C_1 | Gh_D02G2387 | NA | Cytochrome P450 CYP749A22 | -0.4597 | -0.3372 | 0.1242 | -0.5857 |
| C_1 | Gh_D02G2393 | CSLC12 | Probable xyloglucan glycosyltransferase 12 | 0.5688 | 0.9788 | 1.0902 | 0.8684 |
| C_1 | Gh_D02G2414 | NA | NA | -2.0028 | 0.0515 | -0.2108 | -0.2443 |
| C_1 | Gh_D03G0288 | At3g49720 | Uncharacterized protein | -0.0973 | 0.3316 | 1.1140 | 0.2615 |
| C_1 | Gh_D03G0311 | PCKA | Phosphoenolpyruvate carboxykinase [ATP] | -0.3745 | 0.2058 | -0.0084 | 0.2451 |
| C_1 | Gh_D03G0431 | SEP | Stress enhanced protein 2, chloroplastic | -0.4736 | -0.8623 | -0.3206 | 0.7137 |
| C_1 | Gh_D03G0454 | NA | NA | -1.4677 | -1.4935 | 0.9769 | 0.8572 |
| C_1 | Gh_D03G0509 | NA | NA | -0.5073 | -0.4328 | 0.1405 | -0.2260 |
| C_1 | Gh_D03G0758 | NA | NA | -0.7413 | -0.5410 | 0.8133 | 0.7634 |
| C_1 | Gh_D03G0782 | CYP78A3 | Cytochrome P450 78A3 | -0.1924 | -0.1835 | 1.0543 | 0.0736 |
| C_1 | Gh_D03G0992 | GDH2 | Glutamate dehydrogenase 2 | -0.7244 | -0.0526 | -0.2722 | 0.1297 |
| C_1 | Gh_D03G1185 | SPAC24B11.0 | Uncharacterized protein C24B11.05 | 0.5607 | 0.5506 | 1.9854 | 0.8727 |
|  |  | 5 |  |  |  |  |  |
| C_1 | Gh_D03G1232 | UXS5 | UDP-glucuronic acid decarboxylase 5 | 0.2734 | 0.1006 | 0.2173 | 0.5445 |
| C_1 | Gh_D03G1247 | ZAT12 | Zinc finger protein ZAT12 | -0.3024 | -1.5180 | 1.6022 | 1.0352 |
| C_1 | Gh_D03G1262 | EXO70A1 | Exocyst complex component | 0.2768 | -0.2646 | 2.6197 | 1.9416 |
| C_1 | Gh_D03G1307 | NRT2.1 | High-affinity nitrate transporter 2.1 | -1.2213 | -1.4043 | 0.7751 | -1.0428 |
| C_1 | Gh_D03G1308 | NRT2.3 | High affinity nitrate transporter 2.3 | 0.5041 | -2.8136 | 4.5257 | 5.3082 |
| C_1 | Gh_D03G1332 | MIEL1 | E3 ubiquitin-protein ligase MIEL1 | -0.3755 | 0.6255 | 0.6129 | 0.4182 |
| C_1 | Gh_D03G1372 | FBL3 | F-box/LRR-repeat protein 3 | -1.2812 | -1.0744 | -0.1895 | -0.3993 |
| C_1 | Gh_D03G1410 | PURA1 | Transcription factor Pur-alpha 1 | -0.7318 | -0.2164 | -0.3673 | 0.1362 |
| C_1 | Gh_D03G1479 | IQD14 | Protein IQ-DOMAIN 14 | -0.0075 | 0.2213 | 1.0159 | 0.6563 |
| C_1 | Gh_D03G1480 | EDR1 | Serine/threonine-protein kinase EDR1 | -0.3203 | -0.3253 | 0.2459 | 0.4858 |
| C_1 | Gh_D03G1493 | MADS50 | MADS-box transcription factor 50 | -3.7350 | -1.6108 | 3.7347 | -1.2202 |
| C_1 | Gh_D03G1511 | NA | NA | -1.8512 | -1.8378 | 1.8010 | -0.0889 |
| C_1 | Gh_D03G1512 | CYP71Z7 | Ent-cassadiene C2-hydroxylase | -1.0362 | -1.4843 | 0.6637 | -0.7307 |
| C_1 | Gh_D03G1520 | PHR1 | Deoxyribodipyrimidine photo-lyase | -2.1900 | -0.8360 | -0.0820 | -0.5967 |
| C_1 | Gh_D03G1809 | CXE18 | Probable carboxylesterase 18 | 0.0070 | -0.5447 | 0.8633 | 1.2279 |
| C_1 | Gh_D03G1840 | 4CLL6 | 4-coumarate--CoA ligase-like 6 | -1.3721 | -1.0953 | 0.9486 | -0.5316 |
| C_1 | Gh_D03G1841 | PLGG1 | Plastidal glycolate/glycerate translocator 1, chloroplastic | -1.7529 | 0.0979 | 3.4704 | 3.6565 |
| C_1 | Gh_D04G0059 | PIRL6 | Plant intracellular Ras-group-related LRR protein 6 | 0.5293 | -0.4884 | 1.3222 | 1.8300 |
| C_1 | Gh_D04G0135 | NA | NA | 0.4519 | -0.0029 | 3.9071 | 3.1673 |
| C_1 | Gh_D04G0168 | ALA1 | Phospholipid-transporting ATPase 1 | 0.1961 | -0.3375 | 1.3731 | 0.5127 |
| C_1 | Gh_D04G0188 | At1g65240 | Aspartic proteinase-like protein 2 | 0.1536 | 0.0299 | 0.9855 | 0.2350 |
| C_1 | Gh_D04G0205 | At1g05000 | Probable tyrosine-protein phosphatase | -0.3882 | -1.6151 | 1.7642 | 0.5296 |
| C_1 | Gh_D04G0272 | RABC1 | Ras-related protein RABC1 | -1.7505 | -2.1901 | -0.4764 | -1.2284 |
| C_1 | Gh_D04G0354 | HSFA4B | Heat stress transcription factor A-4b | -0.5855 | -0.5266 | 0.7394 | 0.2443 |
| C_1 | Gh_D04G0438 | CML45 | Probable calcium-binding protein | -0.9585 | -0.8722 | 1.1843 | 1.2819 |
| C_1 | Gh_D04G0536 | RPS17D | 40S ribosomal protein S17-4 | 0.0261 | -0.4919 | 0.7539 | 0.4009 |
| C_1 | Gh_D04G0893 | SYP121 | Syntaxin-121 | 0.0811 | 0.0900 | 1.1546 | 0.7482 |
| C_1 | Gh_D04G0970 | NA | NA | -0.1592 | -0.3397 | -0.0514 | 0.1695 |
| C_1 | Gh_D04G1153 | NA | NA | 0.3307 | 0.3833 | 0.8488 | 0.5541 |
| C_1 | Gh_D04G1165 | NA | NA | -0.7232 | -0.6015 | 0.0552 | 0.0104 |
| C_1 | Gh_D04G1187 | LECRKS4 | L-type lectin-domain containing receptor kinase S.4 | -0.1405 | 0.1423 | 0.2653 | 0.1008 |
| C_1 | Gh_D04G1223 | LAC7 | Laccase-7 | -1.1090 | -1.6092 | 1.4277 | 1.1356 |
| C_1 | Gh_D04G1311 | TBL35 | Protein trichome birefringence-like 35 | -0.0228 | -0.9342 | 1.0776 | 1.6394 |
| C_1 | Gh_D04G1318 | WRKY33 | Probable WRKY transcription factor 33 | -0.4558 | -0.7744 | 1.2242 | 1.0037 |
| C_1 | Gh_D04G1337 | NA | NA | -1.3488 | -1.1171 | -0.5032 | 0.0185 |
| C_1 | Gh_D04G1399 | STH-2 | Pathogenesis-related protein STH-2 | -0.1757 | -0.4463 | -0.0386 | 0.2493 |
| C_1 | Gh_D04G1542 | YLR126C | Putative glutamine amidotransferase | -1.3441 | 0.4328 | 0.7636 | -0.4493 |
| C_1 | Gh_D04G1677 | FMO1 | Probable flavin-containing monooxygenase 1 | -1.8901 | -0.8153 | 0.0411 | -0.3578 |
| C_1 | Gh_D04G1699 | IKU2 | Receptor-like protein kinase HAIKU2 | -1.3256 | -1.5624 | 0.3095 | 0.5913 |
| C_1 | Gh_D04G1714 | XLG1 | Extra-large guanine nucleotide-binding protein 1 | -0.0753 | 0.0153 | 1.1442 | 0.9723 |
| C_1 | Gh_D04G1718 | NA | NA | -1.3410 | -2.4074 | 1.3497 | -0.5345 |
| C_1 | Gh_D04G1778 | NA | NA | -0.5478 | -1.5483 | 0.3485 | 0.5179 |
| C_1 | Gh_D04G1904 | CDF1 | Cyclic dof factor 1 | -0.3452 | 0.0316 | 1.8681 | 1.2610 |
| C_1 | Gh_D04G1938 | SIB1 | Sigma factor binding protein 1, chloroplastic | -0.5942 | -1.3979 | 0.8162 | 0.3843 |
| C_1 | Gh_D05G0061 | At4g30420 | WAT1-related protein | -0.2149 | 1.0885 | 8.4464 | -0.2525 |
| C_1 | Gh_D05G0173 | NA | Early nodulin-93 | 0.0412 | 1.3860 | 6.8078 | 3.4230 |
| C_1 | Gh_D05G0174 | NA | Early nodulin-93 | -0.6417 | 1.0570 | 1.7600 | 1.6096 |
| C_1 | Gh_D05G0263 | At5g56590 | Glucan endo-1,3-beta-glucosidase 13 | -1.5580 | -1.5036 | -0.0164 | 0.1415 |
| C_1 | Gh_D05G0308 | IQM5 | IQ domain-containing protein IQM5 | -0.0445 | -1.1363 | 1.8956 | 0.7516 |
| C_1 | Gh_D05G0405 | NA | NA | -0.8616 | 0.2938 | 0.4948 | 0.3036 |
| C_1 | Gh_D05G0487 | GLIP1 | GDSL esterase/lipase 1 | -0.3674 | 0.2799 | 1.2779 | -0.1994 |
| C_1 | Gh_D05G0511 | NA | NA | -1.1104 | 0.1722 | -0.3298 | 0.1850 |
| C_1 | Gh_D05G0514 | NA | NA | -0.5570 | 0.0083 | 0.3341 | 0.7926 |
| C_1 | Gh_D05G0563 | NA | NA | -0.1470 | 0.8115 | 1.0173 | 0.6192 |
| C_1 | Gh_D05G0581 | At1g80640 | Probable receptor-like protein kinase | -0.9650 | 2.5953 | 2.9165 | 2.0587 |
| C_1 | Gh_D05G0651 | NA | NA | -0.0315 | 0.1243 | 1.3403 | 1.2808 |
| C_1 | Gh_D05G0683 | LOX2.1 | Linoleate 13S-lipoxygenase 2-1, chloroplastic | -2.0313 | -1.8330 | -1.1537 | -1.7303 |
| C_1 | Gh_D05G0753 | MLO6 | MLO-like protein 6 | -0.6332 | -0.7410 | 0.6566 | 0.2171 |
| C_1 | Gh_D05G0754 | MLO3 | MLO-like protein 3 | -1.6818 | -1.7656 | 0.5239 | 0.1144 |
| C_1 | Gh_D05G0809 | At2g27730 | Uncharacterized protein | 0.1824 | 0.9990 | 1.9137 | 1.0528 |
| C_1 | Gh_D05G0854 | STOP1 | Protein SENSITIVE TO PROTON RHIZOTOXICITY 1 | -2.7961 | -2.3983 | -1.0120 | -0.6617 |
| C_1 | Gh_D05G0895 | At2g38010 | Neutral ceramidase | 0.1514 | -0.0161 | 0.6501 | 0.6417 |
| C_1 | Gh_D05G0969 | HERK1 | Receptor-like protein kinase HERK 1 | -1.6388 | 0.6297 | 1.7740 | 2.3311 |
| C_1 | Gh_D05G1165 | NA | Pyrophosphate-energized vacuolar membrane proton | -0.1490 | -0.0049 | 0.5090 | 0.2828 |
|  |  |  | pump |  |  |  |  |
| C_1 | Gh_D05G1206 | HSP26.5 | 26.5 kDa heat shock protein, mitochondrial | -1.5668 | 1.4000 | 0.7558 | 0.5680 |
| C_1 | Gh_D05G1308 | TIM23-1 | Mitochondrial import inner membrane translocase subunit | 0.3008 | 0.2064 | 1.2368 | 0.7102 |
| C_1 | Gh_D05G1347 | GLIP1 | GDSL esterase/lipase 1 | -0.8967 | 0.4782 | 0.5367 | 1.0965 |
| C_1 | Gh_D05G1418 | CYP710A1 | Cytochrome P450 710A1 | -0.0039 | 0.5383 | 0.3965 | 0.4346 |
| C_1 | Gh_D05G1459 | NA | NA | -1.0166 | -0.8441 | 1.1357 | 1.0015 |
| C_1 | Gh_D05G1544 | PBF | Dof zinc finger protein PBF | -0.2916 | 0.4721 | 0.8634 | 0.5608 |
| C_1 | Gh_D05G1585 | MYB39 | Transcription factor MYB39 | -2.6701 | 0.7378 | 1.3077 | 1.3132 |
| C_1 | Gh_D05G1591 | PBP1 | Calcium-binding protein PBP1 | 0.5769 | 0.5977 | 3.1359 | 1.3972 |
| C_1 | Gh_D05G1649 | At1g50720 | Stigma-specific STIG1-like protein 3 | -1.7849 | -2.3796 | 3.3023 | 2.2853 |
| C_1 | Gh_D05G1844 | fadD32 | Long-chain-fatty-acid--AMP ligase | -0.0317 | 0.6899 | 2.6066 | 2.9528 |
| C_1 | Gh_D05G1857 | OFP8 | Transcription repressor OFP8 | -0.0834 | -1.4387 | 1.4048 | 3.5137 |
| C_1 | Gh_D05G1858 | OEP24A | Outer envelope pore protein 24A, chloroplastic | -0.2854 | -0.4221 | 0.0757 | -0.0167 |
| C_1 | Gh_D05G1864 | RBOHD | Respiratory burst oxidase homolog protein D | -2.0179 | -1.6913 | 0.7514 | 0.3579 |
| C_1 | Gh_D05G1894 | CYP82A3 | Cytochrome P450 82A3 | -1.0740 | -0.6119 | -0.2523 | -0.5388 |
| C_1 | Gh_D05G1911 | OSB1 | Protein OSB1, mitochondrial | -0.1644 | 0.2613 | 0.8695 | 0.2925 |
| C_1 | Gh_D05G2068 | NA | Tubulin alpha-4 chain | 0.1347 | 0.4138 | 0.3480 | 0.3713 |
| C_1 | Gh_D05G2073 | TGA21 | TGACG-sequence-specific DNA-binding protein | 0.3157 | -0.4004 | 2.1081 | 1.5122 |
| C_1 | Gh_D05G2085 | OPT4 | Oligopeptide transporter 4 | -4.9835 | -5.0084 | -1.8476 | -1.3869 |
| C_1 | Gh_D05G2178 | STP5 | Sugar transport protein 5 | -0.0235 | 0.3041 | 1.0101 | -0.3583 |
| C_1 | Gh_D05G2252 | BBR | E3 ubiquitin ligase BIG BROTHER-related | -0.9708 | 1.5339 | 2.4819 | 1.4915 |
| C_1 | Gh_D05G2382 | B'BETA | Serine/threonine protein phosphatase 2A 57 kDa | -0.3675 | -0.1590 | 0.6812 | 0.5368 |
|  |  |  | regulatory subunit B' beta isoform |  |  |  |  |
| C_1 | Gh_D05G2506 | CYP81E8 | Cytochrome P450 81E8 | 0.0395 | -0.3369 | 0.6799 | 0.4141 |
| C_1 | Gh_D05G2513 | CSLC12 | Probable xyloglucan glycosyltransferase 12 | 0.2113 | -0.1402 | 1.7401 | 0.7646 |
| C_1 | Gh_D05G2719 | SPP1 | Sucrose-phosphatase 1 | 0.4704 | 0.0009 | 1.1702 | 0.9819 |
| C_1 | Gh_D05G2740 | ALIS3 | ALA-interacting subunit 3 | -0.1157 | -0.2452 | 0.5620 | 0.0829 |
| C_1 | Gh_D05G2746 | ZIFL1 | Protein ZINC INDUCED FACILITATOR-LIKE 1 | -1.4418 | -0.5508 | -0.7566 | 0.1220 |
| C_1 | Gh_D05G2885 | At2g39490 | F-box protein | -1.8655 | -0.4318 | -0.2814 | -0.4003 |
| C_1 | Gh_D05G2935 | PHO1 | Phosphate transporter PHO1 homolog 3 | -0.0248 | -0.4765 | 1.3111 | 0.3413 |
| C_1 | Gh_D05G3106 | RCOM_06994 | UPF0392 protein RCOM_0530710 | -0.4685 | -0.7012 | 0.6109 | 0.6304 |
|  |  | 80 |  |  |  |  |  |
| C_1 | Gh_D05G3234 | CYP71D10 | Cytochrome P450 71D10 | -0.3995 | -0.5840 | 0.0002 | 0.6419 |
| C_1 | Gh_D05G3310 | RPPL1 | Putative disease resistance RPP13-like protein 1 | -3.9334 | -1.7880 | -1.6523 | -0.0838 |
| C_1 | Gh_D05G3435 | NA | NA | -0.9827 | -1.4379 | -0.1242 | 0.6257 |
| C_1 | Gh_D05G3481 | menG | Demethylmenaquinone methyltransferase | -0.0644 | 0.0675 | 1.4783 | 0.8352 |
| C_1 | Gh_D05G3546 | At3g47570 | Probable LRR receptor-like serine/threonine-protein | -2.4118 | -1.9509 | 3.2793 | -0.7695 |
|  |  |  | kinase |  |  |  |  |
| C_1 | Gh_D05G3555 | At4g27190 | Disease resistance protein | -0.8617 | 0.4916 | 5.6096 | -2.6910 |
| C_1 | Gh_D05G3661 | DIR4 | Dirigent protein 4 | -1.0401 | -0.5648 | 0.0653 | -1.1447 |
| C_1 | Gh_D05G3710 | ATJ11 | Chaperone protein dnaJ 11, chloroplastic | 1.1746 | 1.0945 | 1.5130 | 1.1653 |
| C_1 | Gh_D05G3845 | At1g06800 | Phospholipase A1-Igamma1, chloroplastic | -0.8535 | -2.4486 | 3.1316 | 1.6314 |
| C_1 | Gh_D05G3896 | KCS12 | 3-ketoacyl-CoA synthase 12 | -2.6558 | -2.1381 | -0.0568 | -0.6649 |
| C_1 | Gh_D06G0101 | CYP82A3 | Cytochrome P450 82A3 | -0.3418 | -0.2941 | -0.0603 | -0.2327 |
| C_1 | Gh_D06G0114 | At4g27520 | Early nodulin-like protein 2 | -1.1330 | 0.5384 | -0.1566 | 0.5407 |
| C_1 | Gh_D06G0179 | At3g16150 | Probable isoaspartyl peptidase/L-asparaginase 2 | -0.6320 | -0.2189 | 0.7444 | -0.9439 |
| C_1 | Gh_D06G0194 | NA | NA | -0.5039 | -1.0551 | 0.3092 | 0.6607 |
| C_1 | Gh_D06G0219 | Slc38a1 | Sodium-coupled neutral amino acid transporter 1 | 0.3179 | 0.3617 | 1.4013 | 0.4029 |
| C_1 | Gh_D06G0233 | KLHL30 | Kelch-like protein 30 | 0.1001 | 1.4534 | 9.4599 | -0.2525 |
| C_1 | Gh_D06G0237 | KLHL30 | Kelch-like protein 30 | 1.0619 | 1.4534 | 11.6165 | 1.5391 |
| C_1 | Gh_D06G0263 | NA | NA | -0.6377 | -0.5981 | 0.8126 | -0.1722 |
| C_1 | Gh_D06G0300 | FBW2 | F-box protein FBW2 | 0.2671 | 0.9189 | 1.2818 | 1.3049 |
| C_1 | Gh_D06G0690 | MIOX4 | Inositol oxygenase 4 | -1.1285 | -0.4631 | -0.4816 | -0.3824 |
| C_1 | Gh_D06G0741 | IQM1 | IQ domain-containing protein IQM1 | -0.5085 | -1.1609 | 1.8477 | 0.8555 |
| C_1 | Gh_D06G0778 | MOB1-A | MOB kinase activator-like 1A | -0.9752 | 0.4821 | 0.4695 | 0.7629 |
| C_1 | Gh_D06G1180 | DPE2 | 4-alpha-glucanotransferase DPE2 | -0.2965 | -0.6432 | 0.1074 | 0.0740 |
| C_1 | Gh_D06G1235 | NA | NA | -1.0381 | -1.9370 | -0.1506 | 0.8880 |
| C_1 | Gh_D06G1739 | BHLH91 | Transcription factor bHLH91 | -1.2680 | -0.0439 | 2.2874 | 5.4645 |
| C_1 | Gh_D06G1781 | At2g40140 | Zinc finger CCCH domain-containing protein 29 | -0.4450 | -0.1658 | 0.1943 | 1.1814 |
| C_1 | Gh_D06G1939 | WRKY70 | Probable WRKY transcription factor 70 | 0.6014 | 0.4079 | 2.0480 | 1.3375 |
| C_1 | Gh_D06G2030 | At3g16270 | VHS domain-containing protein | 1.3681 | 1.6455 | 2.6989 | 3.2311 |
| C_1 | Gh_D06G2179 | At2g20760 | Clathrin light chain 1 | 0.1245 | 0.0909 | 1.8161 | -0.0740 |
| C_1 | Gh_D06G2204 | NA | NA | -0.6392 | -1.5189 | 0.7332 | 0.4750 |
| C_1 | Gh_D06G2263 | IGS1 | Isoeugenol synthase 1 | -0.1116 | -0.9003 | 1.7766 | 0.1208 |
| C_1 | Gh_D06G2271 | erd-2 | ER lumen protein-retaining receptor | -0.1930 | 0.2891 | 0.4256 | 0.5523 |
| C_1 | Gh_D06G2281 | NA | Thaumatin-like protein 1 | -0.5210 | 0.3457 | 0.6452 | 0.8210 |
| C_1 | Gh_D06G2288 | RAC5 | Rac-like GTP-binding protein 5 | 0.1568 | 0.3198 | 0.5172 | 0.1160 |
| C_1 | Gh_D06G2308 | NA | NA | -0.6360 | -1.1558 | 3.1471 | -0.0913 |
| C_1 | Gh_D06G2309 | NACK2 | Kinesin-like protein NACK2 | -0.2486 | -0.7080 | 6.1815 | -0.2578 |
| C_1 | Gh_D06G2319 | sll0103 | Uncharacterized protein sll0103 | -0.5132 | -0.3353 | 1.4268 | 0.0135 |
| C_1 | Gh_D06G2323 | RGA1 | Putative disease resistance protein RGA1 | -0.2616 | 0.1975 | 0.8398 | -0.0260 |
| C_1 | Gh_D07G0187 | IQM1 | IQ domain-containing protein IQM1 | 0.0023 | -0.2348 | 1.6384 | 1.3781 |
| C_1 | Gh_D07G0203 | PFK3 | ATP-dependent 6-phosphofructokinase 3 | -2.1082 | -0.8489 | 3.7450 | 3.7044 |
| C_1 | Gh_D07G0243 | DDB_G02689 | Putative methyltransferase | -0.9161 | -0.7711 | 0.1273 | 0.8580 |
|  |  | 48 |  |  |  |  |  |
| C_1 | Gh_D07G0263 | GAPN | NADP-dependent glyceraldehyde-3-phosphate | -2.1057 | -1.5395 | 0.0372 | -0.4068 |
|  |  |  | dehydrogenase |  |  |  |  |
| C_1 | Gh_D07G0309 | NA | NA | 0.2123 | 1.5336 | 1.5384 | 1.9004 |
| C_1 | Gh_D07G0317 | WRKY18 | WRKY transcription factor 18 | 0.9107 | 0.9882 | 3.0002 | 0.3534 |
| C_1 | Gh_D07G0333 | EDR2L | Protein ENHANCED DISEASE RESISTANCE 2-like | 0.7535 | 1.1462 | 1.3706 | 1.9024 |
| C_1 | Gh_D07G0374 | IAA29 | Auxin-responsive protein IAA29 | -1.0707 | -0.8138 | 0.0203 | -0.8476 |
| C_1 | Gh_D07G0466 | NA | NA | -1.0730 | -0.9616 | 0.4975 | 0.9265 |
| C_1 | Gh_D07G0491 | SAUR71 | Auxin-responsive protein SAUR71 | -2.1682 | -1.6845 | -0.9304 | -0.5010 |
| C_1 | Gh_D07G0502 | PDR1 | Pleiotropic drug resistance protein 1 | -1.5210 | -1.3054 | -0.4258 | 1.1942 |
| C_1 | Gh_D07G0549 | BT1 | BTB/POZ and TAZ domain-containing protein 1 | 0.1032 | -0.0885 | 0.6998 | 0.3068 |
| C_1 | Gh_D07G0656 | NA | (-)-isopiperitenol/(-)-carveol dehydrogenase, mitochondrial | -0.2661 | -0.6570 | 0.3049 | 0.8331 |
| C_1 | Gh_D07G0692 | UGD5 | UDP-glucose 6-dehydrogenase 5 | 0.5339 | 1.3016 | 1.8541 | 1.1295 |
| C_1 | Gh_D07G0710 | Tnpo3 | Transportin-3 | -0.2465 | -0.0902 | 0.1328 | -0.1134 |
| C_1 | Gh_D07G0804 | nep1 | Aspartic proteinase nepenthesin-1 | -0.7607 | -0.4393 | 0.5980 | 0.5168 |
| C_1 | Gh_D07G0909 | NA | NA | -0.4359 | -0.2469 | 0.8689 | 0.3783 |
| C_1 | Gh_D07G0937 | EXO70A1 | Exocyst complex component EXO70A1 | 0.3929 | 0.2806 | 0.7423 | 1.1223 |
| C_1 | Gh_D07G1010 | CRCK1 | Calmodulin-binding receptor-like cytoplasmic kinase 1 | -0.1318 | 0.2468 | 1.3060 | 1.4043 |
| C_1 | Gh_D07G1041 | VPS50 | Syndetin | -0.4037 | -0.3603 | -0.3495 | -0.2921 |
| C_1 | Gh_D07G1111 | ALMT2 | Aluminum-activated malate transporter 2 | -0.8617 | 0.4916 | 1.8033 | 1.2613 |
| C_1 | Gh_D07G1337 | NA | NA | -2.5452 | -1.6871 | -1.3634 | -0.8195 |
| C_1 | Gh_D07G1436 | SALR | Salutaridine reductase | -1.1348 | 0.4474 | 0.9046 | -0.7651 |
| C_1 | Gh_D07G1690 | NA | NA | 0.8376 | 0.8120 | 1.7880 | 0.4922 |
| C_1 | Gh_D07G1691 | NA | NA | 0.8326 | 0.9852 | 1.2464 | 0.9590 |
| C_1 | Gh_D07G1872 | GT6 | UDP-glucose flavonoid 3-O-glucosyltransferase 6 | 0.1428 | -0.3094 | 0.3378 | 1.0736 |
| C_1 | Gh_D07G2008 | At4g13010 | Putative quinone-oxidoreductase homolog, chloroplastic | -0.1761 | -0.0371 | 0.3173 | -0.3142 |
| C_1 | Gh_D07G2066 | NA | NA | -2.4575 | -4.0058 | 0.3496 | -0.0478 |
| C_1 | Gh_D07G2253 | PCMP-E76 | Pentatricopeptide repeat-containing protein | -0.5852 | -0.3638 | -0.1032 | 0.0239 |
| C_1 | Gh_D07G2328 | WRKY75 | Probable WRKY transcription factor 75 | -2.3838 | -1.4096 | 0.0196 | -0.5846 |
| C_1 | Gh_D07G2341 | NAC083 | NAC domain-containing protein 83 | -0.4148 | 0.1309 | 1.5758 | 1.1986 |
| C_1 | Gh_D07G2371 | BHLH41 | Putative transcription factor bHLH041 | -1.6647 | -0.5758 | 2.4747 | 0.4913 |
| C_1 | Gh_D07G2458 | ZAT9 | Zinc finger protein ZAT9 | 0.6770 | 0.0343 | 2.0646 | 1.6018 |
| C_1 | Gh_D07G2460 | PUB3 | U-box domain-containing protein 3 | -1.5488 | 1.0598 | 0.6285 | 2.1021 |
| C_1 | Gh_D07G2488 | NA | NA | -0.1162 | 0.1668 | 0.6639 | 0.2992 |
| C_1 | Gh_D07G2497 | ERF098 | Ethylene-responsive transcription factor | -1.4246 | -1.2725 | 5.7058 | 3.1059 |
| C_1 | Gh_D08G0207 | RLP12 | Receptor-like protein 12 | -1.8075 | 0.3252 | 2.0408 | 1.5260 |
| C_1 | Gh_D08G0356 | B34 | Histone H3.2 | -0.9073 | -1.1672 | 0.5227 | 0.7138 |
| C_1 | Gh_D08G0442 | GSVIVT0003 | Peroxidase 5 | 0.4851 | 0.0409 | 1.1143 | 1.9851 |
|  |  | 7159001 |  |  |  |  |  |
| C_1 | Gh_D08G0503 | ACA12 | Calcium-transporting ATPase 12, plasma membrane-type | -0.3878 | -0.3359 | 0.0846 | -0.2674 |
| C_1 | Gh_D08G0630 | NA | NA | -3.4133 | -2.0600 | 5.9142 | 3.9929 |
| C_1 | Gh_D08G0716 | NA | NA | -0.3855 | -0.0917 | 1.0353 | 1.0900 |
| C_1 | Gh_D08G0799 | NA | NA | -0.2555 | 0.1580 | 0.2196 | 0.0418 |
| C_1 | Gh_D08G1187 | NA | Myb-related protein Zm38 | -4.2540 | -2.1097 | 0.9366 | 1.5411 |
| C_1 | Gh_D08G1323 | NA | NA | 0.3243 | 0.6128 | 3.5398 | 0.8567 |
| C_1 | Gh_D08G1359 | NA | Peptidyl-prolyl cis-trans isomerase, chloroplastic | -0.0428 | 0.5253 | 0.5298 | 0.5062 |
| C_1 | Gh_D08G1393 | NA | Pathogenesis-related protein 1A | 0.9545 | 1.3116 | 3.6401 | 2.9462 |
| C_1 | Gh_D08G1534 | ERF13 | Ethylene-responsive transcription factor 13 | -0.6335 | -4.0528 | 4.4152 | 3.1562 |
| C_1 | Gh_D08G1585 | NA | NA | -0.2769 | -0.3531 | 1.6744 | 1.0418 |
| C_1 | Gh_D08G1699 | NA | NA | -0.2308 | 0.4078 | 0.9941 | 1.9641 |
| C_1 | Gh_D08G1754 | NA | NA | 0.1215 | 0.9055 | 2.7867 | 2.4334 |
| C_1 | Gh_D08G1810 | aco | 1-aminocyclopropane-1-carboxylate oxidase | -0.3442 | -0.3250 | 0.0616 | -0.0999 |
| C_1 | Gh_D08G1911 | PLDBETA1 | Phospholipase D beta 1 | -0.2203 | -0.8019 | 1.2172 | -0.0888 |
| C_1 | Gh_D08G1949 | AAE3 | Oxalate--CoA ligase | -0.0580 | -0.0748 | 0.6918 | 0.6425 |
| C_1 | Gh_D08G2033 | CYP94A1 | Cytochrome P450 94A1 | -0.6147 | -0.6165 | -0.4234 | -0.1047 |
| C_1 | Gh_D08G2152 | MRH1 | Probable LRR receptor-like serine/threonine-protein | -0.4410 | 0.3811 | 0.0460 | 0.4038 |
|  |  |  | kinase |  |  |  |  |
| C_1 | Gh_D08G2322 | PCO2 | Plant cysteine oxidase 2 | 0.4873 | 1.7233 | 1.4031 | 1.8138 |
| C_1 | Gh_D08G2508 | AGP22 | Arabinogalactan peptide 22 | -1.1651 | 0.4470 | 3.0195 | 2.7313 |
| C_1 | Gh_D08G2557 | ABI2 | Protein phosphatase 2C 77 | -0.1930 | -0.0621 | 1.0869 | 0.3456 |
| C_1 | Gh_D08G2702 | NA | Caffeic acid 3-O-methyltransferase | -0.9072 | -0.6993 | -0.1947 | 1.6547 |
| C_1 | Gh_D09G0001 | CHS | Chalcone synthase | -0.4585 | 0.0681 | 2.1185 | 2.3882 |
| C_1 | Gh_D09G0130 | CML45 | Probable calcium-binding protein CML45 | -1.7599 | -1.7256 | 0.5371 | 0.0171 |
| C_1 | Gh_D09G0155 | WAKL1 | Wall-associated receptor kinase-like 1 | 0.4341 | 0.7075 | 1.6709 | 0.7617 |
| C_1 | Gh_D09G0164 | WAKL9 | Wall-associated receptor kinase-like 9 | -0.0967 | -0.1820 | 0.7389 | -0.2525 |
| C_1 | Gh_D09G0249 | ATL44 | RING-H2 finger protein ATL44 | -0.6296 | -0.1664 | 2.4837 | 3.2485 |
| C_1 | Gh_D09G0393 | PBS1 | Serine/threonine-protein kinase PBS1 | 1.0742 | 1.7086 | 2.1217 | 0.9852 |
| C_1 | Gh_D09G0478 | ATK4 | Kinesin-4 | -0.8427 | -0.5067 | 0.4846 | -0.3946 |
| C_1 | Gh_D09G0496 | GDPDL2 | Glycerophosphodiester phosphodiesterase protein kinase | 0.4503 | -0.0207 | 1.6198 | 0.9795 |
|  |  |  | domain-containing |  |  |  |  |
| C_1 | Gh_D09G0627 | AAE7 | Acetate/butyrate--CoA ligase AAE7, peroxisomal | -0.7028 | 0.6141 | 1.5636 | 3.6898 |
| C_1 | Gh_D09G0648 | MYB108 | Transcription factor MYB108 | -2.7104 | -1.3719 | -0.5095 | 0.4053 |
| C_1 | Gh_D09G0666 | CRK26 | Cysteine-rich receptor-like protein kinase 26 | -0.5556 | 1.2002 | 1.3885 | 0.7429 |
| C_1 | Gh_D09G0676 | CRK25 | Cysteine-rich receptor-like protein kinase 25 | -1.1610 | -0.2949 | 2.4983 | 2.1672 |
| C_1 | Gh_D09G0745 | At1g30090 | F-box/kelch-repeat protein\ | 0.3106 | 0.2378 | 1.6202 | -0.1367 |
| C_1 | Gh_D09G0856 | STOP1 | Protein SENSITIVE TO PROTON RHIZOTOXICITY 1 | -4.5432 | -1.9436 | -0.7047 | 0.6227 |
| C_1 | Gh_D09G0953 | CAF1-9 | Probable CCR4-associated factor 1 homolog 9 | 0.2509 | 0.3605 | 1.3143 | 0.8467 |
| C_1 | Gh_D09G1086 | NA | NA | 0.1482 | 0.0810 | 0.8783 | 0.1483 |
| C_1 | Gh_D09G1122 | CML35 | Probable calcium-binding protein CML35 | 0.1112 | -0.2711 | 0.8029 | 1.1417 |
| C_1 | Gh_D09G1163 | CPK32 | Calcium-dependent protein kinase 32 | -0.6295 | -0.2480 | 0.9192 | -0.1911 |
| C_1 | Gh_D09G1168 | F8H | Probable glucuronoxylan glucuronosyltransferase F8H | -0.7277 | -0.6480 | 0.3648 | -0.0709 |
| C_1 | Gh_D09G1215 | rpsI | 30S ribosomal protein S9 | -0.2712 | -0.2341 | 0.6505 | -0.3640 |
| C_1 | Gh_D09G1275 | YLS9 | Protein YLS9 | 0.1791 | -0.1792 | 1.6124 | 1.3075 |
| C_1 | Gh_D09G1338 | HSFA3 | Heat stress transcription factor A-3 | -0.1536 | 0.6551 | 1.3692 | 0.6811 |
| C_1 | Gh_D09G1343 | At5g03795 | Probable glycosyltransferase | -0.7153 | 0.4159 | 1.4319 | -0.4209 |
| C_1 | Gh_D09G1372 | 4CLL7 | 4-coumarate--CoA ligase-like 7 | -0.4548 | 1.7083 | 3.3321 | 2.3064 |
| C_1 | Gh_D09G1408 | RPL12C | 60S ribosomal protein L12-3 | -0.4926 | 0.0984 | 0.8443 | -0.4803 |
| C_1 | Gh_D09G1418 | UBQ11 | Polyubiquitin 11 | -0.4981 | -0.1499 | 0.7734 | -1.2221 |
| C_1 | Gh_D09G1420 | PER21 | Peroxidase 21 | -0.7562 | 0.5772 | 0.7459 | 0.5642 |
| C_1 | Gh_D09G1436 | GAOA | Galactose oxidase | -0.7847 | 0.0667 | 0.0834 | 0.1377 |
| C_1 | Gh_D09G1450 | RING1 | E3 ubiquitin-protein ligase RING1 | -0.2284 | 0.3077 | 0.3552 | 0.4521 |
| C_1 | Gh_D09G1546 | GPAT6 | Glycerol-3-phosphate 2-O-acyltransferase 6 | -0.8592 | -0.3625 | 0.0545 | -0.5258 |
| C_1 | Gh_D09G1575 | PDC1 | Pyruvate decarboxylase 1 | 0.2728 | 1.0454 | 0.9879 | 0.8883 |
| C_1 | Gh_D09G1590 | NA | Macrophage migration inhibitory factor homolog | -0.2259 | 2.0756 | 1.1527 | 2.0762 |
| C_1 | Gh_D09G1595 | CSA | Probable phospholipid hydroperoxide glutathione | 0.2839 | -0.7516 | 1.3666 | 1.5433 |
|  |  |  | peroxidase |  |  |  |  |
| C_1 | Gh_D09G1750 | ABIL2 | Protein ABIL2 | 0.2395 | -0.0675 | 1.4866 | 0.1829 |
| C_1 | Gh_D09G1766 | YSL3 | Metal-nicotianamine transporter YSL3 | 0.1248 | 0.1098 | 0.7947 | 1.1896 |
| C_1 | Gh_D09G1770 | NA | NA | -0.8843 | 1.0742 | 0.9951 | 1.4935 |
| C_1 | Gh_D09G1791 | C27H6.8 | UPF0160 protein C27H6.8 | -0.9855 | -0.4115 | 0.6075 | 0.2657 |
| C_1 | Gh_D09G1831 | NA | NA | -2.0655 | 0.8584 | 3.3701 | 4.0000 |
| C_1 | Gh_D09G1845 | ARK3 | Armadillo repeat-containing kinesin-like protein 3 | -0.1772 | -0.1876 | -0.1489 | 0.4406 |
| C_1 | Gh_D09G1854 | SAUR36 | Auxin-responsive protein SAUR36 | -0.3925 | 1.3075 | 1.1491 | 1.6773 |
| C_1 | Gh_D09G1867 | ACX1 | Peroxisomal acyl-coenzyme A oxidase 1 | 0.2180 | 0.2112 | 0.4553 | 0.3329 |
| C_1 | Gh_D09G1938 | Gba2 | Non-lysosomal glucosylceramidase | -0.1062 | 0.1043 | 0.2890 | -0.1389 |
| C_1 | Gh_D09G1950 | PME29 | Probable pectinesterase 29 | -2.3700 | 0.5140 | 1.2242 | 2.4373 |
| C_1 | Gh_D09G1967 | Os03g0144800 | Xyloglucan galactosyltransferase KATAMARI1 homolog | -0.7866 | -0.8982 | -0.0014 | 0.4595 |
| C_1 | Gh_D09G2046 | PNC1 | Cationic peroxidase 1 | -1.6970 | -2.3586 | -0.2677 | 0.2687 |
| C_1 | Gh_D09G2047 | PNC1 | Cationic peroxidase 1 | -2.0418 | -1.3296 | 3.0009 | 2.0646 |
| C_1 | Gh_D09G2060 | NA | NA | 0.0496 | 0.3318 | 0.6367 | -0.1038 |
| C_1 | Gh_D09G2258 | CML10 | Probable calcium-binding protein CML10 | -0.3870 | -0.9015 | 0.7220 | -0.0743 |
| C_1 | Gh_D09G2490 | PAP29 | Probable inactive purple acid phosphatase 29 | 1.0619 | 1.4533 | 4.3308 | 1.9593 |
| C_1 | Gh_D09G2492 | RAP2-11 | Ethylene-responsive transcription factor RAP2-11 | -0.3754 | 0.1215 | 1.3420 | 2.0917 |
| C_1 | Gh_D10G0099 | NA | Tropinone reductase-like 3 | -0.6367 | 0.3934 | 0.9478 | 0.6645 |
| C_1 | Gh_D10G0119 | ANP2 | Mitogen-activated protein kinase kinase kinase 2 | 0.1058 | 0.4505 | 1.3724 | 0.4780 |
| C_1 | Gh_D10G0121 | NA | NA | -0.4120 | -0.8713 | 0.9618 | 1.2558 |
| C_1 | Gh_D10G0162 | CRK25 | Cysteine-rich receptor-like protein kinase 25 | -2.4783 | -0.3196 | -0.8120 | 0.1412 |
| C_1 | Gh_D10G0163 | CRK10 | Cysteine-rich receptor-like protein kinase 10 | -2.9973 | -1.9491 | -0.8159 | -0.9124 |
| C_1 | Gh_D10G0236 | LECRK71 | L-type lectin-domain containing receptor kinase VII.1 | -1.2102 | 1.4670 | 1.5868 | 1.2953 |
| C_1 | Gh_D10G0353 | EP1 | Epidermis-specific secreted glycoprotein EP1 | -0.8016 | 0.0937 | 2.2190 | 0.7093 |
| C_1 | Gh_D10G0429 | At1g06800 | Phospholipase A1-Igamma1, chloroplastic | 0.0286 | -0.9147 | 2.4869 | 1.5124 |
| C_1 | Gh_D10G0443 | NPR3 | Regulatory protein NPR3 | 0.0786 | 0.2148 | 0.6582 | 0.0916 |
| C_1 | Gh_D10G0491 | NA | NA | -0.3160 | -1.0451 | 3.7118 | 2.9093 |
| C_1 | Gh_D10G0563 | NA | NA | -0.2414 | 0.6444 | 1.2692 | 0.7194 |
| C_1 | Gh_D10G0582 | DOF1.7 | Dof zinc finger protein DOF1.7 | -2.0941 | -0.5853 | 0.6449 | 1.5931 |
| C_1 | Gh_D10G0591 | DIR1 | Dirigent protein 1 | 0.6849 | 0.8280 | 1.8982 | 0.4953 |
| C_1 | Gh_D10G0606 | At2g19210 | Putative leucine-rich repeat receptor-like protein kinase | 0.5766 | 1.8044 | 2.5081 | 3.1423 |
| C_1 | Gh_D10G0648 | NA | NA | -0.8319 | -0.2282 | 4.3636 | 4.4822 |
| C_1 | Gh_D10G0649 | AAE13 | Malonate--CoA ligase | -0.6276 | -0.2726 | -0.1531 | 0.1142 |
| C_1 | Gh_D10G0722 | M3KE1 | MAP3K epsilon protein kinase 1 | -0.7127 | -0.5882 | -0.2229 | 0.9672 |
| C_1 | Gh_D10G0852 | HIR1 | Hypersensitive-induced response protein 1 | -3.2952 | -1.7370 | -1.3585 | -0.9240 |
| C_1 | Gh_D10G0861 | PIP2-7 | Aquaporin PIP2-7 | -0.6866 | 0.6575 | 1.7759 | 2.6875 |
| C_1 | Gh_D10G0913 | NA | NA | -0.5720 | 0.3361 | 0.7389 | 0.7093 |
| C_1 | Gh_D10G1155 | ASP1 | Aspartate aminotransferase, mitochondrial | -0.1537 | 1.0428 | 4.4050 | 5.0447 |
| C_1 | Gh_D10G1205 | At1g03370 | C2 and GRAM domain-containing protein | 0.0200 | -0.0753 | 0.3579 | 0.3624 |
| C_1 | Gh_D10G1264 | CML5 | Calmodulin-like protein 5 | 0.0459 | 0.9189 | 1.0440 | 1.1630 |
| C_1 | Gh_D10G1351 | RALFL32 | Protein RALF-like 32 | -0.9748 | -0.4367 | 0.3871 | 0.2544 |
| C_1 | Gh_D10G1580 | BHLH63 | Transcription factor bHLH63 | -4.3459 | -2.9926 | 0.6087 | 0.0799 |
| C_1 | Gh_D10G1883 | NA | NA | -0.0736 | 1.2481 | 4.1067 | 3.5497 |
| C_1 | Gh_D10G1885 | NA | NA | -0.2756 | 0.9317 | 2.0120 | 0.3316 |
| C_1 | Gh_D10G1956 | At4g08850 | Probable LRR receptor-like serine/threonine-protein | -0.2356 | 1.1141 | 1.2576 | 0.8588 |
|  |  |  | kinase |  |  |  |  |
| C_1 | Gh_D10G2032 | SDR1 | (+)-neomenthol dehydrogenase | -1.0302 | -0.4802 | -0.2175 | 0.1936 |
| C_1 | Gh_D10G2084 | NA | NA | -0.5587 | 0.0101 | 0.5896 | -0.4251 |
| C_1 | Gh_D10G2238 | NA | NA | -3.5580 | -3.7063 | -0.5184 | 0.0004 |
| C_1 | Gh_D10G2262 | At4g27190 | Disease resistance protein | -0.5206 | 0.0754 | 4.3990 | 0.1420 |
| C_1 | Gh_D10G2353 | CSA1 | Disease resistance-like protein CSA1 | -1.5324 | -0.5893 | -0.5993 | -0.5254 |
| C_1 | Gh_D10G2430 | ACA11 | Putative calcium-transporting ATPase 11, plasma | 0.6448 | 0.9138 | 2.1678 | 0.6066 |
|  |  |  | membrane-type |  |  |  |  |
| C_1 | Gh_D10G2444 | CCR3 | Putative serine/threonine-protein kinase-like protein CCR3 | -0.5531 | -0.7917 | 1.4332 | 1.1123 |
| C_1 | Gh_D10G2488 | NA | NA | -0.2840 | -0.9576 | 1.4745 | 0.5418 |
| C_1 | Gh_D10G2519 | NA | NA | -1.2094 | -3.3975 | 1.7989 | 1.0443 |
| C_1 | Gh_D10G2524 | GATA8 | GATA transcription factor 8 | 0.3547 | 1.1335 | 1.2591 | 1.3998 |
| C_1 | Gh_D10G2604 | ABCB19 | ABC transporter B family member 19 | -0.9517 | -2.0989 | -0.4746 | 0.8766 |
| C_1 | Gh_D11G0006 | BOR1 | Boron transporter 1 | -0.1254 | -1.0590 | 2.7608 | 2.1690 |
| C_1 | Gh_D11G0150 | MFSD5 | Molybdate-anion transporter | 0.1545 | 0.1968 | 0.5600 | 0.4955 |
| C_1 | Gh_D11G0224 | At5g41590 | Protein LURP-one-related 17 | 0.4940 | 0.5831 | 1.3925 | 1.0959 |
| C_1 | Gh_D11G0241 | OXA1 | Mitochondrial inner membrane protein OXA1 | 0.2763 | 0.3738 | 1.2148 | -0.0171 |
| C_1 | Gh_D11G0272 | BI-1 | Bax inhibitor 1 | -0.6681 | -0.5563 | 0.1386 | -0.5336 |
| C_1 | Gh_D11G0434 | At1g67623 | Putative F-box protein | -1.6619 | 1.6817 | 2.6056 | 2.1178 |
| C_1 | Gh_D11G0484 | NA | NA | 0.6744 | 1.5716 | 1.6687 | 1.4606 |
| C_1 | Gh_D11G0496 | HSF24 | Heat shock factor protein HSF24 | -0.0394 | -0.4736 | 1.1407 | 0.7950 |
| C_1 | Gh_D11G0523 | VQ29 | VQ motif-containing protein 29 | -1.2945 | -0.4836 | 0.4148 | 0.1213 |
| C_1 | Gh_D11G0723 | P4H7 | Probable prolyl 4-hydroxylase 7 | -0.0375 | -1.0765 | 3.5782 | 0.8918 |
| C_1 | Gh_D11G0741 | CML45 | Probable calcium-binding protein CML45 | -1.8286 | -2.9973 | 0.8674 | 0.2502 |
| C_1 | Gh_D11G0790 | At2g44680 | Putative casein kinase II subunit beta-4 | -0.7082 | -0.0727 | 0.1760 | -0.3751 |
| C_1 | Gh_D11G0799 | NA | NA | -0.3034 | -0.5978 | 0.2219 | 1.4678 |
| C_1 | Gh_D11G0846 | PECS-2.1 | Pectinesterase 2 | 0.0956 | -0.5548 | 1.5177 | 3.5569 |
| C_1 | Gh_D11G0858 | NA | NA | -2.4301 | -3.2385 | 1.2031 | 0.5065 |
| C_1 | Gh_D11G0910 | BAP2 | BON1-associated protein 2 | -1.8103 | -2.0214 | 0.4111 | 0.7739 |
| C_1 | Gh_D11G0914 | DGD2 | Digalactosyldiacylglycerol synthase 2, chloroplastic | -0.0216 | -0.2596 | 0.3879 | 0.2819 |
| C_1 | Gh_D11G0993 | Exosc4 | Exosome complex component RRP41 | -0.6836 | 0.2246 | 0.4139 | -0.3474 |
| C_1 | Gh_D11G1011 | WRKY46 | Probable WRKY transcription factor 46 | -0.4384 | -0.1074 | 1.2074 | 1.5599 |
| C_1 | Gh_D11G1094 | ABCB21 | ABC transporter B family member 21 | -0.2433 | -0.2827 | 0.1564 | 1.0798 |
| C_1 | Gh_D11G1126 | At1g09390 | GDSL esterase/lipase | -0.6678 | 1.0741 | 0.7405 | 1.2438 |
| C_1 | Gh_D11G1180 | EXL2 | Protein EXORDIUM-like 2 | -1.4940 | 0.9883 | 4.2275 | 4.5674 |
| C_1 | Gh_D11G1237 | BRN1 | RNA-binding protein BRN1 | 0.2136 | 0.5302 | 0.7488 | 0.4890 |
| C_1 | Gh_D11G1586 | CPX1 | Coproporphyrinogen-III oxidase 1, chloroplastic | 0.2566 | 0.9179 | 1.4431 | 0.9644 |
| C_1 | Gh_D11G1607 | NA | NA | 0.8377 | 0.8309 | 1.5948 | 1.3829 |
| C_1 | Gh_D11G1622 | ANX2 | Receptor-like protein kinase ANXUR2 | 0.0243 | -0.5502 | 1.3645 | 0.6078 |
| C_1 | Gh_D11G1685 | At1g67000 | Probable receptor-like protein kinase | -0.2266 | -0.7115 | 1.1813 | 0.8300 |
| C_1 | Gh_D11G1703 | RGA4 | Putative disease resistance protein RGA4 | 0.0282 | 0.0472 | 0.9113 | 0.5711 |
| C_1 | Gh_D11G1718 | TMN4 | Transmembrane 9 superfamily member 4 | -0.0834 | -0.1308 | 1.0421 | -0.4423 |
| C_1 | Gh_D11G1740 | At1g07870 | Probable serine/threonine-protein kinase RLCKVII | -0.6208 | 0.6597 | 0.5921 | 1.2069 |
| C_1 | Gh_D11G1774 | CPK28 | Calcium-dependent protein kinase 28 | 0.2892 | 0.2827 | 0.6466 | 0.5713 |
| C_1 | Gh_D11G1821 | EPHX2 | Bifunctional epoxide hydrolase 2 | -0.5929 | -1.5554 | 0.7646 | 0.5986 |
| C_1 | Gh_D11G1886 | GATL2 | Probable galacturonosyltransferase-like 2 | -0.0234 | -0.3292 | 0.0325 | 2.1775 |
| C_1 | Gh_D11G1887 | CML41 | Probable calcium-binding protein CML41 | -0.9738 | -2.1000 | -0.2324 | 0.6339 |
| C_1 | Gh_D11G1937 | YDA | Mitogen-activated protein kinase kinase kinase YODA | 0.4484 | 0.3784 | 2.8271 | 2.2129 |
| C_1 | Gh_D11G1983 | At5g24010 | Probable receptor-like protein kinase | -0.8139 | 0.5731 | 0.0668 | 0.4190 |
| C_1 | Gh_D11G2001 | mvd | Diphosphomevalonate decarboxylase | -0.4381 | -0.0335 | 2.0964 | 1.6908 |
| C_1 | Gh_D11G2005 | P4H9 | Probable prolyl 4-hydroxylase 9 | -0.1057 | 0.9586 | 4.2025 | 3.0898 |
| C_1 | Gh_D11G2074 | BHLH25 | Transcription factor bHLH25 | -0.1309 | -0.7201 | 3.1614 | 1.2738 |
| C_1 | Gh_D11G2120 | DDB_G02890 | IST1-like protein | 0.4282 | 0.0310 | 2.4479 | 1.7938 |
|  |  | 29 |  |  |  |  |  |
| C_1 | Gh_D11G2121 | PUMP5 | Mitochondrial uncoupling protein 5 | 0.2259 | 0.2189 | 0.7052 | 0.4937 |
| C_1 | Gh_D11G2432 | NA | NA | 0.8881 | -0.4461 | 3.9450 | 3.9369 |
| C_1 | Gh_D11G2522 | IPUT1 | Inositol phosphorylceramide glucuronosyltransferase 1 | -0.6287 | -0.5800 | 0.0493 | 0.0354 |
| C_1 | Gh_D11G2523 | RGA3 | Putative disease resistance protein RGA3 | -0.4991 | -0.9422 | 0.0740 | 0.1707 |
| C_1 | Gh_D11G2571 | MYOB1 | Myosin-binding protein 1 | -0.1065 | 0.2103 | 0.4758 | 0.1776 |
| C_1 | Gh_D11G2574 | NA | NA | -1.2780 | -2.6311 | 0.9130 | 2.0950 |
| C_1 | Gh_D11G2772 | PP2B15 | F-box protein PP2-B15 | 0.2640 | 0.8591 | 1.5512 | 1.4348 |
| C_1 | Gh_D11G2792 | At3g01520 | Universal stress protein A-like protein | -0.2783 | 1.1712 | 1.0989 | 1.2676 |
| C_1 | Gh_D11G2935 | NA | NA | -0.7388 | -0.2601 | 0.3994 | -0.2500 |
| C_1 | Gh_D11G3077 | NA | NA | -2.5411 | -1.3264 | -0.1806 | 0.1339 |
| C_1 | Gh_D11G3111 | NA | NA | -0.4370 | -0.4966 | 1.5209 | 1.1134 |
| C_1 | Gh_D11G3135 | NA | NA | 0.0475 | 1.3047 | 1.3336 | 1.6245 |
| C_1 | Gh_D11G3369 | N | TMV resistance protein N | -0.3382 | -0.1281 | 0.9425 | 0.5738 |
| C_1 | Gh_D11G3383 | GSO1 | LRR receptor-like serine/threonine-protein kinase GSO1 | -0.9886 | -2.5449 | 1.4757 | 2.3217 |
| C_1 | Gh_D11G3401 | RFL1 | Disease resistance protein RFL1 | -0.2481 | 0.4380 | 0.5513 | 0.7056 |
| C_1 | Gh_D11G3471 | P4H7 | Probable prolyl 4-hydroxylase 7 | 0.4025 | -1.0086 | 6.2386 | -0.2525 |
| C_1 | Gh_D11G3477 | NA | NA | 0.0520 | 0.4833 | 0.5515 | 0.2526 |
| C_1 | Gh_D11G3494 | RGA1 | Putative disease resistance protein RGA1 | -0.0708 | -0.3973 | 0.9283 | 0.6113 |
| C_1 | Gh_D12G0136 | FAM126A | Hyccin | -0.2328 | -0.5422 | 1.6260 | 0.5390 |
| C_1 | Gh_D12G0179 | ATL47 | RING-H2 finger protein ATL47 | 0.2698 | -0.2668 | 2.2908 | 1.3552 |
| C_1 | Gh_D12G0269 | SFH8 | Phosphatidylinositol/phosphatidylcholine transfer protein | -0.4290 | -0.5691 | 0.0110 | 0.0040 |
|  |  |  | SFH8 |  |  |  |  |
| C_1 | Gh_D12G0472 | ctps | CTP synthase | 0.5566 | 1.0128 | 1.3905 | 1.0156 |
| C_1 | Gh_D12G0508 | ntpR | Protein NtpR | -0.5738 | 0.5377 | 0.7584 | 0.1316 |
| C_1 | Gh_D12G0510 | NA | NA | -0.5029 | -0.6717 | 0.6830 | 0.4328 |
| C_1 | Gh_D12G0534 | CYP81D1 | Cytochrome P450 81D1 | -1.3860 | -2.0944 | 1.3238 | 0.1950 |
| C_1 | Gh_D12G0658 | ERF114 | Ethylene-responsive transcription factor | -0.0270 | 1.3471 | 2.1894 | 2.1881 |
| C_1 | Gh_D12G0806 | XTHB | Xyloglucan endotransglucosylase/hydrolase protein B | 1.9463 | 2.3836 | 2.6315 | 2.7956 |
| C_1 | Gh_D12G0934 | SCL6 | Scarecrow-like protein 6 | -0.6566 | -0.5841 | -0.1844 | 0.1278 |
| C_1 | Gh_D12G0960 | ERF2 | Ethylene-responsive transcription factor 2 | 0.3436 | -1.5634 | 3.6761 | 2.9753 |
| C_1 | Gh_D12G1014 | NA | NA | 0.3804 | -0.9292 | 1.9403 | 3.0524 |
| C_1 | Gh_D12G1050 | BAP2 | BON1-associated protein 2 | -0.7577 | -0.6735 | 1.6821 | 1.4219 |
| C_1 | Gh_D12G1143 | KIC | Calcium-binding protein KIC | -0.3828 | -0.9496 | 0.1445 | 0.7759 |
| C_1 | Gh_D12G1163 | KCS1 | 3-ketoacyl-CoA synthase 1 | -0.9182 | -0.6239 | 0.7185 | -0.4553 |
| C_1 | Gh_D12G1297 | PME7 | Probable pectinesterase/pectinesterase inhibitor 7 | 0.2172 | -0.0323 | 1.4292 | 0.6778 |
| C_1 | Gh_D12G1557 | Esyt3 | Extended synaptotagmin-3 | 0.2215 | 1.1644 | 1.4180 | 1.0036 |
| C_1 | Gh_D12G1599 | AFP2 | Ninja-family protein AFP2 | -0.3902 | -1.0283 | 0.8455 | 0.4181 |
| C_1 | Gh_D12G1818 | COB | Protein COBRA | 0.0227 | 0.4645 | 0.5984 | 0.2038 |
| C_1 | Gh_D12G1955 | At5g07610 | F-box protein | -1.7014 | -2.2997 | 0.5262 | 0.6498 |
| C_1 | Gh_D12G1978 | TPPA | Trehalose-phosphate phosphatase A | 0.1754 | 0.2035 | 1.4767 | 0.6848 |
| C_1 | Gh_D12G2074 | MYC2 | Transcription factor MYC2 | -0.1872 | -0.6133 | 0.3939 | 0.3534 |
| C_1 | Gh_D12G2137 | At4g17486 | DeSI-like protein | -0.5688 | 0.2338 | 0.2052 | 0.0193 |
| C_1 | Gh_D12G2236 | ERF025 | Ethylene-responsive transcription factor | 1.6479 | 1.9737 | 2.9045 | 2.1874 |
| C_1 | Gh_D12G2348 | CYP78A3 | Cytochrome P450 78A3 | 0.0816 | 0.5489 | 0.6053 | 0.8388 |
| C_1 | Gh_D12G2376 | NA | NA | -0.6187 | -0.7445 | 0.4313 | 0.0631 |
| C_1 | Gh_D12G2552 | CML27 | Probable calcium-binding protein CML27 | 1.1434 | 1.6827 | 2.5606 | 1.4915 |
| C_1 | Gh_D12G2608 | SRO5 | Probable inactive poly [ADP-ribose] polymerase SRO5 | 0.5414 | 1.0322 | 2.5960 | 2.0437 |
| C_1 | Gh_D12G2645 | At1g18440 | Peptidyl-tRNA hydrolase, chloroplastic | 0.1554 | 0.1366 | 1.2022 | 0.4754 |
| C_1 | Gh_D12G2772 | PHO1 | Phosphate transporter PHO1 homolog 3 | -0.4918 | -0.5207 | 3.2828 | 1.0802 |
| C_1 | Gh_D13G0052 | NA | NA | -1.3883 | -0.2560 | 0.9865 | 0.4971 |
| C_1 | Gh_D13G0152 | At5g39865 | Uncharacterized protein | 0.4506 | 1.3584 | 1.3697 | 1.7282 |
| C_1 | Gh_D13G0202 | NA | Actin | -0.1036 | -0.2055 | 1.5741 | 5.1468 |
| C_1 | Gh_D13G0241 | NA | NA | -0.0834 | -0.5845 | 1.3052 | 0.2598 |
| C_1 | Gh_D13G0245 | NA | NA | -0.1076 | -2.4367 | 6.3585 | 3.7158 |
| C_1 | Gh_D13G0263 | asd | Aspartate-semialdehyde dehydrogenase | 0.2740 | 0.9244 | 0.9178 | 0.8064 |
| C_1 | Gh_D13G0393 | LAG2 | LAG1 longevity assurance homolog 2 | -0.2277 | -0.0197 | 1.3291 | 1.6022 |
| C_1 | Gh_D13G0451 | ZAT10 | Zinc finger protein ZAT10 | -0.0384 | -0.4839 | 0.8792 | 0.5061 |
| C_1 | Gh_D13G0472 | MADS27 | MADS-box transcription factor 27 | -0.2154 | -0.4246 | 2.3797 | 2.1076 |
| C_1 | Gh_D13G0493 | NA | NA | 0.0847 | 0.3812 | 1.3650 | 1.0620 |
| C_1 | Gh_D13G0494 | NA | NA | -0.5484 | 0.0067 | 0.6436 | 0.2184 |
| C_1 | Gh_D13G0607 | PXC3 | Leucine-rich repeat receptor-like tyrosine-protein kinase | 0.5941 | 1.2427 | 3.7745 | 3.8266 |
| C_1 | Gh_D13G0900 | PUB42 | Putative U-box domain-containing protein 42 | -1.0140 | -0.9686 | -0.2880 | -0.1872 |
| C_1 | Gh_D13G1458 | NIP1 | NEP1-interacting protein 1 | -1.5097 | -0.4644 | 0.8916 | 0.6138 |
| C_1 | Gh_D13G1502 | CIGR1 | Chitin-inducible gibberellin-responsive protein 1 | -0.7255 | -0.3125 | 0.3541 | -0.2249 |
| C_1 | Gh_D13G1816 | NA | Pathogenesis-related protein PR-4B | 0.8389 | 0.2306 | 0.9561 | 1.7668 |
| C_1 | Gh_D13G2023 | CKX7 | Cytokinin dehydrogenase 7 | -0.7500 | -0.2767 | 0.6201 | 1.9134 |
| C_1 | Gh_D13G2056 | XI-K | Myosin-17 | -0.3678 | -0.2575 | 0.8329 | -0.8139 |
| C_1 | Gh_D13G2143 | TMN11 | Transmembrane 9 superfamily member 11 | 0.1126 | 0.0530 | 0.4074 | 0.0898 |
| C_1 | Gh_D13G2160 | AOP1 | Probable 2-oxoglutarate-dependent dioxygenase AOP1 | -0.2645 | -1.4825 | 0.6343 | 2.0919 |
| C_1 | Gh_D13G2161 | AOP1 | Probable 2-oxoglutarate-dependent dioxygenase AOP1 | 0.2931 | -0.6389 | 1.3963 | 3.6518 |
| C_1 | Gh_D13G2207 | VRN1 | B3 domain-containing transcription factor VRN1 | -0.7074 | -0.5631 | -0.2201 | -0.3232 |
| C_1 | Gh_D13G2258 | fp-1 | Foot protein 1 variant 2 | 0.5657 | 0.3447 | 2.2940 | 0.4665 |
| C_1 | Gh_D13G2374 | FDM4 | Factor of DNA methylation 4 | 0.1377 | 0.4311 | 1.0311 | 0.0753 |
| C_1 | Gh_D13G2392 | ZAT12 | Zinc finger protein ZAT12 | -0.1943 | -0.0826 | 2.1441 | 1.7355 |
| C_1 | Gh_D13G2431 | 4CLL7 | 4-coumarate--CoA ligase-like 7 | -0.3108 | -0.1527 | 0.2358 | -0.0395 |
| C_1 | Gh_D13G2470 | Ttc1 | Tetratricopeptide repeat protein 1 | -0.0118 | 0.8944 | 0.7538 | 0.5808 |
| C_1 | Gh_D13G2472 | NA | Pyrophosphate-energized vacuolar membrane proton | -0.0275 | 0.3236 | 0.2165 | 0.4636 |
|  |  |  | pump |  |  |  |  |
| C_1 | Gh_Sca004827G01 | At4g27220 | Probable disease resistance protein | -0.8617 | 0.4916 | 1.8699 | 0.6902 |
| C_1 | Gh_Sca004932G05 | NA | NA | -0.2319 | 1.2063 | 0.6409 | 1.0664 |
| C_1 | Gh_Sca004958G01 | NA | 21 kDa protein | 0.2044 | 0.0600 | 0.7228 | 0.6386 |
| C_1 | Gh_Sca004965G01 | UPL1 | E3 ubiquitin-protein ligase UPL1 | 0.0477 | -0.6829 | 1.9310 | 0.8602 |
| C_1 | Gh_Sca005047G02 | AP4M | AP-4 complex subunit mu | -0.1465 | 0.3029 | 1.2383 | -0.7160 |
| C_1 | Gh_Sca005681G01 | SKP1B | SKP1-like protein 1B | 0.0805 | 0.0741 | 0.3480 | 0.3076 |
| C_1 | Gh_Sca005978G01 | WRKY33 | Probable WRKY transcription factor 33 | -0.7697 | -0.9667 | 0.9458 | 0.7436 |
| C_1 | Gh_Sca006111G01 | PEX5 | Peroxisome biogenesis protein 5 | -0.3315 | -0.1584 | 2.6743 | 0.9839 |
| C_1 | Gh_Sca008280G01 | SEOB | Protein SIEVE ELEMENT OCCLUSION B | -2.7582 | 1.6239 | 0.7389 | 2.7110 |
| C_1 | Gh_Sca009205G01 | At2g48040/ | Piezo-type mechanosensitive ion channel homolog | -4.3477 | -2.9945 | 5.1769 | 2.5645 |
| C_1 | Gh_Sca011512G01 | NA | NA | -0.9842 | 0.0169 | 0.9067 | 0.1002 |
| C_1 | Gh_Sca014110G01 | EXLB1 | Expansin-like B1 | -2.2567 | -1.3267 | 0.0961 | 0.1977 |
| C_1 | Gh_Sca014485G01 | PNC1 | Cationic peroxidase 1 | -1.8942 | -2.5737 | -0.1295 | 0.6554 |
| C_1 | Gh_Sca016688G01 | DIR4 | Dirigent protein 4 | 0.2334 | 0.8804 | 5.1412 | 0.7093 |
| C_1 | Gh_Sca018101G01 | At4g27190 | Disease resistance protein | -0.2425 | -0.1726 | 0.8648 | 0.8221 |
| C_1 | Gh_Sca027192G01 | At1g61190 | Probable disease resistance protein | -1.1451 | 0.1520 | 2.3943 | -0.4857 |
| C_2 | Gh_A01G0013 | ANAC094 | Putative NAC domain-containing protein 94 | 0.4414 | -1.3451 | 2.7591 | 1.3522 |
| C_2 | Gh_A01G0064 | Os01g0234100 | B3 domain-containing protein | 0.2692 | -0.3134 | -0.1230 | 0.0224 |
| C_2 | Gh_A01G0086 | P4H9 | Probable prolyl 4-hydroxylase 9 | 0.6377 | 0.3068 | 0.6833 | 0.3356 |
| C_2 | Gh_A01G0102 | PLT5 | Polyol transporter 5 | 0.8102 | -2.2323 | -0.1880 | -0.2463 |
| C_2 | Gh_A01G0235 | At5g49610 | F-box protein | -0.0924 | -0.6452 | 0.9070 | 0.0777 |
| C_2 | Gh_A01G0246 | WAG2 | Serine/threonine-protein kinase WAG2 | 1.0946 | -0.0495 | 0.4149 | -0.7642 |
| C_2 | Gh_A01G0270 | F6'H1 | Feruloyl CoA ortho-hydroxylase 1 | 0.6964 | -2.4132 | 2.5997 | 1.3402 |
| C_2 | Gh_A01G0293 | NA | Cytochrome P450 CYP72A219 | -0.9655 | -1.3907 | -1.0081 | -1.8266 |
| C_2 | Gh_A01G0346 | DIR15 | Dirigent protein 15 | 0.7914 | -1.8361 | -1.5801 | -0.4151 |
| C_2 | Gh_A01G0372 | NA | Pathogen-related protein | -0.1141 | -0.9555 | 0.1277 | -0.8495 |
| C_2 | Gh_A01G0490 | NCS2 | S-norcoclaurine synthase 2 | -0.0064 | -1.4386 | 1.9903 | 0.4112 |
| C_2 | Gh_A01G0547 | GH3.6 | Indole-3-acetic acid-amido synthetase GH3.6 | 0.1651 | -1.3603 | -1.1695 | -0.1440 |
| C_2 | Gh_A01G0581 | THI1 | Thiamine thiazole synthase, chloroplastic | 0.4514 | -0.2015 | 0.9283 | 0.5331 |
| C_2 | Gh_A01G0639 | WRKY6 | WRKY transcription factor 6 | -0.0641 | -0.7572 | 0.3711 | 0.2727 |
| C_2 | Gh_A01G0705 | ZIP11 | Zinc transporter 11 | 1.1860 | -0.4619 | 1.2281 | 0.6925 |
| C_2 | Gh_A01G0715 | CYP86A1 | Cytochrome P450 86A1 | 0.6946 | -0.2378 | -0.2197 | -0.5929 |
| C_2 | Gh_A01G0716 | NUDT10 | Nudix hydrolase 10 | 0.2090 | -1.0766 | 1.1222 | 0.0848 |
| C_2 | Gh_A01G0760 | XERICO | Probable E3 ubiquitin-protein ligase | 0.1405 | -1.7462 | 1.4963 | 1.3021 |
| C_2 | Gh_A01G0769 | SAUR72 | Auxin-responsive protein SAUR72 | 0.3680 | -2.0691 | 1.6747 | -1.2186 |
| C_2 | Gh_A01G0779 | NA | NA | -0.9750 | -2.1484 | -1.3650 | -0.1714 |
| C_2 | Gh_A01G0780 | NA | NA | 0.6654 | -3.7545 | -0.7018 | -1.8946 |
| C_2 | Gh_A01G0891 | PERK11 | Putative proline-rich receptor-like protein kinase | 0.3114 | -0.4904 | 0.8278 | -0.4028 |
| C_2 | Gh_A01G1117 | SYP121 | Syntaxin-121 | -0.1905 | -1.1274 | 1.0406 | 0.0150 |
| C_2 | Gh_A01G1184 | PAP3 | Purple acid phosphatase 3 | 0.2968 | -0.8083 | -0.6619 | -0.3692 |
| C_2 | Gh_A01G1185 | At1g25240 | Putative clathrin assembly protein | -0.8144 | -2.4762 | -1.9192 | 0.1922 |
| C_2 | Gh_A01G1369 | DIR4 | Dirigent protein 4 | 1.3202 | 0.4379 | 4.0683 | 1.1135 |
| C_2 | Gh_A01G1456 | At1g04910 | Uncharacterized protein | -0.0428 | -2.2589 | -1.1642 | -0.9707 |
| C_2 | Gh_A01G1559 | PUR2 | Phosphoribosylamine--glycine ligase, chloroplastic | 0.0575 | -0.4769 | -0.0369 | -0.4427 |
| C_2 | Gh_A01G1737 | NA | Secoisolariciresinol dehydrogenase (Fragment) | 0.4550 | -0.9957 | 0.0905 | -0.1385 |
| C_2 | Gh_A01G1785 | FBA | Fructose-bisphosphate aldolase cytoplasmic isozyme | 0.5941 | -1.2517 | 0.9267 | 0.1010 |
| C_2 | Gh_A01G1846 | NA | NA | 0.2404 | -0.7605 | -0.4630 | -0.0224 |
| C_2 | Gh_A01G1898 | RE | Protein RETICULATA, chloroplastic | 0.1329 | -0.1996 | 0.2241 | 0.2182 |
| C_2 | Gh_A01G1913 | MT1 | Metallothionein-like protein type 2 | 0.6844 | -0.0196 | 0.9734 | -0.0034 |
| C_2 | Gh_A01G1929 | NA | NA | -0.0062 | -0.8708 | 1.5550 | 0.3566 |
| C_2 | Gh_A01G1950 | SQD2 | Sulfoquinovosyl transferase SQD2 | 0.3369 | -0.6170 | 0.0989 | 0.3908 |
| C_2 | Gh_A01G1974 | At1g53430 | Probable LRR receptor-like serine/threonine-protein | 1.0196 | -0.1796 | 1.8975 | -0.1608 |
|  |  |  | kinase |  |  |  |  |
| C_2 | Gh_A01G2081 | PHF1 | SEC12-like protein 1 | 0.4026 | -0.6337 | 0.9324 | 0.1262 |
| C_2 | Gh_A02G0017 | At1g06840 | Probable LRR receptor-like serine/threonine-protein | 0.4220 | -0.4928 | 0.0149 | -0.4283 |
|  |  |  | kinase |  |  |  |  |
| C_2 | Gh_A02G0088 | NA | NA | -0.4463 | -2.1715 | 0.9617 | -0.0567 |
| C_2 | Gh_A02G0135 | EPSIN2 | Clathrin interactor EPSIN 2 | 0.3475 | -0.1146 | 0.2660 | -0.0543 |
| C_2 | Gh_A02G0170 | CBDAS2 | Cannabidiolic acid synthase-like 1 | 0.1540 | -1.3382 | -0.5197 | -0.8132 |
| C_2 | Gh_A02G0194 | RLP12 | Receptor-like protein 12 | 0.5139 | -0.7316 | 1.5756 | 0.1770 |
| C_2 | Gh_A02G0212 | PCMP-H64 | Pentatricopeptide repeat-containing protein | 0.1131 | -0.0353 | 0.6263 | -0.5007 |
| C_2 | Gh_A02G0222 | NA | Cytochrome P450 CYP749A22 | 0.3566 | -1.1809 | -0.3955 | -0.8200 |
| C_2 | Gh_A02G0260 | NA | Glutathione transferase GST 23 | 1.0309 | -2.0437 | 2.0411 | 0.4437 |
| C_2 | Gh_A02G0339 | CIPK7 | CBL-interacting serine/threonine-protein kinase 7 | 0.5244 | -0.3833 | 0.0569 | -1.5354 |
| C_2 | Gh_A02G0380 | PRP | Repetitive proline-rich cell wall protein | 0.5332 | -1.0174 | 0.0809 | 1.0603 |
| C_2 | Gh_A02G0500 | NA | Major allergen Pru ar 1 | 0.1829 | -0.9734 | 0.3155 | -0.1711 |
| C_2 | Gh_A02G0524 | NA | NA | -0.0567 | -1.1116 | -0.0735 | -0.1944 |
| C_2 | Gh_A02G0578 | SAMS | S-adenosylmethionine synthase | 0.5121 | 0.0355 | 0.3164 | -0.6196 |
| C_2 | Gh_A02G0615 | MLO1 | MLO-like protein 1 | 0.4907 | -0.9211 | 0.5139 | 0.2357 |
| C_2 | Gh_A02G0617 | STOP1 | Protein SENSITIVE TO PROTON RHIZOTOXICITY 1 | 0.2573 | -0.2565 | -0.0143 | 0.2106 |
| C_2 | Gh_A02G0636 | PDCD4 | Programmed cell death protein 4 | 0.4486 | -0.1132 | -0.0635 | -0.4593 |
| C_2 | Gh_A02G0655 | ERF114 | Ethylene-responsive transcription factor ERF114 | -0.1125 | -1.6224 | 0.8429 | 1.4349 |
| C_2 | Gh_A02G0657 | RPL3 | 60S ribosomal protein L3 | 0.2846 | -0.7094 | -0.4184 | 0.2797 |
| C_2 | Gh_A02G0665 | VIP1 | Transcription factor VIP1 | 0.3691 | -0.1614 | 0.2112 | -0.2466 |
| C_2 | Gh_A02G0741 | At1g77330 | 1-aminocyclopropane-1-carboxylate oxidase 5 | 0.4966 | -1.9036 | -1.4721 | -1.5190 |
| C_2 | Gh_A02G0821 | ROG1 | Putative lipase ROG1 | -0.0018 | -0.9548 | 0.8906 | 0.0294 |
| C_2 | Gh_A02G0984 | PI4KG2 | Phosphatidylinositol 4-kinase gamma 2 | -0.0694 | -0.6323 | 0.5057 | 0.3507 |
| C_2 | Gh_A02G1051 | CRT3 | Calreticulin-3 | -0.2609 | -0.7028 | -0.5273 | -0.1590 |
| C_2 | Gh_A02G1074 | NA | NA | 0.0771 | -1.0172 | 0.7032 | 0.2162 |
| C_2 | Gh_A02G1167 | KINESIN-13A | Kinesin-13A | -0.3112 | -1.1875 | 0.6568 | -0.1984 |
| C_2 | Gh_A02G1185 | GLYK | D-glycerate 3-kinase, chloroplastic | 0.0069 | -0.3308 | 0.1361 | -0.2531 |
| C_2 | Gh_A02G1215 | TIF3A1 | Eukaryotic translation initiation factor 3 subunit A | -0.5496 | -2.9212 | -0.4164 | -0.7808 |
| C_2 | Gh_A02G1275 | Acin1 | Apoptotic chromatin condensation inducer in the nucleus | 0.1079 | 0.0328 | 0.1814 | -0.0354 |
| C_2 | Gh_A02G1316 | NA | NA | 1.0361 | -0.4217 | 1.6300 | 1.4169 |
| C_2 | Gh_A02G1319 | ZED1 | Non-functional pseudokinase ZED1 | 0.2623 | -0.9598 | 0.6042 | -0.1749 |
| C_2 | Gh_A02G1421 | COX6A | Cytochrome c oxidase subunit 6a, mitochondrial | 0.3210 | -0.0571 | 0.1669 | 0.0926 |
| C_2 | Gh_A02G1424 | At5g47360 | Pentatricopeptide repeat-containing protein | 0.3699 | -1.4653 | 0.4415 | -0.0778 |
| C_2 | Gh_A02G1442 | PSK3 | Phytosulfokines 3 | 0.8775 | -1.7648 | 0.3239 | -0.7235 |
| C_2 | Gh_A02G1495 | AP2 | Floral homeotic protein APETALA 2 | -0.3412 | -0.7356 | -0.5648 | -0.5842 |
| C_2 | Gh_A02G1524 | NA | NA | 0.0621 | -1.9810 | 1.8755 | 1.1610 |
| C_2 | Gh_A02G1546 | BLH4 | BEL1-like homeodomain protein 4 | 0.6056 | -1.5546 | 0.1180 | -0.6531 |
| C_2 | Gh_A02G1576 | SPCC23B6.04 | CRAL-TRIO domain-containing protein C23B6.04c | 0.2499 | -0.8184 | 0.5240 | -0.1580 |
| C_2 | Gh_A02G1590 | PUB5 | U-box domain-containing protein 5 | 0.2194 | 0.0114 | 0.6362 | 0.1908 |
| C_2 | Gh_A02G1609 | RPP8L3 | Disease resistance RPP8-like protein 3 | 0.5072 | -2.3054 | 0.6418 | 0.3539 |
| C_2 | Gh_A02G1629 | At3g50940 | AAA-ATPase | 0.0084 | -0.5391 | 0.7626 | -0.1808 |
| C_2 | Gh_A02G1635 | CPK28 | Calcium-dependent protein kinase 28 | 0.4900 | -0.0935 | 1.0721 | 0.6287 |
| C_2 | Gh_A02G1689 | AAE1 | Probable acyl-activating enzyme 1, peroxisomal | -0.0918 | -2.3615 | 0.4719 | -0.6303 |
| C_2 | Gh_A02G1727 | NA | NA | -0.3137 | -1.7074 | -0.1975 | -0.6119 |
| C_2 | Gh_A02G1728 | NA | NA | -0.0151 | -0.4846 | 0.0854 | 0.2767 |
| C_2 | Gh_A02G1751 | ATL21A | Putative RING-H2 finger protein ATL21A | 0.4622 | -2.1106 | 0.2943 | -0.9606 |
| C_2 | Gh_A02G1820 | FLACCA | Molybdenum cofactor sulfurase | 0.3294 | -0.0517 | 0.0708 | -0.2745 |
| C_2 | Gh_A03G0005 | NA | NA | 0.4153 | -0.9353 | 1.2724 | -0.0611 |
| C_2 | Gh_A03G0081 | PSD1 | Phosphatidylserine decarboxylase proenzyme 1, | 0.0857 | -0.4227 | 0.4243 | -0.4647 |
|  |  |  | mitochondrial |  |  |  |  |
| C_2 | Gh_A03G0225 | NA | Probable phytol kinase 3, chloroplastic | 0.0659 | -1.2860 | -0.2339 | -0.3381 |
| C_2 | Gh_A03G0247 | GLY | Lactoylglutathione lyase | 0.0654 | -1.3544 | 1.4188 | -1.7610 |
| C_2 | Gh_A03G0317 | RPL35 | 60S ribosomal protein L35 | 0.1063 | -0.7330 | 0.2191 | 0.2460 |
| C_2 | Gh_A03G0391 | NA | NA | 0.0778 | -1.7356 | -1.0314 | -0.4216 |
| C_2 | Gh_A03G0427 | GONST2 | GDP-mannose transporter GONST2 | -0.0951 | -1.1430 | 0.0565 | -0.0676 |
| C_2 | Gh_A03G0493 | NBR1 | Protein NBR1 homolog | 0.0753 | -0.3083 | -0.3435 | -0.2285 |
| C_2 | Gh_A03G0563 | SOBIR1 | Leucine-rich repeat receptor-like | 0.1329 | -0.2243 | 0.2289 | 0.2901 |
|  |  |  | serine/threonine/tyrosine-protein kinase |  |  |  |  |
| C_2 | Gh_A03G0628 | Os08g0135800 | Zinc finger CCCH domain-containing protein 55 | 0.0859 | -0.7337 | 0.6285 | -0.6296 |
| C_2 | Gh_A03G0639 | UGT89B2 | UDP-glycosyltransferase 89B2 | 0.5500 | -0.2881 | 0.2751 | 0.8269 |
| C_2 | Gh_A03G0701 | KCS4 | 3-ketoacyl-CoA synthase 4 | 0.0302 | -1.0109 | 0.7292 | -0.0640 |
| C_2 | Gh_A03G0797 | RPL19B | 60S ribosomal protein L19-2 | 0.6331 | -1.9765 | -0.4817 | -1.1473 |
| C_2 | Gh_A03G0837 | NA | NA | 0.7951 | -1.4751 | 2.5949 | 1.7253 |
| C_2 | Gh_A03G0959 | SYP131 | Putative syntaxin-131 | -0.0261 | -0.7153 | -0.4315 | 0.1213 |
| C_2 | Gh_A03G1147 | At1g67750 | Probable pectate lyase 5 | 1.5104 | -0.4553 | 0.4116 | 0.3990 |
| C_2 | Gh_A03G1323 | DTX12 | Protein DETOXIFICATION 12 | 0.1089 | -1.4618 | 0.3244 | 0.1492 |
| C_2 | Gh_A03G1472 | OXP1 | 5-oxoprolinase | 0.2469 | -0.2001 | -0.2140 | 0.0476 |
| C_2 | Gh_A03G1477 | NA | NA | -0.3765 | -1.0679 | -0.8538 | -0.9660 |
| C_2 | Gh_A03G1496 | HMG2 | 3-hydroxy-3-methylglutaryl-coenzyme A reductase 2 | 0.2677 | -0.8451 | 0.7470 | -0.1990 |
| C_2 | Gh_A03G1517 | PNC2 | Cationic peroxidase 2 | 1.1133 | -0.3234 | 0.3485 | 0.1633 |
| C_2 | Gh_A03G1628 | GH3.1 | Probable indole-3-acetic acid-amido synthetase GH3.1 | 0.6750 | -0.2672 | 1.7996 | 0.5332 |
| C_2 | Gh_A03G1677 | CXE7 | Probable carboxylesterase 7 | 0.0393 | -1.0082 | -0.6545 | 0.2897 |
| C_2 | Gh_A03G1880 | NA | NA | 0.8205 | 0.4806 | 1.2104 | -0.0782 |
| C_2 | Gh_A03G1989 | YLS3 | Protein YLS3 | 0.1248 | -1.2591 | 0.9238 | -0.1569 |
| C_2 | Gh_A03G2111 | UGD5 | UDP-glucose 6-dehydrogenase 5 | 0.8854 | 0.6022 | 1.2731 | 0.2976 |
| C_2 | Gh_A03G2147 | 7OMT | (R,S)-reticuline 7-O-methyltransferase | -0.0239 | -2.1302 | 0.9283 | 0.2565 |
| C_2 | Gh_A04G0070 | DIR4 | Dirigent protein 4 | 0.3943 | -2.4215 | 0.4066 | -0.4360 |
| C_2 | Gh_A04G0073 | DIR4 | Dirigent protein 4 | -0.4003 | -3.4064 | -0.2932 | -1.1084 |
| C_2 | Gh_A04G0098 | SYP51 | Syntaxin-51 | 0.6103 | -1.1378 | 1.2847 | -4.2796 |
| C_2 | Gh_A04G0204 | SD18 | Receptor-like serine/threonine-protein kinase SD1-8 | -0.0598 | -1.0916 | 0.6654 | -0.2179 |
| C_2 | Gh_A04G0205 | B120 | G-type lectin S-receptor-like serine/threonine-protein | -0.4401 | -3.7911 | 0.1077 | -0.1758 |
|  |  |  | kinase B120 |  |  |  |  |
| C_2 | Gh_A04G0223 | NA | NA | -0.0068 | -1.6515 | -0.1654 | 1.4155 |
| C_2 | Gh_A04G0261 | NA | NA | 0.7300 | -1.6079 | 0.4148 | 0.9156 |
| C_2 | Gh_A04G0283 | PSKR2 | Phytosulfokine receptor 2 | -0.1308 | -1.0784 | -0.1192 | 0.0433 |
| C_2 | Gh_A04G0354 | NA | NA | 0.1938 | -1.7428 | -0.6401 | -0.9557 |
| C_2 | Gh_A04G0571 | NA | NA | 0.2101 | -1.4478 | 0.7655 | 0.6301 |
| C_2 | Gh_A04G0579 | DIOX2 | Probable 2-oxoglutarate/Fe(II)-dependent dioxygenase | 1.1268 | -0.3570 | -0.2813 | -1.1616 |
| C_2 | Gh_A04G0581 | UGT73D1 | UDP-glycosyltransferase 73D1 | 0.3619 | -1.4034 | 0.4393 | -0.1279 |
| C_2 | Gh_A04G0683 | IP5P8 | Type I inositol polyphosphate 5-phosphatase 8 | 0.5019 | -1.3844 | -0.1221 | -0.9948 |
| C_2 | Gh_A04G0686 | PPA4 | Soluble inorganic pyrophosphatase 4 | 0.6912 | -0.6444 | 0.5776 | 0.1595 |
| C_2 | Gh_A04G0743 | LAC7 | Laccase-7 | 0.7002 | -1.3821 | 0.4338 | 0.1070 |
| C_2 | Gh_A04G0750 | SSUH2 | Protein SSUH2 homolog | 0.2977 | -0.4318 | -0.0104 | 0.0022 |
| C_2 | Gh_A04G0761 | SQD2 | Sulfoquinovosyl transferase SQD2 | 0.6528 | -0.5937 | -0.0382 | -0.1026 |
| C_2 | Gh_A04G0829 | GSTU17 | Glutathione S-transferase U17 | -0.2230 | -1.9380 | -0.0665 | -0.0794 |
| C_2 | Gh_A04G0978 | TFT6 | 14-3-3 protein 6 | 0.1851 | -0.0442 | 0.1522 | -0.1066 |
| C_2 | Gh_A04G0991 | LECRK91 | L-type lectin-domain containing receptor kinase IX.1 | -0.9364 | -1.7925 | -0.2966 | -0.4903 |
| C_2 | Gh_A04G1037 | CNR1 | Cell number regulator 1 | 0.7327 | -1.4640 | 0.8496 | -0.0348 |
| C_2 | Gh_A04G1107 | CYP82C4 | Cytochrome P450 82C4 | 0.2989 | -2.7527 | 2.1211 | 1.5986 |
| C_2 | Gh_A05G0052 | Os02g0799000 | Probable protein phosphatase 2C 27 | -0.1641 | -1.8970 | 1.0389 | 0.1001 |
| C_2 | Gh_A05G0067 | MGD2 | Monogalactosyldiacylglycerol synthase 2, chloroplastic | -0.0324 | -0.7449 | -0.6547 | -0.1755 |
| C_2 | Gh_A05G0092 | SPX1 | SPX domain-containing protein 1 | 0.4473 | -1.2803 | -0.8160 | -0.5491 |
| C_2 | Gh_A05G0189 | At5g56590 | Glucan endo-1,3-beta-glucosidase 13 | 0.4279 | -2.2989 | 2.7706 | -0.5591 |
| C_2 | Gh_A05G0214 | SLC25A16 | Graves disease carrier protein | -0.3682 | -1.7770 | -0.6506 | -1.1793 |
| C_2 | Gh_A05G0404 | CHIA | Acidic mammalian chitinase | 2.0485 | -1.1288 | -0.1054 | -3.2918 |
| C_2 | Gh_A05G0437 | RDR6 | RNA-dependent RNA polymerase 6 | 0.3462 | -0.3597 | 0.9349 | 0.3310 |
| C_2 | Gh_A05G0496 | At5g10820 | Probable folate-biopterin transporter 6 | -0.1883 | -0.4917 | 0.3600 | -0.4577 |
| C_2 | Gh_A05G0537 | adck1 | Uncharacterized aarF domain-containing protein kinase 1 | -0.2447 | -0.6953 | 0.1555 | -0.2631 |
| C_2 | Gh_A05G0553 | fadD28 | Long-chain-fatty-acid--AMP ligase FadD28 | 1.6400 | -1.5124 | -0.8926 | -0.6519 |
| C_2 | Gh_A05G0616 | At2g32560 | F-box protein At2g32560 | 0.7238 | 0.2015 | 0.4400 | -0.1448 |
| C_2 | Gh_A05G0666 | SEC23 | Protein transport protein SEC23 | 2.7827 | -0.5207 | 0.0829 | -0.6169 |
| C_2 | Gh_A05G0756 | BACOVA_02659 | Beta-glucosidase BoGH3B | 1.0720 | -0.9199 | 0.9314 | 0.4171 |
| C_2 | Gh_A05G0813 | TRM32 | Protein TRM32 | -0.4456 | -1.1112 | 0.9404 | -0.4966 |
| C_2 | Gh_A05G0832 | Harbi1 | Putative nuclease HARBI1 | -0.4424 | -0.7444 | -0.6044 | -0.1990 |
| C_2 | Gh_A05G0910 | ACA7 | Alpha carbonic anhydrase 7 | 0.0233 | -2.9027 | 0.8677 | 0.1657 |
| C_2 | Gh_A05G0926 | IRX7 | Probable glucuronoxylan glucuronosyltransferase IRX7 | 0.8867 | -0.2408 | 0.5596 | 2.0054 |
| C_2 | Gh_A05G0954 | MMT1 | Methionine S-methyltransferase | 0.0834 | -0.2459 | 0.1469 | 0.2852 |
| C_2 | Gh_A05G1356 | At5g49770 | Probable leucine-rich repeat receptor-like protein kinase | 0.0899 | -0.8984 | -0.4627 | -0.1943 |
| C_2 | Gh_A05G1374 | RMA1H1 | E3 ubiquitin-protein ligase RMA1H1 | 0.5400 | -0.0779 | 0.0441 | -0.1593 |
| C_2 | Gh_A05G1474 | At1g50720 | Stigma-specific STIG1-like protein 3 | -0.4517 | -4.7554 | -0.2229 | -0.2526 |
| C_2 | Gh_A05G1489 | At1g16860 | Uncharacterized membrane protein | -0.5038 | -0.9994 | -0.2890 | -0.0239 |
| C_2 | Gh_A05G1507 | SCL13 | Scarecrow-like protein 13 | 0.6222 | -1.1123 | 1.0035 | -0.5654 |
| C_2 | Gh_A05G1579 | CAD6 | Probable cinnamyl alcohol dehydrogenase 6 | 0.6850 | -1.2612 | 0.9133 | -0.2177 |
| C_2 | Gh_A05G1740 | GUX2 | UDP-glucuronate:xylan alpha-glucuronosyltransferase 2 | 0.4224 | 0.0018 | 0.5073 | 0.8419 |
| C_2 | Gh_A05G1787 | PK1 | Putative receptor protein kinase ZmPK1 | -0.5324 | -1.9064 | -0.6068 | -1.0116 |
| C_2 | Gh_A05G1814 | LBD39 | LOB domain-containing protein 39 | 0.8073 | -0.1496 | 0.8088 | 0.3869 |
| C_2 | Gh_A05G1906 | ATL54 | RING-H2 finger protein | 0.5308 | -0.8930 | -0.6602 | 0.0538 |
| C_2 | Gh_A05G1994 | NA | NA | 0.2421 | -1.3887 | 1.6780 | 1.5707 |
| C_2 | Gh_A05G2036 | NA | NA | -0.0485 | -0.4303 | -0.0367 | -0.0626 |
| C_2 | Gh_A05G2290 | NA | NA | -0.4876 | -1.2141 | 0.2542 | -0.0827 |
| C_2 | Gh_A05G2344 | YLS9 | Protein YLS9 | 0.1106 | -1.4149 | 1.8034 | 0.3111 |
| C_2 | Gh_A05G2378 | scy1 | Probable inactive serine/threonine-protein kinase scy1 | 0.2476 | -0.1715 | 0.6056 | 0.1216 |
| C_2 | Gh_A05G2468 | ALIS3 | ALA-interacting subunit 3 | -0.0395 | -0.5623 | 0.5826 | -0.1608 |
| C_2 | Gh_A05G2582 | ACD6 | Protein ACCELERATED CELL DEATH 6 | 1.1542 | -1.3332 | -1.1358 | -0.6476 |
| C_2 | Gh_A05G2615 | UGT76C4 | UDP-glycosyltransferase 76C4 | 0.1317 | -0.8290 | -0.3386 | 0.2080 |
| C_2 | Gh_A05G2705 | UTR4 | UDP-galactose/UDP-glucose transporter 4 | -0.8397 | -0.8649 | -0.3025 | -1.2939 |
| C_2 | Gh_A05G2734 | NA | NA | 0.4823 | -1.9496 | -1.1139 | -0.4641 |
| C_2 | Gh_A05G2786 | At5g40240 | WAT1-related protein | -0.2578 | -1.3187 | -0.2747 | -0.0928 |
| C_2 | Gh_A05G2793 | 2MMP | Metalloendoproteinase 2-MMP | 0.1545 | -1.2859 | 1.1114 | 0.2994 |
| C_2 | Gh_A05G2986 | NA | NA | 0.8914 | -1.2470 | 1.1352 | -0.2755 |
| C_2 | Gh_A05G3047 | AC58 | Actin-58 | 0.4340 | 0.2816 | 0.4975 | 0.3532 |
| C_2 | Gh_A05G3075 | ABCG16 | ABC transporter G family member 16 | 0.3697 | -1.9707 | 3.0302 | 0.7030 |
| C_2 | Gh_A05G3078 | NA | NA | 0.3996 | -2.2479 | 1.1049 | -0.5848 |
| C_2 | Gh_A05G3154 | pvaA | Polyvinylalcohol dehydrogenase | -1.6916 | -3.6444 | -0.3547 | -1.8695 |
| C_2 | Gh_A05G3327 | NA | NA | 0.5232 | -0.7838 | 0.2670 | -0.4413 |
| C_2 | Gh_A05G3343 | RLK1 | G-type lectin S-receptor-like serine/threonine-protein | 1.0271 | -0.5258 | 2.8331 | 1.0410 |
|  |  |  | kinase RLK1 |  |  |  |  |
| C_2 | Gh_A05G3408 | NA | NA | 0.7170 | -0.0690 | 0.0054 | -0.4235 |
| C_2 | Gh_A05G3591 | At5g39020 | Probable receptor-like protein kinase | 1.2632 | 0.3622 | 1.2244 | 1.5680 |
| C_2 | Gh_A05G3621 | NA | NA | 0.2929 | -0.1437 | -0.0323 | 0.0124 |
| C_2 | Gh_A05G3760 | PGM | Phosphoglycerate mutase-like protein | 0.1357 | -0.3747 | 0.2160 | 0.2399 |
| C_2 | Gh_A05G3775 | AZG1 | Adenine/guanine permease AZG1 | 0.3530 | -0.7698 | -0.4128 | -0.9759 |
| C_2 | Gh_A05G3820 | At1g17710 | Inorganic pyrophosphatase 2 | 0.9398 | -1.6871 | 0.1585 | -0.8888 |
| C_2 | Gh_A05G3831 | PDR1 | Pleiotropic drug resistance protein 1 | 0.0205 | -0.1229 | 0.5956 | -0.3880 |
| C_2 | Gh_A05G3958 | SPAC17H9.04 | Uncharacterized RNA-binding protein | 0.1585 | -2.0773 | -0.7649 | 0.3211 |
| C_2 | Gh_A05G4009 | PMA4 | Plasma membrane ATPase 4 | 0.4288 | 0.1568 | 0.3409 | 0.2321 |
| C_2 | Gh_A06G0053 | FPP4 | Filament-like plant protein 4 | 0.0189 | -0.1694 | 0.0721 | 0.0181 |
| C_2 | Gh_A06G0083 | rsc5 | Random slug protein 5 | 0.0807 | -1.8213 | 0.1707 | -0.7764 |
| C_2 | Gh_A06G0119 | CYP82A4 | Cytochrome P450 82A4 | 0.1106 | -0.2402 | 0.7409 | -0.2768 |
| C_2 | Gh_A06G0167 | At3g50280 | Uncharacterized acetyltransferase | 0.4463 | -2.6829 | 0.5364 | -0.9893 |
| C_2 | Gh_A06G0667 | PAL | Phenylalanine ammonia-lyase | 0.5584 | -2.1599 | -0.1914 | -0.4726 |
| C_2 | Gh_A06G0687 | At3g45310 | Thiol protease aleurain-like | 0.0627 | -0.4728 | -0.3008 | 0.2287 |
| C_2 | Gh_A06G0709 | ERF017 | Ethylene-responsive transcription factor | -0.2112 | -1.5682 | 0.1440 | 1.3156 |
| C_2 | Gh_A06G0762 | At2g01680 | Ankyrin repeat-containing protein | -1.6461 | -2.9553 | -1.5833 | -0.9498 |
| C_2 | Gh_A06G0764 | WRKY50 | Probable WRKY transcription factor 50 | 0.7956 | -0.9332 | 1.6958 | 1.3264 |
| C_2 | Gh_A06G0944 | GDU1 | Protein GLUTAMINE DUMPER 1 | 0.9776 | -0.3036 | 0.0761 | -0.8015 |
| C_2 | Gh_A06G1070 | CRK30 | Putative cysteine-rich receptor-like protein kinase 30 | -0.0931 | -1.6643 | 2.3629 | 0.5201 |
| C_2 | Gh_A06G1075 | NA | NA | -1.7503 | -3.9817 | -0.2897 | -0.1632 |
| C_2 | Gh_A06G1084 | At4g18930 | Cyclic phosphodiesterase | 0.5907 | -0.5598 | 0.5044 | -0.1118 |
| C_2 | Gh_A06G1091 | NDA1 | Internal alternative NAD(P)H-ubiquinone oxidoreductase | 0.3832 | -1.1058 | 0.9469 | 0.3281 |
|  |  |  | A1, mitochondrial |  |  |  |  |
| C_2 | Gh_A06G1092 | NA | NA | 0.8511 | 0.1772 | 1.2659 | 1.2259 |
| C_2 | Gh_A06G1095 | SCAMP1 | Secretory carrier-associated membrane protein 1 | -0.2941 | -0.4105 | -0.0261 | -0.3882 |
| C_2 | Gh_A06G1166 | NA | NA | 0.5118 | -0.0213 | 0.2310 | -0.1243 |
| C_2 | Gh_A06G1462 | NA | Cucumisin | 0.9186 | -1.7638 | -0.0633 | -0.2074 |
| C_2 | Gh_A06G1491 | OFP13 | Transcription repressor OFP13 | 0.8103 | -2.8468 | 0.0696 | 0.0557 |
| C_2 | Gh_A06G1600 | At4g27290 | G-type lectin S-receptor-like serine/threonine-protein kinase | -0.0316 | -4.0247 | -4.9479 | -4.9775 |
| C_2 | Gh_A06G1633 | At4g14610 | Probable disease resistance protein | 0.3036 | -0.6435 | 0.4587 | -0.0048 |
| C_2 | Gh_A06G1714 | IGS1 | Isoeugenol synthase 1 | 0.7661 | -1.8243 | -0.0962 | -0.7094 |
| C_2 | Gh_A06G1730 | PUB33 | U-box domain-containing protein 33 | 0.8132 | -0.2569 | -0.3983 | -0.4401 |
| C_2 | Gh_A06G1774 | NA | NA | -0.3058 | -2.9619 | 0.9224 | -0.3570 |
| C_2 | Gh_A06G1801 | NSF | Vesicle-fusing ATPase | 0.2530 | -0.2969 | 0.3493 | -0.1089 |
| C_2 | Gh_A06G1871 | At5g64460 | Phosphoglycerate mutase-like protein 1 | 1.1455 | -2.3593 | 3.6619 | 0.7876 |
| C_2 | Gh_A06G2011 | GA2OX1 | Gibberellin 2-beta-dioxygenase | 1.1795 | -2.1693 | -1.3791 | -2.4259 |
| C_2 | Gh_A06G2100 | GLPK | Glycerol kinase | -0.0337 | -0.3230 | -0.1425 | -0.0440 |
| C_2 | Gh_A07G0005 | rsmH | Ribosomal RNA small subunit methyltransferase H | -0.1625 | -1.9628 | -0.2988 | -0.1394 |
| C_2 | Gh_A07G0188 | DDB_G02689 | Putative methyltransferase | 0.2019 | -0.7297 | 0.6486 | 0.1003 |
|  |  | 48 |  |  |  |  |  |
| C_2 | Gh_A07G0189 | DDB_G0268948 | Putative methyltransferase | -0.0289 | -1.4371 | 0.5912 | 0.2675 |
| C_2 | Gh_A07G0194 | CHIT1 | Chitotriosidase-1 | -1.4078 | -2.0322 | -1.1379 | -1.3072 |
| C_2 | Gh_A07G0363 | TDR | Leucine-rich repeat receptor-like protein kinase TDR | -0.0244 | -0.8887 | -0.7124 | 0.1990 |
| C_2 | Gh_A07G0395 | CLPD | Chaperone protein ClpD, chloroplastic | 0.6061 | -0.3322 | 0.2715 | 0.3589 |
| C_2 | Gh_A07G0397 | NA | NA | 0.4437 | -2.1539 | -0.0569 | -1.5057 |
| C_2 | Gh_A07G0413 | MPT2 | Mitochondrial phosphate carrier protein 2, mitochondrial | -0.4113 | -2.6667 | -0.3097 | -0.0339 |
| C_2 | Gh_A07G0439 | PDR1 | Pleiotropic drug resistance protein 1 | 0.1612 | -1.6924 | 1.0050 | 1.1333 |
| C_2 | Gh_A07G0494 | NRT3.1 | High-affinity nitrate transporter 3.1 | -0.2022 | -0.5897 | -0.3584 | 0.3008 |
| C_2 | Gh_A07G0602 | POPTR_0012s | L-Ala-D/L-amino acid epimerase | -0.2063 | -1.7077 | -0.3730 | -0.3491 |
|  |  | 05040g |  |  |  |  |  |
| C_2 | Gh_A07G0606 | OsABCB25 | ABC transporter B family member 25 | 0.2927 | -0.4701 | 0.1357 | 0.3611 |
| C_2 | Gh_A07G0607 | CSTF50 | Cleavage stimulation factor subunit 50 | -0.6796 | -1.2662 | -0.3609 | -0.5842 |
| C_2 | Gh_A07G0714 | CYP81D1 | Cytochrome P450 81D1 | 0.2609 | -1.1846 | -0.3122 | -0.0529 |
| C_2 | Gh_A07G0950 | PMR5 | Protein PMR5 | -0.4407 | -1.8857 | -0.5672 | -0.4014 |
| C_2 | Gh_A07G0999 | CER1 | Protein ECERIFERUM 1 | -0.1887 | -0.3933 | -0.2004 | -0.1998 |
| C_2 | Gh_A07G1000 | CER1 | Protein ECERIFERUM 1 | 0.6735 | -1.0722 | -0.3194 | -0.1841 |
| C_2 | Gh_A07G1075 | CIPK3 | CBL-interacting serine/threonine-protein kinase 3 | 0.3415 | -0.3038 | 0.4662 | -0.1171 |
| C_2 | Gh_A07G1173 | COX5C | Cytochrome c oxidase subunit 5C | 0.4846 | 0.0076 | 0.0417 | -0.3895 |
| C_2 | Gh_A07G1190 | SDR3b | Short-chain dehydrogenase reductase 3b | 0.3116 | -1.8428 | -0.3827 | -0.5070 |
| C_2 | Gh_A07G1199 | LTI6B | Hydrophobic protein LTI6B | 1.1221 | -0.0721 | 0.3164 | -0.3579 |
| C_2 | Gh_A07G1312 | PUB33 | U-box domain-containing protein 33 | 0.1571 | -0.1853 | 0.2527 | 0.3954 |
| C_2 | Gh_A07G1421 | RPL23A | 60S ribosomal protein L23 | -0.0358 | -0.7211 | 0.7125 | 0.2494 |
| C_2 | Gh_A07G1489 | NA | NA | 0.4838 | -1.6511 | 0.0218 | -1.6292 |
| C_2 | Gh_A07G1529 | At5g35735 | Cytochrome b561 and DOMON domain-containing | 0.5642 | -0.5632 | 1.8211 | 0.5081 |
|  |  |  | protein |  |  |  |  |
| C_2 | Gh_A07G1532 | NA | NA | 1.0350 | 0.7525 | 1.6742 | 1.1106 |
| C_2 | Gh_A07G1743 | NUDT10 | Nudix hydrolase 10 | 1.1151 | -0.7024 | 2.3834 | 0.2519 |
| C_2 | Gh_A07G1771 | RNS2 | Ribonuclease 2 | 0.2098 | -0.2558 | 0.0829 | -0.2759 |
| C_2 | Gh_A07G1787 | NA | NA | -0.4530 | -1.1825 | -0.8994 | -0.3471 |
| C_2 | Gh_A07G1798 | OXI1 | Serine/threonine-protein kinase OXI1 | -0.1769 | -1.4117 | 0.8848 | 0.4089 |
| C_2 | Gh_A07G1804 | At4g13010 | Putative quinone-oxidoreductase homolog, chloroplastic | -0.4529 | -1.4837 | 1.1362 | -0.7686 |
| C_2 | Gh_A07G1857 | NA | NA | -1.7603 | -4.0191 | 0.4428 | -0.5213 |
| C_2 | Gh_A07G1858 | NA | NA | -0.1917 | -3.9615 | 0.8896 | -0.0324 |
| C_2 | Gh_A07G1902 | ELC | Protein ELC | 0.6544 | -2.9136 | 1.3122 | 0.1203 |
| C_2 | Gh_A07G1937 | PHT1-7 | Probable inorganic phosphate transporter 1-7 | 0.6817 | -1.7934 | 1.2470 | -0.1682 |
| C_2 | Gh_A07G1945 | NA | NA | -0.3019 | -1.2766 | -0.7399 | -0.7842 |
| C_2 | Gh_A07G1987 | ABCB15 | ABC transporter B family member 15 | 0.6254 | -0.9028 | -0.4228 | -0.7166 |
| C_2 | Gh_A07G1988 | ABCB15 | ABC transporter B family member 15 | 0.8387 | -1.1260 | -0.3958 | -1.0854 |
| C_2 | Gh_A07G1998 | mcd4 | GPI ethanolamine phosphate transferase 1 | 0.5163 | -0.3728 | 1.0212 | 0.0994 |
| C_2 | Gh_A07G2022 | AO | L-aspartate oxidase, chloroplastic | 0.2346 | -0.5874 | 0.6841 | 0.4338 |
| C_2 | Gh_A07G2265 | NA | NA | 0.5105 | -1.7580 | -0.1975 | -0.3062 |
| C_2 | Gh_A07G2303 | CSE | Caffeoylshikimate esterase | -0.4012 | -1.2126 | 0.7757 | -0.1726 |
| C_2 | Gh_A08G0045 | HIRA | Protein HIRA | 0.1635 | -0.1334 | 0.0117 | -0.1521 |
| C_2 | Gh_A08G0071 | RHF2A | E3 ubiquitin-protein ligase RHF2A | 0.4548 | -0.2359 | 0.4574 | 0.1119 |
| C_2 | Gh_A08G0167 | COQ5 | 2-methoxy-6-polyprenyl-1,4-benzoquinol methylase, | -0.4818 | -0.6580 | -0.2667 | -0.3824 |
|  |  |  | mitochondrial |  |  |  |  |
| C_2 | Gh_A08G0459 | MPC3 | Mitochondrial pyruvate carrier 3 | 0.4912 | -1.6097 | -0.7580 | -1.2172 |
| C_2 | Gh_A08G0510 | ASAT1 | Acyl-CoA--sterol O-acyltransferase 1 | 0.2581 | -2.4593 | 0.1790 | -0.1221 |
| C_2 | Gh_A08G0651 | ROPGAP2 | Rho GTPase-activating protein 2 | -0.1315 | -1.2787 | 0.5006 | 0.4805 |
| C_2 | Gh_A08G0713 | zgc:55781 | UPF0415 protein C7orf25 homolog | 0.3887 | -3.3648 | 0.0350 | 1.4689 |
| C_2 | Gh_A08G0714 | pod | Peroxidase 15 | 0.7539 | -0.6106 | 0.4468 | 0.7591 |
| C_2 | Gh_A08G0960 | At1g64710 | Alcohol dehydrogenase-like 4 | 0.7206 | -1.1900 | 1.6154 | 0.3398 |
| C_2 | Gh_A08G0979 | At1g64065 | Late embryogenesis abundant protein | -0.1433 | -2.6727 | 2.5293 | 1.3051 |
| C_2 | Gh_A08G1012 | YUC10 | Probable indole-3-pyruvate monooxygenase YUCCA10 | 0.4393 | -0.7750 | -0.4265 | -0.0650 |
| C_2 | Gh_A08G1098 | CYP78A6 | Cytochrome P450 78A6 | -0.0430 | -0.3806 | 1.6565 | -0.7305 |
| C_2 | Gh_A08G1168 | QKY | Protein QUIRKY | 0.4913 | -0.1627 | 0.9049 | 0.7282 |
| C_2 | Gh_A08G1402 | MYB39 | Transcription factor MYB39 | -0.0247 | -2.2979 | 0.8010 | -0.0179 |
| C_2 | Gh_A08G1484 | GATA26 | GATA transcription factor 26 | -0.1218 | -0.5310 | 0.4364 | -0.0341 |
| C_2 | Gh_A08G1486 | BI-1 | Bax inhibitor 1 | 0.3761 | -0.4689 | 0.8410 | 0.1785 |
| C_2 | Gh_A08G1531 | RPN1B | 26S proteasome non-ATPase regulatory subunit 2 | 0.1087 | -1.4467 | -0.3338 | -0.5955 |
|  |  |  | homolog B |  |  |  |  |
| C_2 | Gh_A08G1600 | PLDBETA1 | Phospholipase D beta 1 | 0.0177 | -0.4455 | 1.1716 | -0.0313 |
| C_2 | Gh_A08G1647 | HIS2A | Histone H2AX | 0.4177 | -0.1591 | 0.8794 | 0.6078 |
| C_2 | Gh_A08G1778 | NRT2.1 | High-affinity nitrate transporter 2.1 | -0.7132 | -2.2127 | -0.9818 | -0.1837 |
| C_2 | Gh_A08G1834 | CBP60B | Calmodulin-binding protein 60 B | 0.6785 | -0.0370 | 1.2962 | 0.7826 |
| C_2 | Gh_A08G1879 | ATL6 | E3 ubiquitin-protein ligase ATL6 | 0.0513 | -1.3888 | 0.2925 | 0.5918 |
| C_2 | Gh_A08G1906 | AKR1 | Probable aldo-keto reductase 1 | 0.2951 | -0.3159 | 0.1527 | -0.1216 |
| C_2 | Gh_A08G2290 | RGLG2 | E3 ubiquitin-protein ligase RGLG2 | 0.0931 | -0.1579 | -0.0800 | -0.1756 |
| C_2 | Gh_A08G2301 | At1g11330 | G-type lectin S-receptor-like serine/threonine-protein | -0.2688 | -1.2091 | 0.8303 | 0.3746 |
|  |  |  | kinase |  |  |  |  |
| C_2 | Gh_A08G2322 | CYP89A9 | Cytochrome P450 89A9 | 0.8030 | -2.1373 | -0.7690 | -0.0536 |
| C_2 | Gh_A08G2370 | PRPF18 | Pre-mRNA-splicing factor 18 | 0.2410 | 0.0650 | 0.1321 | -0.1299 |
| C_2 | Gh_A09G0017 | ABCG10 | ABC transporter G family member 10 | 0.2391 | -0.9921 | -0.9345 | -1.9259 |
| C_2 | Gh_A09G0228 | AATP1 | AAA-ATPase ASD, mitochondrial | 1.4500 | -1.7606 | -0.5569 | 0.6368 |
| C_2 | Gh_A09G0482 | SARD1 | Protein SAR DEFICIENT 1 | 0.4372 | -0.9083 | 0.9303 | 0.3223 |
| C_2 | Gh_A09G0661 | JMJ25 | Lysine-specific demethylase JMJ25 | 0.0824 | -0.0621 | 0.0864 | -0.0388 |
| C_2 | Gh_A09G0686 | NA | Polyphenol oxidase, chloroplastic | 0.2847 | -2.2032 | -0.1757 | -1.0538 |
| C_2 | Gh_A09G0779 | VIP2 | Protein PAF1 homolog | 0.4022 | -0.0349 | 0.0854 | 0.0532 |
| C_2 | Gh_A09G0826 | NA | Pyrophosphate-energized vacuolar membrane proton | -0.1869 | -0.8407 | 0.0824 | -0.1691 |
|  |  |  | pump |  |  |  |  |
| C_2 | Gh_A09G0948 | OEP80 | Outer envelope protein 80, chloroplastic | -0.3267 | -1.5725 | 0.0575 | 0.1014 |
| C_2 | Gh_A09G0978 | CBR1 | NADH--cytochrome b5 reductase 1 | 0.1498 | -0.3998 | 0.1237 | -0.0136 |
| C_2 | Gh_A09G0987 | pitB | Phosphatidylinositol transfer protein 2 | 0.2336 | -0.3436 | 1.3687 | -0.1199 |
| C_2 | Gh_A09G1001 | APK2B | Protein kinase 2B, chloroplastic | 0.4021 | -1.4186 | 1.5299 | 0.6420 |
| C_2 | Gh_A09G1020 | F6'H1 | Feruloyl CoA ortho-hydroxylase 1 | 0.2005 | 0.0743 | 0.3676 | -0.1233 |
| C_2 | Gh_A09G1046 | At5g12440 | Zinc finger CCCH domain-containing protein 55 | -0.0063 | -1.2395 | -0.5742 | 0.1793 |
| C_2 | Gh_A09G1057 | NADK1 | NAD(H) kinase 1 | -0.0618 | -0.3231 | 0.2330 | -0.2682 |
| C_2 | Gh_A09G1084 | YLS9 | Protein YLS9 | 0.1712 | -0.8332 | 0.8722 | 0.3309 |
| C_2 | Gh_A09G1134 | FLOT1 | Flotillin-like protein 1 | 0.9016 | -2.3864 | 1.8226 | 0.2698 |
| C_2 | Gh_A09G1305 | At2g23930 | Probable small nuclear ribonucleoprotein G | 0.2829 | -0.2702 | 0.1532 | -0.0225 |
| C_2 | Gh_A09G1356 | ZAT5 | Zinc finger protein ZAT5 | -0.3490 | -1.0555 | -0.0853 | 0.0214 |
| C_2 | Gh_A09G1440 | LECRK42 | L-type lectin-domain containing receptor kinase IV.2 | 0.0351 | -0.8219 | 0.2445 | -0.3390 |
| C_2 | Gh_A09G1501 | GSTL3 | Glutathione S-transferase L3 | -0.0759 | -0.5363 | 0.0657 | -0.2496 |
| C_2 | Gh_A09G1577 | ORTH2 | E3 ubiquitin-protein ligase ORTHRUS 2 | -0.0940 | -2.2301 | 0.7911 | 0.6996 |
| C_2 | Gh_A09G1584 | At1g67000 | Probable receptor-like protein kinase | 0.7375 | -1.9387 | 1.9553 | 2.1330 |
| C_2 | Gh_A09G1722 | CPK34 | Calcium-dependent protein kinase 34 | -0.0659 | -1.2076 | 0.1258 | 0.1442 |
| C_2 | Gh_A09G1814 | PP2B11 | F-box protein PP2-B11 | 0.7005 | -2.7780 | 0.8979 | -0.3767 |
| C_2 | Gh_A09G1845 | HSFA5 | Heat stress transcription factor A-5 | 0.2507 | -0.2681 | 0.6842 | 0.0627 |
| C_2 | Gh_A09G2007 | STK38 | Serine/threonine-protein kinase 38 | 0.0662 | -0.2969 | -0.0576 | 0.2134 |
| C_2 | Gh_A09G2051 | CMD1 | Calmodulin | -0.2103 | -1.8646 | 0.2846 | -0.5851 |
| C_2 | Gh_A09G2213 | EIN4 | Protein EIN4 | 0.2066 | -0.5085 | -0.0639 | -0.4137 |
| C_2 | Gh_A09G2262 | FAF3 | Protein FANTASTIC FOUR 3 | 0.1498 | -1.2371 | -1.1641 | -0.2431 |
| C_2 | Gh_A09G2284 | UGT89C1 | UDP-glycosyltransferase 89C1 | 0.5272 | -0.7660 | -0.6325 | -1.0161 |
| C_2 | Gh_A09G2318 | EP3 | Endochitinase EP3 | 0.8565 | -1.5211 | 2.3809 | 0.5230 |
| C_2 | Gh_A09G2360 | NA | NA | 0.1165 | -0.8647 | -0.6546 | -0.3296 |
| C_2 | Gh_A09G2415 | NSP2 | Nodulation-signaling pathway 2 protein | 0.3986 | -1.0712 | 0.1217 | 0.3682 |
| C_2 | Gh_A09G2435 | LYSRS | Lysine--tRNA ligase | 0.5219 | -2.5164 | 0.4440 | -0.8126 |
| C_2 | Gh_A09G2481 | At1g65240 | Aspartic proteinase-like protein 2 | 0.1072 | -1.5232 | 0.3271 | -0.6594 |
| C_2 | Gh_A10G0061 | At4g20830 | Reticuline oxidase-like protein | -0.1751 | -2.1219 | 3.5648 | -0.4838 |
| C_2 | Gh_A10G0103 | PFP-BETA | Pyrophosphate--fructose 6-phosphate 1- | 0.2963 | 0.0411 | 0.2442 | 0.3674 |
|  |  |  | phosphotransferase subunit beta |  |  |  |  |
| C_2 | Gh_A10G0106 | MLYCD | Malonyl-CoA decarboxylase, mitochondrial | -0.4282 | -0.5258 | 0.4356 | -0.7354 |
| C_2 | Gh_A10G0210 | STC | Sugar carrier protein C | 0.1663 | -0.5485 | -0.1055 | -0.7011 |
| C_2 | Gh_A10G0275 | GLR2.7 | Glutamate receptor 2.7 | 0.5154 | -1.6341 | 2.8946 | -0.6912 |
| C_2 | Gh_A10G0551 | DIR1 | Dirigent protein 1 | 1.9441 | 1.2565 | 1.7855 | 0.3309 |
| C_2 | Gh_A10G0564 | NA | NA | 1.3850 | -2.5670 | 1.6521 | 1.0006 |
| C_2 | Gh_A10G0599 | nep1 | Aspartic proteinase nepenthesin-1 | 0.1339 | -0.3492 | -0.2248 | 0.1326 |
| C_2 | Gh_A10G0612 | FPG1 | Formamidopyrimidine-DNA glycosylase | -0.8199 | -2.8310 | 0.0228 | -0.2784 |
| C_2 | Gh_A10G0661 | NA | NA | -0.3017 | -1.7579 | 1.1618 | -0.3274 |
| C_2 | Gh_A10G0665 | SPX1 | SPX domain-containing protein 1 | 1.3782 | -0.8058 | 2.0316 | -0.0787 |
| C_2 | Gh_A10G0696 | PDR1 | Pleiotropic drug resistance protein 1 | 0.8401 | 0.2113 | 1.6258 | 0.4159 |
| C_2 | Gh_A10G0710 | At1g52360 | Coatomer subunit beta'-2 | -0.0148 | -1.4343 | -1.6812 | -1.5953 |
| C_2 | Gh_A10G0718 | RAB7 | Ras-related protein Rab7 | 0.0451 | -0.2002 | -0.1366 | 0.0074 |
| C_2 | Gh_A10G0780 | At4g40080 | Putative clathrin assembly protein | -0.0414 | -0.7932 | -0.1330 | -0.0398 |
| C_2 | Gh_A10G0842 | WAKL14 | Wall-associated receptor kinase-like 14 | 0.0859 | -0.8296 | 0.9271 | -0.1156 |
| C_2 | Gh_A10G0896 | NA | NA | 0.4501 | -1.2172 | 0.6227 | 0.0936 |
| C_2 | Gh_A10G0923 | CRK42 | Cysteine-rich receptor-like protein kinase 42 | -0.3970 | -0.8368 | 0.2021 | -0.4428 |
| C_2 | Gh_A10G0956 | AMT1-1 | Ammonium transporter 1 member 1 | 0.2138 | -1.3634 | 0.3283 | 0.9539 |
| C_2 | Gh_A10G1124 | PHT1-5 | Probable inorganic phosphate transporter 1-5 | 1.0534 | -1.1483 | -0.5639 | -0.7000 |
| C_2 | Gh_A10G1175 | NA | NA | 1.1003 | -3.2478 | 2.7301 | 4.3344 |
| C_2 | Gh_A10G1254 | NA | NA | 0.9579 | 0.2167 | 0.2271 | -0.2570 |
| C_2 | Gh_A10G1266 | NA | NA | -0.4089 | -2.1646 | 1.8589 | 0.7327 |
| C_2 | Gh_A10G1494 | NA | Major allergen Pru ar 1 | 1.1962 | 0.0936 | 0.2875 | -0.3467 |
| C_2 | Gh_A10G1497 | NA | NA | -0.2115 | -1.1102 | -0.1633 | 0.3159 |
| C_2 | Gh_A10G1507 | BETVIA | Major pollen allergen Bet v 1-A | -0.3261 | -2.4252 | 0.7428 | 0.8929 |
| C_2 | Gh_A10G1509 | BETV1F | Major pollen allergen Bet v 1-F/I | -0.3742 | -1.9081 | 0.1961 | 0.0147 |
| C_2 | Gh_A10G1513 | BETVIA | Major pollen allergen Bet v 1-A | 0.6879 | -0.9944 | 0.3427 | -0.3959 |
| C_2 | Gh_A10G1584 | Y-1 | Uncharacterized protein At5g05190 | 0.5010 | -1.1118 | 0.8320 | 0.6331 |
| C_2 | Gh_A10G1587 | NA | NA | 0.7123 | -1.0379 | 1.1412 | -0.1058 |
| C_2 | Gh_A10G1750 | UBP12 | Ubiquitin carboxyl-terminal hydrolase 12 | 0.5578 | -0.2495 | 0.4795 | -0.2718 |
| C_2 | Gh_A10G1753 | UBP12 | Ubiquitin carboxyl-terminal hydrolase 12 | 0.3527 | -0.8404 | 0.1135 | -0.4189 |
| C_2 | Gh_A10G1775 | NA | NA | 0.4797 | -2.2994 | -1.9991 | 0.5284 |
| C_2 | Gh_A10G1776 | NA | NA | 0.8001 | -2.0013 | -1.5858 | 0.0050 |
| C_2 | Gh_A10G1803 | NFD4 | Protein NUCLEAR FUSION DEFECTIVE 4 | 0.1839 | -1.3323 | 1.3470 | 0.1749 |
| C_2 | Gh_A10G1947 | DOF3.6 | Dof zinc finger protein DOF3.6 | 0.1695 | -0.0992 | 0.3475 | -0.4462 |
| C_2 | Gh_A10G1957 | NA | NA | 0.1254 | -1.3010 | 1.1066 | -0.1482 |
| C_2 | Gh_A10G1975 | ERG1 | Elicitor-responsive protein 1 | -0.5409 | -0.9344 | 0.5891 | -0.6384 |
| C_2 | Gh_A11G0164 | CID11 | Polyadenylate-binding protein-interacting protein 11 | 0.0636 | -0.8656 | -0.4280 | -0.3823 |
| C_2 | Gh_A11G0399 | At3g51070 | Probable methyltransferase PMT27 | 0.3593 | -2.0315 | -0.1638 | 0.3536 |
| C_2 | Gh_A11G0547 | At1g47710 | Serpin-ZX | -0.1699 | -2.4618 | 1.5467 | 0.8564 |
| C_2 | Gh_A11G0663 | TKPR1 | Tetraketide alpha-pyrone reductase 1 | 0.1194 | -0.7100 | 0.1294 | 0.1698 |
| C_2 | Gh_A11G0674 | PIP5K8 | Phosphatidylinositol 4-phosphate 5-kinase 8 | -0.0646 | -1.2117 | 0.5580 | 0.1539 |
| C_2 | Gh_A11G0721 | At1g63370 | Flavin-containing monooxygenase FMO GS-OX-like 5 | -0.0932 | -1.1679 | -0.4842 | -0.3898 |
| C_2 | Gh_A11G0799 | PUB33 | U-box domain-containing protein 33 | 0.6974 | -0.5348 | 1.9068 | 0.5509 |
| C_2 | Gh_A11G0932 | NAC062 | NAC domain-containing protein 62 | -0.2416 | -1.4170 | -0.1036 | -0.7775 |
| C_2 | Gh_A11G0968 | SPCC1223.01 | E3 ubiquitin-protein ligase hel2 | 0.5603 | -1.4407 | 2.4549 | 1.1068 |
| C_2 | Gh_A11G1084 | NA | Secoisolariciresinol dehydrogenase (Fragment) | 0.5054 | -0.4658 | -0.2970 | 0.0112 |
| C_2 | Gh_A11G1119 | ALMT12 | Aluminum-activated malate transporter 12 | 0.7156 | -0.9940 | 2.2133 | -0.2532 |
| C_2 | Gh_A11G1131 | NA | Ent-copalyl diphosphate synthase, chloroplastic | 0.6808 | 0.0410 | 0.8972 | 0.7551 |
| C_2 | Gh_A11G1143 | At2g13820 | Non-specific lipid-transfer protein-like protein | 0.7368 | -0.8216 | 0.9091 | 0.0927 |
| C_2 | Gh_A11G1152 | CAM | Calmodulin | -0.1539 | -2.9386 | 1.4216 | 0.1323 |
| C_2 | Gh_A11G1160 | CPRF2 | Light-inducible protein CPRF2 | 0.3002 | -0.3551 | 0.6665 | 0.9425 |
| C_2 | Gh_A11G1179 | NA | Glu S.griseus protease inhibitor | 1.2710 | -1.8628 | -0.7049 | -1.3659 |
| C_2 | Gh_A11G1244 | NA | Probable glutathione S-transferase | 0.6999 | -1.8362 | 1.4015 | 0.0803 |
| C_2 | Gh_A11G1450 | NA | NA | 0.7376 | 0.2455 | 0.4935 | 0.6839 |
| C_2 | Gh_A11G1461 | FER | Receptor-like protein kinase FERONIA | 0.4665 | -0.7093 | 0.7171 | 0.4325 |
| C_2 | Gh_A11G1522 | At1g67000 | Probable receptor-like protein kinase | 1.2198 | -0.0566 | 1.3607 | 0.9076 |
| C_2 | Gh_A11G1537 | NA | NA | -0.5112 | -1.8681 | -0.0765 | -0.8412 |
| C_2 | Gh_A11G1539 | HACL | 2-hydroxyacyl-CoA lyase | -0.1248 | -0.4333 | -0.2131 | 0.0401 |
| C_2 | Gh_A11G1623 | At3g50940 | AAA-ATPase | 0.4516 | -0.6349 | 0.8045 | 0.7361 |
| C_2 | Gh_A11G1630 | MUR1 | GDP-mannose 4,6 dehydratase 2 | 0.3538 | -0.0217 | 0.6906 | 0.2971 |
| C_2 | Gh_A11G1837 | RABH1E | Ras-related protein RABH1e | -0.0763 | -0.5723 | 0.6158 | -0.2858 |
| C_2 | Gh_A11G1875 | LECRK91 | L-type lectin-domain containing receptor kinase IX.1 | 0.8245 | -0.8930 | 0.2611 | 0.3402 |
| C_2 | Gh_A11G1937 | HHP4 | Heptahelical transmembrane protein 4 | -0.1374 | -0.5743 | -0.5279 | -0.3856 |
| C_2 | Gh_A11G2003 | At5g24010 | Probable receptor-like protein kinase | 0.0298 | -0.6297 | -0.0568 | -0.0379 |
| C_2 | Gh_A11G2095 | TUBA3 | Tubulin alpha-3 chain | 0.1224 | -0.1585 | 0.3534 | -0.7340 |
| C_2 | Gh_A11G2137 | NA | NA | -0.0183 | -1.3939 | -0.5872 | -0.0028 |
| C_2 | Gh_A11G2216 | FTSHI5 | Probable inactive ATP-dependent zinc metalloprotease | -0.0182 | -0.2149 | 0.0237 | 0.1035 |
|  |  |  | FTSHI 5, chloroplastic |  |  |  |  |
| C_2 | Gh_A11G2290 | AKR1 | Probable aldo-keto reductase 1 | -0.0716 | -1.3609 | 0.1607 | -0.3455 |
| C_2 | Gh_A11G2387 | ATL6 | E3 ubiquitin-protein ligase ATL6 | 0.0937 | -1.2476 | 1.3331 | 0.2545 |
| C_2 | Gh_A11G2431 | At3g05500 | REF/SRPP-like protein | 0.3929 | -0.3414 | 0.0767 | -0.1325 |
| C_2 | Gh_A11G2440 | NA | Serine/threonine-protein phosphatase PP1 | 0.6022 | -2.2459 | 0.4016 | 0.2452 |
| C_2 | Gh_A11G2491 | CHLN | Nicotianamine synthase | -0.9524 | -2.0038 | -1.3485 | 0.4058 |
| C_2 | Gh_A11G2494 | PIF3 | Transcription factor PIF3 | -0.2470 | -1.1839 | -1.0221 | 0.0271 |
| C_2 | Gh_A11G2508 | ABCC3 | ABC transporter C family member 3 | 1.1608 | -2.4567 | 3.4280 | -0.8146 |
| C_2 | Gh_A11G2625 | 7OMT | (R,S)-reticuline 7-O-methyltransferase | 0.1951 | -1.4281 | 0.1843 | 0.3433 |
| C_2 | Gh_A11G2819 | MED7B | Mediator of RNA polymerase II transcription subunit 7b | 0.0737 | -1.0454 | -0.2094 | -0.1032 |
| C_2 | Gh_A11G2851 | UBICEP52-7 | Ubiquitin-60S ribosomal protein L40 | -0.1211 | -0.2738 | 0.6695 | -0.3591 |
| C_2 | Gh_A11G3003 | ABCA2 | ABC transporter A family member 2 | 0.2550 | -1.2358 | 0.9330 | -0.3142 |
| C_2 | Gh_A11G3049 | CXE8 | Probable carboxylesterase 8 | 0.2524 | -0.8518 | -0.5517 | -0.1026 |
| C_2 | Gh_A11G3171 | POLD4 | DNA polymerase delta subunit 4 | -0.1349 | -1.1059 | -0.9616 | -0.6533 |
| C_2 | Gh_A11G3289 | At1g67000 | Probable receptor-like protein kinase | -0.6983 | -1.9141 | -0.5382 | 0.3568 |
| C_2 | Gh_A12G0095 | UDP-GALT2 | UDP-galactose transporter 2 | -0.4219 | -3.0454 | 1.3271 | 0.5543 |
| C_2 | Gh_A12G0103 | HMG1 | 3-hydroxy-3-methylglutaryl-coenzyme A reductase 1 | -0.0489 | -1.1795 | 0.9506 | -0.0550 |
| C_2 | Gh_A12G0132 | yrpB | Probable nitronate monooxygenase | 0.1071 | -0.6562 | -0.3354 | 0.1264 |
| C_2 | Gh_A12G0180 | NA | NA | 0.3215 | -0.1780 | 0.6216 | 0.3262 |
| C_2 | Gh_A12G0187 | FUC95A | Alpha-L-fucosidase 2 | -1.4424 | -2.4765 | 0.1419 | -0.9488 |
| C_2 | Gh_A12G0319 | ANAC094 | Putative NAC domain-containing protein 94 | 1.4100 | -1.6492 | 3.3604 | 1.0974 |
| C_2 | Gh_A12G0424 | ABCC2 | ABC transporter C family member 2 | 0.4125 | -0.1366 | 0.6270 | 0.1459 |
| C_2 | Gh_A12G0441 | NA | NA | 0.2232 | -1.2642 | 1.3030 | 1.1180 |
| C_2 | Gh_A12G0615 | At2g18630 | UPF0496 protein At2g18630 | 0.3552 | -0.2354 | 0.8644 | 0.4686 |
| C_2 | Gh_A12G0840 | NA | L-ascorbate oxidase | 0.6113 | -1.5134 | 1.4604 | 0.3388 |
| C_2 | Gh_A12G0924 | NA | NA | 0.7936 | -1.6869 | -0.2229 | 3.6681 |
| C_2 | Gh_A12G1004 | CNGC13 | Putative cyclic nucleotide-gated ion channel 13 | 0.4422 | -0.3602 | 1.2384 | 0.2557 |
| C_2 | Gh_A12G1029 | CYP78A3 | Cytochrome P450 78A3 | 0.7350 | -0.7041 | -0.6740 | -0.2294 |
| C_2 | Gh_A12G1076 | GLP10 | Germin-like protein subfamily 2 member 4 | 0.0375 | -0.1589 | 0.2639 | 0.1637 |
| C_2 | Gh_A12G1265 | NA | NA | 0.0422 | -1.3229 | -0.2895 | -0.1662 |
| C_2 | Gh_A12G1318 | SRO2 | Probable inactive poly [ADP-ribose] polymerase SRO2 | -0.2096 | -0.6081 | -0.3736 | -0.1757 |
| C_2 | Gh_A12G1365 | NA | NA | 0.3189 | -1.4397 | -0.2163 | -0.4122 |
| C_2 | Gh_A12G1407 | ADO3 | Adagio protein 3 | 0.9716 | -3.3509 | 1.9924 | -1.4993 |
| C_2 | Gh_A12G1513 | RNS1 | Ribonuclease 1 | 0.1951 | -0.6627 | -0.0063 | 0.4207 |
| C_2 | Gh_A12G1577 | ALA9 | Putative phospholipid-transporting ATPase 9 | 0.0180 | -0.7551 | 1.3635 | 0.4109 |
| C_2 | Gh_A12G1598 | ABCA7 | ABC transporter A family member 7 | 0.5025 | -0.5645 | 0.7011 | -0.1334 |
| C_2 | Gh_A12G1689 | SOBIR1 | Leucine-rich repeat receptor-like | 0.8198 | -0.9029 | 1.4975 | 0.2673 |
|  |  |  | serine/threonine/tyrosine-protein kinase |  |  |  |  |
| C_2 | Gh_A12G1846 | ERD3 | Probable methyltransferase PMT21 | 1.0378 | 0.5314 | 0.9032 | 0.0881 |
| C_2 | Gh_A12G1864 | CYP76B6 | Geraniol 8-hydroxylase | 0.8517 | -0.2741 | -0.0959 | 0.1801 |
| C_2 | Gh_A12G1865 | RPM1 | Disease resistance protein RPM1 | 0.7329 | -0.9748 | 1.4152 | 1.3237 |
| C_2 | Gh_A12G1870 | erg8 | Probable phosphomevalonate kinase | -0.1728 | -0.4746 | 0.6539 | -0.3279 |
| C_2 | Gh_A12G1892 | AGD8 | ADP-ribosylation factor GTPase-activating protein | 0.1914 | -0.2041 | 0.6266 | -0.0181 |
| C_2 | Gh_A12G1949 | At4g16820 | Phospholipase A1-Ibeta2, chloroplastic | 0.1286 | -1.4176 | -0.1480 | 0.0908 |
| C_2 | Gh_A12G2187 | SBT3.3 | Subtilisin-like protease SBT3.3 | 0.4695 | -0.7323 | 0.4917 | -1.0805 |
| C_2 | Gh_A12G2189 | NA | NA | -0.6624 | -1.6120 | 0.0497 | -0.8205 |
| C_2 | Gh_A12G2190 | COMT1 | Caffeic acid 3-O-methyltransferase | -0.0559 | -1.0305 | -0.0124 | 0.0206 |
| C_2 | Gh_A12G2203 | CAD1 | Probable cinnamyl alcohol dehydrogenase 1 | -0.3240 | -0.9486 | 0.1757 | -0.3585 |
| C_2 | Gh_A12G2296 | NA | NA | 0.7044 | -0.4137 | 1.9225 | 0.4606 |
| C_2 | Gh_A12G2352 | BAG1 | BAG family molecular chaperone regulator 1 | 0.0152 | -0.1231 | 0.6756 | -0.4139 |
| C_2 | Gh_A12G2375 | CXE1 | Carboxylesterase 1 | 0.0031 | -2.7440 | 0.2836 | 1.0069 |
| C_2 | Gh_A12G2425 | SARD1 | Protein SAR DEFICIENT 1 | 0.5265 | -0.8285 | 1.3103 | 0.7328 |
| C_2 | Gh_A12G2432 | CAR4 | Protein C2-DOMAIN ABA-RELATED 4 | 0.3522 | 0.1096 | 0.4042 | 0.0360 |
| C_2 | Gh_A12G2493 | AOX1A | Ubiquinol oxidase 1a, mitochondrial | -0.0897 | -2.2530 | 0.3586 | -1.5765 |
| C_2 | Gh_A12G2710 | ABCC10 | ABC transporter C family member 10 | 0.2120 | -1.0059 | 0.2516 | -0.8583 |
| C_2 | Gh_A13G0029 | ARR8 | Two-component response regulator ARR8 | 1.1913 | 0.2968 | 2.1550 | 0.3691 |
| C_2 | Gh_A13G0045 | DRP4C | Dynamin-related protein 4C | -1.2038 | -1.5248 | -1.0988 | -1.4674 |
| C_2 | Gh_A13G0046 | DRP4C | Dynamin-related protein 4C | -0.6130 | -1.6002 | -0.7000 | -1.6135 |
| C_2 | Gh_A13G0155 | CYP714A1 | Cytochrome P450 714A1 | 0.6856 | -1.8520 | 0.9911 | -1.1971 |
| C_2 | Gh_A13G0202 | RS2Z32 | Serine/arginine-rich splicing factor RS2Z32 | 0.2897 | 0.0722 | 0.1253 | 0.1396 |
| C_2 | Gh_A13G0229 | NA | NA | 0.8222 | -2.1175 | 3.4952 | 1.0380 |
| C_2 | Gh_A13G0247 | RDR1 | RNA-dependent RNA polymerase 1 | 0.1746 | -0.7131 | -0.1196 | 0.0257 |
| C_2 | Gh_A13G0289 | PAP2 | Purple acid phosphatase 2 | 0.9652 | -0.5491 | 0.5589 | -0.1702 |
| C_2 | Gh_A13G0371 | TOGT1 | Scopoletin glucosyltransferase | -0.5279 | -3.5146 | 0.1727 | 0.2702 |
| C_2 | Gh_A13G0374 | TOGT1 | Scopoletin glucosyltransferase | 0.3241 | -1.6536 | 1.0042 | 0.5323 |
| C_2 | Gh_A13G0387 | NA | Basic secretory protease (Fragments) | 0.5901 | -1.5192 | 0.8803 | -1.0801 |
| C_2 | Gh_A13G0400 | NA | NA | 0.2586 | -2.5406 | -0.5197 | 0.1756 |
| C_2 | Gh_A13G0427 | LECRK91 | L-type lectin-domain containing receptor kinase IX.1 | -0.0998 | -2.9726 | 0.7663 | 1.6415 |
| C_2 | Gh_A13G0434 | DRP3A | Dynamin-related protein 3A | -0.0304 | -0.1252 | 1.2544 | -0.9149 |
| C_2 | Gh_A13G0617 | NA | NA | -1.3242 | -1.2242 | -0.0077 | -2.4439 |
| C_2 | Gh_A13G0630 | WRKY72 | Probable WRKY transcription factor 72 | 0.4017 | -0.9656 | -0.3605 | -0.7633 |
| C_2 | Gh_A13G0643 | NA | NA | 0.0987 | -1.0097 | -0.4306 | -0.0653 |
| C_2 | Gh_A13G0692 | NA | NA | 0.7283 | -1.7668 | 0.5107 | -0.3869 |
| C_2 | Gh_A13G0693 | NA | NA | 0.7722 | -1.2770 | 0.1371 | -0.1508 |
| C_2 | Gh_A13G0728 | At1g15670 | F-box/kelch-repeat protein | 0.0949 | -1.7887 | 1.2372 | -0.6223 |
| C_2 | Gh_A13G0767 | PUB42 | Putative U-box domain-containing protein 42 | -0.0991 | -1.3560 | 0.1095 | -0.0416 |
| C_2 | Gh_A13G0895 | BIP5 | Luminal-binding protein 5 | 0.4921 | -2.5637 | -0.2154 | 0.0284 |
| C_2 | Gh_A13G0925 | NA | Pyrophosphate-energized vacuolar membrane proton | 1.1343 | -1.9383 | -1.8237 | 0.2130 |
|  |  |  | pump |  |  |  |  |
| C_2 | Gh_A13G1132 | SAT3 | Serine acetyltransferase 3, mitochondrial | -1.1035 | -3.2685 | -0.5620 | -0.8228 |
| C_2 | Gh_A13G1196 | Os02g0190300 | Putative multidrug resistance protein | 0.4092 | -1.0008 | -0.9787 | -0.5995 |
| C_2 | Gh_A13G1198 | FIE2 | Polycomb group protein FIE2 | 0.1618 | -0.1335 | -0.1368 | 0.0737 |
| C_2 | Gh_A13G1349 | UGT73C1 | UDP-glycosyltransferase 73C1 | 0.3559 | -1.7622 | -0.0814 | -0.6020 |
| C_2 | Gh_A13G1373 | ppk15 | Serine/threonine-protein kinase ppk15 | 0.4933 | -0.0548 | -0.0739 | -0.3615 |
| C_2 | Gh_A13G1551 | NA | NA | -0.1590 | -0.5370 | 0.3446 | -0.1729 |
| C_2 | Gh_A13G1564 | SOT16 | Cytosolic sulfotransferase 16 | 0.7158 | -1.7912 | 0.1628 | -0.4945 |
| C_2 | Gh_A13G1648 | TIM17-2 | Mitochondrial import inner membrane translocase subunit | 0.0717 | -0.3863 | 1.1095 | -0.1494 |
| C_2 | Gh_A13G1658 | APM1 | Aminopeptidase M1 | 0.7893 | 0.5685 | 1.2243 | 0.1099 |
| C_2 | Gh_A13G1686 | At3g45310 | Thiol protease aleurain-like | -0.2727 | -1.6140 | -0.1172 | -0.3345 |
| C_2 | Gh_A13G1787 | AOP1 | Probable 2-oxoglutarate-dependent dioxygenase AOP1 | 0.0523 | -1.7348 | 1.2401 | -0.3631 |
| C_2 | Gh_A13G1797 | AOP1 | Probable 2-oxoglutarate-dependent dioxygenase AOP1 | 0.1259 | -1.4945 | -0.0854 | 1.7512 |
| C_2 | Gh_A13G1798 | AOP1 | Probable 2-oxoglutarate-dependent dioxygenase AOP1 | 0.1507 | -0.9540 | 0.2312 | 1.5307 |
| C_2 | Gh_A13G1827 | NA | NA | 0.1268 | -0.1823 | 0.6013 | 0.0871 |
| C_2 | Gh_A13G1926 | aifA | Apoptosis-inducing factor homolog A | 0.2702 | -1.4788 | 0.9007 | -0.1979 |
| C_2 | Gh_A13G1951 | ATOBGM | Probable GTP-binding protein OBGM, mitochondrial | 0.7411 | -0.5123 | -0.5278 | 0.2257 |
| C_2 | Gh_A13G1959 | NA | NA | 0.1611 | -0.9125 | -0.5524 | -0.2314 |
| C_2 | Gh_A13G2105 | RLIM | E3 ubiquitin-protein ligase RLIM | 0.3038 | -0.0521 | 0.9751 | 0.2568 |
| C_2 | Gh_A13G2118 | VDAC5 | Mitochondrial outer membrane protein porin 5 | 0.0635 | -2.1599 | 0.7724 | -0.0427 |
| C_2 | Gh_A13G2175 | NPC3 | Non-specific phospholipase C3 | 0.1749 | 0.0652 | 0.1028 | -0.0502 |
| C_2 | Gh_A13G2341 | AOP1 | Probable 2-oxoglutarate-dependent dioxygenase AOP1 | 0.0411 | -1.2759 | -0.4655 | 0.3870 |
| C_2 | Gh_A13G2343 | AOP1 | Probable 2-oxoglutarate-dependent dioxygenase AOP1 | 0.2913 | -0.8067 | 0.1807 | -0.1338 |
| C_2 | Gh_D01G0131 | MSSP2 | Monosaccharide-sensing protein 2 | -0.1656 | -1.1391 | -0.5659 | -0.0888 |
| C_2 | Gh_D01G0208 | NA | Glucose-6-phosphate 1-dehydrogenase, chloroplastic | -0.3174 | -1.6030 | -0.1990 | 0.3311 |
| C_2 | Gh_D01G0260 | At1g11330 | G-type lectin S-receptor-like serine/threonine-protein | -0.7744 | -1.2285 | -0.3980 | -0.6779 |
|  |  |  | kinase |  |  |  |  |
| C_2 | Gh_D01G0268 | At1g11330 | G-type lectin S-receptor-like serine/threonine-protein | -0.3125 | -1.5324 | 0.6367 | -0.0090 |
|  |  |  | kinase |  |  |  |  |
| C_2 | Gh_D01G0281 | F6'H1 | Feruloyl CoA ortho-hydroxylase 1 | 0.4661 | -3.0207 | 1.0330 | -0.2248 |
| C_2 | Gh_D01G0325 | At5g39020 | Probable receptor-like protein kinase | 0.9475 | -0.8149 | 1.2901 | 0.0193 |
| C_2 | Gh_D01G0332 | At4g27220 | Probable disease resistance protein | -0.1688 | -0.6214 | -0.1259 | -0.5902 |
| C_2 | Gh_D01G0337 | DIR4 | Dirigent protein 4 | -0.1490 | -1.2576 | -0.1036 | -0.6885 |
| C_2 | Gh_D01G0341 | SRG1 | Protein SRG1 | 0.7710 | -1.2089 | -1.4256 | -1.2093 |
| C_2 | Gh_D01G0390 | BEH4 | BES1/BZR1 homolog protein 4 | 0.7823 | 0.4334 | 0.7952 | -0.1651 |
| C_2 | Gh_D01G0406 | NA | NA | -0.3667 | -3.5889 | -0.2137 | 0.4307 |
| C_2 | Gh_D01G0409 | NA | NA | -0.1147 | -0.4261 | -0.2925 | -0.4213 |
| C_2 | Gh_D01G0492 | EXPA12 | Expansin-A12 | 1.8055 | -1.4835 | 1.8782 | 0.0685 |
| C_2 | Gh_D01G0520 | NA | NA | 0.8853 | -1.5285 | 1.4647 | 0.9929 |
| C_2 | Gh_D01G0546 | At4g03230 | G-type lectin S-receptor-like serine/threonine-protein | 0.1131 | -2.0945 | 0.5259 | 0.6258 |
|  |  |  | kinase |  |  |  |  |
| C_2 | Gh_D01G0628 | NA | NA | 0.3346 | -0.4514 | -0.1198 | -0.1997 |
| C_2 | Gh_D01G0679 | LIP5 | Protein HOMOLOG OF MAMMALIAN LYST-INTERACTING PROTEIN 5 | 0.1773 | -0.1765 | -0.0433 | -0.2102 |
| C_2 | Gh_D01G0723 | ZIP11 | Zinc transporter 11 | 1.1602 | -0.2313 | 1.4495 | 1.4707 |
| C_2 | Gh_D01G0774 | RLK1 | G-type lectin S-receptor-like serine/threonine-protein | 0.2243 | -1.6987 | 0.2658 | -0.1340 |
|  |  |  | kinase RLK1 |  |  |  |  |
| C_2 | Gh_D01G0780 | XERICO | Probable E3 ubiquitin-protein ligase XERICO | 0.3730 | -1.0402 | 0.9844 | -0.0292 |
| C_2 | Gh_D01G0787 | At1g56130 | Probable LRR receptor-like serine/threonine-protein | 0.2153 | -1.6126 | -0.3464 | -0.0263 |
|  |  |  | kinase |  |  |  |  |
| C_2 | Gh_D01G0789 | SAUR72 | Auxin-responsive protein SAUR72 | 0.4368 | -1.2884 | 1.0770 | -0.8824 |
| C_2 | Gh_D01G0803 | NA | NA | -0.7416 | -1.5956 | -1.2475 | 0.0269 |
| C_2 | Gh_D01G0804 | UGT73C5 | UDP-glycosyltransferase 73C5 | 2.0145 | -3.5281 | 2.3852 | -0.0949 |
| C_2 | Gh_D01G0806 | NA | NA | 1.1602 | -5.0947 | -0.3364 | -1.8435 |
| C_2 | Gh_D01G0929 | RBK2 | Receptor-like cytosolic serine/threonine-protein kinase | -0.0850 | -0.7791 | 0.3985 | -0.7102 |
|  |  |  | RBK2 |  |  |  |  |
| C_2 | Gh_D01G1121 | PUP3 | Purine permease 3 | 0.1448 | -1.7691 | -0.8765 | -0.7171 |
| C_2 | Gh_D01G1191 | SYP121 | Syntaxin-121 | -0.2582 | -1.1607 | 0.7182 | 0.1349 |
| C_2 | Gh_D01G1328 | PAP3 | Purple acid phosphatase 3 | 0.4027 | -0.2254 | -0.2173 | 0.0546 |
| C_2 | Gh_D01G1470 | HOX3 | Homeobox-leucine zipper protein HOX3 | 0.4290 | -3.9937 | -0.5143 | -0.1663 |
| C_2 | Gh_D01G1489 | SUD1 | Probable E3 ubiquitin ligase SUD1 | 0.5452 | -0.2302 | 1.3961 | -0.0887 |
| C_2 | Gh_D01G1561 | MKP1 | Protein-tyrosine-phosphatase MKP1 | 0.2254 | -0.7563 | 0.4376 | -0.0248 |
| C_2 | Gh_D01G1814 | PUR2 | Phosphoribosylamine--glycine ligase, chloroplastic | 0.5660 | -0.5997 | -0.2323 | -0.3107 |
| C_2 | Gh_D01G1820 | TPPD | Probable trehalose-phosphate phosphatase D | 0.6902 | -0.4732 | -0.2247 | -0.0795 |
| C_2 | Gh_D01G1986 | NA | Secoisolariciresinol dehydrogenase (Fragment) | 0.4386 | -0.9264 | 0.3590 | -0.2236 |
| C_2 | Gh_D01G1988 | NA | Secoisolariciresinol dehydrogenase (Fragment) | 0.3661 | -0.9963 | 1.0482 | -0.0686 |
| C_2 | Gh_D01G1989 | NA | Secoisolariciresinol dehydrogenase (Fragment) | 0.3756 | -1.1305 | 0.2199 | -0.5454 |
| C_2 | Gh_D01G2145 | CERBERUS | Putative E3 ubiquitin-protein ligase LIN-1 | 0.0303 | -1.1036 | -0.1054 | -0.5815 |
| C_2 | Gh_D01G2186 | NA | NA | 0.3356 | -0.8601 | 1.4857 | 0.1848 |
| C_2 | Gh_D01G2211 | SQD2 | Sulfoquinovosyl transferase SQD2 | 0.2504 | -0.8851 | -0.6614 | -0.3205 |
| C_2 | Gh_D01G2242 | At1g14600 | Putative Myb family transcription factor | 0.1524 | -0.5800 | 0.0192 | -0.2659 |
| C_2 | Gh_D01G2314 | CYP72A15 | Cytochrome P450 72A15 | 0.3155 | -0.3052 | -0.1456 | -0.0086 |
| C_2 | Gh_D02G0021 | NA | NA | -0.1258 | -0.3688 | -0.2322 | -0.6223 |
| C_2 | Gh_D02G0216 | CBDAS2 | Cannabidiolic acid synthase-like 1 | -0.2463 | -1.7235 | -0.5149 | 0.3744 |
| C_2 | Gh_D02G0225 | UGT75L6 | Crocetin glucosyltransferase, chloroplastic | -0.5287 | -1.7529 | -0.9716 | -0.2826 |
| C_2 | Gh_D02G0229 | UGT75L6 | Crocetin glucosyltransferase, chloroplastic | 0.6559 | -1.5543 | -0.0874 | 1.7824 |
| C_2 | Gh_D02G0257 | RLP12 | Receptor-like protein 12 | 0.1092 | -1.9191 | 1.3570 | 1.8540 |
| C_2 | Gh_D02G0259 | RLP12 | Receptor-like protein 12 | -0.0004 | -1.0697 | 1.4175 | 0.5522 |
| C_2 | Gh_D02G0404 | CIPK7 | CBL-interacting serine/threonine-protein kinase 7 | 0.7003 | -0.9293 | 0.2526 | -1.4648 |
| C_2 | Gh_D02G0504 | NHX2 | Sodium/hydrogen exchanger 2 | 0.1653 | -0.4375 | -0.1930 | 0.1162 |
| C_2 | Gh_D02G0560 | NA | Major allergen Pru ar 1 | 0.3418 | -0.5858 | 0.0571 | 0.1912 |
| C_2 | Gh_D02G0578 | IMPA2 | Importin subunit alpha-2 | 0.2074 | 0.0229 | 0.0649 | 0.0704 |
| C_2 | Gh_D02G0580 | AAE7 | Acetate/butyrate--CoA ligase AAE7, peroxisomal | 0.3879 | -0.6257 | 0.5388 | 0.1361 |
| C_2 | Gh_D02G0636 | SAMS | S-adenosylmethionine synthase | 0.4644 | -0.2379 | 0.0745 | -0.7255 |
| C_2 | Gh_D02G0671 | STOP1 | Protein SENSITIVE TO PROTON RHIZOTOXICITY 1 | 0.0353 | -0.3952 | -0.0882 | -0.0558 |
| C_2 | Gh_D02G0732 | ADH1 | Alcohol dehydrogenase 1 | 0.7903 | 0.6649 | 1.3264 | 0.4155 |
| C_2 | Gh_D02G0752 | NA | NA | 0.3728 | -0.6946 | 1.3409 | 0.2706 |
| C_2 | Gh_D02G0756 | SKP2A | F-box protein SKP2A | 0.2408 | -0.4836 | 0.1791 | 0.6013 |
| C_2 | Gh_D02G0770 | WAK2 | Wall-associated receptor kinase 2 | -0.0632 | -2.1141 | 2.5297 | 0.7442 |
| C_2 | Gh_D02G0783 | At1g77330 | 1-aminocyclopropane-1-carboxylate oxidase 5 | 1.0706 | -1.7249 | -0.7039 | -1.0248 |
| C_2 | Gh_D02G0870 | ROG1 | Putative lipase ROG1 | -0.0142 | -1.6673 | 0.6687 | -0.3714 |
| C_2 | Gh_D02G0935 | HOS3 | Elongation of fatty acids protein 3-like | -1.0734 | -3.7467 | 0.7237 | 0.1511 |
| C_2 | Gh_D02G1013 | PHT1-9 | Probable inorganic phosphate transporter 1-9 | 0.4301 | -2.1902 | -0.0673 | -0.2735 |
| C_2 | Gh_D02G1086 | NA | NA | -0.1346 | -2.6582 | 0.4582 | 0.6494 |
| C_2 | Gh_D02G1221 | RPL19B | 60S ribosomal protein L19-2 | 1.0881 | -2.7598 | 0.3658 | -1.2538 |
| C_2 | Gh_D02G1336 | DTX40 | Protein DETOXIFICATION 40 | -0.3641 | -1.2202 | -1.1700 | -0.6206 |
| C_2 | Gh_D02G1493 | WRKY75 | Probable WRKY transcription factor 75 | -0.0234 | -0.6723 | 0.8479 | -0.3263 |
| C_2 | Gh_D02G1579 | At1g67750 | Probable pectate lyase 5 | 1.1444 | -0.8069 | -0.6814 | -0.1980 |
| C_2 | Gh_D02G1620 | DTX29 | Protein DETOXIFICATION 29 | 0.3227 | 0.0166 | 0.6133 | 0.4085 |
| C_2 | Gh_D02G1622 | NA | NA | 0.3878 | -0.9065 | 0.5007 | 0.3632 |
| C_2 | Gh_D02G1645 | At1g60420 | Probable nucleoredoxin 1 | 0.2852 | -0.9882 | 0.3520 | -0.0621 |
| C_2 | Gh_D02G1761 | DTX12 | Protein DETOXIFICATION 12 | -0.1631 | -0.7564 | -0.6174 | -0.5168 |
| C_2 | Gh_D02G1762 | DTX12 | Protein DETOXIFICATION 12 | 0.2279 | -1.4890 | 0.2433 | -0.2209 |
| C_2 | Gh_D02G1782 | NA | NA | 0.2826 | 0.0636 | 0.3596 | 0.0828 |
| C_2 | Gh_D02G1816 | TAT | Tyrosine aminotransferase | 0.3763 | -0.4061 | 2.0030 | 0.5460 |
| C_2 | Gh_D02G1849 | 7OMT | (R,S)-reticuline 7-O-methyltransferase | 0.3926 | -1.6637 | -0.2480 | -0.0195 |
| C_2 | Gh_D02G1945 | NA | NA | -0.2620 | -1.1403 | 0.2581 | -1.1110 |
| C_2 | Gh_D02G1965 | HMG1 | 3-hydroxy-3-methylglutaryl-coenzyme A reductase 1 | -0.1494 | -0.6082 | -0.0433 | -0.0781 |
| C_2 | Gh_D02G1980 | At4g35600 | Probable serine/threonine-protein kinase Cx32, | 0.2019 | -0.6783 | 2.5531 | 0.2813 |
|  |  |  | chloroplastic |  |  |  |  |
| C_2 | Gh_D02G2077 | PUB26 | U-box domain-containing protein 26 | 0.4293 | -0.7902 | 0.8005 | 1.0242 |
| C_2 | Gh_D02G2390 | tatB | Sec-independent protein translocase protein TatB | -0.0120 | -0.4144 | 0.8528 | -0.7219 |
| C_2 | Gh_D03G0041 | CG18812 | Protein GDAP2 homolog | 0.4526 | -1.0032 | -0.8851 | -0.4551 |
| C_2 | Gh_D03G0087 | CPK28 | Calcium-dependent protein kinase 28 | 0.5163 | -0.3188 | 1.3122 | 0.8359 |
| C_2 | Gh_D03G0092 | At3g50940 | AAA-ATPase | 0.1244 | -0.9169 | 0.6893 | -0.0447 |
| C_2 | Gh_D03G0166 | At2g18630 | UPF0496 protein | -0.0759 | -0.0481 | 0.3343 | -0.4218 |
| C_2 | Gh_D03G0175 | BLH2 | BEL1-like homeodomain protein 2 | 0.6061 | -1.5443 | -0.0834 | -0.7211 |
| C_2 | Gh_D03G0209 | GLR3.3 | Glutamate receptor 3.3 | -0.1360 | -1.5526 | 0.1161 | 0.8674 |
| C_2 | Gh_D03G0220 | ERF008 | Ethylene-responsive transcription factor | 0.3872 | -0.5387 | -0.5029 | -0.8761 |
| C_2 | Gh_D03G0278 | PSK3 | Phytosulfokines 3 | 0.3228 | -1.3026 | -0.4536 | -1.0701 |
| C_2 | Gh_D03G0385 | SOT16 | Cytosolic sulfotransferase 16 | 0.3595 | -0.6957 | 1.3089 | -0.1938 |
| C_2 | Gh_D03G0629 | ANS | Leucoanthocyanidin dioxygenase | 0.3366 | -1.6589 | -0.3191 | 0.4702 |
| C_2 | Gh_D03G0632 | H6H | Hyoscyamine 6-dioxygenase | 0.2574 | -1.8955 | -0.3363 | 0.1246 |
| C_2 | Gh_D03G0737 | NA | NA | -0.6968 | -1.1683 | -0.3187 | -0.4089 |
| C_2 | Gh_D03G0926 | UGT89B1 | UDP-glycosyltransferase 89B1 | 0.4861 | -1.1391 | 1.3301 | 0.8527 |
| C_2 | Gh_D03G0928 | UGT89B2 | UDP-glycosyltransferase 89B2 | 0.7146 | -0.6478 | 0.8522 | 0.8914 |
| C_2 | Gh_D03G1042 | NBR1 | Protein NBR1 homolog | 0.1604 | -0.4444 | -0.0230 | -0.2289 |
| C_2 | Gh_D03G1073 | RPL7A | 60S ribosomal protein L7a | 0.4358 | 0.1538 | 1.6353 | 0.2029 |
| C_2 | Gh_D03G1282 | RAP2-7 | Ethylene-responsive transcription factor RAP2-7 | 0.3323 | -0.8725 | -0.1375 | -0.4650 |
| C_2 | Gh_D03G1301 | CNGC15 | Putative cyclic nucleotide-gated ion channel 15 | 0.2892 | 0.2527 | 0.4616 | -0.0433 |
| C_2 | Gh_D03G1361 | LBD12 | LOB domain-containing protein 12 | 1.0693 | -0.6678 | 1.4983 | 1.5250 |
| C_2 | Gh_D03G1513 | CYP93A2 | Cytochrome P450 93A2 | 0.2644 | -0.6718 | 1.1079 | -0.0152 |
| C_2 | Gh_D03G1537 | YLS3 | Protein YLS3 | -0.3309 | -0.9277 | 0.3387 | -0.1637 |
| C_2 | Gh_D03G1671 | At2g23790 | Calcium uniporter protein 2, mitochondrial | -0.2581 | -0.7412 | 0.3415 | -0.3086 |
| C_2 | Gh_D03G1725 | NA | NA | 0.0210 | -0.9246 | 0.7722 | 0.2903 |
| C_2 | Gh_D03G1797 | PDR1 | Pleiotropic drug resistance protein 1 | 2.1273 | 0.3075 | 0.6573 | 0.4058 |
| C_2 | Gh_D04G0112 | WRKY70 | Probable WRKY transcription factor 70 | 1.9305 | 0.6441 | 2.5427 | 0.8302 |
| C_2 | Gh_D04G0264 | PP2B1 | F-box protein PP2-B1 | 0.5529 | -0.7134 | 0.1145 | -0.2413 |
| C_2 | Gh_D04G0271 | NA | NA | 0.3311 | -1.5023 | 1.2832 | -0.2355 |
| C_2 | Gh_D04G0287 | SAUR32 | Auxin-responsive protein SAUR32 | -0.1835 | -0.8201 | 1.0789 | -1.4067 |
| C_2 | Gh_D04G0474 | pvaA | Polyvinylalcohol dehydrogenase | -1.1808 | -2.6975 | 0.1938 | -1.4555 |
| C_2 | Gh_D04G0626 | NA | NA | 0.6462 | 0.0139 | 0.3020 | 0.3555 |
| C_2 | Gh_D04G0704 | VHA-C | V-type proton ATPase subunit C | 0.1553 | -0.2347 | -0.0817 | -0.0868 |
| C_2 | Gh_D04G0971 | SP | Protein SELF-PRUNING | 0.2137 | -1.6684 | -1.7395 | -0.9042 |
| C_2 | Gh_D04G0974 | PAP22 | Purple acid phosphatase 22 | 0.4772 | -0.8393 | 0.6303 | 0.6078 |
| C_2 | Gh_D04G1016 | CYP76A2 | Cytochrome P450 76A2 | 0.2641 | -0.4206 | -0.1555 | -0.1085 |
| C_2 | Gh_D04G1038 | UGT73C5 | UDP-glycosyltransferase 73C5 | 0.8596 | -2.2085 | 1.0005 | -0.1333 |
| C_2 | Gh_D04G1073 | At5g47070 | Probable receptor-like protein kinase | 0.2661 | -0.8782 | 0.5920 | -0.0703 |
| C_2 | Gh_D04G1149 | IP5P8 | Type I inositol polyphosphate 5-phosphatase 8 | 0.4822 | -1.7314 | 0.0026 | -1.3261 |
| C_2 | Gh_D04G1221 | LAC7 | Laccase-7 | 0.8741 | -1.5284 | 0.2049 | 0.3974 |
| C_2 | Gh_D04G1377 | NUDT13 | Nudix hydrolase 13, mitochondrial | 0.6443 | 0.3469 | 1.0595 | -0.4193 |
| C_2 | Gh_D04G1413 | Ephx2 | Bifunctional epoxide hydrolase 2 | 0.4011 | -0.3294 | -0.0906 | -0.3642 |
| C_2 | Gh_D04G1481 | CBR1 | NADH--cytochrome b5 reductase 1 | 1.9555 | -1.9387 | 1.0214 | 0.4003 |
| C_2 | Gh_D04G1584 | TOGT1 | Scopoletin glucosyltransferase | 0.5910 | -0.3301 | 0.1253 | 0.0432 |
| C_2 | Gh_D04G1590 | CNR1 | Cell number regulator 1 | 1.2526 | -1.8285 | 0.9898 | -0.2920 |
| C_2 | Gh_D04G1785 | ACLA-1 | ATP-citrate synthase alpha chain protein 1 | 0.0949 | -0.5067 | 0.0253 | -0.1058 |
| C_2 | Gh_D04G1827 | SPL2 | Squamosa promoter-binding-like protein 2 | -0.3452 | -1.1610 | 0.5109 | -0.1549 |
| C_2 | Gh_D04G1835 | ACD6 | Protein ACCELERATED CELL DEATH 6 | 1.2608 | -1.8318 | 2.5565 | 0.6907 |
| C_2 | Gh_D04G1873 | RDR1 | RNA-dependent RNA polymerase 1 | 0.6367 | 0.0864 | 0.6467 | -0.1255 |
| C_2 | Gh_D04G2010 | UDP-GALT2 | UDP-galactose transporter 2 | 0.4040 | -0.5797 | 1.4075 | 0.4026 |
| C_2 | Gh_D05G0045 | At1g34300 | G-type lectin S-receptor-like serine/threonine-protein kinase | 1.2742 | -2.0092 | 2.6528 | 1.5319 |
| C_2 | Gh_D05G0053 | R1A-6 | Putative late blight resistance protein homolog R1A-6 | 0.5333 | -1.4745 | -1.5046 | -1.7555 |
| C_2 | Gh_D05G0068 | SAMDC | S-adenosylmethionine decarboxylase proenzyme | 0.3139 | -0.2790 | 0.3672 | -0.9852 |
| C_2 | Gh_D05G0109 | Os02g0799000 | Probable protein phosphatase 2C 27 | 0.3918 | -0.6963 | 1.2110 | 0.3466 |
| C_2 | Gh_D05G0124 | MGD2 | Monogalactosyldiacylglycerol synthase 2, chloroplastic | -0.0472 | -0.7187 | -0.5329 | -0.0489 |
| C_2 | Gh_D05G0128 | CRCK3 | Calmodulin-binding receptor-like cytoplasmic kinase 3 | 0.3795 | 0.3429 | 0.9965 | 0.0459 |
| C_2 | Gh_D05G0318 | UGT91C1 | UDP-glycosyltransferase 91C1 | -0.4229 | -1.8434 | -0.6030 | -0.2952 |
| C_2 | Gh_D05G0345 | At5g01020 | Serine/threonine-protein kinase | 0.4233 | -0.1894 | 0.6061 | 0.7480 |
| C_2 | Gh_D05G0520 | CHI3L1 | Chitinase-3-like protein 1 | 2.1274 | -1.2160 | 0.0436 | -1.5993 |
| C_2 | Gh_D05G0523 | ABHD17B | Alpha/beta hydrolase domain-containing protein 17B | 0.4325 | -0.0044 | 0.7671 | 0.3772 |
| C_2 | Gh_D05G0559 | RDR6 | RNA-dependent RNA polymerase 6 | 0.3369 | -1.0119 | 0.4486 | -0.3720 |
| C_2 | Gh_D05G0615 | At5g10820 | Probable folate-biopterin transporter 6 | -0.9619 | -1.8250 | 0.3438 | -0.8394 |
| C_2 | Gh_D05G0637 | GDU1 | Protein GLUTAMINE DUMPER 1 | 0.1559 | -1.1831 | -1.5175 | -1.5628 |
| C_2 | Gh_D05G0886 | BACOVA_02 | Beta-glucosidase BoGH3B | -0.0669 | -1.0041 | -0.1029 | 0.0483 |
|  |  | 659 |  |  |  |  |  |
| C_2 | Gh_D05G0991 | ACA7 | Alpha carbonic anhydrase 7 | 0.0186 | -1.5403 | 1.1334 | -0.1455 |
| C_2 | Gh_D05G1143 | ZFP1 | Zinc finger protein 1 | 0.4230 | -0.2698 | 0.1777 | 0.6306 |
| C_2 | Gh_D05G1359 | Os04g0590900 | E3 ubiquitin-protein ligase | -0.2447 | -2.6909 | 1.4387 | 1.2586 |
| C_2 | Gh_D05G1369 | rnf144ab | Probable E3 ubiquitin-protein ligase RNF144A-B | -0.4133 | -2.3799 | 1.1756 | 0.6869 |
| C_2 | Gh_D05G1417 | CYP710A1 | Cytochrome P450 710A1 | 0.2616 | 0.0036 | 0.9045 | 0.0881 |
| C_2 | Gh_D05G1430 | PUP9 | Probable purine permease 9 | 0.7146 | -0.8498 | 1.2468 | -0.1446 |
| C_2 | Gh_D05G1523 | At5g49770 | Probable leucine-rich repeat receptor-like protein kinase | 0.2826 | -0.6333 | -0.1632 | -0.3050 |
| C_2 | Gh_D05G1578 | Wrap53 | Telomerase Cajal body protein 1 | -0.0418 | -0.7095 | -0.2132 | 0.8304 |
| C_2 | Gh_D05G1643 | PMP22 | Peroxisomal membrane protein PMP22 | -0.0261 | -0.8010 | -0.0360 | 0.1035 |
| C_2 | Gh_D05G1869 | At4g16580 | Probable protein phosphatase 2C 55 | 0.3664 | -0.0878 | 0.0198 | -0.0361 |
| C_2 | Gh_D05G1890 | WAKL14 | Wall-associated receptor kinase-like 14 | 0.1122 | -0.8239 | 0.8468 | -0.2021 |
| C_2 | Gh_D05G1904 | TPP2 | Probable thylakoidal processing peptidase 2, chloroplastic | 0.0871 | -0.4536 | 0.2013 | -0.0270 |
| C_2 | Gh_D05G1955 | SBT1.7 | Subtilisin-like protease SBT1.7 | -0.2597 | -1.8723 | 2.1162 | -1.0642 |
| C_2 | Gh_D05G2064 | RCD1 | Inactive poly [ADP-ribose] polymerase RCD1 | 0.0842 | -0.1749 | 0.1044 | -0.2496 |
| C_2 | Gh_D05G2354 | LAC9 | Putative laccase-9 | 0.1739 | -0.5307 | -0.2572 | -0.3862 |
| C_2 | Gh_D05G2500 | At3g16150 | Probable isoaspartyl peptidase/L-asparaginase 2 | -0.0616 | -1.3792 | 0.4248 | -0.5369 |
| C_2 | Gh_D05G2522 | NA | NA | 0.9973 | 0.1381 | 1.7001 | 1.7643 |
| C_2 | Gh_D05G2589 | LAC14 | Laccase-14 | 0.2620 | -1.5893 | 0.6887 | 0.5531 |
| C_2 | Gh_D05G2642 | WRKY70 | Probable WRKY transcription factor 70 | 0.7168 | -0.7377 | 0.9068 | 0.5788 |
| C_2 | Gh_D05G2685 | PIP5K6 | Phosphatidylinositol 4-phosphate 5-kinase 6 | 0.5140 | -3.7745 | -0.1205 | -0.2375 |
| C_2 | Gh_D05G2756 | GDPDL2 | Glycerophosphodiester phosphodiesterase protein kinase | 0.4960 | -1.7778 | 1.7668 | -0.0928 |
|  |  |  | domain-containing GDPDL2 |  |  |  |  |
| C_2 | Gh_D05G2870 | At2g01680 | Ankyrin repeat-containing protein | 1.4888 | -3.9030 | 0.7397 | -0.3176 |
| C_2 | Gh_D05G2886 | At2g39490 | F-box protein | -1.0562 | -2.7017 | 0.1122 | -0.0290 |
| C_2 | Gh_D05G2906 | UGT76C4 | UDP-glycosyltransferase 76C4 | 0.3528 | -3.6718 | 0.4675 | 1.2875 |
| C_2 | Gh_D05G2960 | NA | NA | 0.4050 | -0.3654 | 0.1634 | 0.0091 |
| C_2 | Gh_D05G3098 | 2MMP | Metalloendoproteinase 2-MMP | -0.1179 | -1.5975 | 1.5418 | 0.6737 |
| C_2 | Gh_D05G3099 | NA | NA | 0.0221 | -1.0853 | 0.9715 | -0.2210 |
| C_2 | Gh_D05G3113 | NA | NA | 0.1220 | -0.3377 | 0.1948 | -0.0897 |
| C_2 | Gh_D05G3139 | NA | NA | 0.3605 | 0.0746 | 0.5333 | 0.3017 |
| C_2 | Gh_D05G3222 | NA | Probable glutathione S-transferase | 1.0041 | -1.0489 | 1.5276 | -0.1097 |
| C_2 | Gh_D05G3373 | At5g63020 | Probable disease resistance protein | -0.0797 | -0.0592 | 0.2690 | -0.3081 |
| C_2 | Gh_D05G3479 | RFL1 | Disease resistance protein RFL1 | 0.1442 | -1.2154 | -0.0739 | -0.5063 |
| C_2 | Gh_D05G3480 | At4g27190 | Disease resistance protein | -0.2918 | -1.9206 | 1.1703 | 1.0673 |
| C_2 | Gh_D05G3533 | NA | NA | -0.9504 | -2.5709 | -0.2491 | 0.0140 |
| C_2 | Gh_D05G3544 | FAX6 | Protein FATTY ACID EXPORT 6 | -0.2013 | -0.6488 | -0.0399 | -0.2046 |
| C_2 | Gh_D05G3548 | At4g27190 | Disease resistance protein | -0.7293 | -1.1655 | -0.4257 | -0.7272 |
| C_2 | Gh_D05G3644 | SCL5 | Scarecrow-like protein 5 | 0.9993 | -1.8289 | 0.6575 | -0.4424 |
| C_2 | Gh_D05G3654 | DIR4 | Dirigent protein 4 | -0.1007 | -1.9153 | -0.0628 | -0.5268 |
| C_2 | Gh_D05G3657 | DIR4 | Dirigent protein 4 | 0.4910 | -1.5488 | 0.2671 | -0.1034 |
| C_2 | Gh_D05G3690 | SFH2 | Phosphatidylinositol/phosphatidylcholine transfer protein | -0.0285 | -0.1824 | 0.1707 | -0.0280 |
|  |  |  | SFH2 |  |  |  |  |
| C_2 | Gh_D05G3813 | grx | Glutaredoxin | 0.1110 | -0.1115 | 0.2265 | -0.1666 |
| C_2 | Gh_D05G3818 | NA | NA | 0.1633 | -1.6490 | 1.1308 | 0.9038 |
| C_2 | Gh_D06G0070 | NAC053 | NAC domain-containing protein 53 | 0.0713 | -0.5016 | -0.0899 | -0.1090 |
| C_2 | Gh_D06G0106 | KO | Ent-kaurene oxidase, chloroplastic | 0.6860 | 0.3722 | 0.4218 | 0.2032 |
| C_2 | Gh_D06G0108 | TPP2 | Probable thylakoidal processing peptidase 2, chloroplastic | 0.3024 | -0.2267 | 0.5315 | -0.0286 |
| C_2 | Gh_D06G0115 | MYB44 | Transcription factor MYB44 | 0.2934 | -0.2930 | 0.2339 | 0.1090 |
| C_2 | Gh_D06G0230 | ITPK3 | Inositol-tetrakisphosphate 1-kinase 3 | 0.6106 | -1.7017 | -0.4877 | -1.1865 |
| C_2 | Gh_D06G0420 | BGAL16 | Beta-galactosidase 16 | 1.2204 | -0.5487 | -0.0654 | 0.2832 |
| C_2 | Gh_D06G0426 | NA | NA | 0.1513 | -0.1137 | -0.0088 | -0.2896 |
| C_2 | Gh_D06G0506 | IAA9 | Auxin-responsive protein IAA9 | -0.1194 | -0.3362 | 0.2165 | -0.0932 |
| C_2 | Gh_D06G0534 | NPF6.2 | Protein NRT1/ PTR FAMILY 6.2 | 0.2928 | -1.6967 | -0.8086 | -0.7016 |
| C_2 | Gh_D06G0580 | ALDH2C4 | Aldehyde dehydrogenase family 2 member C4 | -0.1627 | -1.9703 | 1.2497 | -0.0159 |
| C_2 | Gh_D06G0817 | ERF017 | Ethylene-responsive transcription factor | -0.6662 | -3.0507 | 0.3796 | 1.1807 |
| C_2 | Gh_D06G0856 | FMO1 | Probable flavin-containing monooxygenase 1 | 2.0943 | -0.9936 | -1.0846 | -1.1939 |
| C_2 | Gh_D06G0866 | PME12 | Probable pectinesterase/pectinesterase inhibitor 12 | 0.9651 | -1.0277 | 1.1654 | -0.3950 |
| C_2 | Gh_D06G0885 | CKX7 | Cytokinin dehydrogenase 7 | 1.2323 | -1.6953 | -0.1762 | -0.0504 |
| C_2 | Gh_D06G0938 | At5g26010 | Probable protein phosphatase 2C 72 | -0.2523 | -2.7375 | -1.1566 | -0.3062 |
| C_2 | Gh_D06G0952 | HSL1 | Receptor-like protein kinase HSL1 | 0.8093 | -0.5393 | 1.3136 | 0.9025 |
| C_2 | Gh_D06G1104 | ASPG2 | Protein ASPARTIC PROTEASE IN GUARD CELL 2 | -0.4661 | -1.2649 | -0.6280 | -0.6963 |
| C_2 | Gh_D06G1167 | PSY1 | Phytoene synthase 1, chloroplastic | 0.6442 | -0.8098 | -0.3273 | 0.0569 |
| C_2 | Gh_D06G1337 | STC | Sugar carrier protein C | 0.9792 | 0.9697 | 1.7795 | 0.6110 |
| C_2 | Gh_D06G1340 | NDA1 | Internal alternative NAD(P)H-ubiquinone oxidoreductase | -0.3704 | -1.9580 | 1.6405 | 0.1291 |
|  |  |  | A1, mitochondrial |  |  |  |  |
| C_2 | Gh_D06G1341 | NA | NA | 0.8162 | -1.4231 | 1.4255 | 0.5542 |
| C_2 | Gh_D06G1475 | At4g18930 | Cyclic phosphodiesterase | 1.1609 | -0.6253 | 0.8540 | 0.3176 |
| C_2 | Gh_D06G1546 | JUB1 | Transcription factor JUNGBRUNNEN 1 | 0.3595 | -1.4492 | 1.8187 | 1.7157 |
| C_2 | Gh_D06G1685 | At2g25060 | Early nodulin-like protein 1 | 0.4919 | -0.7737 | -0.2725 | 0.1217 |
| C_2 | Gh_D06G1954 | At4g27290 | G-type lectin S-receptor-like serine/threonine-protein | 0.6055 | -2.1544 | 0.3183 | 0.6709 |
|  |  |  | kinase |  |  |  |  |
| C_2 | Gh_D06G2007 | BOR4 | Boron transporter 4 | 0.2101 | -1.8983 | 0.1431 | -1.4918 |
| C_2 | Gh_D06G2248 | PUB33 | U-box domain-containing protein 33 | 1.0141 | -0.4983 | 0.5866 | 0.1177 |
| C_2 | Gh_D06G2290 | BRG3 | Probable BOI-related E3 ubiquitin-protein ligase 3 | 1.6994 | -1.2748 | 1.6611 | 1.3538 |
| C_2 | Gh_D06G2328 | WRKY50 | Probable WRKY transcription factor 50 | 0.3516 | -2.3670 | 0.5040 | 0.0887 |
| C_2 | Gh_D07G0070 | BSPA | Bark storage protein A | -0.1769 | -0.6909 | -0.1444 | -0.2092 |
| C_2 | Gh_D07G0071 | XI-H | Myosin-14 | 0.0739 | -0.2929 | 0.3734 | -0.3777 |
| C_2 | Gh_D07G0159 | At5g01020 | Serine/threonine-protein kinase | 0.4127 | -1.1784 | -0.4065 | -0.4626 |
| C_2 | Gh_D07G0177 | WRKY50 | Probable WRKY transcription factor 50 | 2.8882 | 0.5111 | 3.4074 | 2.5292 |
| C_2 | Gh_D07G0212 | HSL1 | Receptor-like protein kinase HSL1 | 0.0309 | -1.0350 | 0.3427 | -0.4681 |
| C_2 | Gh_D07G0217 | CSE | Caffeoylshikimate esterase | 0.1178 | -1.5564 | 1.3464 | 0.6273 |
| C_2 | Gh_D07G0246 | CHIA | Acidic mammalian chitinase | 0.2309 | -0.9708 | 0.0591 | -1.6978 |
| C_2 | Gh_D07G0247 | At1g11330 | G-type lectin S-receptor-like serine/threonine-protein kinase | 0.4117 | -1.4865 | 0.6859 | 0.9149 |
| C_2 | Gh_D07G0796 | NA | NA | -0.5448 | -3.0410 | -2.2560 | -0.7089 |
| C_2 | Gh_D07G0802 | JUB1 | Transcription factor JUNGBRUNNEN 1 | 0.5831 | -1.5590 | -0.0004 | -0.4300 |
| C_2 | Gh_D07G0841 | ACO3 | 1-aminocyclopropane-1-carboxylate oxidase 3 | 0.4370 | -1.4106 | 1.5651 | -0.6824 |
| C_2 | Gh_D07G0857 | LACS8 | Long chain acyl-CoA synthetase 8 | -0.0454 | -0.5153 | 0.0859 | -0.5822 |
| C_2 | Gh_D07G0881 | GLR1.2 | Glutamate receptor 1.2 | -0.1642 | -2.1791 | 1.0495 | 0.3232 |
| C_2 | Gh_D07G0896 | NA | NA | 0.7797 | -3.0142 | -0.2369 | -0.5848 |
| C_2 | Gh_D07G0899 | At3g25210 | Pentatricopeptide repeat-containing protein | 0.1620 | -0.5796 | 0.1000 | -1.0123 |
| C_2 | Gh_D07G1043 | NA | NA | 0.6023 | -0.3426 | -0.0512 | 0.1834 |
| C_2 | Gh_D07G1238 | NA | NA | 0.9592 | -1.4993 | 1.1765 | 0.8274 |
| C_2 | Gh_D07G1422 | PHT4 | Ascorbate transporter, chloroplastic | 0.4570 | -0.0420 | 0.1524 | 0.4558 |
| C_2 | Gh_D07G1461 | AHL22 | AT-hook motif nuclear-localized protein 22 | -0.0987 | -0.2536 | 0.0236 | -0.0909 |
| C_2 | Gh_D07G1519 | RPL23A | 60S ribosomal protein L23 | 0.5174 | 0.1172 | 0.6981 | 0.3283 |
| C_2 | Gh_D07G1562 | VAMP726 | Putative vesicle-associated membrane protein 726 | -0.3510 | -0.7795 | 0.4442 | -0.4374 |
| C_2 | Gh_D07G1923 | At5g35735 | Cytochrome b561 and DOMON domain-containing | 0.3000 | -1.4743 | 1.3698 | 0.9292 |
|  |  |  | protein |  |  |  |  |
| C_2 | Gh_D07G1946 | NUDT2 | Nudix hydrolase 2 | 1.4395 | -1.3585 | 2.7011 | 0.3671 |
| C_2 | Gh_D07G1983 | GLR1.3 | Glutamate receptor 1.3 | 0.1581 | -1.1789 | 1.6210 | 0.5280 |
| C_2 | Gh_D07G2009 | At4g13010 | Putative quinone-oxidoreductase homolog, chloroplastic | 0.1270 | -0.8920 | 1.1037 | -0.5397 |
| C_2 | Gh_D07G2010 | QOR | Quinone-oxidoreductase homolog, chloroplastic | 0.5669 | -0.4813 | 0.8971 | -0.3900 |
| C_2 | Gh_D07G2014 | NAC091 | NAC domain-containing protein 91 | 0.4164 | -2.2663 | 2.2313 | 0.8507 |
| C_2 | Gh_D07G2060 | NA | Glutathione transferase GST 23 | -0.1806 | -1.6394 | 1.1660 | 0.2646 |
| C_2 | Gh_D07G2099 | CGS1 | Cystathionine gamma-synthase 1, chloroplastic | -0.3359 | -1.1646 | 0.7661 | -0.7509 |
| C_2 | Gh_D07G2154 | PHT1-7 | Probable inorganic phosphate transporter 1-7 | 0.6450 | -1.3716 | 0.3002 | -0.4193 |
| C_2 | Gh_D07G2196 | pvaA | Polyvinylalcohol dehydrogenase | 0.0780 | -1.2421 | -0.9783 | -0.8814 |
| C_2 | Gh_D07G2197 | pvaA | Polyvinylalcohol dehydrogenase | -0.2665 | -1.2778 | -0.9507 | -0.5610 |
| C_2 | Gh_D07G2239 | At1g23740 | Quinone oxidoreductase-like protein | 0.0148 | -0.7984 | 1.5974 | -0.7582 |
| C_2 | Gh_D07G2297 | LHT1 | Lysine histidine transporter 1 | 0.7139 | 0.0642 | 0.2335 | 1.0829 |
| C_2 | Gh_D07G2376 | ALE2 | Receptor-like serine/threonine-protein kinase ALE2 | 0.2246 | -0.4198 | 1.4923 | 0.4899 |
| C_2 | Gh_D07G2435 | COP1 | E3 ubiquitin-protein ligase COP1 | -0.4869 | -1.2928 | -0.3032 | 0.0200 |
| C_2 | Gh_D08G0030 | At5g02620 | Ankyrin repeat-containing protein | 0.3798 | -0.5890 | 0.9048 | 0.3039 |
| C_2 | Gh_D08G0177 | At2g37990 | Ribosome biogenesis regulatory protein homolog | -0.0069 | -1.3745 | -0.8943 | -1.4509 |
| C_2 | Gh_D08G0432 | AAE11 | Butyrate--CoA ligase AAE11, peroxisomal | 1.7533 | -1.3271 | 2.5513 | 0.1119 |
| C_2 | Gh_D08G0476 | NA | NA | 1.1334 | -1.4164 | 4.0940 | 1.5854 |
| C_2 | Gh_D08G0480 | GSO1 | LRR receptor-like serine/threonine-protein kinase GSO1 | 0.1344 | -0.7178 | -0.2229 | -0.2525 |
| C_2 | Gh_D08G0606 | ATL4 | E3 ubiquitin-protein ligase | -0.6556 | -4.1545 | 0.3414 | 0.7054 |
| C_2 | Gh_D08G0635 | FBX5 | Protein ARABIDILLO 1 | 0.0980 | -0.5105 | 0.3338 | 0.6460 |
| C_2 | Gh_D08G0925 | At3g15890 | PTI1-like tyrosine-protein kinase | 0.2719 | -1.8419 | -0.3441 | 0.3602 |
| C_2 | Gh_D08G0989 | NUB1 | NEDD8 ultimate buster 1 | 0.2074 | -0.7822 | -0.1338 | -0.3874 |
| C_2 | Gh_D08G1192 | BIP5 | Luminal-binding protein 5 | -0.1295 | -0.8866 | -0.0747 | -0.3366 |
| C_2 | Gh_D08G1325 | COX5C | Cytochrome c oxidase subunit 5C | -0.0628 | -0.1274 | 0.0060 | -0.0503 |
| C_2 | Gh_D08G1407 | ARF3 | Auxin response factor 3 | 0.2047 | -0.5135 | 0.6641 | 0.1877 |
| C_2 | Gh_D08G1506 | SAUR36 | Auxin-responsive protein | 0.7367 | 0.1100 | 1.7738 | 0.3243 |
| C_2 | Gh_D08G1885 | ACR8 | ACT domain-containing protein | 0.1885 | -0.2565 | 0.9742 | 0.0687 |
| C_2 | Gh_D08G1981 | GID1B | Gibberellin receptor GID1B | -0.1730 | -1.5557 | -0.6387 | -0.4568 |
| C_2 | Gh_D08G2272 | AKR1 | Probable aldo-keto reductase 1 | 0.2676 | -0.4753 | 0.4064 | -0.0892 |
| C_2 | Gh_D08G2316 | NA | Caffeic acid 3-O-methyltransferase | 1.4218 | 0.1348 | 2.4797 | -1.2485 |
| C_2 | Gh_D08G2534 | EMB1027 | Arginine--tRNA ligase, chloroplastic/mitochondrial | 0.2081 | -0.5281 | -0.1027 | 0.0068 |
| C_2 | Gh_D08G2575 | CID7 | Polyadenylate-binding protein-interacting protein 7 | 0.0907 | -0.5123 | -0.4821 | -0.4944 |
| C_2 | Gh_D08G2577 | CKX7 | Cytokinin dehydrogenase 7 | 3.0930 | -0.8776 | -0.7607 | -1.4228 |
| C_2 | Gh_D08G2578 | RBP1 | RNA-binding protein 1 | -0.0845 | -0.2152 | -0.1419 | -0.3477 |
| C_2 | Gh_D08G2671 | JUB1 | Transcription factor JUNGBRUNNEN 1 | -0.3930 | -1.6095 | 0.8987 | 0.4120 |
| C_2 | Gh_D08G2680 | F6'H1 | Feruloyl CoA ortho-hydroxylase 1 | 0.0069 | -1.5240 | 0.8745 | 1.0799 |
| C_2 | Gh_D08G2692 | SIGA | RNA polymerase sigma factor sigA | 0.0983 | -0.3644 | 0.8127 | -0.6777 |
| C_2 | Gh_D08G2695 | CYP89A2 | Cytochrome P450 89A2 | 0.9674 | -2.9526 | 0.1376 | -1.1478 |
| C_2 | Gh_D08G2765 | IQM2 | IQ domain-containing protein IQM2 | 0.8658 | -0.1048 | 0.3974 | 0.6819 |
| C_2 | Gh_D09G0036 | pof4 | Elongin-A | 0.0259 | -0.2262 | -0.1400 | -0.0972 |
| C_2 | Gh_D09G0064 | NA | NA | -0.4725 | -0.6470 | 0.6799 | -1.1981 |
| C_2 | Gh_D09G0108 | NA | NA | 1.6572 | -0.7438 | -0.3459 | 0.1231 |
| C_2 | Gh_D09G0219 | AATP1 | AAA-ATPase ASD, mitochondrial | 1.3658 | -1.5226 | -0.6885 | 0.5077 |
| C_2 | Gh_D09G0229 | SNL2 | Paired amphipathic helix protein Sin3-like 2 | 0.4903 | 0.3198 | 2.8632 | -1.2727 |
| C_2 | Gh_D09G0319 | MYB315 | Myb-related protein 315 | 0.1293 | -1.0309 | -1.2903 | -1.1690 |
| C_2 | Gh_D09G0337 | LHT1 | Lysine histidine transporter 1 | 0.0895 | -0.2496 | -0.2439 | -0.2950 |
| C_2 | Gh_D09G0356 | At4g15970 | Uncharacterized protein | 0.5790 | 0.2518 | 1.0606 | 0.1751 |
| C_2 | Gh_D09G0363 | TOM20 | Mitochondrial import receptor subunit | -0.0132 | -0.6399 | -0.5308 | 0.1332 |
| C_2 | Gh_D09G0489 | SARD1 | Protein SAR DEFICIENT 1 | -0.0208 | -0.9216 | 0.9032 | 0.4882 |
| C_2 | Gh_D09G0497 | NFXL1 | NF-X1-type zinc finger protein NFXL1 | 0.5814 | -0.4444 | 1.2070 | 0.0503 |
| C_2 | Gh_D09G0639 | At3g01520 | Universal stress protein A-like protein | 0.1880 | -0.1266 | -0.0777 | -0.0231 |
| C_2 | Gh_D09G0678 | CRK26 | Cysteine-rich receptor-like protein kinase 26 | 0.1892 | -1.6731 | 0.3213 | 0.7077 |
| C_2 | Gh_D09G0697 | NA | Polyphenol oxidase, chloroplastic | 0.4587 | -1.2942 | -0.2237 | -0.1444 |
| C_2 | Gh_D09G0737 | NPR3 | Regulatory protein NPR3 | 0.3591 | -0.2673 | 0.3232 | -0.1705 |
| C_2 | Gh_D09G0742 | NA | Peroxisomal (S)-2-hydroxy-acid oxidase | -0.2590 | -1.2205 | -0.9290 | -0.4968 |
| C_2 | Gh_D09G0832 | NA | Pyrophosphate-energized vacuolar membrane proton | -0.3763 | -1.2145 | -0.3410 | -0.3521 |
|  |  |  | pump |  |  |  |  |
| C_2 | Gh_D09G0841 | At1g65240 | Aspartic proteinase-like protein 2 | -0.1739 | -0.8716 | 0.3525 | -0.5107 |
| C_2 | Gh_D09G0982 | CXE15 | Probable carboxylesterase 15 | 1.0300 | -0.7635 | -0.2794 | -0.5211 |
| C_2 | Gh_D09G1007 | pitA | Phosphatidylinositol transfer protein 1 | 0.9211 | 0.2014 | 1.8193 | 0.0946 |
| C_2 | Gh_D09G1021 | APK2B | Protein kinase 2B, chloroplastic | -0.0078 | -1.3329 | 1.2879 | 0.1482 |
| C_2 | Gh_D09G1045 | LRP1 | Protein LATERAL ROOT PRIMORDIUM 1 | 1.2238 | -0.3621 | 0.2476 | 0.3726 |
| C_2 | Gh_D09G1139 | FLOT1 | Flotillin-like protein 1 | 0.5043 | -1.4818 | 1.0146 | 0.6804 |
| C_2 | Gh_D09G1253 | NA | NA | 0.1014 | -0.2965 | 0.2832 | -0.2255 |
| C_2 | Gh_D09G1412 | NA | Putative serine/threonine-protein kinase (Fragment) | 0.6636 | -0.1964 | 1.0802 | -0.2626 |
| C_2 | Gh_D09G1511 | GSTL3 | Glutathione S-transferase L3 | -0.1070 | -1.0987 | 0.0358 | -0.7334 |
| C_2 | Gh_D09G1521 | GSTU8 | Glutathione S-transferase U8 | -0.0791 | -1.7936 | 0.7548 | 0.3836 |
| C_2 | Gh_D09G1568 | LYSRS | Lysine--tRNA ligase | 0.0947 | -1.9985 | 0.1444 | -0.4526 |
| C_2 | Gh_D09G1604 | Gtpbp4 | Nucleolar GTP-binding protein 1 | -0.3485 | -0.4252 | -0.1678 | -0.4831 |
| C_2 | Gh_D09G1675 | At1g67000 | Probable receptor-like protein kinase | 0.7509 | 0.1757 | 0.5949 | 0.8024 |
| C_2 | Gh_D09G1687 | ASPG1 | Protein ASPARTIC PROTEASE IN GUARD CELL 1 | -0.0183 | -1.8551 | -0.8483 | 0.2714 |
| C_2 | Gh_D09G1784 | NA | NA | 0.8915 | -1.6395 | 0.8440 | 0.0566 |
| C_2 | Gh_D09G1823 | NA | NA | 0.5467 | -1.3218 | -0.2946 | -0.1521 |
| C_2 | Gh_D09G1835 | CPK17 | Calcium-dependent protein kinase 17 | 0.3303 | -0.1919 | 0.2922 | 0.4446 |
| C_2 | Gh_D09G1940 | NA | Malate dehydrogenase, cytoplasmic | 0.9863 | -0.4727 | 0.2121 | -0.7311 |
| C_2 | Gh_D09G1968 | NA | Cytochrome P450 CYP736A12 | -0.7731 | -2.6013 | -0.3210 | -0.3987 |
| C_2 | Gh_D09G1969 | NA | Cytochrome P450 CYP736A12 | 0.0520 | -0.9270 | -0.9765 | -0.9800 |
| C_2 | Gh_D09G1999 | UGT89C1 | UDP-glycosyltransferase 89C1 | 1.0709 | 0.1349 | 4.3939 | -2.8301 |
| C_2 | Gh_D09G2019 | EP3 | Endochitinase EP3 | -0.1026 | -1.5837 | 0.4156 | 0.5785 |
| C_2 | Gh_D09G2020 | EP3 | Endochitinase EP3 | 0.5999 | -1.4838 | 1.6304 | 0.1699 |
| C_2 | Gh_D09G2051 | NA | Non-specific lipid-transfer protein 3 | 0.6483 | -0.9690 | 4.8722 | -3.8461 |
| C_2 | Gh_D09G2067 | FAF3 | Protein FANTASTIC FOUR 3 | 0.1248 | 0.0154 | 0.9551 | -0.1634 |
| C_2 | Gh_D09G2169 | ETR2 | Ethylene receptor 2 | 0.1309 | -0.1705 | 0.0765 | -0.2407 |
| C_2 | Gh_D09G2347 | NA | NA | 0.7588 | -2.2018 | 0.6221 | -0.4336 |
| C_2 | Gh_D09G2385 | 4CLL9 | 4-coumarate--CoA ligase-like 9 | 0.0847 | -0.2565 | 0.1259 | 0.3105 |
| C_2 | Gh_D09G2395 | At1g54610 | Probable serine/threonine-protein kinase | 0.9811 | -3.3252 | 2.1797 | 0.7844 |
| C_2 | Gh_D09G2481 | At1g18390 | Probable serine/threonine-protein kinase | -0.2362 | -0.9579 | 0.2809 | -0.2352 |
| C_2 | Gh_D10G0110 | MLYCD | Malonyl-CoA decarboxylase, mitochondrial | -0.1677 | -0.8914 | -0.2666 | -0.4508 |
| C_2 | Gh_D10G0120 | BCAP31 | B-cell receptor-associated protein 31 | 0.3604 | 0.0243 | 1.1214 | 0.0690 |
| C_2 | Gh_D10G0190 | STC | Sugar carrier protein C | 0.1598 | -0.8335 | 0.6286 | 0.0730 |
| C_2 | Gh_D10G0440 | ZIP10 | Probable zinc transporter 10 | 1.1795 | 0.1934 | 0.9052 | 0.3806 |
| C_2 | Gh_D10G0480 | NA | NA | 0.2745 | -0.2120 | 0.2178 | 0.0134 |
| C_2 | Gh_D10G0526 | NA | NA | 0.1373 | 0.1496 | 0.6977 | -0.1593 |
| C_2 | Gh_D10G0628 | ABCG36 | ABC transporter G family member 36 | 0.4362 | -0.5858 | 0.7772 | -0.2316 |
| C_2 | Gh_D10G0647 | ACOT13 | Acyl-coenzyme A thioesterase 13 | 0.0597 | -0.5932 | 0.3196 | 0.2864 |
| C_2 | Gh_D10G0667 | CES101 | G-type lectin S-receptor-like serine/threonine-protein | 0.5262 | -3.7452 | 1.8537 | 0.9942 |
|  |  |  | kinase CES101 |  |  |  |  |
| C_2 | Gh_D10G0681 | At1g80440 | F-box/kelch-repeat protein | 1.8610 | 1.5112 | 1.9705 | 0.9939 |
| C_2 | Gh_D10G0732 | NA | NA | 0.2008 | -1.1796 | 1.3545 | 0.1811 |
| C_2 | Gh_D10G0748 | HSR4 | Protein HYPER-SENSITIVITY-RELATED 4 | -0.7686 | -4.0207 | 1.4574 | 0.7091 |
| C_2 | Gh_D10G0774 | EXO70B1 | Exocyst complex component EXO70B1 | 0.6714 | -2.5683 | 0.8086 | -0.1459 |
| C_2 | Gh_D10G0847 | HAT5 | Homeobox-leucine zipper protein HAT5 | 0.3698 | -1.2504 | -0.0885 | -0.4966 |
| C_2 | Gh_D10G0926 | PLP1 | Patatin-like protein 1 | 0.1542 | -1.3466 | 0.4310 | 0.4209 |
| C_2 | Gh_D10G0943 | NA | NA | 0.4844 | -1.4725 | 1.3135 | 2.0331 |
| C_2 | Gh_D10G0973 | NA | NA | -1.0042 | -4.2824 | -1.3048 | -1.0395 |
| C_2 | Gh_D10G1229 | Tbc1d15 | TBC1 domain family member 15 | 0.1700 | -0.7571 | 0.8102 | 0.0579 |
| C_2 | Gh_D10G1314 | Os04g0338000 | Probable aldo-keto reductase 2 | -0.2023 | -0.7446 | -0.3799 | -0.1130 |
| C_2 | Gh_D10G1372 | PHT1-5 | Probable inorganic phosphate transporter 1-5 | 0.8849 | -1.0765 | -0.3599 | -0.3449 |
| C_2 | Gh_D10G1453 | NA | Probable glutathione S-transferase | -0.3321 | -2.0213 | -0.4269 | -0.7647 |
| C_2 | Gh_D10G1521 | NA | NA | -0.4198 | -0.6595 | 0.6274 | -0.7943 |
| C_2 | Gh_D10G1543 | PPCK1 | Phosphoenolpyruvate carboxylase kinase 1 | -0.0536 | -1.9868 | 1.9206 | 0.8235 |
| C_2 | Gh_D10G1603 | AMT1-1 | Ammonium transporter 1 member 1 | 0.1820 | -0.9410 | -0.6205 | -0.3317 |
| C_2 | Gh_D10G1739 | NA | Major allergen Pru ar 1 | 1.2474 | -1.4108 | 0.3988 | -0.3670 |
| C_2 | Gh_D10G1752 | NA | Major allergen Pru ar 1 | 0.5015 | -0.7879 | 0.6886 | -0.0829 |
| C_2 | Gh_D10G1753 | BETV1F | Major pollen allergen Bet v 1-F/I | -0.1138 | -1.2528 | 0.4590 | 1.0907 |
| C_2 | Gh_D10G1754 | BETV1E | Major pollen allergen Bet v 1-E | 0.2691 | -1.6200 | 0.6580 | 0.0320 |
| C_2 | Gh_D10G1756 | NA | Major allergen Pru ar 1 | -0.5333 | -0.8807 | -0.8076 | -0.7494 |
| C_2 | Gh_D10G1762 | BETVIA | Major pollen allergen Bet v 1-A | -0.1765 | -1.3900 | 0.0879 | -0.2361 |
| C_2 | Gh_D10G1811 | ACT | Vinorine synthase | 0.8454 | -0.7065 | 1.9323 | 0.4703 |
| C_2 | Gh_D10G1840 | Y-1 | Uncharacterized protein | 0.3340 | -1.7293 | 0.7975 | -0.2511 |
| C_2 | Gh_D10G1887 | NA | NA | 0.7603 | -2.7354 | 3.2719 | 2.1595 |
| C_2 | Gh_D10G1977 | ELC | Protein ELC | 0.1565 | -0.0467 | 0.5105 | -0.1549 |
| C_2 | Gh_D10G2050 | CYP714B2 | Cytochrome P450 714B2 | 0.4586 | -0.8963 | 0.5520 | 0.2579 |
| C_2 | Gh_D10G2215 | ZAT11 | Zinc finger protein ZAT11 | -0.5434 | -1.5589 | 0.1083 | -0.6161 |
| C_2 | Gh_D10G2237 | NA | NA | -1.0226 | -4.0152 | 1.5835 | 1.1208 |
| C_2 | Gh_D10G2242 | ECR | Very-long-chain enoyl-CoA reductase | 0.3565 | 0.1951 | 0.4936 | -0.1552 |
| C_2 | Gh_D10G2369 | At3g47570 | Probable LRR receptor-like serine/threonine-protein | -0.4205 | -1.9216 | -1.3810 | -3.2501 |
|  |  |  | kinase |  |  |  |  |
| C_2 | Gh_D10G2371 | At5g61250 | Heparanase-like protein 2 | -0.3349 | -3.1793 | -1.1736 | -1.2032 |
| C_2 | Gh_D10G2375 | At5g61250 | Heparanase-like protein 2 | -0.0087 | -2.9430 | -0.2229 | -0.2525 |
| C_2 | Gh_D10G2414 | CAN2 | Staphylococcal-like nuclease CAN2 | 0.3839 | -2.3005 | 0.3256 | -0.4655 |
| C_2 | Gh_D11G0151 | GYP7 | GTPase-activating protein GYP7 | 0.0550 | -0.1821 | 0.2888 | -0.1407 |
| C_2 | Gh_D11G0313 | At1g74750 | Pentatricopeptide repeat-containing protein | 0.2019 | -0.9482 | -0.1761 | -0.5257 |
| C_2 | Gh_D11G0323 | ABCA2 | ABC transporter A family member 2 | 0.4522 | -0.7929 | 0.8539 | 0.0272 |
| C_2 | Gh_D11G0450 | RPL8 | 60S ribosomal protein L8 | 0.6692 | -0.0640 | 0.0095 | 0.2065 |
| C_2 | Gh_D11G0463 | PER72 | Peroxidase 72 | 0.4372 | -0.4594 | 0.2186 | -0.4536 |
| C_2 | Gh_D11G0526 | HAT22 | Homeobox-leucine zipper protein | 0.0406 | -1.2491 | 0.6080 | 0.5269 |
| C_2 | Gh_D11G0610 | csd | Probable cysteine desulfurase | 0.7746 | 0.2709 | 1.3083 | -0.5556 |
| C_2 | Gh_D11G0840 | At1g62620 | Flavin-containing monooxygenase FMO GS-OX-like 3 | 0.4271 | -1.3965 | 0.3579 | -0.2128 |
| C_2 | Gh_D11G0845 | CRRSP15 | Cysteine-rich repeat secretory protein 15 | -0.6915 | -1.8022 | -0.8245 | -0.1492 |
| C_2 | Gh_D11G0915 | NA | NA | 0.0859 | -0.0514 | 0.1271 | -0.2165 |
| C_2 | Gh_D11G1235 | NA | Secoisolariciresinol dehydrogenase (Fragment) | 0.3737 | -1.1357 | 0.0425 | 0.0866 |
| C_2 | Gh_D11G1391 | NA | Glutathione transferase GST 23 | 0.2928 | -2.2208 | 0.8835 | -0.1845 |
| C_2 | Gh_D11G1438 | nxt3 | Putative G3BP-like protein | 0.1317 | 0.0503 | 0.1253 | 0.0983 |
| C_2 | Gh_D11G1515 | NA | NA | 1.7372 | -1.4496 | 2.0398 | -0.5319 |
| C_2 | Gh_D11G1623 | FER | Receptor-like protein kinase FERONIA | 0.0174 | -1.5855 | 0.6179 | 0.2982 |
| C_2 | Gh_D11G1691 | At1g67000 | Probable receptor-like protein kinase | -0.1494 | -1.7776 | 1.5548 | 0.4706 |
| C_2 | Gh_D11G1702 | NA | NA | -0.1343 | -2.2073 | -0.3729 | -0.7784 |
| C_2 | Gh_D11G1785 | Os09g0533900 | Endoglucanase 24 | -0.3657 | -2.3009 | 0.7548 | 1.4492 |
| C_2 | Gh_D11G1848 | NA | Putative glucose-6-phosphate 1-epimerase | 0.7851 | -0.4369 | -0.3299 | -0.1611 |
| C_2 | Gh_D11G1866 | At5g49610 | F-box protein | 0.4041 | -1.2980 | 1.0135 | 0.1197 |
| C_2 | Gh_D11G2039 | HHP4 | Heptahelical transmembrane protein 4 | 0.3182 | -0.5953 | -0.2395 | -0.3274 |
| C_2 | Gh_D11G2048 | EMB2001 | GTP-binding protein | -0.1143 | -1.5805 | 0.1811 | -0.1075 |
| C_2 | Gh_D11G2094 | LECRK91 | L-type lectin-domain containing receptor kinase IX.1 | 0.1377 | -1.9989 | -0.6021 | 0.4094 |
| C_2 | Gh_D11G2117 | PAS2 | Very-long-chain (3R)-3-hydroxyacyl-CoA dehydratase | 0.5909 | -0.1940 | 0.2346 | -0.4180 |
|  |  |  | PASTICCINO 2 |  |  |  |  |
| C_2 | Gh_D11G2183 | PER53 | Peroxidase 53 | -0.0429 | -1.1683 | 0.3556 | 0.5984 |
| C_2 | Gh_D11G2307 | LYK5 | Protein LYK5 | 0.2753 | -0.4348 | 0.7021 | 0.0973 |
| C_2 | Gh_D11G2413 | CSLE6 | Cellulose synthase-like protein E6 | 0.1657 | 0.0578 | 0.1856 | -0.1151 |
| C_2 | Gh_D11G2446 | NA | NA | -0.3569 | -1.3086 | -0.1711 | -0.7879 |
| C_2 | Gh_D11G2510 | TUBB2 | Tubulin beta-2 chain (Fragment) | 0.2446 | -0.1521 | 0.1893 | -0.2776 |
| C_2 | Gh_D11G2600 | AKR1 | Probable aldo-keto reductase 1 | -0.0850 | -0.5132 | -0.2121 | -0.3920 |
| C_2 | Gh_D11G2602 | AKR1 | Probable aldo-keto reductase 1 | 0.2559 | -0.4523 | 0.1621 | -0.0213 |
| C_2 | Gh_D11G2603 | AKR1 | Probable aldo-keto reductase 1 | 0.2222 | -0.4836 | 0.4004 | 0.1918 |
| C_2 | Gh_D11G2702 | ATL6 | E3 ubiquitin-protein ligase ATL6 | -0.4816 | -1.7711 | 0.6596 | -0.0526 |
| C_2 | Gh_D11G2748 | At3g05500 | REF/SRPP-like protein | 0.0416 | -0.3817 | 0.0003 | -0.2069 |
| C_2 | Gh_D11G2775 | PLPZETA2 | Phospholipase D zeta 2 | 0.2175 | -0.7640 | -0.6563 | -0.5645 |
| C_2 | Gh_D11G2850 | ABCC3 | ABC transporter C family member 3 | 0.2329 | -3.1816 | 2.8053 | 0.7497 |
| C_2 | Gh_D11G2852 | ABCC3 | ABC transporter C family member 3 | 0.3420 | -3.6151 | 0.7199 | -0.8401 |
| C_2 | Gh_D11G2853 | ABCC3 | ABC transporter C family member 3 | 0.3910 | -4.1242 | 1.0139 | -0.8050 |
| C_2 | Gh_D11G2924 | N | TMV resistance protein N | 0.6065 | 0.4853 | 0.8386 | 0.3620 |
| C_2 | Gh_D11G2925 | NA | NA | 0.3226 | -0.0362 | 1.2569 | -0.1019 |
| C_2 | Gh_D11G2984 | CALS8 | Putative callose synthase 8 | -0.1365 | -0.6732 | -0.1509 | -0.2806 |
| C_2 | Gh_D11G3059 | NCS2 | S-norcoclaurine synthase 2 | 2.2148 | -1.8660 | -1.1517 | 3.3382 |
| C_2 | Gh_D11G3160 | NA | NA | -0.4783 | -0.9592 | -0.2700 | -0.2504 |
| C_2 | Gh_D11G3192 | RGA4 | Putative disease resistance protein RGA4 | 0.8906 | -1.2761 | -0.9089 | -0.9615 |
| C_2 | Gh_D11G3212 | UEV1C | Ubiquitin-conjugating enzyme E2 variant 1C | 0.2423 | -0.4199 | -0.3438 | -0.0846 |
| C_2 | Gh_D11G3247 | IQM3 | IQ domain-containing protein IQM3 | 1.4692 | -0.1789 | 1.5668 | 0.1433 |
| C_2 | Gh_D11G3352 | CAMBP25 | Calmodulin-binding protein 25 | -0.1789 | -1.0746 | 0.2428 | -0.1083 |
| C_2 | Gh_D11G3379 | DIR15 | Dirigent protein 15 | 1.9192 | -2.0814 | -1.4598 | 1.9403 |
| C_2 | Gh_D11G3493 | HACL | 2-hydroxyacyl-CoA lyase | -0.1511 | -0.6635 | 0.1192 | 0.0220 |
| C_2 | Gh_D11G3531 | QKY | Protein QUIRKY | 0.4611 | -0.2874 | 0.0829 | 0.0702 |
| C_2 | Gh_D12G0006 | NA | ATP synthase subunit epsilon, mitochondrial | 0.4493 | -0.1105 | 0.2708 | -0.0624 |
| C_2 | Gh_D12G0043 | FAF1 | Protein FANTASTIC FOUR 1 | -0.1034 | -1.1091 | 1.5148 | -0.2976 |
| C_2 | Gh_D12G0070 | PER3 | Peroxidase 3 | 2.5146 | -0.3941 | 3.7276 | 4.2824 |
| C_2 | Gh_D12G0077 | COL13 | Zinc finger protein CONSTANS-LIKE 13 | -0.0490 | -1.6568 | 1.6948 | 0.1050 |
| C_2 | Gh_D12G0090 | POT5 | Potassium transporter 5 | -0.9538 | -2.3284 | -0.6495 | -0.4783 |
| C_2 | Gh_D12G0113 | LECRKS5 | Probable L-type lectin-domain containing receptor kinase | 0.6976 | -2.0021 | 0.1002 | -0.8591 |
|  |  |  | S.5 |  |  |  |  |
| C_2 | Gh_D12G0115 | HMG1 | 3-hydroxy-3-methylglutaryl-coenzyme A reductase 1 | -0.2094 | -1.1650 | 1.0320 | 0.2837 |
| C_2 | Gh_D12G0146 | yrpB | Probable nitronate monooxygenase | 0.3307 | -0.9634 | 0.3314 | 0.2113 |
| C_2 | Gh_D12G0150 | MCM9 | Probable DNA helicase MCM9 | 0.4688 | -0.3155 | 0.4326 | -0.1671 |
| C_2 | Gh_D12G0234 | MES3 | Methylesterase 3 | -0.2326 | -1.8372 | -0.8770 | -0.8004 |
| C_2 | Gh_D12G0326 | At3g58360 | MATH domain and coiled-coil domain-containing protein | 0.1376 | -0.0641 | 0.2394 | -0.4100 |
| C_2 | Gh_D12G0418 | ABCC12 | ABC transporter C family member 12 | -0.2174 | -0.5811 | 0.2562 | -0.2246 |
| C_2 | Gh_D12G0420 | ABCC12 | ABC transporter C family member 12 | 0.2438 | 0.0510 | 0.1975 | -0.1996 |
| C_2 | Gh_D12G0421 | ABCC2 | ABC transporter C family member 2 | 0.3526 | -0.2294 | 0.3169 | -0.0178 |
| C_2 | Gh_D12G0443 | NA | NA | 0.0010 | -1.7752 | 0.9594 | 0.9020 |
| C_2 | Gh_D12G0528 | NA | NA | 0.8685 | -0.9759 | 1.5759 | 0.3586 |
| C_2 | Gh_D12G0602 | NA | NA | 0.1627 | -0.6684 | 1.1460 | 0.0598 |
| C_2 | Gh_D12G0619 | At4g36750 | Probable NAD(P)H dehydrogenase (quinone) FQR1-like 2 | -0.0273 | -1.0916 | -0.1430 | -0.1686 |
| C_2 | Gh_D12G0627 | At2g18630 | UPF0496 protein At2g18630 | 0.3705 | -0.2471 | 1.0285 | 0.7155 |
| C_2 | Gh_D12G0779 | ATL54 | RING-H2 finger protein ATL54 | -0.1472 | -1.8932 | 1.7234 | -0.1145 |
| C_2 | Gh_D12G0973 | NA | NA | 1.3462 | -1.4309 | 1.9847 | 0.7754 |
| C_2 | Gh_D12G0977 | NA | NA | 1.0320 | -1.0514 | 1.8420 | 1.2395 |
| C_2 | Gh_D12G0981 | CYP714C2 | Cytochrome P450 714C2 | -0.4422 | -1.5825 | -0.6069 | -0.6249 |
| C_2 | Gh_D12G1124 | CNGC1 | Cyclic nucleotide-gated ion channel 1 | 0.5860 | -0.5927 | 1.1982 | 0.3240 |
| C_2 | Gh_D12G1126 | ATL21A | Putative RING-H2 finger protein | 0.7777 | -1.4837 | -0.3732 | -0.9487 |
| C_2 | Gh_D12G1199 | GLP10 | Germin-like protein subfamily 2 member 4 | 0.0815 | -0.2098 | 0.4258 | 0.1870 |
| C_2 | Gh_D12G1341 | At5g23160 | Uncharacterized protein | -0.4136 | -1.4223 | 0.7123 | -0.1365 |
| C_2 | Gh_D12G1391 | ERF053 | Ethylene-responsive transcription factor ERF053 | 1.6121 | 0.3568 | 1.9153 | -1.6677 |
| C_2 | Gh_D12G1525 | ADO3 | Adagio protein 3 | 0.9349 | -3.8408 | 1.5787 | -1.3112 |
| C_2 | Gh_D12G1564 | tmem97 | Transmembrane protein 97 | 0.2762 | -0.4828 | 0.1095 | -0.0319 |
| C_2 | Gh_D12G1593 | NPF5.6 | Protein NRT1/ PTR FAMILY 5.6 | 0.5273 | -1.1017 | 1.2442 | 0.9395 |
| C_2 | Gh_D12G1635 | B3GALT15 | Beta-1,3-galactosyltransferase 15 | 0.3268 | -0.6348 | 0.5600 | 0.7197 |
| C_2 | Gh_D12G1708 | ALA9 | Putative phospholipid-transporting ATPase 9 | -0.0742 | -0.8212 | 1.0029 | 0.2289 |
| C_2 | Gh_D12G1738 | ABCA7 | ABC transporter A family member 7 | -0.1532 | -1.3671 | 0.2062 | -0.2750 |
| C_2 | Gh_D12G1780 | At5g61290 | Flavin-containing monooxygenase FMO GS-OX-like 8 | 0.2958 | -0.4030 | 0.4313 | 0.1547 |
| C_2 | Gh_D12G1850 | SOBIR1 | Leucine-rich repeat receptor-like | 1.1824 | -0.5199 | 2.1653 | 0.7356 |
|  |  |  | serine/threonine/tyrosine-protein kinase |  |  |  |  |
| C_2 | Gh_D12G1954 | At5g07610 | F-box protein At5g07610 | 0.7885 | -4.2216 | 0.7513 | 0.4163 |
| C_2 | Gh_D12G1972 | NA | NA | 0.4677 | -0.6359 | -0.7830 | -0.6285 |
| C_2 | Gh_D12G1987 | EDS1 | Protein EDS1L | 0.0069 | -0.6248 | 0.1479 | -0.3727 |
| C_2 | Gh_D12G2016 | ERD3 | Probable methyltransferase PMT21 | 0.7441 | 0.5392 | 0.8506 | 0.2490 |
| C_2 | Gh_D12G2036 | CYP76B6 | Geraniol 8-hydroxylase | 0.4017 | -0.3899 | 0.4579 | -0.4457 |
| C_2 | Gh_D12G2065 | ABCB2 | ABC transporter B family member 2 | 0.3779 | -1.1521 | -0.9425 | -1.9031 |
| C_2 | Gh_D12G2090 | PLL1 | Protein phosphatase 2C 29 | 0.1205 | -1.1760 | 0.5655 | 0.6445 |
| C_2 | Gh_D12G2181 | NA | L-lactate dehydrogenase A | 1.0845 | -1.4119 | 1.3059 | -0.2505 |
| C_2 | Gh_D12G2332 | At3g05675 | BTB/POZ domain-containing protein | 0.2982 | -0.5179 | -0.5489 | -0.8837 |
| C_2 | Gh_D12G2367 | SBT3.3 | Subtilisin-like protease SBT3.3 | 0.1929 | -0.8136 | 0.3686 | -0.2662 |
| C_2 | Gh_D12G2433 | NA | NA | 0.4151 | -0.1228 | 1.1300 | -0.0522 |
| C_2 | Gh_D12G2434 | NA | NA | 1.0869 | -0.4132 | 1.4742 | 0.0606 |
| C_2 | Gh_D12G2531 | NA | NA | -0.1542 | -1.0730 | 0.0530 | -0.8141 |
| C_2 | Gh_D12G2533 | SARD1 | Protein SAR DEFICIENT 1 | -0.0856 | -2.0974 | 1.6390 | 0.4302 |
| C_2 | Gh_D12G2599 | ALDH2B4 | Aldehyde dehydrogenase family 2 member B4, | 0.3453 | -0.9472 | 0.2126 | -0.2176 |
|  |  |  | mitochondrial |  |  |  |  |
| C_2 | Gh_D12G2621 | AOX1A | Ubiquinol oxidase 1a, mitochondrial | 0.0793 | -3.7520 | 0.8126 | -1.6007 |
| C_2 | Gh_D12G2658 | CXE1 | Carboxylesterase 1 | 0.6589 | -2.0201 | 0.7813 | 0.0713 |
| C_2 | Gh_D12G2664 | NA | Vicianin hydrolase (Fragment) | 1.0611 | 0.4117 | 1.2838 | 0.0047 |
| C_2 | Gh_D12G2666 | ABCC10 | ABC transporter C family member 10 | 0.3871 | -0.0771 | 0.5478 | 0.3021 |
| C_2 | Gh_D12G2670 | CHIT3 | Acidic endochitinase | -0.8213 | -2.9469 | 0.8021 | 0.7478 |
| C_2 | Gh_D12G2822 | PCKR1 | Peptidyl-prolyl cis-trans isomerase | 0.4939 | -0.3388 | 0.3854 | 0.2858 |
| C_2 | Gh_D13G0053 | SINAT2 | E3 ubiquitin-protein ligase SINAT2 | -0.0406 | -0.8741 | -0.0309 | 0.3086 |
| C_2 | Gh_D13G0118 | PUB23 | E3 ubiquitin-protein ligase PUB23 | 0.8782 | -1.0726 | 0.4031 | -0.3426 |
| C_2 | Gh_D13G0190 | UGT91C1 | UDP-glycosyltransferase 91C1 | 0.4005 | -2.2796 | 0.7058 | -0.7374 |
| C_2 | Gh_D13G0264 | RDR1 | RNA-dependent RNA polymerase 1 | 0.1509 | -1.1223 | 0.1049 | 0.1510 |
| C_2 | Gh_D13G0308 | PAP2 | Purple acid phosphatase 2 | 0.8327 | -0.6538 | -0.0414 | -0.0924 |
| C_2 | Gh_D13G0312 | SD25 | G-type lectin S-receptor-like serine/threonine-protein | -0.3577 | -2.0024 | -0.3355 | 0.4902 |
|  |  |  | kinase |  |  |  |  |
| C_2 | Gh_D13G0415 | TOGT1 | Scopoletin glucosyltransferase | 0.0525 | -1.2724 | 1.0707 | 0.3337 |
| C_2 | Gh_D13G0417 | TOGT1 | Scopoletin glucosyltransferase | 0.3005 | -0.9516 | 0.3641 | 0.2084 |
| C_2 | Gh_D13G0430 | NA | Basic secretory protease (Fragments) | 0.3033 | -1.9636 | 2.0872 | -0.0917 |
| C_2 | Gh_D13G0460 | DIR1 | Putative lipid-transfer protein DIR1 | 0.8899 | -1.6779 | 1.1691 | 0.0059 |
| C_2 | Gh_D13G0476 | LECRK91 | L-type lectin-domain containing receptor kinase IX.1 | -0.5715 | -1.8240 | 0.8267 | 0.3290 |
| C_2 | Gh_D13G0546 | ST2 | High affinity sulfate transporter 2 | -2.8619 | -4.8834 | -1.7209 | -4.1586 |
| C_2 | Gh_D13G0551 | BAK1 | BRASSINOSTEROID INSENSITIVE 1-associated | 0.7629 | -0.2252 | 1.1925 | 0.6043 |
|  |  |  | receptor kinase 1 |  |  |  |  |
| C_2 | Gh_D13G0628 | NA | Cytochrome P450 CYP749A22 | -0.4429 | -0.6637 | -0.2524 | -0.4425 |
| C_2 | Gh_D13G0709 | NUDT1 | Nudix hydrolase 1 | 0.4327 | -0.4771 | 0.6694 | -0.1341 |
| C_2 | Gh_D13G0921 | NA | Cytochrome P450 CYP749A22 | -0.2476 | -0.8269 | 0.3992 | -0.3059 |
| C_2 | Gh_D13G1046 | RAP2-11 | Ethylene-responsive transcription factor RAP2-11 | -0.1458 | -1.3982 | -0.4639 | -1.0845 |
| C_2 | Gh_D13G1100 | NA | NA | -0.6512 | -1.5794 | -0.2471 | -0.3322 |
| C_2 | Gh_D13G1245 | NA | NA | -0.0243 | -0.3625 | 0.1145 | 0.3163 |
| C_2 | Gh_D13G1254 | NA | NA | 0.5143 | -0.9791 | 2.5401 | 1.0093 |
| C_2 | Gh_D13G1280 | STR19 | Rhodanese-like domain-containing protein 19, | 0.5115 | -0.5047 | -0.7226 | -0.7177 |
|  |  |  | mitochondrial |  |  |  |  |
| C_2 | Gh_D13G1616 | NA | Probable glutathione S-transferase | -2.1796 | -3.5069 | -2.0028 | -1.0693 |
| C_2 | Gh_D13G1652 | UGT73C1 | UDP-glycosyltransferase 73C1 | 0.4631 | -2.0734 | -0.3168 | -0.3217 |
| C_2 | Gh_D13G1658 | UGT73C3 | UDP-glycosyltransferase 73C3 | 0.3945 | -1.6130 | 0.0459 | 0.0386 |
| C_2 | Gh_D13G1838 | HST1 | Protein HASTY 1 | -0.0853 | -0.1374 | -0.1132 | -0.1205 |
| C_2 | Gh_D13G2060 | IQM1 | IQ domain-containing protein IQM1 | 0.7829 | -1.2839 | 0.2962 | -0.0548 |
| C_2 | Gh_D13G2148 | At4g11680 | E3 ubiquitin-protein ligase | 0.4184 | 0.1372 | 0.3121 | -0.0895 |
| C_2 | Gh_D13G2153 | AOP1 | Probable 2-oxoglutarate-dependent dioxygenase AOP1 | 0.1530 | -1.5128 | 1.7438 | -0.4313 |
| C_2 | Gh_D13G2155 | AOP1 | Probable 2-oxoglutarate-dependent dioxygenase AOP1 | 2.6972 | -0.3967 | 0.0993 | -0.9393 |
| C_2 | Gh_D13G2158 | AOP1 | Probable 2-oxoglutarate-dependent dioxygenase AOP1 | 0.5795 | -1.0907 | -0.0302 | -0.4270 |
| C_2 | Gh_D13G2176 | CASBPX2 | Cycloartenol synthase 2 | 0.7733 | -2.2541 | -0.4670 | 0.8143 |
| C_2 | Gh_D13G2192 | NA | NA | -0.0091 | -0.5751 | 0.7364 | -0.0951 |
| C_2 | Gh_D13G2197 | PPH | Pheophytinase, chloroplastic | -0.7856 | -1.6913 | -0.2269 | -0.5602 |
| C_2 | Gh_D13G2263 | SIR1 | Sulfite reductase 1 [ferredoxin], chloroplastic | -0.0701 | -0.8400 | -0.6567 | -0.7317 |
| C_2 | Gh_D13G2314 | aifA | Apoptosis-inducing factor homolog A | 0.0991 | -1.7700 | 0.7850 | -0.5099 |
| C_2 | Gh_D13G2451 | UGT87A2 | UDP-glycosyltransferase 87A2 | -0.2555 | -1.2084 | -0.1953 | -0.1131 |
| C_2 | Gh_D13G2465 | AOP1.2 | Probable 2-oxoglutarate-dependent dioxygenase AOP1.2 | 1.3995 | -0.5584 | 0.9822 | -1.1561 |
| C_2 | Gh_D13G2467 | PSKR2 | Phytosulfokine receptor 2 | 0.6373 | -1.6394 | 1.3271 | 0.2530 |
| C_2 | Gh_D13G2490 | At5g01020 | Serine/threonine-protein kinase | 0.7850 | -0.3307 | 1.1581 | 0.2101 |
| C_2 | Gh_D13G2524 | LAC7 | Laccase-7 | 1.1110 | -1.9050 | 0.7591 | -2.2979 |
| C_2 | Gh_Sca004760G01 | NAC090 | NAC domain-containing protein 90 | -0.3150 | -4.4150 | 2.5388 | 0.3631 |
| C_2 | Gh_Sca004768G06 | Trafd1 | TRAF-type zinc finger domain-containing protein 1 | 0.4386 | 0.0147 | 0.5708 | -0.3387 |
| C_2 | Gh_Sca004827G03 | NA | NA | -0.0093 | -0.8715 | -0.2079 | -0.6731 |
| C_2 | Gh_Sca004885G02 | CPIJ013394 | O-glucosyltransferase rumi homolog | 0.0507 | -0.8384 | -0.5846 | -0.1376 |
| C_2 | Gh_Sca004922G01 | NA | Basic secretory protease (Fragments) | 0.6167 | -1.4692 | 1.7481 | 0.3348 |
| C_2 | Gh_Sca004965G02 | UPL2 | E3 ubiquitin-protein ligase UPL2 | 0.2060 | -0.3938 | -0.5168 | -0.4978 |
| C_2 | Gh_Sca004981G04 | CAN1 | Staphylococcal-like nuclease CAN1 | -0.0652 | -1.7438 | 0.2912 | -1.0323 |
| C_2 | Gh_Sca005059G05 | PEX5 | Peroxisome biogenesis protein 5 | -0.0005 | -0.5053 | -0.1720 | -0.2212 |
| C_2 | Gh_Sca005098G01 | SUVH1 | Histone-lysine N-methyltransferase, H3 lysine-9 specific | 0.2032 | -0.1672 | 0.1137 | -0.2199 |
|  |  |  | SUVH1 |  |  |  |  |
| C_2 | Gh_Sca005135G02 | ASP | 21 kDa seed protein | 1.3091 | -2.5012 | -0.8463 | -2.3995 |
| C_2 | Gh_Sca005145G01 | E2FA | Transcription factor E2FA | 0.5485 | -0.6525 | 0.2001 | 0.0906 |
| C_2 | Gh_Sca005145G02 | HVA22J | HVA22-like protein j | -0.4842 | -1.6252 | -0.3188 | -0.7819 |
| C_2 | Gh_Sca005221G01 | CYP88D6 | Beta-amyrin 11-oxidase | 0.1181 | -1.4014 | 1.5270 | 0.9617 |
| C_2 | Gh_Sca005341G02 | NA | NA | 0.3914 | -0.8094 | -0.0612 | 0.1306 |
| C_2 | Gh_Sca005400G01 | AGO7 | Protein argonaute 7 | 0.7189 | -0.3812 | 0.5086 | 0.6586 |
| C_2 | Gh_Sca005979G01 | HAT5 | Homeobox-leucine zipper protein HAT5 | 0.2834 | -0.7526 | -0.3904 | -0.5229 |
| C_2 | Gh_Sca008656G01 | AOP1 | Probable 2-oxoglutarate-dependent dioxygenase AOP1 | 0.4447 | -0.8928 | 0.7765 | -0.9917 |
| C_2 | Gh_Sca010215G01 | ABCC3 | ABC transporter C family member 3 | 0.4622 | -3.0877 | 1.1025 | -0.6556 |
| C_2 | Gh_Sca013634G01 | APRR5 | Two-component response regulator-like APRR5 | 0.7522 | -1.9900 | 1.0609 | -0.5320 |
| C_3 | Gh_A01G0025 | UPL3 | E3 ubiquitin-protein ligase UPL3 | -0.3446 | 0.6617 | -1.0697 | 0.4671 |
| C_3 | Gh_A01G0095 | MRS2-3 | Magnesium transporter MRS2-3 | 0.6871 | 0.3297 | -0.3897 | 0.3620 |
| C_3 | Gh_A01G0167 | LACS7 | Long chain acyl-CoA synthetase 7, peroxisomal | -0.3421 | -0.0191 | -0.2086 | -0.0065 |
| C_3 | Gh_A01G0176 | Stard7 | StAR-related lipid transfer protein 7, mitochondrial | 0.5137 | 0.5634 | 0.0926 | 0.6237 |
| C_3 | Gh_A01G0233 | NA | NA | -0.0662 | -0.0558 | -0.4102 | -0.0719 |
| C_3 | Gh_A01G0448 | NA | NA | -0.5250 | 1.3546 | -0.2229 | 4.9452 |
| C_3 | Gh_A01G0457 | POPTR_0001s40980g | Probable bifunctional methylthioribulose-1-phosphate dehydratase/enolase-phosphatase E1 | 0.0534 | -0.2226 | -0.4606 | -0.1932 |
| C_3 | Gh_A01G0501 | ELAC2 | Zinc phosphodiesterase ELAC protein 2 | 0.3546 | 0.2022 | -0.8412 | -0.4679 |
| C_3 | Gh_A01G0868 | NA | NA | 1.3417 | 2.8132 | -3.4409 | 1.0571 |
| C_3 | Gh_A01G0877 | CG18812 | Protein GDAP2 homolog | -0.5579 | -0.1628 | -2.4037 | -1.2580 |
| C_3 | Gh_A01G1001 | TAR4 | Tryptophan aminotransferase-related protein 4 | -0.6347 | 0.1072 | -0.5714 | 0.4547 |
| C_3 | Gh_A01G1254 | PIN3 | Auxin efflux carrier component 3 | -0.2366 | 0.6912 | -0.4522 | 0.5259 |
| C_3 | Gh_A01G1660 | CYP93A1 | 3,9-dihydroxypterocarpan 6A-monooxygenase | -0.3099 | -0.7071 | -2.2074 | -1.0230 |
| C_3 | Gh_A01G1784 | PTL | Trihelix transcription factor PTL | -1.4486 | 1.9216 | -3.1495 | 1.7966 |
| C_3 | Gh_A02G0011 | NA | NA | -1.1399 | -0.4878 | -3.3109 | -1.5776 |
| C_3 | Gh_A02G0055 | At3g08860 | Alanine--glyoxylate aminotransferase 2 homolog 3, | -1.3816 | 0.2579 | -2.8314 | 0.0511 |
|  |  |  | mitochondrial |  |  |  |  |
| C_3 | Gh_A02G0224 | NA | Cytochrome P450 CYP749A22 | 3.4182 | 1.4674 | 0.4338 | 1.7534 |
| C_3 | Gh_A02G0234 | NA | NA | -1.2239 | -1.6986 | -5.2353 | 0.4230 |
| C_3 | Gh_A02G0261 | NA | Probable glutathione S-transferase | -0.8859 | -0.7080 | -0.8647 | -0.3060 |
| C_3 | Gh_A02G0272 | At2g33170 | Probable leucine-rich repeat receptor-like protein kinase | -0.0670 | -0.1701 | -7.8598 | -1.1980 |
| C_3 | Gh_A02G0442 | RF298 | Putative E3 ubiquitin-protein ligase | 0.0907 | -0.3481 | -0.3082 | 0.3614 |
| C_3 | Gh_A02G0677 | RCH1 | LRR receptor-like serine/threonine-protein kinase | -0.3065 | 0.8503 | -3.9195 | 0.6486 |
| C_3 | Gh_A02G0689 | ADH1 | Alcohol dehydrogenase class-P | -0.1711 | 0.7751 | 0.3284 | 0.7100 |
| C_3 | Gh_A02G0744 | tolB | Protein TolB | -1.2532 | -1.3857 | -1.7531 | -0.5123 |
| C_3 | Gh_A02G0845 | NA | NA | -0.7984 | -1.2706 | -1.8037 | 0.5011 |
| C_3 | Gh_A02G0980 | NA | NA | -0.1698 | -0.5399 | -2.5229 | -1.1998 |
| C_3 | Gh_A02G1009 | ABCC10 | ABC transporter C family member 10 | -0.3793 | -0.1780 | -0.4340 | -0.1843 |
| C_3 | Gh_A02G1014 | CHX18 | Cation/H(+) antiporter 18 | -0.0059 | -0.9960 | -3.0742 | -2.0930 |
| C_3 | Gh_A02G1160 | PDR3 | Pleiotropic drug resistance protein 3 | -0.1353 | 1.5148 | -0.4072 | 1.6791 |
| C_3 | Gh_A02G1211 | At3g02910 | Putative gamma-glutamylcyclotransferase | 0.4951 | -1.1891 | -1.5850 | -0.3777 |
| C_3 | Gh_A02G1212 | At3g50808 | Uncharacterized protein At3g50808 | -0.5842 | -0.4404 | -0.5719 | -0.0632 |
| C_3 | Gh_A02G1452 | NA | NA | -0.2143 | 0.1654 | -0.9557 | -0.4336 |
| C_3 | Gh_A02G1597 | NA | NA | 0.3607 | -0.4483 | -0.8438 | -0.8116 |
| C_3 | Gh_A02G1621 | NA | NA | -1.7658 | -0.2855 | -3.9190 | -0.3275 |
| C_3 | Gh_A02G1638 | NA | Thaumatin-like protein 1 | -2.1412 | 0.1387 | -1.4062 | 0.7645 |
| C_3 | Gh_A03G0050 | SEOB | Protein SIEVE ELEMENT OCCLUSION B | 2.3307 | -0.1726 | -1.5906 | -0.7039 |
| C_3 | Gh_A03G0097 | NPF6.3 | Protein NRT1/ PTR FAMILY 6.3 | -1.0137 | -0.5229 | -1.1286 | -0.2975 |
| C_3 | Gh_A03G0199 | GSVIVT00023967001 | Peroxidase 4 | 0.3241 | 0.2827 | -0.1003 | 0.0786 |
| C_3 | Gh_A03G0228 | At2g23060 | Probable N-acetyltransferase HLS1-like | 0.3804 | 0.0450 | -1.6694 | -0.2859 |
| C_3 | Gh_A03G0312 | NA | NA | -0.4724 | -0.3798 | -2.7342 | 0.1455 |
| C_3 | Gh_A03G0314 | HERK1 | Receptor-like protein kinase HERK 1 | -0.4952 | 1.2290 | -0.3275 | 0.8701 |
| C_3 | Gh_A03G0379 | TOPP2 | Serine/threonine-protein phosphatase PP1 isozyme 2 | 1.0618 | 1.4533 | -3.5214 | 0.6238 |
| C_3 | Gh_A03G0386 | At3g01520 | Universal stress protein A-like protein | 0.2832 | 0.7949 | -1.5189 | 0.0472 |
| C_3 | Gh_A03G0536 | GDH2 | Glutamate dehydrogenase 2 | -0.9253 | 0.0981 | -0.4167 | 0.3851 |
| C_3 | Gh_A03G0673 | NA | Transcription factor MYB1R1 | -2.1117 | 0.5678 | -4.7967 | -0.8685 |
| C_3 | Gh_A03G0803 | NMT1 | Phosphoethanolamine N-methyltransferase 1 | 0.2435 | 0.7817 | -0.5408 | 1.0916 |
| C_3 | Gh_A03G1179 | PHT2-1 | Inorganic phosphate transporter 2-1, chloroplastic | -0.1709 | 0.3307 | -0.5097 | 1.2082 |
| C_3 | Gh_A03G1391 | 7OMT | (R,S)-reticuline 7-O-methyltransferase | -0.0368 | 0.1309 | 0.0570 | 0.3136 |
| C_3 | Gh_A03G1463 | RING1 | E3 ubiquitin-protein ligase RING1 | 0.0455 | -0.0289 | -21.4654 | -4.8028 |
| C_3 | Gh_A03G1811 | MYB308 | Myb-related protein 308 | -2.0612 | -0.3827 | -2.1048 | -0.4253 |
| C_3 | Gh_A03G1909 | NA | NA | -0.2824 | 0.4752 | -0.0057 | 0.4014 |
| C_3 | Gh_A03G1987 | NA | NA | -2.3645 | 0.8970 | -1.8683 | 0.0323 |
| C_3 | Gh_A03G2050 | TAF15B | Transcription initiation factor TFIID subunit 15b | 0.4509 | 0.1928 | 0.1204 | 0.4966 |
| C_3 | Gh_A03G2119 | CYP74A | Allene oxide synthase, chloroplastic | 0.0308 | 0.0009 | -0.2286 | 0.3756 |
| C_3 | Gh_A04G0049 | At1g06890 | Uncharacterized membrane protein | -0.2073 | 0.5014 | -0.8440 | 0.5658 |
| C_3 | Gh_A04G0108 | NA | Flavonol sulfotransferase-like | -3.3888 | -0.2434 | -2.0186 | 0.5160 |
| C_3 | Gh_A04G0147 | AUG | AUGMIN subunit 4 | -0.1859 | -0.8367 | -0.9079 | -0.1613 |
| C_3 | Gh_A04G0279 | EMB2654 | Pentatricopeptide repeat-containing protein | -0.4497 | 0.5801 | -0.8224 | 0.0186 |
| C_3 | Gh_A04G0398 | CYP71D10 | Cytochrome P450 71D10 | 0.4886 | -0.2179 | -0.5434 | 0.6518 |
| C_3 | Gh_A04G0520 | SP | Protein SELF-PRUNING | 2.6117 | 0.7350 | -2.3052 | -0.9314 |
| C_3 | Gh_A04G0636 | UBQ10 | Polyubiquitin 10 | -0.9389 | 0.4642 | -0.7053 | 0.2362 |
| C_3 | Gh_A04G0721 | RCA1 | Ribulose bisphosphate carboxylase/oxygenase activase 1, | -1.2875 | 0.5310 | -1.5895 | 0.7744 |
|  |  |  | chloroplastic |  |  |  |  |
| C_3 | Gh_A04G0739 | UGT709C2 | 7-deoxyloganetic acid glucosyltransferase | 0.3892 | 0.4194 | -1.2799 | 0.2966 |
| C_3 | Gh_A04G0823 | NA | Polygalacturonase | 0.1582 | 0.0364 | -2.7476 | -0.5841 |
| C_3 | Gh_A04G1189 | UPB1 | Transcription factor UPBEAT1 | -0.2406 | 0.5001 | 0.0927 | 0.5938 |
| C_3 | Gh_A05G0024 | At1g16060 | AP2-like ethylene-responsive transcription factor | -0.0051 | 0.7458 | -1.4766 | 1.3307 |
| C_3 | Gh_A05G0041 | CID6 | Polyadenylate-binding protein-interacting protein 6 | -1.0732 | -1.2682 | -3.6302 | -1.8924 |
| C_3 | Gh_A05G0083 | NA | NA | -1.1611 | 0.4150 | -0.7283 | 1.0846 |
| C_3 | Gh_A05G0327 | IRT3 | Fe(2+) transport protein 3, chloroplastic | -1.4144 | 0.1741 | -3.5856 | -0.5858 |
| C_3 | Gh_A05G0670 | FD3 | Ferredoxin-3, chloroplastic | -1.2092 | 0.6047 | -0.2414 | 0.6351 |
| C_3 | Gh_A05G0706 | NA | NA | 0.1929 | 1.7066 | -2.8497 | -0.8967 |
| C_3 | Gh_A05G0721 | ZAT5 | Zinc finger protein ZAT5 | -0.5491 | 0.6739 | -1.1826 | 0.2127 |
| C_3 | Gh_A05G0763 | At2g38010 | Neutral ceramidase | -0.4306 | 0.5806 | -1.0022 | 0.2011 |
| C_3 | Gh_A05G0830 | RPS15AA | 40S ribosomal protein S15a-1 | 0.6165 | 0.3863 | 0.1448 | 0.4626 |
| C_3 | Gh_A05G0854 | PHOT1 | Phototropin-1 | -1.1458 | 1.0007 | -0.3106 | 2.0841 |
| C_3 | Gh_A05G0919 | NRT2.1 | High-affinity nitrate transporter 2.1 | -1.1651 | -0.6408 | -1.0814 | 1.3856 |
| C_3 | Gh_A05G0934 | BAG4 | BAG family molecular chaperone regulator 4 | -0.0891 | 0.6662 | -2.0252 | 0.5472 |
| C_3 | Gh_A05G0992 | HST | Shikimate O-hydroxycinnamoyltransferase | -0.0057 | -0.5710 | -1.0252 | -0.2071 |
| C_3 | Gh_A05G1057 | HSP26.5 | 26.5 kDa heat shock protein, mitochondrial | -1.0219 | 1.3891 | -1.1518 | 0.2157 |
| C_3 | Gh_A05G1059 | NA | NA | 0.2397 | 0.6619 | -2.1497 | -1.0188 |
| C_3 | Gh_A05G1092 | IAA26 | Auxin-responsive protein IAA26 | 0.0356 | 0.7774 | -0.6575 | -0.0206 |
| C_3 | Gh_A05G1142 | At3g13980 | Protein BIG GRAIN 1-like A | 0.1818 | 1.1684 | 0.2187 | 1.2761 |
| C_3 | Gh_A05G1179 | MPE3 | Pectinesterase 3 | 0.2224 | 0.6194 | 0.1350 | 0.4307 |
| C_3 | Gh_A05G1186 | SRF6 | Protein STRUBBELIG-RECEPTOR FAMILY 6 | -0.0969 | 0.3539 | -0.2012 | 0.8144 |
| C_3 | Gh_A05G1492 | ACO | 1-aminocyclopropane-1-carboxylate oxidase | -1.0374 | -0.1582 | -1.6542 | -0.1125 |
| C_3 | Gh_A05G1580 | FD | Protein FD | -0.3215 | -3.0244 | -5.4027 | -2.3259 |
| C_3 | Gh_A05G1619 | SRS5 | Protein SHI RELATED SEQUENCE 5 | 0.5850 | 0.5505 | -0.8130 | 0.1532 |
| C_3 | Gh_A05G1722 | MIZ1 | Protein MIZU-KUSSEI 1 | 0.0806 | 2.0161 | 0.4330 | 1.6720 |
| C_3 | Gh_A05G1792 | CYP81E1 | Isoflavone 2'-hydroxylase | 0.1493 | 0.1887 | -0.1344 | 0.1519 |
| C_3 | Gh_A05G1798 | CYP81E8 | Cytochrome P450 81E8 | -0.3048 | 1.0839 | -1.1759 | 0.8565 |
| C_3 | Gh_A05G1889 | CUTA | Protein CutA, chloroplastic | -0.2608 | 0.4049 | 0.1013 | 0.6855 |
| C_3 | Gh_A05G1895 | FIP1 | GEM-like protein 1 | -0.0869 | 0.9646 | -0.3085 | 0.6059 |
| C_3 | Gh_A05G1910 | NA | Blue copper protein | -0.5472 | 1.0313 | -0.7617 | 0.8760 |
| C_3 | Gh_A05G1913 | ATHB-6 | Homeobox-leucine zipper protein ATHB-6 | -0.1389 | 1.7244 | -0.5909 | 0.7216 |
| C_3 | Gh_A05G1941 | CRY1 | Cryptochrome-1 | -0.5159 | 0.8768 | -0.5264 | 1.0079 |
| C_3 | Gh_A05G2005 | CPK7 | Calcium-dependent protein kinase 7 | -0.2860 | 0.2428 | -8.3585 | -0.4765 |
| C_3 | Gh_A05G2039 | PERK13 | Proline-rich receptor-like protein kinase PERK13 | 0.4369 | -1.2007 | -3.9326 | 2.1678 |
| C_3 | Gh_A05G2062 | At3g43660 | Vacuolar iron transporter homolog 4 | -1.0100 | -0.2502 | -2.3523 | 0.3114 |
| C_3 | Gh_A05G2144 | Cag_1601 | UPF0301 protein Cag_1601 | 0.1617 | -0.1687 | -0.5615 | 0.1100 |
| C_3 | Gh_A05G2275 | NA | Blue copper protein | -1.3374 | -1.0895 | -2.7457 | 0.7206 |
| C_3 | Gh_A05G2316 | VIP1 | Transcription factor VIP1 | 0.2220 | -0.3760 | -0.5164 | -0.3701 |
| C_3 | Gh_A05G2336 | PATL5 | Patellin-5 | -0.1262 | 0.7543 | 0.0322 | 0.4403 |
| C_3 | Gh_A05G2391 | At5g24080 | G-type lectin S-receptor-like serine/threonine-protein | -0.5080 | 0.4012 | -2.3784 | 0.0562 |
|  |  |  | kinase |  |  |  |  |
| C_3 | Gh_A05G2476 | PTL | Trihelix transcription factor PTL | 4.4461 | 1.4533 | -1.7754 | 1.2062 |
| C_3 | Gh_A05G2477 | ATL7 | RING-H2 finger protein ATL7 | 0.1205 | -0.2470 | -0.7063 | -0.3418 |
| C_3 | Gh_A05G2925 | SKL1 | Probable inactive shikimate kinase like 1, chloroplastic | -0.5324 | 1.0546 | -2.4602 | -0.0721 |
| C_3 | Gh_A05G3065 | SULTR4 | Sulfate transporter 4.1, chloroplastic | -1.7201 | 0.6594 | -0.8703 | 0.7489 |
| C_3 | Gh_A05G3125 | NPHP3 | Nephrocystin-3 | -0.3856 | -0.0752 | -0.6205 | 0.0830 |
| C_3 | Gh_A05G3144 | NPF5.8 | Protein NRT1/ PTR FAMILY 5.8 | -0.5586 | 0.0954 | -1.2542 | -0.2272 |
| C_3 | Gh_A05G3268 | GAUT15 | Probable galacturonosyltransferase 15 | -0.3332 | 0.7245 | -0.4067 | 0.6737 |
| C_3 | Gh_A05G3319 | NA | NA | -0.2373 | -0.4779 | -1.7485 | -0.4934 |
| C_3 | Gh_A05G3361 | GH3.17 | Indole-3-acetic acid-amido synthetase GH3.17 | -0.6339 | 1.2664 | -0.7851 | 0.8105 |
| C_3 | Gh_A05G3362 | GH3.17 | Indole-3-acetic acid-amido synthetase GH3.17 | -0.5401 | 0.2667 | -0.4975 | 0.5822 |
| C_3 | Gh_A05G3383 | Seipin | Seipin | 3.3143 | 4.7188 | -1.1658 | 2.5339 |
| C_3 | Gh_A05G3397 | PKS4 | Protein PHYTOCHROME KINASE SUBSTRATE 4 | -0.6445 | 1.9921 | -1.8113 | 0.6707 |
| C_3 | Gh_A05G3466 | ASIL2 | Trihelix transcription factor ASIL2 | 0.1901 | 0.3089 | -0.6066 | -0.2171 |
| C_3 | Gh_A05G3744 | SELK | Selenoprotein K | 0.0680 | 0.3203 | -0.5750 | -0.0514 |
| C_3 | Gh_A05G3756 | CIPK8 | CBL-interacting serine/threonine-protein kinase 8 | -0.6382 | 0.6230 | -0.4699 | 0.3084 |
| C_3 | Gh_A05G3812 | CYP710A1 | Cytochrome P450 710A1 | 0.3067 | 0.5945 | 0.3023 | 0.4976 |
| C_3 | Gh_A05G3873 | APT5 | Adenine phosphoribosyltransferase 5 | -0.0865 | 0.4763 | 0.1779 | 0.6035 |
| C_3 | Gh_A05G3967 | CESA1 | Cellulose synthase A catalytic subunit 1 [UDP-forming] | -0.0089 | 0.6589 | -0.0100 | 0.3411 |
| C_3 | Gh_A05G3995 | CHLN | Nicotianamine synthase | 0.2431 | 0.5158 | 0.3736 | 0.5970 |
| C_3 | Gh_A06G0020 | CRSP | CO(2)-response secreted protease | 0.1906 | 0.8254 | -0.8873 | 0.2203 |
| C_3 | Gh_A06G0128 | KO | Ent-kaurene oxidase, chloroplastic | 0.1915 | -0.1380 | -1.6898 | -0.5960 |
| C_3 | Gh_A06G0168 | At3g50280 | Uncharacterized acetyltransferase | -0.1647 | -1.6921 | -5.5325 | -0.0808 |
| C_3 | Gh_A06G0223 | BHLH74 | Transcription factor bHLH74 | -0.1186 | 0.7607 | 0.2966 | 0.8359 |
| C_3 | Gh_A06G0321 | MUB6 | Membrane-anchored ubiquitin-fold protein 6 | 0.1654 | 0.4756 | -0.5367 | 0.0338 |
| C_3 | Gh_A06G0323 | NA | NA | -0.3761 | -1.3009 | -1.6561 | -0.3243 |
| C_3 | Gh_A06G0390 | NRAMP5 | Metal transporter Nramp5 | 1.0205 | -0.1662 | -0.1997 | 5.6655 |
| C_3 | Gh_A06G0409 | STP7 | Sugar transport protein 7 | -0.2298 | -1.0180 | -1.3638 | -1.3189 |
| C_3 | Gh_A06G0433 | NA | NA | -0.0421 | 0.4755 | 0.0114 | 0.4003 |
| C_3 | Gh_A06G0437 | SRG1 | Protein SRG1 | -0.4898 | 1.4507 | 0.4308 | 4.8953 |
| C_3 | Gh_A06G0459 | NA | NA | -1.1152 | -0.1506 | -0.9118 | 0.0846 |
| C_3 | Gh_A06G0482 | SEC11C | Signal peptidase complex catalytic subunit SEC11C | -0.6918 | 0.0687 | -0.3154 | 0.8533 |
| C_3 | Gh_A06G0552 | NA | NA | -0.0916 | -2.2309 | -3.0480 | -0.4820 |
| C_3 | Gh_A06G0562 | At2g20050/At | Protein phosphatase 2C and cyclic nucleotide- | -0.7113 | 0.9533 | -0.2516 | 0.5037 |
|  |  | 2g20040 | binding/kinase domain-containing protein |  |  |  |  |
| C_3 | Gh_A06G0584 | At4g29120 | Probable 3-hydroxyisobutyrate dehydrogenase-like 1, | 0.2559 | -0.1969 | -0.7582 | 0.3087 |
|  |  |  | mitochondrial |  |  |  |  |
| C_3 | Gh_A06G0597 | APS1 | Acid phosphatase 1 | -0.0871 | -0.0815 | -0.6695 | -0.2571 |
| C_3 | Gh_A06G0628 | NA | Transcription factor MYB1R1 | -1.7001 | 0.5259 | -2.0349 | -0.2495 |
| C_3 | Gh_A06G0664 | SPBC776.05 | Uncharacterized membrane protein C776.05 | -0.3205 | -0.1039 | -0.2545 | 0.2422 |
| C_3 | Gh_A06G0723 | mhpC | 2-hydroxy-6-oxononadienedioate/2-hydroxy-6- | -0.4832 | 0.1060 | -1.2822 | 1.2955 |
|  |  |  | oxononatrienedioate hydrolase |  |  |  |  |
| C_3 | Gh_A06G0785 | NA | NA | -0.4562 | 0.4967 | -1.3990 | 0.5240 |
| C_3 | Gh_A06G0839 | NA | NA | -0.0545 | 0.6907 | -1.9495 | -0.6519 |
| C_3 | Gh_A06G0948 | At2g24580 | Probable sarcosine oxidase | -0.7018 | -1.4383 | -1.4747 | -0.8034 |
| C_3 | Gh_A06G1093 | AHL | PAP-specific phosphatase HAL2-like | -1.2818 | 1.1430 | -0.8416 | 2.5295 |
| C_3 | Gh_A06G1144 | ERF4 | Ethylene-responsive transcription factor 4 | 0.2521 | 0.1130 | 0.0251 | 0.3149 |
| C_3 | Gh_A06G1148 | BBX32 | B-box zinc finger protein 32 | 0.3200 | 0.7232 | 0.5447 | 0.7330 |
| C_3 | Gh_A06G1205 | YUC6 | Indole-3-pyruvate monooxygenase YUCCA6 | -1.4112 | -0.3607 | -4.0925 | -0.5820 |
| C_3 | Gh_A06G1270 | CRK25 | Cysteine-rich receptor-like protein kinase 25 | 0.1223 | 0.9627 | 0.3406 | 0.8831 |
| C_3 | Gh_A06G1276 | UBQ11 | Polyubiquitin 11 | -0.0481 | 0.3807 | -0.5542 | 0.1735 |
| C_3 | Gh_A06G1450 | TRG-31 | Probable aquaporin PIP-type 7a | -0.8473 | 0.6638 | -1.1617 | 0.2551 |
| C_3 | Gh_A06G1578 | NA | Taxadiene 5-alpha hydroxylase | 0.9800 | -0.3786 | -1.0645 | -0.2661 |
| C_3 | Gh_A06G1686 | At4g14610 | Probable disease resistance protein | -0.1987 | 0.3188 | -2.8418 | -0.3412 |
| C_3 | Gh_A06G1734 | GT-2 | Trihelix transcription factor GT-2 | 0.0808 | 0.3123 | 0.0497 | 0.4758 |
| C_3 | Gh_A06G1861 | NA | Squalene monooxygenase | 0.2285 | 0.3070 | 0.0552 | 0.2621 |
| C_3 | Gh_A06G1895 | ZHD1 | Zinc-finger homeodomain protein 2 | 1.0544 | 0.7326 | 0.5047 | 2.1281 |
| C_3 | Gh_A07G0026 | FATB1 | Palmitoyl-acyl carrier protein thioesterase, chloroplastic | 0.3020 | 0.5378 | 0.2391 | 0.8355 |
| C_3 | Gh_A07G0087 | At4g29190 | Zinc finger CCCH domain-containing protein 49 | -0.5177 | -0.7613 | -1.7070 | -0.3131 |
| C_3 | Gh_A07G0115 | HSFA2 | Heat stress transcription factor A-2 | -1.6793 | -0.0843 | -3.3501 | 0.7104 |
| C_3 | Gh_A07G0260 | CIPK5 | CBL-interacting serine/threonine-protein kinase 5 | 0.3513 | -0.0932 | -0.8255 | -0.2111 |
| C_3 | Gh_A07G0277 | EDR2L | Protein ENHANCED DISEASE RESISTANCE 2-like | 0.5914 | 0.7408 | 0.1709 | 0.8941 |
| C_3 | Gh_A07G0317 | IAA29 | Auxin-responsive protein IAA29 | -0.0773 | -0.1182 | -0.6945 | 1.0519 |
| C_3 | Gh_A07G0432 | NA | NA | -0.8434 | -0.2460 | -2.9975 | 1.9918 |
| C_3 | Gh_A07G0466 | At1g04910 | Uncharacterized protein | -0.6315 | -0.5687 | -1.1389 | -0.3174 |
| C_3 | Gh_A07G0484 | PIMT1 | Protein-L-isoaspartate O-methyltransferase 1 | 0.0030 | -0.7414 | -0.6703 | 0.7833 |
| C_3 | Gh_A07G0682 | tmp1 | Thymidylate kinase | 0.2057 | -0.4808 | -0.7550 | 0.1558 |
| C_3 | Gh_A07G0687 | HIPP26 | Heavy metal-associated isoprenylated plant protein 26 | -0.6570 | -1.5980 | -2.5132 | -1.4849 |
| C_3 | Gh_A07G0716 | DHAR3 | Glutathione S-transferase DHAR3, chloroplastic | -0.3289 | 0.7094 | -2.3557 | -0.2605 |
| C_3 | Gh_A07G0842 | GAUT8 | Galacturonosyltransferase 8 | 0.0910 | 0.3930 | 0.2299 | 0.4522 |
| C_3 | Gh_A07G0879 | NA | NA | -0.9322 | 0.3134 | -0.7793 | 0.2750 |
| C_3 | Gh_A07G0906 | SCL14 | Scarecrow-like protein 14 | -0.1965 | 1.3774 | -0.6088 | 1.4157 |
| C_3 | Gh_A07G0924 | HSP18.2 | 18.2 kDa class I heat shock protein | -1.4694 | 1.0297 | -7.5035 | -0.9804 |
| C_3 | Gh_A07G1181 | LACS1 | Long chain acyl-CoA synthetase 1 | -0.6657 | 0.5733 | -0.6531 | 0.2329 |
| C_3 | Gh_A07G1256 | GLP6 | Germin-like protein subfamily 1 member 13 | -1.0809 | -1.2155 | -1.8837 | 0.8828 |
| C_3 | Gh_A07G1310 | PHT4 | Ascorbate transporter, chloroplastic | 0.1500 | 0.3211 | -0.4930 | 0.5520 |
| C_3 | Gh_A07G1878 | SS2 | Granule-bound starch synthase 2, | -0.3268 | -0.0735 | -1.2611 | 0.4119 |
|  |  |  | chloroplastic/amyloplastic |  |  |  |  |
| C_3 | Gh_A07G1886 | At5g39570 | Uncharacterized protein | 0.1782 | 0.2280 | -0.1466 | 0.0969 |
| C_3 | Gh_A07G2014 | FDH1 | Formate dehydrogenase, mitochondrial | -0.8449 | 0.3876 | -0.9647 | 0.3294 |
| C_3 | Gh_A07G2033 | At1g48100 | Polygalacturonase | 1.1363 | 2.1344 | -0.6928 | 2.7599 |
| C_3 | Gh_A07G2085 | NA | NA | -0.0820 | 0.5211 | -0.6319 | 0.4787 |
| C_3 | Gh_A07G2098 | SOX | Sulfite oxidase | -0.9522 | 0.6353 | -0.9058 | -0.0581 |
| C_3 | Gh_A07G2176 | At3g07870 | F-box protein | -0.5751 | -0.3146 | -1.0247 | -0.2122 |
| C_3 | Gh_A08G0102 | NA | NA | 0.1227 | 0.4087 | -0.3463 | 0.5097 |
| C_3 | Gh_A08G0125 | At2g37990 | Ribosome biogenesis regulatory protein homolog | 0.9389 | -2.3312 | -5.8914 | -0.2464 |
| C_3 | Gh_A08G0224 | PRA1G2 | PRA1 family protein G2 | 0.3826 | 0.0968 | 0.0653 | 0.3299 |
| C_3 | Gh_A08G0299 | MYB3 | Transcription factor MYB3 | -0.2536 | -0.3042 | -4.3141 | 1.3187 |
| C_3 | Gh_A08G0386 | NA | Desiccation-related protein PCC13-62 | -1.2424 | 0.0566 | -2.7921 | 2.0468 |
| C_3 | Gh_A08G0403 | CBSDUF2 | DUF21 domain-containing protein | -0.8081 | -1.3984 | -1.9376 | 1.4206 |
| C_3 | Gh_A08G0508 | RPPL1 | Putative disease resistance RPP13-like protein 1 | -0.5472 | 0.5277 | -0.7022 | 0.0214 |
| C_3 | Gh_A08G0518 | NA | NA | 0.5949 | 1.1726 | 0.4952 | 0.9922 |
| C_3 | Gh_A08G0608 | NA | NA | -0.8540 | 0.0498 | -1.2427 | 0.8906 |
| C_3 | Gh_A08G0708 | NA | NA | -0.3236 | 0.1233 | -0.1071 | 0.1181 |
| C_3 | Gh_A08G0775 | COL5 | Zinc finger protein CONSTANS-LIKE 5 | -0.5983 | 0.6617 | -0.0156 | 0.8540 |
| C_3 | Gh_A08G0819 | NA | NA | 0.4176 | 0.8957 | -0.2104 | 0.3078 |
| C_3 | Gh_A08G0834 | NA | NA | -0.6775 | -1.0745 | -1.5741 | -0.5011 |
| C_3 | Gh_A08G0887 | NA | NA | -0.2282 | -0.3377 | -2.3026 | -0.6494 |
| C_3 | Gh_A08G0957 | WAXY | Granule-bound starch synthase 1, | -1.6032 | 0.1782 | -2.0576 | 0.6534 |
|  |  |  | chloroplastic/amyloplastic |  |  |  |  |
| C_3 | Gh_A08G1003 | NA | NA | -0.6669 | -0.4293 | -1.0289 | 0.0231 |
| C_3 | Gh_A08G1074 | IAA20 | Auxin-responsive protein IAA20 | -1.0758 | -1.9890 | -5.4134 | -0.2016 |
| C_3 | Gh_A08G1210 | NA | NA | 0.5480 | 0.0850 | -1.0005 | -0.6413 |
| C_3 | Gh_A08G1248 | VAD1 | Protein VASCULAR ASSOCIATED DEATH 1, | -0.2511 | 0.0506 | -0.4277 | -0.1814 |
|  |  |  | chloroplastic |  |  |  |  |
| C_3 | Gh_A08G1420 | AHL23 | AT-hook motif nuclear-localized protein 23 | 0.1329 | 0.0118 | -0.4509 | 0.0492 |
| C_3 | Gh_A08G1434 | NPF7.3 | Protein NRT1/ PTR FAMILY 7.3 | 0.2194 | 0.2741 | -0.2776 | 0.1957 |
| C_3 | Gh_A08G1445 | SGPP | Haloacid dehalogenase-like hydrolase domain-containing | 0.7191 | -0.6884 | -3.1891 | -1.9291 |
|  |  |  | protein Sgpp |  |  |  |  |
| C_3 | Gh_A08G1752 | AGO4 | Protein argonaute 4 | -0.1947 | 0.1607 | -0.4578 | 0.1256 |
| C_3 | Gh_A08G1781 | RAX3 | Transcription factor RAX3 | 0.5035 | 0.3193 | -0.8838 | 0.1515 |
| C_3 | Gh_A08G1827 | NA | Pyrophosphate-energized vacuolar membrane proton | -1.4906 | 0.6161 | -1.2981 | 1.0369 |
|  |  |  | pump |  |  |  |  |
| C_3 | Gh_A08G1908 | AKR1 | Probable aldo-keto reductase 1 | 0.4484 | 0.4302 | -0.4485 | 0.6282 |
| C_3 | Gh_A08G2045 | Eif2d | Eukaryotic translation initiation factor 2D | 0.0883 | 0.0522 | -0.3980 | -0.2078 |
| C_3 | Gh_A08G2075 | NA | Olee1-like protein | 0.3867 | 0.6796 | -2.2132 | -0.1586 |
| C_3 | Gh_A08G2423 | MAKR5 | Probable membrane-associated kinase regulator 5 | -0.1584 | 0.7943 | -1.0268 | 1.1355 |
| C_3 | Gh_A08G2500 | PMA4 | Plasma membrane ATPase 4 | -0.2753 | -0.7473 | -1.1337 | -0.2006 |
| C_3 | Gh_A09G0058 | NA | Transcription factor HBP-1b(c1) (Fragment) | -0.8263 | 0.2939 | -1.4824 | 1.3892 |
| C_3 | Gh_A09G0111 | CNGC2 | Cyclic nucleotide-gated ion channel 2 | -0.3454 | 0.0680 | -1.7912 | 0.6155 |
| C_3 | Gh_A09G0304 | TPS10 | Terpene synthase 10 | -3.8000 | -1.2963 | -2.8837 | -0.4458 |
| C_3 | Gh_A09G0460 | NA | NA | 0.3205 | 0.7655 | -1.2705 | 0.2608 |
| C_3 | Gh_A09G0478 | MYB108 | Transcription factor MYB108 | -1.3954 | -0.1733 | -0.8356 | 1.2554 |
| C_3 | Gh_A09G0642 | IMPA4 | Importin subunit alpha-4 | 0.4664 | -0.3583 | -2.0505 | -0.4588 |
| C_3 | Gh_A09G0689 | TSJT1 | Stem-specific protein TSJT1 | -0.8515 | 0.3744 | -0.9372 | 0.0601 |
| C_3 | Gh_A09G0939 | LHW | Transcription factor LHW | 0.3359 | 0.6737 | -0.2672 | 0.2029 |
| C_3 | Gh_A09G1092 | LCMT1 | Leucine carboxyl methyltransferase 1 | 0.2477 | -2.7272 | -3.8133 | -0.7631 |
| C_3 | Gh_A09G1126 | GNS1 | Glucan endo-1,3-beta-glucosidase, basic isoform | 5.4453 | 4.5090 | -1.1408 | 4.6129 |
| C_3 | Gh_A09G1182 | PGIP | Polygalacturonase inhibitor | -0.7780 | -0.2776 | -1.1835 | -0.6217 |
| C_3 | Gh_A09G1282 | TLDC1 | TLD domain-containing protein 1 | -0.1053 | 0.1594 | -1.2451 | -0.1847 |
| C_3 | Gh_A09G1330 | NA | Tropinone reductase-like 1 | 0.8807 | 0.6446 | -1.2024 | 2.8732 |
| C_3 | Gh_A09G1646 | PYL8 | Abscisic acid receptor PYL8 | -0.2230 | -0.0262 | -0.8061 | -0.3006 |
| C_3 | Gh_A09G1689 | NLP7 | Protein NLP7 | -0.0113 | 0.5814 | -0.7269 | -0.0214 |
| C_3 | Gh_A09G1899 | At2g32040 | Folate-biopterin transporter 1, chloroplastic | -0.5525 | -0.2594 | -0.4408 | -0.1033 |
| C_3 | Gh_A09G2135 | At1g48100 | Polygalacturonase | -1.2453 | 0.9826 | -3.0206 | 0.2153 |
| C_3 | Gh_A09G2221 | FAO1 | Long-chain-alcohol oxidase FAO1 | -0.4300 | 0.1676 | -0.7077 | 0.9459 |
| C_3 | Gh_A09G2422 | TBL35 | Protein trichome birefringence-like 35 | -0.1833 | 0.5131 | -0.3561 | 0.3295 |
| C_3 | Gh_A10G0059 | NA | Tetrahydrocannabinolic acid synthase | -1.2794 | -1.0362 | -2.4995 | 1.0413 |
| C_3 | Gh_A10G0060 | At4g20830 | Reticuline oxidase-like protein | -0.5099 | -0.7542 | -3.2317 | 0.9094 |
| C_3 | Gh_A10G0114 | APA1 | Aspartic proteinase A1 | 0.0779 | 0.2794 | 0.0486 | 0.3299 |
| C_3 | Gh_A10G0153 | RGA4 | Putative disease resistance protein RGA4 | -0.5817 | 0.1895 | -0.4552 | 0.4384 |
| C_3 | Gh_A10G0218 | NA | NA | -0.1187 | 0.2558 | -0.7082 | -0.2372 |
| C_3 | Gh_A10G0460 | SRF6 | Protein STRUBBELIG-RECEPTOR FAMILY 6 | -0.2359 | 1.0251 | -0.0265 | 0.6144 |
| C_3 | Gh_A10G0735 | PME51 | Probable pectinesterase/pectinesterase inhibitor 51 | -0.0299 | 0.6814 | 0.3046 | 0.8507 |
| C_3 | Gh_A10G0767 | NA | NA | 0.5302 | -0.0641 | -0.2971 | -0.0302 |
| C_3 | Gh_A10G0821 | At3g50210 | Probable 2-oxoglutarate-dependent dioxygenase | -0.3668 | 0.0480 | -0.4720 | -0.0959 |
| C_3 | Gh_A10G0826 | NA | NA | -0.0351 | -0.1187 | -1.3951 | -0.5781 |
| C_3 | Gh_A10G0827 | HSF24 | Heat shock factor protein HSF24 | -0.1538 | 0.4375 | -0.9326 | -0.2000 |
| C_3 | Gh_A10G0836 | NA | L-ascorbate oxidase homolog | 0.0737 | 0.7135 | -2.8792 | 1.3319 |
| C_3 | Gh_A10G1202 | SLC35F1 | Solute carrier family 35 member F1 | 0.3538 | 0.3415 | -3.7403 | -2.1373 |
| C_3 | Gh_A10G1218 | SPPL5 | Signal peptide peptidase-like 5 | 0.2427 | 0.4300 | -1.7302 | 0.0184 |
| C_3 | Gh_A10G1307 | UGT74E2 | UDP-glycosyltransferase 74E2 | -0.8091 | -0.6226 | -1.0272 | -0.3559 |
| C_3 | Gh_A10G1361 | ATPK2 | Serine/threonine-protein kinase AtPK2/AtPK19 | -0.4835 | 0.2347 | -0.2243 | 0.0942 |
| C_3 | Gh_A10G1552 | C/VIF1 | Cell wall / vacuolar inhibitor of fructosidase 1 | -1.0373 | -0.5955 | -2.5616 | 0.3592 |
| C_3 | Gh_A10G1744 | CYP94A1 | Cytochrome P450 94A1 | -0.8946 | 0.7902 | -1.9473 | -0.6861 |
| C_3 | Gh_A10G1908 | At2g01680 | Ankyrin repeat-containing protein | -0.6053 | -1.7448 | -6.2398 | -0.7588 |
| C_3 | Gh_A10G2137 | PCMP-E28 | Pentatricopeptide repeat-containing protein | 0.7138 | 0.1036 | -3.0816 | -0.7299 |
| C_3 | Gh_A10G2138 | PRA1B1 | PRA1 family protein B1 | -0.5471 | -0.4051 | -0.6398 | -0.0808 |
| C_3 | Gh_A10G2170 | NA | NA | -0.5016 | 1.3755 | -0.5322 | 1.2259 |
| C_3 | Gh_A10G2231 | ABCB19 | ABC transporter B family member 19 | -0.4860 | -0.6389 | -1.2442 | -0.0575 |
| C_3 | Gh_A10G2306 | AAE5 | Probable acyl-activating enzyme 5, peroxisomal | 0.5921 | 0.2123 | -0.8361 | 1.1964 |
| C_3 | Gh_A10G2319 | SHR | Protein SHORT-ROOT | 0.6024 | 0.2176 | -0.4284 | 0.1378 |
| C_3 | Gh_A11G0149 | NA | NA | -0.2014 | 0.4924 | -0.0303 | 0.2735 |
| C_3 | Gh_A11G0366 | ERF2 | Ethylene-responsive transcription factor 2 | -0.9054 | 2.1020 | 0.4699 | 1.5475 |
| C_3 | Gh_A11G0394 | STR18 | Thiosulfate sulfurtransferase 18 | -0.1540 | 0.7703 | -0.4823 | 0.5694 |
| C_3 | Gh_A11G0409 | PLT6 | Probable polyol transporter 6 | -0.5074 | -0.6956 | -1.1811 | 0.0802 |
| C_3 | Gh_A11G0521 | ADH1 | Alcohol dehydrogenase 1 | 0.1847 | 1.3265 | -2.1559 | 0.2379 |
| C_3 | Gh_A11G0541 | FAAH | Fatty acid amide hydrolase | -0.2334 | -0.3790 | -0.5197 | -0.3355 |
| C_3 | Gh_A11G0984 | WRKY23 | Probable WRKY transcription factor 23 | -0.0952 | 1.7153 | -0.9087 | 0.4765 |
| C_3 | Gh_A11G1024 | EXL2 | Protein EXORDIUM-like 2 | -1.9874 | 2.3059 | -3.7906 | 0.1051 |
| C_3 | Gh_A11G1092 | AHP5 | Histidine-containing phosphotransfer protein 5 | 0.2258 | 0.6736 | 0.3437 | 0.5875 |
| C_3 | Gh_A11G1096 | NA | NA | -0.6115 | 0.6831 | -0.4181 | 1.2913 |
| C_3 | Gh_A11G1197 | H6H | Hyoscyamine 6-dioxygenase | -0.0521 | -0.2674 | -0.7393 | -0.1733 |
| C_3 | Gh_A11G1337 | FH1 | Formin-like protein 1 | 0.1662 | 0.0850 | 0.0756 | 0.2717 |
| C_3 | Gh_A11G1399 | NA | NA | -0.4600 | 1.6107 | -1.5088 | -0.0647 |
| C_3 | Gh_A11G1400 | At3g07870 | F-box protein | -2.9314 | 1.5112 | -4.1972 | 1.7309 |
| C_3 | Gh_A11G1441 | ZIP5 | Zinc transporter 5 | -0.3476 | 0.1683 | -0.7194 | -0.0733 |
| C_3 | Gh_A11G1442 | CCT1 | Choline-phosphate cytidylyltransferase 1 | -0.3548 | -0.3154 | -1.0676 | -0.1781 |
| C_3 | Gh_A11G1730 | BLH2 | BEL1-like homeodomain protein 2 | 0.1010 | 0.0759 | -1.4723 | -0.5419 |
| C_3 | Gh_A11G1800 | IRX15-L | Protein IRX15-LIKE | -1.0159 | 0.4759 | -0.3350 | 0.4903 |
| C_3 | Gh_A11G1835 | NA | NA | 0.1526 | -0.0184 | -1.9531 | 0.3958 |
| C_3 | Gh_A11G1873 | CAD | Probable mannitol dehydrogenase | -0.1789 | -0.5748 | -1.1967 | 0.3031 |
| C_3 | Gh_A11G1891 | NA | NA | 0.1233 | 0.4668 | -0.5763 | 0.1581 |
| C_3 | Gh_A11G1904 | NA | NA | -1.7541 | 1.4743 | -0.9236 | 2.2319 |
| C_3 | Gh_A11G2118 | FRL3 | FRIGIDA-like protein 3 | 0.2064 | 0.0636 | -1.8230 | 0.1484 |
| C_3 | Gh_A11G2128 | HEXO2 | Beta-hexosaminidase 2 | 0.0818 | 0.1205 | -0.5544 | 1.0915 |
| C_3 | Gh_A11G2138 | NA | NA | -0.0992 | 0.0873 | -0.2144 | -0.0157 |
| C_3 | Gh_A11G2142 | NA | NA | -0.1375 | -0.9557 | -1.9766 | 0.4944 |
| C_3 | Gh_A11G2143 | NA | NA | -1.4688 | -0.5867 | -4.0963 | -0.2385 |
| C_3 | Gh_A11G2218 | NA | NA | -0.0917 | 0.4955 | -0.3896 | 0.1923 |
| C_3 | Gh_A11G2337 | GATA9 | GATA transcription factor 9 | -0.0825 | 1.5306 | -0.9552 | 0.2235 |
| C_3 | Gh_A11G2417 | IDD2 | Protein indeterminate-domain 2 | 0.5334 | 0.1640 | -0.3109 | 0.5889 |
| C_3 | Gh_A11G2459 | PPC6-7 | Probable protein phosphatase 2C 73 | 0.0600 | 0.3730 | -1.0364 | -0.1275 |
| C_3 | Gh_A11G2493 | CpNIFS3 | Probable L-cysteine desulfhydrase, chloroplastic | -0.2530 | -0.3386 | -0.8817 | 0.2571 |
| C_3 | Gh_A11G2533 | At1g54290 | Protein translation factor SUI1 homolog 2 | 0.0415 | 0.4847 | -0.5504 | 0.2152 |
| C_3 | Gh_A11G2538 | CAD1-A | (+)-delta-cadinene synthase isozyme A | 1.4587 | 0.5616 | -3.2742 | -0.2348 |
| C_3 | Gh_A11G2566 | NA | Metallothionein-like protein type 3 | -0.2239 | 0.7877 | -1.2118 | 0.2417 |
| C_3 | Gh_A11G2702 | NCS2 | S-norcoclaurine synthase 2 | 2.0063 | 1.4660 | -4.3771 | 0.3749 |
| C_3 | Gh_A11G2734 | CAMBP25 | Calmodulin-binding protein 25 | -0.0275 | -1.2318 | -0.8927 | 0.9515 |
| C_3 | Gh_A11G2761 | At3g47200 | UPF0481 protein | 0.8534 | -0.4788 | -1.5416 | -1.1707 |
| C_3 | Gh_A11G2773 | NA | NA | 0.1064 | -0.0432 | -4.9446 | -1.2446 |
| C_3 | Gh_A11G2790 | NA | NA | -0.1872 | -0.0163 | -0.0860 | 0.4831 |
| C_3 | Gh_A11G2828 | NFYA10 | Nuclear transcription factor Y subunit A-10 | -1.7459 | -0.2321 | -1.7887 | 0.0215 |
| C_3 | Gh_A11G2836 | RPPL1 | Putative disease resistance RPP13-like protein 1 | 0.2933 | -1.3546 | -2.0666 | -1.0296 |
| C_3 | Gh_A11G3050 | URT1 | UTP:RNA uridylyltransferase 1 | 0.1868 | -0.0344 | -0.2577 | -0.1063 |
| C_3 | Gh_A11G3060 | QKY | Protein QUIRKY | -0.1049 | -0.4664 | -0.7853 | 0.0475 |
| C_3 | Gh_A11G3076 | NA | NA | 0.0107 | 0.9538 | 0.0183 | 0.6359 |
| C_3 | Gh_A11G3098 | AAE11 | Butyrate--CoA ligase AAE11, peroxisomal | -0.3270 | -0.1742 | -0.5565 | -0.0673 |
| C_3 | Gh_A11G3297 | NA | NA | -0.3732 | 0.6667 | -9.7297 | -0.1352 |
| C_3 | Gh_A12G0153 | FAAH | Fatty acid amide hydrolase | -0.4767 | 0.6351 | -0.2375 | 0.4939 |
| C_3 | Gh_A12G0196 | At4g34220 | Receptor protein kinase-like protein | 0.7778 | 0.3626 | -2.2648 | 1.2557 |
| C_3 | Gh_A12G0274 | At2g14610 | Pathogenesis-related protein 1 | -0.0329 | 2.3925 | -0.7805 | 2.2199 |
| C_3 | Gh_A12G0391 | DRD1 | Protein chromatin remodeling 35 | 0.0720 | -0.2216 | -0.5376 | -0.3979 |
| C_3 | Gh_A12G0410 | BAM1 | Leucine-rich repeat receptor-like serine/threonine-protein | -0.3858 | 0.2867 | -1.3857 | -0.1637 |
|  |  |  | kinase BAM1 |  |  |  |  |
| C_3 | Gh_A12G0455 | GT6 | UDP-glucose flavonoid 3-O-glucosyltransferase 6 | 0.4481 | -1.0761 | -1.8641 | -0.4760 |
| C_3 | Gh_A12G0460 | NA | NA | 3.1197 | 3.6162 | -2.2059 | 0.4448 |
| C_3 | Gh_A12G0827 | CAJ1 | Protein CAJ1 | -0.7801 | 0.1487 | -2.1453 | 0.9161 |
| C_3 | Gh_A12G1082 | chmp7 | Charged multivesicular body protein 7 | -0.0366 | 0.2289 | -0.6784 | -0.1515 |
| C_3 | Gh_A12G1091 | ABCB4 | ABC transporter B family member 4 | -1.6759 | 0.7625 | -0.5629 | 1.2660 |
| C_3 | Gh_A12G1195 | BAH1 | E3 ubiquitin-protein ligase BAH1 | -0.3748 | 0.3558 | -0.2728 | 0.3409 |
| C_3 | Gh_A12G1328 | CRK2 | Cysteine-rich receptor-like protein kinase 2 | 0.1128 | 1.6108 | 0.6543 | 1.6503 |
| C_3 | Gh_A12G1488 | WRKY57 | Probable WRKY transcription factor 57 | -0.8514 | 1.4346 | -2.2840 | 0.3913 |
| C_3 | Gh_A12G1556 | STN7 | Serine/threonine-protein kinase STN7, chloroplastic | -0.0525 | -0.0499 | -0.0893 | 0.0860 |
| C_3 | Gh_A12G1574 | NA | NA | -0.4414 | 0.3266 | -2.7891 | -1.4852 |
| C_3 | Gh_A12G1591 | NFD4 | Protein NUCLEAR FUSION DEFECTIVE 4 | -0.9015 | -1.4443 | -1.4121 | -0.7438 |
| C_3 | Gh_A12G1670 | SARD1 | Protein SAR DEFICIENT 1 | -0.6130 | 1.1660 | -0.3991 | 0.5673 |
| C_3 | Gh_A12G1766 | NA | NA | -1.0049 | -0.7678 | -1.1542 | -0.3925 |
| C_3 | Gh_A12G2002 | At4g12490 | pEARLI1-like lipid transfer protein 2 | -1.0433 | 1.6902 | -1.6674 | 1.3690 |
| C_3 | Gh_A12G2016 | CYP93A1 | 3,9-dihydroxypterocarpan 6A-monooxygenase | 0.5429 | 0.2154 | -0.0968 | 0.3887 |
| C_3 | Gh_A12G2134 | NA | NA | -1.5000 | -0.2670 | -4.3468 | -1.1425 |
| C_3 | Gh_A12G2227 | OMT | Caffeic acid 3-O-methyltransferase | -0.0540 | -0.1101 | -0.3482 | 0.3739 |
| C_3 | Gh_A12G2336 | MBD13 | Methyl-CpG-binding domain-containing protein 13 | 0.4000 | -0.0841 | -0.2154 | 0.3374 |
| C_3 | Gh_A12G2427 | NA | Probable non-specific lipid-transfer protein AKCS9 | -0.0383 | 1.7637 | -0.2447 | 0.9390 |
| C_3 | Gh_A12G2430 | NMT1 | Phosphoethanolamine N-methyltransferase 1 | 0.1361 | -0.3292 | -1.1519 | -0.6533 |
| C_3 | Gh_A12G2461 | NA | Glutamate decarboxylase | -0.3804 | 0.9828 | -1.3917 | 0.5293 |
| C_3 | Gh_A12G2478 | NA | Carbonic anhydrase, chloroplastic | -0.2452 | -0.1477 | -0.2372 | -0.0566 |
| C_3 | Gh_A12G2517 | At2g13820 | Non-specific lipid-transfer protein-like protein | -1.1200 | 0.1706 | -2.3374 | 1.0124 |
| C_3 | Gh_A12G2605 | SDR1 | (+)-neomenthol dehydrogenase | 0.4409 | 0.0899 | -0.5474 | 0.1492 |
| C_3 | Gh_A13G0052 | RPS26A | 40S ribosomal protein S26-1 | 0.1205 | 0.1100 | 0.0818 | 0.2037 |
| C_3 | Gh_A13G0087 | ACR12 | ACT domain-containing protein ACR12 | -0.3089 | -0.4658 | -0.6328 | 0.0467 |
| C_3 | Gh_A13G0171 | NA | NA | -0.1003 | -0.2227 | -1.4825 | -0.7774 |
| C_3 | Gh_A13G0173 | UGT91C1 | UDP-glycosyltransferase 91C1 | -0.1683 | 0.1498 | -2.0832 | 1.6255 |
| C_3 | Gh_A13G0187 | NA | Actin | 1.5276 | 0.7532 | -4.1283 | -0.0564 |
| C_3 | Gh_A13G0290 | BAHCC1 | BAH and coiled-coil domain-containing protein 1 | -0.7086 | 0.1194 | -1.7324 | 0.5308 |
| C_3 | Gh_A13G0459 | NPF3.1 | Protein NRT1/ PTR FAMILY 3.1 | -1.0255 | -0.1505 | -1.2501 | 1.5210 |
| C_3 | Gh_A13G0594 | NA | NA | -0.0699 | 0.9251 | -0.3232 | 0.2982 |
| C_3 | Gh_A13G0963 | Gba2 | Non-lysosomal glucosylceramidase | 0.0147 | 0.0129 | -0.0175 | 0.1984 |
| C_3 | Gh_A13G1377 | yabD | Uncharacterized deoxyribonuclease YabD | 0.1716 | 0.4958 | -1.6999 | 0.6418 |
| C_3 | Gh_A13G1463 | COMT1 | Caffeic acid 3-O-methyltransferase | -0.3707 | -0.4713 | -0.8035 | -0.4611 |
| C_3 | Gh_A13G1595 | KNAT3 | Homeobox protein knotted-1-like 3 | 0.0593 | -0.6177 | -0.7641 | 0.9808 |
| C_3 | Gh_A13G1650 | HDA2 | Histone deacetylase 2 | -0.0781 | 0.4304 | -0.5985 | -0.0207 |
| C_3 | Gh_A13G1728 | At4g29190 | Zinc finger CCCH domain-containing protein 49 | -0.8617 | 0.4915 | -3.9789 | -0.4051 |
| C_3 | Gh_A13G1792 | AOP1 | Probable 2-oxoglutarate-dependent dioxygenase AOP1 | 0.2839 | 0.0591 | -0.5982 | 0.0518 |
| C_3 | Gh_A13G1904 | EPHX2 | Bifunctional epoxide hydrolase 2 | -0.8125 | -0.3358 | -1.5630 | -0.3340 |
| C_3 | Gh_A13G1919 | FLA12 | Fasciclin-like arabinogalactan protein 12 | -0.9211 | 0.3441 | -0.7606 | 0.8264 |
| C_3 | Gh_A13G1952 | Nudcd2 | NudC domain-containing protein 2 | -0.1188 | 0.3018 | -0.8341 | -0.1183 |
| C_3 | Gh_A13G2107 | At2g23090 | Uncharacterized protein At2g23090 | -0.2196 | -0.1685 | -0.9733 | -0.6721 |
| C_3 | Gh_A13G2187 | SAUR24 | Auxin-responsive protein SAUR24 | 0.1900 | 1.8872 | 0.9966 | 1.9812 |
| C_3 | Gh_A13G2278 | BZIP43 | Basic leucine zipper 43 | -0.9334 | 0.7460 | -1.0610 | 1.4256 |
| C_3 | Gh_D01G0130 | MSSP2 | Monosaccharide-sensing protein 2 | 0.5670 | -0.2069 | -0.4841 | -0.1259 |
| C_3 | Gh_D01G0215 | LACS7 | Long chain acyl-CoA synthetase 7, peroxisomal | -0.2423 | -0.1658 | -0.7099 | -0.1513 |
| C_3 | Gh_D01G0381 | DIR15 | Dirigent protein 15 | 0.7408 | -1.9511 | -2.7446 | 0.0161 |
| C_3 | Gh_D01G0411 | UBA2C | UBP1-associated protein 2C | 0.1419 | -0.4469 | -0.6690 | -0.2261 |
| C_3 | Gh_D01G1197 | CRTISO | Prolycopene isomerase, chloroplastic | -0.2376 | 0.1939 | -0.9383 | 0.0841 |
| C_3 | Gh_D01G1332 | SGR5 | Protein SHOOT GRAVITROPISM 5 | -0.1000 | -0.8461 | -1.3239 | -0.6467 |
| C_3 | Gh_D01G1471 | PIN3 | Auxin efflux carrier component 3 | -1.0172 | -0.8932 | -1.7675 | -0.2539 |
| C_3 | Gh_D01G1760 | POT5 | Potassium transporter 5 | 0.2941 | -0.8358 | -1.3679 | -1.1264 |
| C_3 | Gh_D01G1838 | VQ17 | VQ motif-containing protein 17 | 0.3076 | 0.3512 | -0.2358 | 0.4335 |
| C_3 | Gh_D01G2119 | GSTL3 | Glutathione S-transferase L3 | -1.2143 | -0.0245 | -0.9109 | 0.2386 |
| C_3 | Gh_D01G2154 | BLH9 | BEL1-like homeodomain protein 9 | 0.0863 | -1.2223 | -3.6991 | 0.9240 |
| C_3 | Gh_D01G2160 | LHW | Transcription factor LHW | 0.8329 | 0.2842 | -0.0242 | 0.0385 |
| C_3 | Gh_D01G2301 | C/VIF1 | Cell wall / vacuolar inhibitor of fructosidase 1 | -0.0679 | 1.3710 | 0.2662 | 2.2358 |
| C_3 | Gh_D01G2303 | NA | NA | 0.2078 | -0.1369 | -0.6996 | 0.1281 |
| C_3 | Gh_D02G0069 | At3g08860 | Alanine--glyoxylate aminotransferase 2 homolog 3, | -1.2612 | 0.0927 | -2.9814 | -0.2024 |
|  |  |  | mitochondrial |  |  |  |  |
| C_3 | Gh_D02G0208 | SEC23 | Protein transport protein SEC23 | -0.0867 | 0.1974 | -0.2250 | 0.1974 |
| C_3 | Gh_D02G0213 | NA | Tetrahydrocannabinolic acid synthase | -0.5123 | -1.2889 | -1.4572 | -0.0938 |
| C_3 | Gh_D02G0217 | CBDAS2 | Cannabidiolic acid synthase-like 1 | -0.2311 | -0.1727 | -0.5890 | 0.9661 |
| C_3 | Gh_D02G0220 | NA | Xyloglucan endotransglucosylase/hydrolase 2 | -0.6695 | -0.1884 | -0.4762 | -0.2482 |
| C_3 | Gh_D02G0243 | SBP2 | Selenium-binding protein 2 | -0.7552 | -0.1146 | -1.1951 | -0.0023 |
| C_3 | Gh_D02G0265 | PHT1-5 | Probable inorganic phosphate transporter 1-5 | 1.2915 | 1.0482 | -1.4291 | -0.3123 |
| C_3 | Gh_D02G0315 | GSTU7 | Glutathione S-transferase U7 | -0.4756 | 0.4646 | -0.1580 | 0.4531 |
| C_3 | Gh_D02G0376 | NA | NA | -0.1255 | 0.0109 | -0.0901 | 0.1395 |
| C_3 | Gh_D02G0378 | HSP22.7 | 22.7 kDa class IV heat shock protein | -6.9337 | -0.2629 | -5.9212 | -1.8798 |
| C_3 | Gh_D02G0599 | EDR1 | Serine/threonine-protein kinase EDR1 | -0.0888 | 0.7411 | -1.6894 | 0.0990 |
| C_3 | Gh_D02G0612 | NA | NA | -0.6005 | -1.3939 | -1.1626 | 3.4739 |
| C_3 | Gh_D02G0724 | RCH1 | LRR receptor-like serine/threonine-protein kinase RCH1 | -1.0481 | 1.5061 | -3.6820 | 0.4424 |
| C_3 | Gh_D02G0733 | ADH1 | Alcohol dehydrogenase class-P | -0.2761 | 0.8255 | -0.0666 | 0.7285 |
| C_3 | Gh_D02G0786 | tolB | Protein TolB | -1.1120 | -1.3411 | -1.9539 | -0.3955 |
| C_3 | Gh_D02G0888 | NA | NA | 0.0968 | 0.1187 | -0.6645 | -0.3540 |
| C_3 | Gh_D02G0995 | SBEII | 1,4-alpha-glucan-branching enzyme 1, | -1.1130 | 0.0858 | -1.2177 | 0.5171 |
|  |  |  | chloroplastic/amyloplastic |  |  |  |  |
| C_3 | Gh_D02G1236 | NA | NA | -1.1394 | -1.8468 | -2.7397 | -0.9261 |
| C_3 | Gh_D02G1311 | AP1C | Floral homeotic protein APETALA 1 C | 0.6537 | 2.0797 | 0.7389 | 4.9332 |
| C_3 | Gh_D02G1378 | IP5P9 | Type IV inositol polyphosphate 5-phosphatase 9 | -0.6110 | -0.6236 | -1.6309 | -0.9411 |
| C_3 | Gh_D02G1405 | BHLH111 | Transcription factor bHLH111 | -0.7260 | -0.4708 | -1.4169 | -0.0051 |
| C_3 | Gh_D02G1613 | PIRL3 | Plant intracellular Ras-group-related LRR protein 3 | 0.3521 | 0.4099 | -0.3537 | -0.0103 |
| C_3 | Gh_D02G1702 | WVD2 | Protein WAVE-DAMPENED 2 | -0.5108 | 0.4923 | -0.1683 | 0.2485 |
| C_3 | Gh_D02G1951 | ABCB19 | ABC transporter B family member 19 | -0.0684 | 0.6306 | -6.3384 | 0.7731 |
| C_3 | Gh_D02G2290 | Agpat9 | Glycerol-3-phosphate acyltransferase 3 | -0.1108 | 0.2940 | -1.2477 | 0.2351 |
| C_3 | Gh_D02G2341 | NA | NA | -1.1391 | 1.1482 | -3.3893 | 1.0371 |
| C_3 | Gh_D02G2366 | PSAK | Photosystem I reaction center subunit psaK, chloroplastic | 0.3505 | 2.2643 | -1.1680 | 2.7801 |
| C_3 | Gh_D02G2442 | CXE7 | Probable carboxylesterase 7 | -0.3465 | -1.6442 | -2.6089 | -1.0486 |
| C_3 | Gh_D03G0004 | YBR287W | Uncharacterized transporter YBR287W | -1.0907 | 0.4007 | -0.3828 | 1.1125 |
| C_3 | Gh_D03G0022 | DSK2B | Ubiquitin domain-containing protein DSK2b | -0.3832 | -0.4192 | -1.1134 | -0.0860 |
| C_3 | Gh_D03G0168 | Os03g0199100 | UPF0496 protein 1 | 1.5279 | 3.0631 | -5.1723 | -0.2757 |
| C_3 | Gh_D03G0280 | BZIP43 | Basic leucine zipper 43 | 0.8819 | -0.2567 | -1.7049 | -0.3505 |
| C_3 | Gh_D03G0302 | BAM1 | Leucine-rich repeat receptor-like serine/threonine-protein | -0.0014 | -0.0042 | -1.0776 | -0.0621 |
|  |  |  | kinase BAM1 |  |  |  |  |
| C_3 | Gh_D03G0335 | At5g65490 | Protein ecdysoneless homolog | 0.3263 | -0.0389 | -0.7289 | -0.2784 |
| C_3 | Gh_D03G0375 | BHLH96 | Transcription factor bHLH96 | -0.5167 | 0.6216 | -0.0545 | 1.3650 |
| C_3 | Gh_D03G0408 | NA | Inositol-3-phosphate synthase | -0.0335 | 0.0808 | -0.0431 | 0.3071 |
| C_3 | Gh_D03G0516 | At3g02910 | Putative gamma-glutamylcyclotransferase | -0.6126 | 1.7727 | -3.4249 | -0.7684 |
| C_3 | Gh_D03G0531 | CDI | Protein CDI | -0.1219 | 1.0761 | 0.3293 | 0.8587 |
| C_3 | Gh_D03G0588 | S-ACP-DES6 | Stearoyl-[acyl-carrier-protein] 9-desaturase 6, | -0.3030 | 0.7651 | 0.2815 | 0.8735 |
|  |  |  | chloroplastic |  |  |  |  |
| C_3 | Gh_D03G0625 | MIZ1 | Protein MIZU-KUSSEI 1 | -0.7022 | -1.5485 | -1.4223 | -0.2488 |
| C_3 | Gh_D03G0682 | WRKY29 | Probable WRKY transcription factor 29 | -0.1002 | -0.9328 | -1.0283 | -0.0891 |
| C_3 | Gh_D03G0818 | MTP1 | Metal tolerance protein 1 | -0.8272 | 0.7267 | -0.3184 | 0.3363 |
| C_3 | Gh_D03G0836 | NA | Transcription factor MYB1R1 | 1.5728 | 0.4437 | -1.8765 | -0.9798 |
| C_3 | Gh_D03G0958 | NA | NA | -0.5088 | 0.3408 | -0.3177 | 0.0953 |
| C_3 | Gh_D03G1049 | AGD1 | ADP-ribosylation factor GTPase-activating protein AGD1 | 0.6459 | 0.2102 | -3.3838 | 0.3337 |
| C_3 | Gh_D03G1099 | WDR44 | WD repeat-containing protein 44 | -0.8513 | -0.7664 | -1.4485 | -0.9053 |
| C_3 | Gh_D03G1203 | CTIMC | Triosephosphate isomerase, cytosolic | 0.0500 | -0.3505 | -0.5865 | 0.0080 |
| C_3 | Gh_D03G1264 | HERK1 | Receptor-like protein kinase HERK 1 | -0.5423 | 1.3738 | -0.3056 | 0.9163 |
| C_3 | Gh_D03G1266 | ECA1 | Calcium-transporting ATPase 1, endoplasmic reticulum- | 2.1140 | 0.7921 | -0.7378 | 0.1322 |
|  |  |  | type |  |  |  |  |
| C_3 | Gh_D03G1342 | At2g23060 | Probable N-acetyltransferase HLS1-like | 0.0131 | -0.2313 | -1.9689 | -1.1855 |
| C_3 | Gh_D03G1385 | CIPK10 | CBL-interacting serine/threonine-protein kinase 10 | -0.8788 | -0.0278 | -0.4003 | 0.1741 |
| C_3 | Gh_D03G1454 | TUBB1 | Tubulin beta-1 chain | 0.0732 | 0.2881 | -0.3397 | 0.6309 |
| C_3 | Gh_D03G1455 | NA | Tubulin beta-9 chain | -0.2106 | 1.0140 | -0.3470 | 1.1053 |
| C_3 | Gh_D03G1462 | tlp | Thaumatin-like protein | -0.0744 | -0.6253 | -1.0181 | 2.2632 |
| C_3 | Gh_D03G1533 | NA | NA | -3.2190 | 0.2929 | -4.1628 | -0.0468 |
| C_3 | Gh_D03G1546 | NA | NA | 0.0924 | 0.0248 | -4.3569 | -0.2142 |
| C_3 | Gh_D03G1554 | NPF6.3 | Protein NRT1/ PTR FAMILY 6.3 | -1.8591 | -0.0274 | -1.5308 | 0.1022 |
| C_3 | Gh_D03G1616 | BRG3 | Probable BOI-related E3 ubiquitin-protein ligase 3 | 0.2083 | -0.3639 | -0.6945 | -0.2953 |
| C_3 | Gh_D03G1646 | SYP22 | Syntaxin-22 | -0.2484 | 0.6069 | -0.2292 | 0.4698 |
| C_3 | Gh_D03G1675 | RTNLB13 | Reticulon-like protein B13 | 0.5746 | -1.1937 | -4.8605 | -0.7395 |
| C_3 | Gh_D03G1842 | At1g32060 | Phosphoribulokinase, chloroplastic | -1.6078 | 0.2188 | -0.5617 | 1.8449 |
| C_3 | Gh_D04G0053 | NA | Malate dehydrogenase [NADP], chloroplastic | -0.6057 | 1.3610 | -2.9423 | 0.7583 |
| C_3 | Gh_D04G0054 | 4CLL7 | 4-coumarate--CoA ligase-like 7 | -2.2108 | -2.2578 | -4.3143 | 0.8300 |
| C_3 | Gh_D04G0109 | At5g05200 | Uncharacterized aarF domain-containing protein kinase | -0.2266 | -0.7491 | -0.7344 | -0.2084 |
| C_3 | Gh_D04G0124 | AZG1 | Adenine/guanine permease AZG1 | 0.9239 | 0.1123 | -0.3829 | -0.3168 |
| C_3 | Gh_D04G0125 | At2g40480 | WEB family protein | 0.5973 | 1.1295 | -1.1062 | 0.0207 |
| C_3 | Gh_D04G0235 | NA | NA | 0.6045 | -0.8224 | -5.0636 | -0.8095 |
| C_3 | Gh_D04G0459 | GALM | Aldose 1-epimerase | 0.2888 | 0.7286 | 0.0338 | 0.3854 |
| C_3 | Gh_D04G0489 | NPF5.9 | Protein NRT1/ PTR FAMILY 5.9 | -0.7730 | -0.2928 | -1.6861 | -0.6276 |
| C_3 | Gh_D04G0696 | NA | NA | 0.2376 | -0.9054 | -1.7842 | -0.4164 |
| C_3 | Gh_D04G0741 | NA | B2 protein | 0.1381 | 0.5624 | -0.2700 | 0.2149 |
| C_3 | Gh_D04G0899 | SPP2 | Sucrose-phosphatase 2 | -0.0488 | 0.1920 | -0.2723 | 0.4083 |
| C_3 | Gh_D04G1066 | NA | NA | -1.0159 | -0.3086 | -2.1582 | -0.3570 |
| C_3 | Gh_D04G1170 | MT1 | Metallothionein-like protein type 2 | -2.0538 | 0.7705 | -1.3198 | 0.7958 |
| C_3 | Gh_D04G1322 | NA | Polygalacturonase | 0.2276 | -0.7946 | -1.0350 | -0.0272 |
| C_3 | Gh_D04G1465 | ASPG2 | Protein ASPARTIC PROTEASE IN GUARD CELL 2 | 0.2723 | 0.4109 | -0.1504 | 1.6320 |
| C_3 | Gh_D04G1941 | ASIL2 | Trihelix transcription factor ASIL2 | 0.2117 | 0.2819 | -0.3191 | -0.0740 |
| C_3 | Gh_D05G0044 | At1g34300 | G-type lectin S-receptor-like serine/threonine-protein | -0.2454 | -1.7702 | -6.9830 | 0.8028 |
|  |  |  | kinase |  |  |  |  |
| C_3 | Gh_D05G0112 | At2g41040 | Uncharacterized methyltransferase | -0.2094 | -0.4507 | -1.0309 | -0.5441 |
| C_3 | Gh_D05G0114 | NA | NA | 0.3644 | 1.0947 | 0.4152 | 0.9379 |
| C_3 | Gh_D05G0146 | NA | NA | -1.1142 | -0.2083 | -2.0093 | 0.3764 |
| C_3 | Gh_D05G0276 | At4g29190 | Zinc finger CCCH domain-containing protein 49 | -0.7067 | -0.3456 | -1.1976 | 0.8178 |
| C_3 | Gh_D05G0409 | NA | (S)-coclaurine N-methyltransferase | -0.1148 | 0.6870 | -0.1875 | 0.5411 |
| C_3 | Gh_D05G0410 | HAB1 | Protein phosphatase 2C 16 | 0.2293 | -0.0069 | -0.2470 | -0.0688 |
| C_3 | Gh_D05G0478 | At2g25790 | Probably inactive leucine-rich repeat receptor-like protein | -0.0408 | 0.6165 | -0.4550 | 0.1765 |
|  |  |  | kinase |  |  |  |  |
| C_3 | Gh_D05G0489 | DREB3 | Dehydration-responsive element-binding protein 3 | 0.7173 | 0.5459 | 0.5091 | 0.6461 |
| C_3 | Gh_D05G0599 | CIPK5 | CBL-interacting serine/threonine-protein kinase 5 | 0.3405 | 0.6230 | 0.4950 | 0.6365 |
| C_3 | Gh_D05G0646 | CYP714A1 | Cytochrome P450 714A1 | -0.6581 | -0.6119 | -1.5838 | -0.5745 |
| C_3 | Gh_D05G0868 | clpB | Chaperone protein ClpB | -0.8444 | 0.0708 | -1.2799 | 0.3612 |
| C_3 | Gh_D05G0953 | RABE1C | Ras-related protein RABE1c | -0.0782 | 0.4471 | 0.0057 | 0.2812 |
| C_3 | Gh_D05G0989 | sll0103 | Uncharacterized protein sll0103 | 0.1257 | -0.1495 | -0.7468 | -0.0514 |
| C_3 | Gh_D05G1082 | MLO13 | MLO-like protein 13 | 3.2838 | 2.3841 | -1.5950 | 0.0686 |
| C_3 | Gh_D05G1097 | CASBPX1 | Cycloartenol synthase | -1.0150 | 0.6310 | -0.2305 | 0.5000 |
| C_3 | Gh_D05G1204 | SPX1 | SPX domain-containing protein 1 | 0.9193 | -1.2415 | -1.9964 | -0.2798 |
| C_3 | Gh_D05G1287 | IDD7 | Protein indeterminate-domain 7 | -0.2394 | 0.2977 | -0.8307 | -0.0889 |
| C_3 | Gh_D05G1419 | CYP710A2 | Cytochrome P450 710A2 | 0.0337 | -0.3987 | -0.3551 | 0.7570 |
| C_3 | Gh_D05G1510 | HSL1 | Receptor-like protein kinase HSL1 | -0.0496 | 0.9846 | -0.7178 | 0.2246 |
| C_3 | Gh_D05G1543 | CBSX5 | CBS domain-containing protein CBSX5 | -1.0267 | -0.0667 | -0.5039 | 0.0766 |
| C_3 | Gh_D05G1609 | NA | Pectinesterase/pectinesterase inhibitor | 0.5622 | 0.8424 | -1.1692 | -0.2423 |
| C_3 | Gh_D05G1663 | ACO | 1-aminocyclopropane-1-carboxylate oxidase | -0.5692 | -0.7086 | -1.6292 | -0.2120 |
| C_3 | Gh_D05G1710 | CAT1 | Catalase isozyme 1 | -0.0340 | -0.1160 | -0.5490 | -0.2287 |
| C_3 | Gh_D05G1847 | RL1 | Protein RADIALIS-like 1 | -1.3211 | 0.4035 | -1.4708 | 2.2590 |
| C_3 | Gh_D05G1865 | rsc5 | Random slug protein 5 | -0.0456 | -0.0050 | -0.4021 | 0.2567 |
| C_3 | Gh_D05G1957 | At1g01540 | Probable serine/threonine-protein kinase | 0.2645 | 0.5060 | 0.2073 | 0.5120 |
| C_3 | Gh_D05G1970 | RD21A | Cysteine proteinase RD21a | -0.0093 | 0.0979 | -0.2610 | 0.0921 |
| C_3 | Gh_D05G1998 | VEP1 | 3-oxo-Delta(4,5)-steroid 5-beta-reductase | -0.0058 | 0.1284 | -0.0051 | 0.3525 |
| C_3 | Gh_D05G2070 | HSL1 | Receptor-like protein kinase HSL1 | -0.3082 | -0.0356 | -0.6419 | 0.1384 |
| C_3 | Gh_D05G2117 | nep1 | Aspartic proteinase nepenthesin-1 | -0.2930 | 0.2972 | -1.6225 | 1.0881 |
| C_3 | Gh_D05G2125 | BHLH145 | Transcription factor bHLH145 | -0.0087 | -0.0116 | -0.3058 | -0.0337 |
| C_3 | Gh_D05G2141 | ATL54 | RING-H2 finger protein ATL54 | -0.2639 | 0.1934 | -0.7926 | -0.1197 |
| C_3 | Gh_D05G2225 | At4g08300 | WAT1-related protein | 0.1392 | -0.2367 | -0.2912 | 0.4026 |
| C_3 | Gh_D05G2279 | NA | NA | 0.2374 | -0.6776 | -0.9927 | -0.7352 |
| C_3 | Gh_D05G2446 | NA | Auxin-induced protein 6B | 4.6776 | 2.5288 | -4.5342 | 0.0967 |
| C_3 | Gh_D05G2463 | NA | NA | -0.9344 | 1.0270 | -1.8134 | -0.2408 |
| C_3 | Gh_D05G2470 | RL6 | Protein RADIALIS-like 6 | 0.5926 | 1.4774 | -0.1474 | 1.8698 |
| C_3 | Gh_D05G2473 | OFP7 | Transcription repressor OFP7 | -0.2308 | -0.7610 | -2.0616 | -0.3471 |
| C_3 | Gh_D05G2487 | At4g33300 | Probable disease resistance protein | 0.1579 | 0.5640 | 0.0074 | 0.5317 |
| C_3 | Gh_D05G2778 | NA | NA | -2.8455 | 0.7738 | -4.5983 | 1.0701 |
| C_3 | Gh_D05G2834 | EMB1144 | Chorismate synthase, chloroplastic | -0.0404 | 0.1761 | -0.5841 | 0.1497 |
| C_3 | Gh_D05G2909 | PROT2 | Proline transporter 2 | -0.2560 | 0.3372 | -0.1061 | 0.5994 |
| C_3 | Gh_D05G3204 | RPD1 | Protein ROOT PRIMORDIUM DEFECTIVE 1 | 0.6991 | -2.6962 | -3.9159 | -0.6013 |
| C_3 | Gh_D05G3434 | NA | Metallothionein-like protein type 3 | -1.1676 | 1.0440 | -0.7482 | 0.8372 |
| C_3 | Gh_D05G3569 | RPPL1 | Putative disease resistance RPP13-like protein 1 | -0.2628 | 1.0018 | -1.6188 | 0.0996 |
| C_3 | Gh_D05G3615 | NA | Flavonol sulfotransferase-like | -0.1905 | -0.4532 | -1.8402 | 0.2810 |
| C_3 | Gh_D05G3623 | TAF6 | Transcription initiation factor TFIID subunit 6 | -0.1122 | -0.2051 | -0.9506 | -0.1351 |
| C_3 | Gh_D05G3821 | BHLH93 | Transcription factor bHLH93 | -1.3316 | 1.7879 | -3.1997 | 0.8190 |
| C_3 | Gh_D05G3829 | NA | NA | -0.8420 | 1.0507 | -4.0228 | 0.0924 |
| C_3 | Gh_D05G3832 | NA | NA | -0.3336 | 0.7039 | -0.2946 | 0.2318 |
| C_3 | Gh_D06G0033 | HHT1 | Omega-hydroxypalmitate O-feruloyl transferase | -1.1769 | 0.0214 | -1.2547 | -0.5338 |
| C_3 | Gh_D06G0162 | At3g50280 | Uncharacterized acetyltransferase | -0.5419 | -1.9504 | -3.6057 | 0.2719 |
| C_3 | Gh_D06G0289 | ATHB-16 | Homeobox-leucine zipper protein ATHB-16 | -0.2796 | 2.1658 | 0.7550 | 4.2450 |
| C_3 | Gh_D06G0615 | NA | Aspartate aminotransferase, cytoplasmic | -0.1216 | 0.5390 | 0.0775 | 0.4316 |
| C_3 | Gh_D06G0908 | NA | NA | -1.4320 | 0.0208 | -2.3384 | 0.3542 |
| C_3 | Gh_D06G0970 | CCD8B | Carotenoid cleavage dioxygenase 8 homolog B, | -0.0663 | -1.4960 | -2.0989 | -0.8284 |
|  |  |  | chloroplastic |  |  |  |  |
| C_3 | Gh_D06G0980 | CCD1 | Carotenoid 9,10(9',10')-cleavage dioxygenase 1 | -0.1803 | -1.5888 | -2.2447 | -2.0374 |
| C_3 | Gh_D06G1118 | At2g24580 | Probable sarcosine oxidase | -0.7378 | -0.8366 | -1.3530 | -0.2778 |
| C_3 | Gh_D06G1201 | At1g54610 | Probable serine/threonine-protein kinase | 1.3503 | -0.0586 | -3.0068 | -1.3598 |
| C_3 | Gh_D06G1352 | SCPL31 | Serine carboxypeptidase-like 31 | 0.7095 | -0.2301 | -1.2237 | 0.4727 |
| C_3 | Gh_D06G1355 | ICT1 | Peptidyl-tRNA hydrolase ICT1, mitochondrial | 0.0265 | -1.0661 | -2.0968 | -0.2639 |
| C_3 | Gh_D06G1695 | TDT | Tonoplast dicarboxylate transporter | -1.4726 | 0.7829 | -0.6133 | 0.6026 |
| C_3 | Gh_D06G1816 | At2g39510 | WAT1-related protein | -0.4530 | -0.4083 | -0.9015 | 0.0066 |
| C_3 | Gh_D06G1984 | RPS5 | Disease resistance protein RPS5 | 0.0037 | -0.2412 | -0.8222 | -0.3814 |
| C_3 | Gh_D06G2077 | HST | Shikimate O-hydroxycinnamoyltransferase | 0.0813 | -0.5300 | -1.5008 | -0.6812 |
| C_3 | Gh_D06G2078 | HST | Shikimate O-hydroxycinnamoyltransferase | -0.2750 | 0.9778 | -1.9148 | 0.5229 |
| C_3 | Gh_D06G2095 | serinc | Probable serine incorporator | -0.0942 | -0.0162 | -0.2954 | -0.1350 |
| C_3 | Gh_D06G2293 | PAP | Purple acid phosphatase | -2.1681 | -0.8210 | -1.5228 | -0.0507 |
| C_3 | Gh_D07G0079 | ATL42 | E3 ubiquitin-protein ligase ATL42 | -0.9442 | -2.1642 | -3.2402 | -1.5210 |
| C_3 | Gh_D07G0096 | At4g29190 | Zinc finger CCCH domain-containing protein 49 | -0.5199 | -0.4430 | -2.0678 | -1.2160 |
| C_3 | Gh_D07G0249 | CHIT1 | Chitotriosidase-1 | -0.2760 | -0.1883 | -0.5206 | 1.2064 |
| C_3 | Gh_D07G0310 | ZFP3 | Zinc finger protein 3 | -0.2520 | 1.2030 | -0.8730 | 0.6819 |
| C_3 | Gh_D07G0314 | pds5b-b | Sister chromatid cohesion protein PDS5 homolog B-B | 0.2803 | -0.2846 | -0.3803 | -0.0414 |
| C_3 | Gh_D07G0360 | SPX1 | SPX domain-containing protein 1 | 0.0543 | -0.5917 | -1.9439 | -0.9116 |
| C_3 | Gh_D07G0368 | At3g15810 | Protein LURP-one-related 12 | 0.1300 | 0.0752 | -1.4307 | 0.0664 |
| C_3 | Gh_D07G0425 | TDR | Leucine-rich repeat receptor-like protein kinase TDR | -0.1275 | -0.5359 | -0.7075 | 0.2414 |
| C_3 | Gh_D07G0833 | NA | NA | 0.5181 | 0.1541 | -0.6957 | 0.5982 |
| C_3 | Gh_D07G0977 | SCL14 | Scarecrow-like protein 14 | 0.3334 | 1.3589 | 0.6824 | 1.1814 |
| C_3 | Gh_D07G1069 | FAR3 | Fatty acyl-CoA reductase 3 | -1.8468 | 0.8121 | -2.1296 | 1.2243 |
| C_3 | Gh_D07G1075 | CET2 | CEN-like protein 2 | 1.0577 | 0.3398 | -4.1391 | -0.9875 |
| C_3 | Gh_D07G1305 | MYB86 | Transcription factor MYB86 | -0.2086 | -0.4814 | -0.9812 | 0.1722 |
| C_3 | Gh_D07G1469 | TKL-2 | Transketolase-2, chloroplastic | 0.1798 | 0.2558 | 0.1912 | 0.8922 |
| C_3 | Gh_D07G1516 | NPF5.2 | Protein NRT1/ PTR FAMILY 5.2 | -0.4099 | -1.1991 | -1.1675 | 1.2219 |
| C_3 | Gh_D07G1526 | IAA11 | Auxin-responsive protein IAA11 | 0.5009 | -0.3820 | -0.5223 | 0.1156 |
| C_3 | Gh_D07G1871 | GT6 | UDP-glucose flavonoid 3-O-glucosyltransferase 6 | -0.1412 | -0.6800 | -0.6593 | 0.1643 |
| C_3 | Gh_D07G2101 | At5g39570 | Uncharacterized protein | 0.1682 | 0.1482 | 0.0238 | 0.1392 |
| C_3 | Gh_D07G2115 | D27 | Beta-carotene isomerase D27, chloroplastic | 0.5286 | -0.3503 | -1.0750 | -0.6094 |
| C_3 | Gh_D07G2138 | CYP94A1 | Cytochrome P450 94A1 | 0.2056 | 0.9823 | 0.1163 | 1.4766 |
| C_3 | Gh_D07G2161 | Tom1l2 | TOM1-like protein 2 | -0.1999 | -0.2210 | -0.2719 | -0.0225 |
| C_3 | Gh_D07G2165 | NAC021 | NAC domain-containing protein 21/22 | -1.1282 | -0.0143 | -1.2974 | -0.6576 |
| C_3 | Gh_D07G2208 | ABCB15 | ABC transporter B family member 15 | -0.1442 | 0.0033 | -1.1300 | -0.4225 |
| C_3 | Gh_D07G2226 | UVH3 | DNA repair protein UVH3 | -0.0859 | 0.0780 | -0.6166 | -0.0546 |
| C_3 | Gh_D07G2235 | FDH1 | Formate dehydrogenase, mitochondrial | -0.8206 | 0.0601 | -0.3990 | 0.0662 |
| C_3 | Gh_D07G2490 | H6H | Hyoscyamine 6-dioxygenase | -0.7949 | 0.3168 | -0.1950 | 0.3026 |
| C_3 | Gh_D08G0072 | At4g19190 | Uncharacterized zinc finger CCHC domain-containing | 0.6426 | -0.6119 | -0.8678 | -0.4103 |
|  |  |  | protein |  |  |  |  |
| C_3 | Gh_D08G0205 | NA | NA | 2.6420 | 2.5112 | 1.3693 | 1.8497 |
| C_3 | Gh_D08G0238 | PMA4 | Plasma membrane ATPase 4 | -0.3304 | -0.2447 | -0.9712 | 0.0943 |
| C_3 | Gh_D08G0310 | YLS3 | Protein YLS3 | -0.2814 | 0.2022 | -0.9371 | -0.0277 |
| C_3 | Gh_D08G0429 | NA | NA | 0.2134 | -0.1167 | -0.2235 | 0.1637 |
| C_3 | Gh_D08G0436 | TPX2 | Protein TPX2 | -0.7420 | 0.7562 | -0.9107 | 0.8452 |
| C_3 | Gh_D08G0691 | STY8 | Serine/threonine-protein kinase STY8 | -0.7142 | 0.0780 | -1.5106 | -0.3196 |
| C_3 | Gh_D08G0692 | HMT-2 | Homocysteine S-methyltransferase 2 | -0.1417 | 1.1057 | -0.3267 | 0.5559 |
| C_3 | Gh_D08G1067 | DCR | BAHD acyltransferase DCR | 0.3473 | -0.3565 | -1.5820 | -0.3171 |
| C_3 | Gh_D08G1135 | OMT | Caffeic acid 3-O-methyltransferase | -0.1422 | 0.2488 | -0.5814 | 0.3121 |
| C_3 | Gh_D08G1275 | NA | NA | -0.1698 | -0.0040 | -0.7547 | 0.0351 |
| C_3 | Gh_D08G1309 | SUS2 | Sucrose synthase 2 | -0.6987 | 0.7143 | -0.7165 | 0.8100 |
| C_3 | Gh_D08G1398 | NA | NA | -0.2462 | -1.0129 | -1.3447 | -0.5558 |
| C_3 | Gh_D08G1541 | NSP5 | Nitrile-specifier protein 5 | -1.1453 | -0.7514 | -1.3539 | -0.1200 |
| C_3 | Gh_D08G1628 | CDKC-2 | Cyclin-dependent kinase C-2 | 0.6833 | -0.0268 | -0.4505 | -0.2457 |
| C_3 | Gh_D08G1647 | COG6 | Conserved oligomeric Golgi complex subunit 6 | 0.2535 | 0.0871 | -0.1406 | -0.0826 |
| C_3 | Gh_D08G1709 | At1g61280 | Phosphatidylinositol N-acetylglucosaminyltransferase | 0.1377 | 0.0552 | 0.0536 | 0.3577 |
|  |  |  | subunit P |  |  |  |  |
| C_3 | Gh_D08G1728 | NPF7.3 | Protein NRT1/ PTR FAMILY 7.3 | 0.0487 | 0.2243 | -0.7171 | -0.0578 |
| C_3 | Gh_D08G2119 | At1g06890 | Uncharacterized membrane protein | -0.8190 | -0.0354 | -0.7911 | 0.8480 |
| C_3 | Gh_D08G2187 | NA | Pyrophosphate-energized vacuolar membrane proton | -1.4110 | 1.1453 | -1.0744 | 1.2792 |
|  |  |  | pump |  |  |  |  |
| C_3 | Gh_D08G2205 | PME58 | Probable pectinesterase/pectinesterase inhibitor 58 | -0.1315 | -0.0747 | -0.2058 | 0.6068 |
| C_3 | Gh_D08G2214 | DEGP14 | Putative protease Do-like 14 | -0.9635 | 0.3673 | -1.3840 | -0.4298 |
| C_3 | Gh_D08G2306 | NA | NA | -0.2118 | 0.5919 | -1.5793 | -0.3015 |
| C_3 | Gh_D08G2503 | IAA16 | Auxin-responsive protein IAA16 | 0.1654 | 0.6397 | -0.3535 | 1.3072 |
| C_3 | Gh_D08G2744 | ctps | CTP synthase | 0.0196 | 0.5925 | -0.7390 | 0.0950 |
| C_3 | Gh_D09G0037 | CHR24 | Protein CHROMATIN REMODELING 24 | -0.0607 | 0.1938 | -0.1737 | 0.0671 |
| C_3 | Gh_D09G0107 | CNGC2 | Cyclic nucleotide-gated ion channel 2 | -1.0313 | 0.3568 | -1.5679 | 0.4217 |
| C_3 | Gh_D09G0174 | At4g08850 | Probable LRR receptor-like serine/threonine-protein | -0.1738 | 0.2128 | -0.1424 | 1.5325 |
|  |  |  | kinase |  |  |  |  |
| C_3 | Gh_D09G0243 | PP2A9 | Protein PHLOEM PROTEIN 2-LIKE A9 | -1.7037 | -0.8031 | -1.2071 | -0.3430 |
| C_3 | Gh_D09G0355 | UKL1 | Uridine kinase-like protein 1, chloroplastic | -0.4318 | 0.1221 | -1.0532 | 0.4247 |
| C_3 | Gh_D09G0432 | yjcL | Uncharacterized membrane protein YjcL | -0.8618 | 2.2215 | -6.8743 | 1.0213 |
| C_3 | Gh_D09G0436 | At4g25390 | Receptor-like serine/threonine-protein kinase | -0.2850 | -0.8794 | -2.1747 | -0.6817 |
| C_3 | Gh_D09G0459 | MSH2 | DNA mismatch repair protein MSH2 | 0.2311 | -0.2048 | -0.4812 | -0.0867 |
| C_3 | Gh_D09G0700 | TSJT1 | Stem-specific protein TSJT1 | -0.9446 | 0.6734 | -0.6564 | 0.1234 |
| C_3 | Gh_D09G0732 | At1g56130 | Probable LRR receptor-like serine/threonine-protein kinase | 0.0088 | 0.5950 | -0.5517 | 0.0503 |
| C_3 | Gh_D09G0966 | LHW | Transcription factor LHW | 0.6901 | 0.5156 | -0.2635 | 0.1038 |
| C_3 | Gh_D09G1051 | CLT3 | Protein CLT3, chloroplastic | -0.8205 | -0.3485 | -3.1636 | -0.2154 |
| C_3 | Gh_D09G1208 | PER53 | Peroxidase 53 | -0.5144 | 0.9596 | -2.3562 | 0.8328 |
| C_3 | Gh_D09G1213 | kif11 | Kinesin-related protein 11 | -0.2300 | -0.1773 | -0.5169 | -0.0600 |
| C_3 | Gh_D09G1349 | IDS3 | 2'-deoxymugineic-acid 2'-dioxygenase | -0.1890 | 0.0776 | -0.9752 | 0.2911 |
| C_3 | Gh_D09G1627 | DXR | 1-deoxy-D-xylulose 5-phosphate reductoisomerase, | -0.7004 | -0.0099 | -0.9110 | -0.0102 |
|  |  |  | chloroplastic |  |  |  |  |
| C_3 | Gh_D09G1765 | atad1a | ATPase family AAA domain-containing protein 1-A | 0.1206 | 0.3633 | -0.3935 | -0.0068 |
| C_3 | Gh_D09G1795 | NLP7 | Protein NLP7 | -0.0125 | -0.0215 | -0.7003 | -0.3031 |
| C_3 | Gh_D09G2000 | UGT89C1 | UDP-glycosyltransferase 89C1 | 0.0708 | -1.5053 | -1.9951 | -0.9728 |
| C_3 | Gh_D09G2080 | LOX5 | Linoleate 9S-lipoxygenase 5, chloroplastic | -0.3871 | 0.1432 | -1.1064 | -0.0747 |
| C_3 | Gh_D09G2089 | FRL4A | FRIGIDA-like protein 4a | 0.3604 | 0.2074 | -0.0403 | 0.3206 |
| C_3 | Gh_D09G2214 | PAP6 | Probable plastid-lipid-associated protein 6, chloroplastic | -0.3053 | -0.5989 | -2.1393 | -0.8464 |
| C_3 | Gh_D09G2436 | NA | NA | -2.5303 | 0.0390 | -6.8303 | -2.0152 |
| C_3 | Gh_D09G2482 | NA | NA | -4.1427 | -2.8627 | -3.8925 | -0.5155 |
| C_3 | Gh_D10G0237 | LECRK71 | L-type lectin-domain containing receptor kinase VII.1 | 1.0348 | -0.4639 | -0.4065 | 2.4142 |
| C_3 | Gh_D10G0336 | RPL11B | 60S ribosomal protein L11-2 | 0.3265 | -0.3013 | -1.0385 | -0.8644 |
| C_3 | Gh_D10G0340 | NAT2 | Nucleobase-ascorbate transporter 2 | 0.1363 | 0.3674 | -0.2137 | 0.0738 |
| C_3 | Gh_D10G0384 | NA | NA | 0.1971 | 0.9252 | 0.3629 | 0.8798 |
| C_3 | Gh_D10G0405 | NA | NA | -0.1443 | 1.1909 | -2.2563 | -0.4813 |
| C_3 | Gh_D10G0650 | ALAAT2 | Alanine aminotransferase 2, mitochondrial | 1.9950 | 1.4533 | -7.1231 | -2.2571 |
| C_3 | Gh_D10G0916 | DOF3.4 | Dof zinc finger protein DOF3.4 | -0.0637 | 1.0140 | -1.8939 | 3.6557 |
| C_3 | Gh_D10G1050 | UVR8 | Ultraviolet-B receptor UVR8 | -0.1848 | -0.1862 | -1.2575 | 0.4039 |
| C_3 | Gh_D10G1122 | SPAC5D6.04 | Uncharacterized transporter C5D6.04 | -0.3319 | -0.1003 | -0.5308 | 0.3312 |
| C_3 | Gh_D10G1245 | SHR | Protein SHORT-ROOT | 1.2981 | 1.3945 | -1.8554 | -0.3981 |
| C_3 | Gh_D10G1296 | SLC35F1 | Solute carrier family 35 member F1 | 0.2817 | -0.5527 | -0.8976 | -0.0506 |
| C_3 | Gh_D10G1368 | SAHH | Adenosylhomocysteinase | 0.3940 | -0.2400 | -0.8682 | -0.3342 |
| C_3 | Gh_D10G1482 | AGO10 | Protein argonaute 10 | 0.0554 | -0.1478 | -0.1074 | 0.2967 |
| C_3 | Gh_D10G1604 | AMT1-1 | Ammonium transporter 1 member 1 | -1.1943 | 1.9601 | -5.2089 | -0.2328 |
| C_3 | Gh_D10G1705 | NA | Endo-1,3 | -1.4546 | -0.3074 | -1.0518 | 0.0629 |
| C_3 | Gh_D10G1793 | NA | NA | 1.1175 | 1.1955 | 0.1176 | 0.7656 |
| C_3 | Gh_D10G1871 | AZG1 | Adenine/guanine permease AZG1 | -1.2612 | -0.5516 | -1.5699 | -0.3311 |
| C_3 | Gh_D10G1873 | At1g18980 | Germin-like protein subfamily T member 2 | -0.9580 | -0.2664 | -0.8348 | 0.5043 |
| C_3 | Gh_D10G1964 | RPPL1 | Putative disease resistance RPP13-like protein 1 | -0.8617 | 0.4916 | -1.2533 | 0.1232 |
| C_3 | Gh_D10G2035 | SDR1 | (+)-neomenthol dehydrogenase | -1.1643 | 1.4008 | -2.4236 | 0.3159 |
| C_3 | Gh_D10G2055 | CYP71A1 | Cytochrome P450 71A1 | -1.1799 | 0.3936 | -1.1984 | -0.1055 |
| C_3 | Gh_D10G2056 | CYP71A1 | Cytochrome P450 71A1 | -1.7991 | 0.9029 | -2.1760 | 0.3575 |
| C_3 | Gh_D10G2059 | Os11g0148500 | Pyruvate kinase 1, cytosolic | -0.2313 | 0.3150 | -0.1909 | 0.1192 |
| C_3 | Gh_D10G2361 | TRG-31 | Probable aquaporin PIP-type 7a | -0.4003 | 0.1948 | -0.7011 | 0.2134 |
| C_3 | Gh_D10G2372 | At3g47570 | Probable LRR receptor-like serine/threonine-protein | -0.4613 | -1.2586 | -2.2461 | 0.3286 |
|  |  |  | kinase |  |  |  |  |
| C_3 | Gh_D10G2413 | CAN2 | Staphylococcal-like nuclease CAN2 | 0.1092 | -0.8001 | -0.9897 | -0.4168 |
| C_3 | Gh_D10G2422 | At4g27190 | Disease resistance protein | -0.1593 | 0.8584 | -0.5403 | 0.3529 |
| C_3 | Gh_D10G2427 | EMB2279 | Pentatricopeptide repeat-containing protein | -0.1133 | -0.8032 | -0.9097 | -0.2431 |
| C_3 | Gh_D10G2429 | CKX6 | Cytokinin dehydrogenase 6 | 0.6836 | 1.1548 | -1.6430 | 0.2311 |
| C_3 | Gh_D10G2520 | NA | NA | 0.0429 | -1.2305 | -3.2097 | -1.3311 |
| C_3 | Gh_D10G2549 | ROMT | Trans-resveratrol di-O-methyltransferase | 0.4647 | 1.2687 | -1.1893 | 0.8165 |
| C_3 | Gh_D10G2619 | ENDO1 | Endonuclease 1 | 0.1181 | 0.5777 | -0.2989 | 0.2606 |
| C_3 | Gh_D11G0214 | GA3OX1 | Gibberellin 3-beta-dioxygenase 1 | -1.4345 | 0.4249 | -2.2508 | -0.5855 |
| C_3 | Gh_D11G0233 | NA | NA | -0.6415 | 0.4633 | -1.8323 | 0.3638 |
| C_3 | Gh_D11G0235 | SBT2.5 | Subtilisin-like protease SBT2.5 | -0.1355 | 0.4524 | -1.4524 | -0.2503 |
| C_3 | Gh_D11G0266 | HAT2 | Homeobox-leucine zipper protein HAT2 | 0.5273 | 0.4891 | -0.0711 | 0.3927 |
| C_3 | Gh_D11G0440 | NA | NA | 0.4707 | -0.1547 | -0.2659 | 0.1410 |
| C_3 | Gh_D11G0441 | ALDH10A8 | Betaine aldehyde dehydrogenase 1, chloroplastic | -0.0682 | 0.6921 | -0.3843 | 0.8905 |
| C_3 | Gh_D11G0449 | PIN1 | Peptidyl-prolyl cis-trans isomerase Pin1 | 0.2638 | -0.0761 | -0.6211 | 0.3415 |
| C_3 | Gh_D11G0458 | STR18 | Thiosulfate sulfurtransferase 18 | -0.3871 | 0.9980 | -0.1344 | 0.8432 |
| C_3 | Gh_D11G0525 | ACS2 | 1-aminocyclopropane-1-carboxylate synthase CMA101 | -0.3739 | 0.0365 | -0.1074 | 2.3350 |
| C_3 | Gh_D11G0532 | At5g65660 | Uncharacterized protein | -0.1349 | 0.7850 | -1.2209 | 0.6212 |
| C_3 | Gh_D11G0606 | ADH1 | Alcohol dehydrogenase 1 | -0.9128 | -1.1829 | -1.4982 | 0.8256 |
| C_3 | Gh_D11G0868 | NA | NA | -0.1769 | -0.4487 | -0.5132 | -0.3958 |
| C_3 | Gh_D11G0918 | NA | NAD-dependent malic enzyme 59 kDa isoform, | -0.1449 | -0.0256 | -1.3527 | 0.2961 |
|  |  |  | mitochondrial |  |  |  |  |
| C_3 | Gh_D11G0926 | KDELC1 | KDEL motif-containing protein 1 | -0.4112 | -0.5857 | -0.8149 | -0.2265 |
| C_3 | Gh_D11G0963 | GSVIVT00026920001 | Probable polygalacturonase | 0.2232 | 0.4428 | -0.1238 | 0.9653 |
| C_3 | Gh_D11G1281 | NA | Ent-copalyl diphosphate synthase, chloroplastic | 1.0204 | 0.1716 | 0.1783 | 0.9457 |
| C_3 | Gh_D11G1302 | EOL1 | ETO1-like protein 1 | 0.0770 | 0.6547 | 0.0498 | 0.4502 |
| C_3 | Gh_D11G1430 | PAP15 | Purple acid phosphatase 15 | -0.1486 | 0.8744 | -0.4703 | 0.2441 |
| C_3 | Gh_D11G1583 | CSLH1 | Cellulose synthase-like protein H1 | -0.0682 | 0.0282 | -0.1082 | 1.6422 |
| C_3 | Gh_D11G1805 | CYP84A1 | Cytochrome P450 84A1 | -1.2179 | -0.1201 | -2.2279 | 0.6788 |
| C_3 | Gh_D11G1998 | TTC1 | Tetratricopeptide repeat protein 1 | -0.2993 | 1.2595 | -1.1545 | 0.2224 |
| C_3 | Gh_D11G2008 | At5g67385 | BTB/POZ domain-containing protein | 0.1547 | 1.7345 | -2.9733 | 1.0002 |
| C_3 | Gh_D11G2011 | SCL32 | Scarecrow-like protein 32 | -0.6195 | 0.4205 | -0.7481 | 0.1750 |
| C_3 | Gh_D11G2019 | FH6 | Formin-like protein 6 | -0.1643 | 0.7014 | 0.2156 | 0.6171 |
| C_3 | Gh_D11G2357 | SOT15 | Cytosolic sulfotransferase 15 | 0.2652 | 0.5951 | -1.7924 | 0.2579 |
| C_3 | Gh_D11G2518 | AMT1-2 | Ammonium transporter 1 member 2 | 0.0275 | -0.7352 | -1.7078 | 0.1673 |
| C_3 | Gh_D11G2521 | NA | NA | 0.2084 | 0.9496 | -0.7845 | 0.0037 |
| C_3 | Gh_D11G2549 | PPCK1 | Phosphoenolpyruvate carboxylase kinase 1 | 0.3917 | 0.4132 | -1.2343 | 0.6075 |
| C_3 | Gh_D11G2812 | CHLN | Nicotianamine synthase | 0.0310 | -1.0735 | -2.2177 | -0.1546 |
| C_3 | Gh_D11G2817 | ABCC3 | ABC transporter C family member 3 | -0.5146 | 0.1823 | -0.3611 | 0.1068 |
| C_3 | Gh_D11G2909 | NA | NA | 0.3637 | 0.4383 | -0.4494 | -0.0884 |
| C_3 | Gh_D11G2943 | GH3.17 | Indole-3-acetic acid-amido synthetase GH3.17 | -1.1932 | -0.4280 | -0.9221 | -0.5955 |
| C_3 | Gh_D11G3083 | Nanp | N-acylneuraminate-9-phosphatase | -0.9548 | -0.3669 | -1.1776 | -0.0436 |
| C_3 | Gh_D11G3106 | NA | NA | -0.5641 | 0.7123 | -6.1469 | 1.4674 |
| C_3 | Gh_D11G3141 | NA | Remorin | 0.0081 | -1.4792 | -1.8047 | -0.9218 |
| C_3 | Gh_D11G3213 | UEV1C | Ubiquitin-conjugating enzyme E2 variant 1C | -0.0865 | -0.3674 | -0.7441 | -0.4483 |
| C_3 | Gh_D11G3214 | UEV1C | Ubiquitin-conjugating enzyme E2 variant 1C | 0.0157 | -0.4326 | -0.5539 | -0.1315 |
| C_3 | Gh_D11G3240 | NA | NA | 0.0133 | 0.0416 | -0.8800 | -0.4965 |
| C_3 | Gh_D11G3245 | NA | NA | 0.0645 | -0.1795 | -0.3041 | 0.1647 |
| C_3 | Gh_D11G3260 | NA | Transcription factor MYB1R1 | -0.3699 | -0.3627 | -1.3479 | -0.5229 |
| C_3 | Gh_D11G3321 | At3g09070 | UPF0503 protein At3g09070, chloroplastic | 1.0969 | 0.8746 | -0.2167 | 0.7345 |
| C_3 | Gh_D11G3399 | CAD | Probable mannitol dehydrogenase | -1.4722 | -0.1638 | -0.9382 | 0.7770 |
| C_3 | Gh_D11G3412 | NAD-ME2 | NAD-dependent malic enzyme 2, mitochondrial | -0.4846 | -1.6760 | -1.8873 | 2.1739 |
| C_3 | Gh_D12G0066 | GID1B | Gibberellin receptor GID1B | -0.3886 | 0.4283 | -0.3765 | 0.2005 |
| C_3 | Gh_D12G0295 | PIP2-6 | Probable aquaporin PIP2-6 | -0.4196 | -1.1516 | -1.5734 | 0.9483 |
| C_3 | Gh_D12G0331 | CPP1 | Protein CHAPERONE-LIKE PROTEIN OF POR1, | -0.5699 | -1.3208 | -1.2019 | -0.2780 |
|  |  |  | chloroplastic |  |  |  |  |
| C_3 | Gh_D12G0389 | CBSDUF3 | DUF21 domain-containing protein At2g14520 | -0.6271 | -0.2034 | -0.8390 | 0.0276 |
| C_3 | Gh_D12G0466 | GSO1 | LRR receptor-like serine/threonine-protein kinase GSO1 | 0.0270 | 0.2478 | -0.0473 | 0.3797 |
| C_3 | Gh_D12G0683 | At4g37250 | Probable LRR receptor-like serine/threonine-protein | -0.2618 | -0.1151 | -0.5357 | 0.3399 |
|  |  |  | kinase |  |  |  |  |
| C_3 | Gh_D12G1169 | NAC043 | NAC domain-containing protein 43 | -1.1972 | 1.4627 | -4.4148 | 1.6519 |
| C_3 | Gh_D12G1410 | ACX3 | Acyl-coenzyme A oxidase 3, peroxisomal | -0.1224 | 0.9837 | -0.6224 | 0.1469 |
| C_3 | Gh_D12G1412 | NUDT13 | Nudix hydrolase 13, mitochondrial | -0.3391 | 0.0296 | -0.7796 | -0.1003 |
| C_3 | Gh_D12G1442 | SRO2 | Probable inactive poly [ADP-ribose] polymerase SRO2 | -0.5597 | 0.3315 | -0.2450 | 0.4268 |
| C_3 | Gh_D12G1637 | RNS1 | Ribonuclease 1 | 0.0209 | 0.5112 | -0.7312 | 0.9593 |
| C_3 | Gh_D12G1688 | BHLH30 | Transcription factor bHLH30 | 0.6976 | 0.5456 | 0.0625 | 0.4098 |
| C_3 | Gh_D12G1702 | PPC4 | Phosphoenolpyruvate carboxylase 4 | -0.0227 | -0.1379 | -0.3573 | 0.1414 |
| C_3 | Gh_D12G2021 | BOB1 | Protein BOBBER 1 | -0.3098 | -0.0568 | -1.4890 | 1.9061 |
| C_3 | Gh_D12G2178 | NA | 14 kDa proline-rich protein DC2.15 | -0.6547 | 1.3778 | -1.8749 | 1.0127 |
| C_3 | Gh_D12G2529 | NA | NA | -0.1178 | 0.2396 | -0.7111 | 0.0184 |
| C_3 | Gh_D12G2574 | MKK7 | Mitogen-activated protein kinase kinase 7 | -0.3871 | 0.2771 | -0.1889 | 0.3960 |
| C_3 | Gh_D12G2728 | PCMP-H3 | Pentatricopeptide repeat-containing protein | -1.2307 | 0.4957 | -0.7520 | 0.8708 |
| C_3 | Gh_D12G2752 | NA | NA | 0.1628 | 1.1181 | -0.3428 | 0.5256 |
| C_3 | Gh_D12G2837 | ISPH | 4-hydroxy-3-methylbut-2-enyl diphosphate reductase, | 0.1001 | 1.4533 | -0.3814 | 0.7944 |
|  |  |  | chloroplastic |  |  |  |  |
| C_3 | Gh_D13G0031 | LBD1 | LOB domain-containing protein 1 | -0.4073 | -0.5732 | -1.2705 | 1.0933 |
| C_3 | Gh_D13G0033 | CPK1 | Calcium-dependent protein kinase 1 | 0.1044 | 0.5589 | -0.5412 | 0.2219 |
| C_3 | Gh_D13G0059 | NA | NA | -0.4912 | 0.1504 | -2.4711 | -0.6059 |
| C_3 | Gh_D13G0102 | TOC159 | Translocase of chloroplast 159, chloroplastic | 0.2244 | -0.0562 | -1.2302 | -0.2987 |
| C_3 | Gh_D13G0109 | PRFB3 | Peptide chain release factor PrfB3, chloroplastic | 0.3184 | 0.5104 | 0.2782 | 0.4055 |
| C_3 | Gh_D13G0137 | ERD15 | Protein EARLY RESPONSIVE TO DEHYDRATION 15 | -0.4482 | 0.6393 | -0.8209 | 0.3421 |
| C_3 | Gh_D13G0219 | AN11010 | Putative GTPase-activating protein AN11010 | -0.3199 | 0.5647 | -0.1820 | 0.8826 |
| C_3 | Gh_D13G0256 | ESYT1 | Extended synaptotagmin-1 | 0.3430 | -0.0030 | -4.6558 | -1.3343 |
| C_3 | Gh_D13G0445 | At2g23090 | Uncharacterized protein | -0.5384 | -0.4113 | -1.0132 | -0.7163 |
| C_3 | Gh_D13G0511 | NA | NA | -3.2091 | 0.0435 | -4.1447 | 0.7158 |
| C_3 | Gh_D13G0646 | NA | NA | 0.9250 | -0.9127 | -2.2867 | -0.7551 |
| C_3 | Gh_D13G0717 | TEM1 | AP2/ERF and B3 domain-containing transcription | 0.3244 | 1.5514 | 0.4385 | 1.1583 |
|  |  |  | repressor TEM1 |  |  |  |  |
| C_3 | Gh_D13G0729 | CPLS1 | ATP-dependent Clp protease adapter protein CLPS1, chloroplastic | 0.6180 | 0.1832 | -0.5102 | 0.4835 |
| C_3 | Gh_D13G0838 | ZAT4 | Zinc finger protein ZAT4 | -1.3401 | 0.9803 | -1.8654 | 0.2287 |
| C_3 | Gh_D13G0865 | CYP78A5 | Cytochrome P450 78A5 | 1.8790 | 2.0012 | -1.0906 | 1.2743 |
| C_3 | Gh_D13G1154 | PPC6-7 | Probable protein phosphatase 2C 73 | -1.2200 | 0.6294 | -0.5999 | 1.0140 |
| C_3 | Gh_D13G1246 | PATL5 | Patellin-5 | -0.0633 | 0.8066 | -0.2574 | 0.4530 |
| C_3 | Gh_D13G1275 | SAUR72 | Auxin-responsive protein SAUR72 | -0.7918 | -0.3342 | -0.5761 | 0.4821 |
| C_3 | Gh_D13G1492 | Os02g0190300 | Putative multidrug resistance protein | -0.1105 | -0.4886 | -2.3580 | -0.1152 |
| C_3 | Gh_D13G1617 | NA | Probable glutathione S-transferase | 0.1364 | -0.6850 | -0.9582 | -0.0631 |
| C_3 | Gh_D13G1632 | CODM | Codeine O-demethylase | -0.8849 | 0.5914 | -1.2767 | 0.4963 |
| C_3 | Gh_D13G1826 | NA | Zeta-carotene desaturase, chloroplastic/chromoplastic | -0.1231 | -0.1929 | -0.4979 | 0.1283 |
| C_3 | Gh_D13G1830 | CYSD1 | Bifunctional L-3-cyanoalanine synthase/cysteine synthase | 0.1866 | 0.8527 | -0.1327 | 0.4453 |
|  |  |  | D1 |  |  |  |  |
| C_3 | Gh_D13G1846 | GID1C | Gibberellin receptor GID1C | -0.1791 | 0.4553 | -0.5452 | 0.0244 |
| C_3 | Gh_D13G2022 | SLC25A44 | Solute carrier family 25 member 44 | -0.2363 | 1.1688 | 0.0556 | 1.9367 |
| C_3 | Gh_D13G2051 | NFYA3 | Nuclear transcription factor Y subunit A-3 | -0.5071 | 0.1644 | -2.0511 | -0.5633 |
| C_3 | Gh_D13G2063 | NA | NA | -0.5018 | -0.7608 | -1.2098 | -0.0745 |
| C_3 | Gh_D13G2128 | NA | NA | 1.0100 | 0.4915 | -0.4841 | 0.5735 |
| C_3 | Gh_D13G2156 | AOP1 | Probable 2-oxoglutarate-dependent dioxygenase AOP1 | -0.8730 | 1.0676 | -0.6099 | 0.6922 |
| C_3 | Gh_D13G2365 | SMO2-2 | Methylsterol monooxygenase 2-2 | -0.6369 | -0.2407 | -1.6167 | -0.5826 |
| C_3 | Gh_D13G2409 | REV | Homeobox-leucine zipper protein REVOLUTA | -0.0407 | -0.1551 | -0.4872 | 0.5175 |
| C_3 | Gh_D13G2434 | IRX7 | Probable glucuronoxylan glucuronosyltransferase IRX7 | 0.0192 | 0.2491 | -0.1225 | 0.3604 |
| C_3 | Gh_Sca004768G02 | TFIIA-S | Transcription initiation factor IIA subunit 2 | -0.1125 | -0.1732 | -1.2482 | -0.7663 |
| C_3 | Gh_Sca004802G01 | Os03g0733400 | Zinc finger BED domain-containing protein | 0.1898 | 1.4307 | -0.9223 | 0.6331 |
|  |  |  | RICESLEEPER 2 |  |  |  |  |
| C_3 | Gh_Sca004838G01 | NA | NA | -0.0618 | 0.6729 | -1.4252 | -0.4332 |
| C_3 | Gh_Sca004867G05 | NA | Alpha-glucosidase | 0.0806 | 0.0610 | -1.5448 | -0.2644 |
| C_3 | Gh_Sca005162G01 | WAK5 | Wall-associated receptor kinase 5 | 0.0066 | -1.5873 | -1.6536 | 1.2926 |
| C_3 | Gh_Sca005495G02 | 4'OMT2 | 3'-hydroxy-N-methyl-(S)-coclaurine 4'-O- | 0.3539 | 1.0489 | -0.7911 | 0.4206 |
|  |  |  | methyltransferase 2 |  |  |  |  |
| C_3 | Gh_Sca005564G01 | WRKY23 | Probable WRKY transcription factor 23 | -0.3101 | 1.2316 | 0.2147 | 1.2185 |
| C_3 | Gh_Sca005608G02 | DRP4C | Dynamin-related protein 4C | 1.1493 | 0.0235 | -0.9322 | -0.1234 |
| C_3 | Gh_Sca008028G01 | H6H | Hyoscyamine 6-dioxygenase | -0.0134 | 0.4306 | -0.4101 | 0.1368 |
| C_3 | Gh_Sca008634G01 | NA | NA | 0.0016 | -0.4429 | -0.4482 | 0.3684 |
| C_3 | Gh_Sca019661G01 | CBP | Citrate-binding protein | 2.5446 | 2.3867 | 0.0590 | 1.2010 |
| C_4 | Gh_A01G0015 | STY46 | Serine/threonine-protein kinase STY46 | -0.7987 | 1.0379 | -0.4808 | -0.5747 |
| C_4 | Gh_A01G0116 | NA | NA | 0.3284 | 0.6277 | 0.6925 | 0.1510 |
| C_4 | Gh_A01G0177 | MRS2-2 | Magnesium transporter MRS2-2 | 0.1000 | 2.3965 | -0.1160 | -0.5376 |
| C_4 | Gh_A01G0265 | BRG3 | Probable BOI-related E3 ubiquitin-protein ligase 3 | -0.0876 | 0.3798 | -0.1191 | -0.7071 |
| C_4 | Gh_A01G0307 | SRG1 | Protein SRG1 | 0.7915 | -0.2669 | -0.5487 | -1.6266 |
| C_4 | Gh_A01G0361 | SPAPJ696.02 | SH3 domain-containing protein PJ696.02 | 1.3643 | 1.1706 | 1.7306 | -0.3882 |
| C_4 | Gh_A01G0417 | VCX1 | Vacuolar calcium ion transporter | -0.4303 | 0.6587 | -0.2874 | -1.1574 |
| C_4 | Gh_A01G0801 | ZIP1 | Zinc transporter 1 | -0.0170 | 0.0241 | -0.4517 | -1.0171 |
| C_4 | Gh_A01G0824 | MLO-H1 | MLO protein homolog 1 | 0.6589 | 0.9147 | 0.0217 | -0.2262 |
| C_4 | Gh_A01G0875 | PREP | Prolyl endopeptidase | -1.8012 | 1.7110 | -2.7633 | -1.6923 |
| C_4 | Gh_A01G0880 | PLDDELTA | Phospholipase D delta | 0.3125 | 0.7862 | 0.0980 | 0.1377 |
| C_4 | Gh_A01G0997 | RAP2-1 | Ethylene-responsive transcription factor RAP2-1 | -0.9236 | 2.6823 | -1.4782 | -1.6918 |
| C_4 | Gh_A01G1111 | NA | NA | 0.1069 | 1.3773 | 0.1780 | 0.4861 |
| C_4 | Gh_A01G1113 | NA | NA | 0.4069 | 1.4614 | 0.7193 | 0.7272 |
| C_4 | Gh_A01G1156 | CCMFC | Cytochrome c biogenesis CcmF C-terminal-like | 0.2573 | 1.0215 | 1.0087 | -0.1132 |
|  |  |  | mitochondrial protein |  |  |  |  |
| C_4 | Gh_A01G1351 | PIP1-5 | Aquaporin PIP1-5 | 3.4655 | 2.9817 | 1.5838 | 1.6043 |
| C_4 | Gh_A01G1381 | DDB_G02887 | CTL-like protein DDB_G0288717 | 0.1424 | 0.2549 | 0.2575 | -0.1883 |
|  |  | 17 |  |  |  |  |  |
| C_4 | Gh_A01G1387 | MYB308 | Myb-related protein 308 | -0.1916 | 0.5451 | -1.6826 | -0.9104 |
| C_4 | Gh_A01G1408 | NA | Blue copper protein | 0.1215 | -0.0156 | -0.0940 | -0.1181 |
| C_4 | Gh_A01G1425 | STY46 | Serine/threonine-protein kinase STY46 | -1.0287 | 0.3544 | -0.4964 | -0.4869 |
| C_4 | Gh_A01G1454 | At4g35230 | Probable serine/threonine-protein kinase | 0.3794 | 1.0218 | 0.4698 | 0.1266 |
| C_4 | Gh_A01G1490 | ARPN | Basic blue protein | 0.5786 | 0.9253 | 0.2456 | -0.4585 |
| C_4 | Gh_A01G1501 | PAT23 | Probable protein S-acyltransferase 23 | -0.1307 | 0.8279 | -0.0774 | -0.0410 |
| C_4 | Gh_A01G1516 | POT5 | Potassium transporter 5 | -0.3909 | -1.4618 | -2.0837 | -2.2129 |
| C_4 | Gh_A01G1580 | PPCS1 | Phosphopantothenate--cysteine ligase 1 | -0.0158 | 0.2188 | -0.0326 | -0.3512 |
| C_4 | Gh_A01G1770 | Yipf7 | Protein YIPF7 | 0.2484 | 0.8305 | 0.6524 | 0.3925 |
| C_4 | Gh_A01G1834 | NA | Flavonol sulfotransferase-like | 1.0630 | 1.6634 | -0.0615 | 0.0146 |
| C_4 | Gh_A01G1900 | LHW | Transcription factor LHW | 0.3669 | 0.6779 | -1.0110 | -0.6154 |
| C_4 | Gh_A01G1909 | SPAC24B11.0 | Uncharacterized protein C24B11.05 | -0.5213 | 0.2836 | -0.8346 | -1.3223 |
|  |  | 5 |  |  |  |  |  |
| C_4 | Gh_A01G2033 | NA | NA | 0.4336 | 0.8977 | -0.3224 | -0.3351 |
| C_4 | Gh_A01G2055 | NA | NA | -0.1118 | 2.5901 | -0.6869 | -0.4342 |
| C_4 | Gh_A01G2056 | NA | NA | 0.2166 | 1.2388 | 0.0597 | -0.2344 |
| C_4 | Gh_A01G2064 | NAC072 | NAC domain-containing protein 72 | 0.2624 | 1.4095 | 0.8434 | -0.0090 |
| C_4 | Gh_A01G2109 | NA | NA | 0.5006 | 2.3225 | 0.2046 | -0.8382 |
| C_4 | Gh_A01G2159 | NA | Cysteine proteinase inhibitor 1 | 0.2257 | 0.3899 | -0.0515 | -0.0267 |
| C_4 | Gh_A02G0016 | At3g08650 | Putative zinc transporter | -0.0222 | 0.6206 | 0.3201 | 0.2835 |
| C_4 | Gh_A02G0025 | NA | NA | -1.0768 | -0.0777 | -1.7315 | -1.3724 |
| C_4 | Gh_A02G0049 | PSAP | Prosaposin | -0.6655 | 0.1958 | -0.5408 | -0.2983 |
| C_4 | Gh_A02G0076 | ccdc94 | Coiled-coil domain-containing protein 94 homolog | 0.4209 | 0.1997 | -0.1691 | -0.3375 |
| C_4 | Gh_A02G0103 | Os03g0733400 | Zinc finger BED domain-containing protein | -0.1724 | 1.6695 | 0.9570 | 0.8345 |
|  |  |  | RICESLEEPER 2 |  |  |  |  |
| C_4 | Gh_A02G0219 | NA | Cytochrome P450 CYP749A22 | -0.8074 | 0.3491 | -1.4310 | -0.6258 |
| C_4 | Gh_A02G0259 | GSTU7 | Glutathione S-transferase U7 | 0.5271 | -0.4981 | -0.0158 | -2.0071 |
| C_4 | Gh_A02G0262 | NA | Glutathione transferase GST 23 | -2.5333 | 0.0615 | -1.4467 | -0.7867 |
| C_4 | Gh_A02G0470 | SNF4 | Sucrose nonfermenting 4-like protein | -0.2931 | 0.4531 | -0.7458 | -0.2614 |
| C_4 | Gh_A02G0501 | NA | NA | -0.3945 | 4.7691 | -0.8100 | -1.5867 |
| C_4 | Gh_A02G0662 | NA | NA | -0.3596 | 0.6647 | -0.3249 | -0.3881 |
| C_4 | Gh_A02G0816 | IKU2 | Receptor-like protein kinase HAIKU2 | 0.1621 | 0.4220 | 0.4002 | -0.0793 |
| C_4 | Gh_A02G0855 | NA | NA | 0.2323 | 0.9679 | 0.0034 | 0.1114 |
| C_4 | Gh_A02G0946 | MTP1 | Metal tolerance protein 1 | -0.2078 | 0.8551 | 0.2478 | 0.2009 |
| C_4 | Gh_A02G0959 | ALMT10 | Aluminum-activated malate transporter 10 | 0.1198 | 1.0591 | 1.0737 | 0.0622 |
| C_4 | Gh_A02G1042 | WRKY29 | Probable WRKY transcription factor 29 | 0.9268 | 0.0197 | -0.0517 | -0.9492 |
| C_4 | Gh_A02G1046 | ACS1 | 1-aminocyclopropane-1-carboxylate synthase | 0.0410 | 1.3517 | -0.1197 | -0.4988 |
| C_4 | Gh_A02G1089 | NA | NA | 0.0991 | 0.1881 | -0.7584 | -2.4339 |
| C_4 | Gh_A02G1317 | CESA6 | Cellulose synthase A catalytic subunit 6 [UDP-forming] | 0.2486 | 1.2639 | 0.7936 | 0.9435 |
| C_4 | Gh_A02G1340 | NA | NA | 0.0790 | 3.2606 | 1.0417 | 0.7027 |
| C_4 | Gh_A02G1371 | At5g64970 | Probable mitochondrial adenine nucleotide transporter | -0.1059 | 0.8711 | -0.6053 | -0.5490 |
|  |  |  | BTL3 |  |  |  |  |
| C_4 | Gh_A02G1381 | TPPJ | Probable trehalose-phosphate phosphatase J | 0.6300 | 1.5164 | 1.5349 | 0.1647 |
| C_4 | Gh_A02G1425 | At5g47360 | Pentatricopeptide repeat-containing protein | 0.2480 | 0.5681 | 0.5695 | 0.3634 |
| C_4 | Gh_A02G1463 | At2g23070 | Casein kinase II subunit alpha, chloroplastic | -0.1735 | 0.4093 | -0.2192 | -0.2671 |
| C_4 | Gh_A02G1480 | At5g24010 | Probable receptor-like protein kinase | -0.1965 | 0.2542 | -0.9748 | -0.7681 |
| C_4 | Gh_A02G1598 | AUL1 | Auxilin-like protein 1 | 0.0173 | 0.0614 | -0.5967 | -0.4059 |
| C_4 | Gh_A02G1750 | NA | NA | 1.1461 | 2.6160 | 0.5324 | 0.2602 |
| C_4 | Gh_A03G0028 | NPF7.3 | Protein NRT1/ PTR FAMILY 7.3 | -0.1484 | 1.1428 | -0.2575 | -0.3619 |
| C_4 | Gh_A03G0047 | At2g01680 | Ankyrin repeat-containing protein | 1.1850 | 0.2612 | -1.9138 | -1.6026 |
| C_4 | Gh_A03G0082 | VAB | VAN3-binding protein | 0.0676 | 0.4921 | 0.4087 | -0.7073 |
| C_4 | Gh_A03G0238 | MIEL1 | E3 ubiquitin-protein ligase MIEL1 | 0.1302 | 1.2959 | 0.5554 | 0.2995 |
| C_4 | Gh_A03G0287 | NA | NA | -0.1386 | -0.5207 | -1.1237 | -1.0390 |
| C_4 | Gh_A03G0363 | TRP5 | Telomere repeat-binding protein 5 | -0.5580 | 1.4951 | 0.1658 | -0.0030 |
| C_4 | Gh_A03G0681 | NA | NA | -0.4987 | 3.3964 | 0.9241 | -0.2889 |
| C_4 | Gh_A03G0693 | PLP2 | Patatin-like protein 2 | -1.1526 | -1.1818 | -1.2610 | -3.4051 |
| C_4 | Gh_A03G0881 | APL | Myb family transcription factor APL | -0.6724 | 0.9570 | -0.7769 | -0.9941 |
| C_4 | Gh_A03G0929 | GEK1 | D-aminoacyl-tRNA deacylase | 0.1215 | 1.1381 | 0.6071 | 0.8708 |
| C_4 | Gh_A03G0984 | AHK4 | Histidine kinase 4 | -0.4926 | 0.6085 | -0.2045 | 0.1163 |
| C_4 | Gh_A03G1064 | LPR1 | Multicopper oxidase LPR1 | -0.1639 | 0.3452 | -0.3534 | -0.3816 |
| C_4 | Gh_A03G1076 | QSOX1 | Sulfhydryl oxidase 1 | -0.7332 | 0.4527 | -0.1390 | -0.2915 |
| C_4 | Gh_A03G1099 | hormad1 | HORMA domain-containing protein 1 | -0.4377 | 0.5152 | -0.2713 | -0.7863 |
| C_4 | Gh_A03G1109 | PUB6 | U-box domain-containing protein 6 | -0.4248 | 1.4495 | -0.3222 | -0.8410 |
| C_4 | Gh_A03G1140 | RIN4 | RPM1-interacting protein 4 | 0.2008 | 1.4359 | 0.5954 | -0.9591 |
| C_4 | Gh_A03G1169 | ATPA | ATP synthase subunit alpha, mitochondrial | 0.2150 | 2.0838 | 0.8521 | -0.4442 |
| C_4 | Gh_A03G1228 | PUB18 | U-box domain-containing protein 18 | -0.5618 | 0.0565 | -0.0123 | -1.0318 |
| C_4 | Gh_A03G1310 | At1g18390 | Probable serine/threonine-protein kinase | 0.4541 | -0.6784 | -3.2548 | -6.7577 |
| C_4 | Gh_A03G1363 | NA | NA | 0.4351 | 0.7873 | 0.3748 | 0.3200 |
| C_4 | Gh_A03G1486 | NA | NA | 0.1405 | 0.1015 | -0.5364 | -0.9918 |
| C_4 | Gh_A03G1672 | ATG2 | Autophagy-related protein 2 | -0.2665 | 0.7717 | -0.3655 | -0.3780 |
| C_4 | Gh_A03G1707 | TCP11L1 | T-complex protein 11-like protein 1 | -0.3840 | 0.8849 | 0.2664 | 0.5966 |
| C_4 | Gh_A03G1718 | NA | Polyphenol oxidase A1, chloroplastic | -0.0135 | 1.0841 | -0.0919 | 0.1859 |
| C_4 | Gh_A03G1802 | ATJ11 | Chaperone protein dnaJ 11, chloroplastic | -0.4312 | 2.5775 | 1.3477 | 0.1138 |
| C_4 | Gh_A03G1812 | APX3 | L-ascorbate peroxidase 3, peroxisomal | -0.1740 | 0.1307 | -0.2544 | -0.0761 |
| C_4 | Gh_A03G1817 | NA | NA | 0.3330 | 0.4564 | 0.5414 | -0.0029 |
| C_4 | Gh_A03G1840 | SAUR66 | Auxin-responsive protein SAUR66 | 0.2667 | 3.9283 | 2.0901 | -1.8736 |
| C_4 | Gh_A03G1870 | NA | NA | -0.6483 | 0.6592 | -0.5374 | -0.2122 |
| C_4 | Gh_A03G1889 | CBSDUF3 | DUF21 domain-containing protein | -0.9964 | 1.0588 | -1.3720 | -0.2276 |
| C_4 | Gh_A03G1943 | PEX5 | Peroxisome biogenesis protein 5 | 0.0630 | 0.6135 | 0.1476 | -0.0805 |
| C_4 | Gh_A03G2004 | AGL19 | Agamous-like MADS-box protein AGL19 | -0.6586 | 1.0678 | 0.9823 | -0.4149 |
| C_4 | Gh_A03G2005 | KNAT7 | Homeobox protein knotted-1-like 7 | -0.0135 | 0.4394 | -0.2746 | -0.4098 |
| C_4 | Gh_A03G2012 | NA | NA | -1.0537 | 1.8515 | 0.7444 | 0.6991 |
| C_4 | Gh_A03G2015 | DTX49 | Protein DETOXIFICATION 49 | 0.2930 | 0.2583 | -0.5103 | -0.7790 |
| C_4 | Gh_A03G2040 | NA | NA | -2.9072 | 1.6273 | -1.1760 | -0.6166 |
| C_4 | Gh_A03G2186 | EXLA1 | Expansin-like A1 | 0.5200 | 1.2218 | 0.7255 | -0.6282 |
| C_4 | Gh_A04G0063 | RLP12 | Receptor-like protein 12 | 0.5781 | 1.1533 | 0.5163 | 0.7559 |
| C_4 | Gh_A04G0180 | UBQ10 | Polyubiquitin 10 | 0.0439 | 0.7406 | -0.0377 | -0.1486 |
| C_4 | Gh_A04G0239 | NA | NA | -0.5061 | 0.9331 | -0.1917 | 0.2205 |
| C_4 | Gh_A04G0245 | CBSX5 | CBS domain-containing protein CBSX5 | 1.1675 | 1.1691 | 0.6096 | 0.6404 |
| C_4 | Gh_A04G0416 | BRG3 | Probable BOI-related E3 ubiquitin-protein ligase 3 | 0.4598 | -0.5492 | -0.9287 | -1.7171 |
| C_4 | Gh_A04G0720 | LECRKS4 | L-type lectin-domain containing receptor kinase S.4 | -0.6259 | 0.3172 | -0.0763 | 0.1532 |
| C_4 | Gh_A04G0735 | ROPGEF7 | Rop guanine nucleotide exchange factor 7 | -0.1307 | 0.9681 | -0.8102 | -0.8716 |
| C_4 | Gh_A04G0788 | NA | NA | 0.0723 | 0.1351 | -0.8965 | -0.6094 |
| C_4 | Gh_A04G0817 | SIEL | Protein SIEL | 0.1957 | 0.2535 | -0.0322 | -0.2384 |
| C_4 | Gh_A04G0844 | NA | NA | 0.6259 | 0.4366 | -0.8560 | -0.6123 |
| C_4 | Gh_A04G0849 | ARR4 | Two-component response regulator ARR4 | 0.8789 | 1.0440 | 0.3867 | -0.0803 |
| C_4 | Gh_A04G0903 | PORA | Protochlorophyllide reductase, chloroplastic | 0.7381 | -0.1316 | -0.7930 | -0.9410 |
| C_4 | Gh_A04G1076 | NA | Fructose-1,6-bisphosphatase, cytosolic | -0.3002 | 0.2233 | -0.2815 | -0.3160 |
| C_4 | Gh_A04G1093 | SQE5 | Squalene epoxidase 5 | -0.5456 | 0.3907 | 0.4481 | -0.8854 |
| C_4 | Gh_A04G1146 | BASS1 | Probable sodium/metabolite cotransporter BASS1, | 1.2450 | 2.2713 | 0.0797 | -0.6197 |
|  |  |  | chloroplastic |  |  |  |  |
| C_4 | Gh_A04G1149 | RPL3 | 60S ribosomal protein L3 | -0.2543 | 0.2128 | -0.4254 | -0.2246 |
| C_4 | Gh_A04G1210 | At1g67360 | REF/SRPP-like protein | -0.6013 | -0.1218 | -1.0817 | -0.6961 |
| C_4 | Gh_A04G1257 | AAPT1 | Choline/ethanolaminephosphotransferase 1 | 0.3168 | 0.1416 | 0.5145 | -0.5570 |
| C_4 | Gh_A04G1325 | NA | NA | -0.1681 | 2.5295 | -0.1242 | -0.6671 |
| C_4 | Gh_A05G0009 | BCDH | 2-oxoisovalerate dehydrogenase subunit beta 1, | -0.6284 | 0.9503 | -0.4602 | -0.1355 |
|  |  |  | mitochondrial |  |  |  |  |
| C_4 | Gh_A05G0085 | EBF1 | EIN3-binding F-box protein 1 | -0.0401 | 0.2939 | -0.3187 | -0.3073 |
| C_4 | Gh_A05G0095 | DBR | 2-alkenal reductase (NADP(+)-dependent) | -0.6296 | 0.7130 | -0.6534 | -0.5660 |
| C_4 | Gh_A05G0266 | NA | 21 kDa protein | -0.0200 | -0.4629 | -1.4989 | -1.2152 |
| C_4 | Gh_A05G0287 | XI-I | Myosin-15 | 0.1314 | 0.3710 | -0.2159 | -0.2971 |
| C_4 | Gh_A05G0302 | Slc25a44 | Solute carrier family 25 member 44 | -0.0896 | 1.3540 | 0.3039 | 0.8178 |
| C_4 | Gh_A05G0303 | NA | NA | -0.8681 | 1.3340 | -0.3943 | 0.5390 |
| C_4 | Gh_A05G0346 | At2g19810 | Zinc finger CCCH domain-containing protein 20 | 0.1090 | 0.4421 | -0.6724 | -1.1811 |
| C_4 | Gh_A05G0359 | SHH1 | Protein SAWADEE HOMEODOMAIN HOMOLOG 1 | -0.6756 | 1.3045 | 0.1901 | -3.2011 |
| C_4 | Gh_A05G0386 | NA | NA | -0.1908 | 0.3609 | -0.4410 | -0.4374 |
| C_4 | Gh_A05G0394 | NA | NA | -1.7934 | 0.0414 | -0.5041 | -0.5931 |
| C_4 | Gh_A05G0433 | NA | NA | 0.0414 | 0.6846 | 0.6051 | 0.0818 |
| C_4 | Gh_A05G0450 | APT5 | Adenine phosphoribosyltransferase 5 | 1.1306 | 2.4151 | 0.1145 | -0.5737 |
| C_4 | Gh_A05G0482 | CIPK5 | CBL-interacting serine/threonine-protein kinase 5 | 0.7473 | 0.9822 | 1.0608 | 0.3656 |
| C_4 | Gh_A05G0495 | At4g31860 | Probable protein phosphatase 2C 60 | -0.1202 | 0.2351 | -1.2070 | -0.6667 |
| C_4 | Gh_A05G0518 | GDU3 | Protein GLUTAMINE DUMPER 3 | -0.1200 | -0.8336 | -1.8800 | -1.7869 |
| C_4 | Gh_A05G0742 | rnf141 | RING finger protein 141 | 0.3946 | 0.7662 | -0.7933 | -0.7117 |
| C_4 | Gh_A05G0744 | At3g47120 | Zinc finger CCCH domain-containing protein 42 | 0.3290 | 0.3127 | 0.0073 | -0.4496 |
| C_4 | Gh_A05G0818 | HSP18.2 | 18.2 kDa class I heat shock protein | -3.0380 | 0.7021 | -2.4957 | -1.1415 |
| C_4 | Gh_A05G0831 | RABE1C | Ras-related protein RABE1c | -0.1581 | 0.6965 | -0.1802 | 0.0961 |
| C_4 | Gh_A05G0903 | CNGC15 | Putative cyclic nucleotide-gated ion channel 15 | 0.1597 | 1.3186 | -0.2288 | -0.0025 |
| C_4 | Gh_A05G0939 | At5g45840 | Probable LRR receptor-like serine/threonine-protein | 0.7060 | 1.2711 | 0.9329 | 0.9257 |
|  |  |  | kinase |  |  |  |  |
| C_4 | Gh_A05G0942 | FLX | Protein FLC EXPRESSOR | -0.2029 | 0.8277 | -0.7103 | -0.2234 |
| C_4 | Gh_A05G0949 | NAA25 | N-alpha-acetyltransferase 25, NatB auxiliary subunit | -0.0491 | 0.8157 | -0.1913 | -0.2540 |
| C_4 | Gh_A05G0951 | SDR2a | Short-chain dehydrogenase reductase 2a | 0.6290 | -0.9295 | -2.3831 | -5.4806 |
| C_4 | Gh_A05G1030 | RAE1 | Protein RAE1 | -0.0647 | 0.7393 | 0.3240 | -0.0827 |
| C_4 | Gh_A05G1048 | NA | Pyrophosphate-energized vacuolar membrane proton | -0.3069 | 0.7804 | 0.1452 | 0.0317 |
|  |  |  | pump |  |  |  |  |
| C_4 | Gh_A05G1099 | MIOX1 | Inositol oxygenase 1 | 0.6962 | 0.2980 | -2.6343 | -3.4990 |
| C_4 | Gh_A05G1152 | NA | NA | 0.1710 | 0.6521 | -0.0182 | -0.0640 |
| C_4 | Gh_A05G1177 | eif2b1 | Translation initiation factor eIF-2B subunit alpha | -2.2045 | 0.8358 | -1.7575 | -1.5661 |
| C_4 | Gh_A05G1205 | CERBERUS | Putative E3 ubiquitin-protein ligase LIN-1 | -0.1213 | 1.5313 | -1.7954 | -0.7178 |
| C_4 | Gh_A05G1210 | BRG1 | BOI-related E3 ubiquitin-protein ligase 1 | -0.0400 | 0.2828 | -0.0538 | -0.2021 |
| C_4 | Gh_A05G1239 | NA | Peroxisomal (S)-2-hydroxy-acid oxidase | -1.4309 | 0.1088 | -0.7732 | -0.2107 |
| C_4 | Gh_A05G1301 | SCAR3 | Protein SCAR3 | -0.3661 | 0.1702 | -0.3883 | -0.3127 |
| C_4 | Gh_A05G1369 | NA | NA | -0.5690 | 1.7746 | 0.1791 | -0.3875 |
| C_4 | Gh_A05G1419 | PCR8 | Protein PLANT CADMIUM RESISTANCE 8 | 0.0846 | 2.2864 | 0.2684 | 0.0545 |
| C_4 | Gh_A05G1428 | CXXS1 | Thioredoxin-like protein CXXS1 | -0.2185 | 0.8676 | -0.7927 | -0.0886 |
| C_4 | Gh_A05G1480 | CSC1 | Calcium permeable stress-gated cation channel 1 | 0.2292 | 2.5149 | 0.8107 | 0.7426 |
| C_4 | Gh_A05G1538 | NLP4 | Protein NLP4 | 0.2747 | 0.9093 | 0.4285 | 0.0687 |
| C_4 | Gh_A05G1539 | CAT1 | Catalase isozyme 1 | -0.2173 | 0.5074 | -1.6282 | -1.0495 |
| C_4 | Gh_A05G1554 | COR47 | Dehydrin COR47 | 1.0815 | 1.5777 | 0.9131 | 0.2434 |
| C_4 | Gh_A05G1572 | NIP1 | NEP1-interacting protein 1 | 3.3531 | 2.4148 | 2.1350 | 0.3007 |
| C_4 | Gh_A05G1594 | NA | Thaumatin-like protein 1 | -0.1274 | 1.1156 | 0.0889 | -0.6850 |
| C_4 | Gh_A05G1688 | Lmln | Leishmanolysin-like peptidase | -1.1391 | 0.3967 | -1.5630 | -0.7824 |
| C_4 | Gh_A05G1749 | trc | Serine/threonine-protein kinase tricorner | -0.3390 | 1.5631 | 0.5827 | 0.4286 |
| C_4 | Gh_A05G1769 | RAP2-1 | Ethylene-responsive transcription factor RAP2-1 | -0.1305 | 1.5736 | -0.1511 | -0.3373 |
| C_4 | Gh_A05G1776 | RD21A | Cysteine proteinase RD21a | -0.1809 | -0.2191 | -0.7689 | -0.6342 |
| C_4 | Gh_A05G1882 | AHL17 | AT-hook motif nuclear-localized protein 17 | 0.4355 | 1.3908 | 0.6434 | 0.7161 |
| C_4 | Gh_A05G1939 | At1g34750 | Probable protein phosphatase 2C 10 | -0.0872 | 0.3433 | 0.0072 | -0.1435 |
| C_4 | Gh_A05G1959 | TPPD | Probable trehalose-phosphate phosphatase D | 0.8387 | 0.2504 | -1.1260 | -0.9012 |
| C_4 | Gh_A05G1961 | RAP2-4 | Ethylene-responsive transcription factor RAP2-4 | 0.1996 | 1.0337 | -0.1817 | 0.2077 |
| C_4 | Gh_A05G1970 | ST2 | High affinity sulfate transporter 2 | -0.1816 | -0.9518 | -2.0627 | -2.9057 |
| C_4 | Gh_A05G1971 | ST3 | Low affinity sulfate transporter 3 | 0.7669 | 0.6707 | -1.0212 | -0.8261 |
| C_4 | Gh_A05G2024 | MBD9 | Methyl-CpG-binding domain-containing protein 9 | 0.2972 | 0.5747 | -0.3987 | -0.8547 |
| C_4 | Gh_A05G2044 | ARPN | Basic blue protein | 2.3023 | 1.3171 | 0.4593 | -0.6905 |
| C_4 | Gh_A05G2055 | GLTP3 | Glycolipid transfer protein 3 | -0.6423 | 1.0646 | -1.5618 | -0.4715 |
| C_4 | Gh_A05G2116 | LAC14 | Laccase-14 | 0.0205 | -0.2421 | -0.7527 | -0.8013 |
| C_4 | Gh_A05G2142 | NA | NA | -0.4586 | 0.5358 | -0.1632 | -1.4010 |
| C_4 | Gh_A05G2172 | P4H3 | Probable prolyl 4-hydroxylase 3 | -0.0536 | 1.1095 | 0.7117 | 0.8042 |
| C_4 | Gh_A05G2342 | MYB6 | Transcription repressor MYB6 | -0.1705 | 0.4241 | -0.8419 | -0.9672 |
| C_4 | Gh_A05G2482 | PU1 | Pullulanase 1, chloroplastic | 0.3895 | 0.4597 | 0.1760 | -0.0790 |
| C_4 | Gh_A05G2499 | DIR4 | Dirigent protein 4 | -1.8881 | -0.5307 | -0.5480 | -1.3939 |
| C_4 | Gh_A05G2531 | AVT1 | Vacuolar amino acid transporter 1 | -0.1006 | 0.0696 | -0.2306 | -0.9075 |
| C_4 | Gh_A05G2536 | Ccdc12 | Coiled-coil domain-containing protein 12 | 0.2889 | 0.5060 | -0.3846 | -0.4170 |
| C_4 | Gh_A05G2630 | PYL4 | Abscisic acid receptor PYL4 | -2.1308 | 0.6148 | -1.2276 | -1.6330 |
| C_4 | Gh_A05G2651 | NA | NA | 0.1974 | 0.4155 | -0.0746 | -0.5656 |
| C_4 | Gh_A05G2660 | BPA1 | Binding partner of ACD11 1 | -0.2605 | 1.3496 | 0.3874 | 0.1336 |
| C_4 | Gh_A05G2690 | CMTA4 | Calmodulin-binding transcription activator 4 | -0.2369 | 0.2415 | -0.1311 | -0.1353 |
| C_4 | Gh_A05G2731 | NA | NA | -0.2054 | 3.3986 | 0.4600 | -0.3653 |
| C_4 | Gh_A05G2826 | NA | NA | 0.0890 | 3.1167 | 0.3516 | -0.5091 |
| C_4 | Gh_A05G2828 | NA | NA | 0.0395 | 3.1144 | -0.0084 | -0.4653 |
| C_4 | Gh_A05G2829 | NA | NA | -0.3342 | 3.0529 | 0.0224 | 0.2903 |
| C_4 | Gh_A05G2830 | NA | NA | -0.0625 | 3.3409 | 0.0232 | -0.4570 |
| C_4 | Gh_A05G2831 | NA | NA | 0.0855 | 3.1139 | 0.5701 | 0.1065 |
| C_4 | Gh_A05G2833 | NA | NA | -0.0732 | 2.5702 | -0.4768 | -0.2348 |
| C_4 | Gh_A05G2834 | NA | NA | 0.0036 | 2.9947 | -0.2940 | -0.4542 |
| C_4 | Gh_A05G2835 | NA | NA | 0.0524 | 3.4479 | -0.6094 | -0.6009 |
| C_4 | Gh_A05G2897 | ERD15 | Protein EARLY RESPONSIVE TO DEHYDRATION 15 | -0.2884 | 0.3019 | -0.8130 | -0.6960 |
| C_4 | Gh_A05G3026 | At2g39795 | Uncharacterized protein | 0.1981 | -0.3528 | -0.6909 | -1.5260 |
| C_4 | Gh_A05G3041 | IDN2 | Protein INVOLVED IN DE NOVO 2 | 0.1931 | 0.0071 | -0.1555 | -0.1670 |
| C_4 | Gh_A05G3056 | ADF2 | Actin-depolymerizing factor 2 | 0.1001 | 1.4533 | 0.8837 | 0.4960 |
| C_4 | Gh_A05G3102 | NA | NA | 0.0877 | 0.2187 | -0.3608 | -0.3844 |
| C_4 | Gh_A05G3206 | ARID2 | AT-rich interactive domain-containing protein 2 | -0.4014 | -0.2613 | -0.4741 | -0.7615 |
| C_4 | Gh_A05G3622 | NA | NA | -0.0857 | 2.6788 | -0.0290 | -0.6326 |
| C_4 | Gh_A05G3625 | NA | NA | -0.4180 | 2.9356 | -0.8810 | -0.2642 |
| C_4 | Gh_A05G3628 | NA | NA | 0.0382 | 3.1498 | 0.2707 | -0.1094 |
| C_4 | Gh_A05G3713 | fam188a | Protein FAM188A | 0.0040 | 0.4995 | -0.1290 | 0.0971 |
| C_4 | Gh_A05G3717 | FPP | Filament-like plant protein (Fragment) | -0.0613 | 1.2090 | 0.3746 | 0.3320 |
| C_4 | Gh_A05G3734 | UDP-GALT1 | UDP-galactose transporter 1 | -0.3471 | 0.7468 | 0.2697 | -0.1524 |
| C_4 | Gh_A05G3738 | At1g21890 | WAT1-related protein | -0.4468 | 0.4300 | -1.2475 | -0.9717 |
| C_4 | Gh_A05G3746 | SOV | DIS3-like exonuclease 2 | -0.1227 | 0.7825 | -0.2754 | -0.1710 |
| C_4 | Gh_A05G3757 | NA | NA | -0.0286 | 0.2938 | 0.0466 | 0.0264 |
| C_4 | Gh_A05G3781 | IKU2 | Receptor-like protein kinase HAIKU2 | -0.1768 | -0.0865 | -0.5702 | -0.7494 |
| C_4 | Gh_A05G3807 | At2g30600/At | BTB/POZ domain-containing protein | -0.5201 | 0.3719 | -0.9202 | -0.4614 |
|  |  | 2g30610 |  |  |  |  |  |
| C_4 | Gh_A05G3862 | At3g15810 | Protein LURP-one-related 12 | 0.1336 | 0.2883 | 0.7762 | -0.3951 |
| C_4 | Gh_A05G3880 | PRUNE | Protein prune homolog | -1.3244 | -0.4588 | -0.6509 | -1.6262 |
| C_4 | Gh_A05G3885 | At1g30630 | Coatomer subunit epsilon-1 | 0.2067 | -0.2261 | -0.2769 | -0.7224 |
| C_4 | Gh_A05G3918 | PMA4 | Plasma membrane ATPase 4 | -1.0671 | -0.0830 | -0.5959 | -1.8479 |
| C_4 | Gh_A05G3961 | BT1 | Adenine nucleotide transporter BT1, | 0.1615 | 1.6980 | 0.1198 | -0.0750 |
|  |  |  | chloroplastic/mitochondrial |  |  |  |  |
| C_4 | Gh_A05G3963 | SGS3 | Protein SUPPRESSOR OF GENE SILENCING 3 | 0.4466 | 2.4689 | 0.2781 | 0.1131 |
| C_4 | Gh_A05G4003 | NA | NA | -0.3436 | 0.6297 | -0.4552 | -0.4907 |
| C_4 | Gh_A05G4004 | At5g57480 | AAA-ATPase | 0.0548 | 1.6157 | -0.1740 | 0.6625 |
| C_4 | Gh_A06G0062 | NA | NA | -0.4912 | -1.3651 | -2.9825 | -3.0122 |
| C_4 | Gh_A06G0111 | CYP74A | Allene oxide synthase, chloroplastic | -5.2691 | 1.4735 | -1.3971 | -4.0433 |
| C_4 | Gh_A06G0174 | ERF008 | Ethylene-responsive transcription factor ERF008 | -0.3313 | 0.3319 | -1.3583 | -1.1195 |
| C_4 | Gh_A06G0226 | NA | NA | -1.7208 | 1.7065 | 2.3958 | -3.6445 |
| C_4 | Gh_A06G0240 | At5g65660 | Uncharacterized protein | 0.1974 | 0.4591 | 0.6025 | -0.3029 |
| C_4 | Gh_A06G0241 | BHLH93 | Transcription factor bHLH93 | -0.8012 | 1.3438 | -1.1188 | -0.1030 |
| C_4 | Gh_A06G0543 | COV1 | Protein CONTINUOUS VASCULAR RING 1 | -0.2449 | 0.7385 | 0.1441 | 0.4322 |
| C_4 | Gh_A06G0593 | NA | NA | 0.1413 | 1.0656 | -0.3918 | -0.8435 |
| C_4 | Gh_A06G0665 | NA | NA | -1.1973 | -0.1683 | -0.9978 | -0.6177 |
| C_4 | Gh_A06G0697 | At4g29890 | Choline monooxygenase, chloroplastic | -0.5049 | 0.0507 | 0.0878 | -0.4746 |
| C_4 | Gh_A06G0735 | AIP2 | E3 ubiquitin-protein ligase AIP2 | -0.1769 | 0.4385 | 0.2445 | -0.1567 |
| C_4 | Gh_A06G0928 | LIN | Putative E3 ubiquitin-protein ligase LIN-2 | 0.5668 | 1.4490 | -0.2872 | -0.9748 |
| C_4 | Gh_A06G0941 | MGD1 | Monogalactosyldiacylglycerol synthase 1, chloroplastic | 0.0657 | 1.7378 | 1.0238 | -0.2226 |
| C_4 | Gh_A06G1214 | AERO1 | Endoplasmic reticulum oxidoreductin-1 | -0.1540 | 1.0062 | 0.7192 | 0.6353 |
| C_4 | Gh_A06G1279 | NA | Pectinesterase/pectinesterase inhibitor | -0.5272 | 0.6681 | -1.1339 | -0.4675 |
| C_4 | Gh_A06G1282 | RPL5 | 60S ribosomal protein L5, mitochondrial | 0.4822 | 1.3743 | 0.7204 | -0.1854 |
| C_4 | Gh_A06G1301 | At1g61900 | Uncharacterized GPI-anchored protein | -0.1106 | 0.4639 | -0.0355 | 0.0172 |
| C_4 | Gh_A06G1327 | NA | NA | 0.1104 | 1.1641 | 0.0433 | 0.1880 |
| C_4 | Gh_A06G1403 | slc38a7 | Putative sodium-coupled neutral amino acid transporter 7 | -0.4591 | -0.4253 | 0.0647 | -1.4386 |
| C_4 | Gh_A06G1440 | PLC2 | Phosphoinositide phospholipase C 2 | -0.3122 | 1.4939 | -0.6474 | -0.1944 |
| C_4 | Gh_A06G1453 | NA | NA | -0.0151 | 0.2624 | -0.5309 | -0.2930 |
| C_4 | Gh_A06G1552 | XYLA | Xylose isomerase | -0.0586 | -0.9232 | -3.1187 | -5.9077 |
| C_4 | Gh_A06G1626 | RPS5 | Disease resistance protein RPS5 | -0.6035 | 0.5352 | -4.1592 | -5.1506 |
| C_4 | Gh_A06G1629 | At1g61310 | Probable disease resistance protein | 0.1000 | 1.4533 | -0.4333 | -0.1000 |
| C_4 | Gh_A06G1757 | PUX4 | Plant UBX domain-containing protein 4 | -0.2381 | 1.6220 | 0.5010 | -0.4967 |
| C_4 | Gh_A06G1842 | CKX5 | Cytokinin dehydrogenase 5 | -0.2205 | 1.3797 | 0.3898 | 0.8664 |
| C_4 | Gh_A06G1905 | SPA2 | Protein SPA1-RELATED 2 | 0.0997 | 0.4527 | -0.6834 | -0.5864 |
| C_4 | Gh_A06G2045 | EBF1 | EIN3-binding F-box protein 1 | 0.2258 | 0.6994 | -0.0359 | 0.0868 |
| C_4 | Gh_A06G2111 | KOR | Endoglucanase 25 | 0.1694 | 0.1648 | 0.1615 | 0.0990 |
| C_4 | Gh_A07G0057 | RAB5 | Ras-related protein Rab5 | -0.0748 | -0.0632 | -1.8241 | -2.8147 |
| C_4 | Gh_A07G0083 | NA | NA | -0.1826 | 1.4190 | -0.8313 | -0.3658 |
| C_4 | Gh_A07G0084 | NA | NA | -0.7610 | 1.8469 | 1.0378 | 0.4261 |
| C_4 | Gh_A07G0249 | NA | NA | 0.0498 | 0.3512 | 0.1387 | 0.0625 |
| C_4 | Gh_A07G0379 | ERF2 | Ethylene-responsive transcription factor 2 | -0.8833 | 0.2233 | -1.9560 | -1.1106 |
| C_4 | Gh_A07G0568 | NA | NA | -0.5334 | -0.1054 | -0.2670 | -0.7900 |
| C_4 | Gh_A07G0623 | HSP18.2 | 18.2 kDa class I heat shock protein | -1.3395 | -0.0436 | -0.5419 | -1.1367 |
| C_4 | Gh_A07G0626 | At1g35710 | Probable leucine-rich repeat receptor-like protein kinase | 0.4271 | 1.1162 | 1.0629 | 0.6434 |
| C_4 | Gh_A07G1121 | TKPR1 | Tetraketide alpha-pyrone reductase 1 | -0.7273 | 0.8080 | -0.0170 | 0.1475 |
| C_4 | Gh_A07G1148 | kz | Probable ATP-dependent RNA helicase kurz | -0.1232 | 0.1172 | -0.0922 | -0.2185 |
| C_4 | Gh_A07G1184 | GA2OX2 | Gibberellin 2-beta-dioxygenase 2 | 0.5129 | 1.1418 | 0.8725 | 0.2235 |
| C_4 | Gh_A07G1191 | TRMT13 | tRNA:m(4)X modification enzyme TRM13 homolog | -0.1214 | 0.0546 | -0.0881 | -0.0970 |
| C_4 | Gh_A07G1194 | PDR3 | Pleiotropic drug resistance protein 3 | 0.2378 | 0.9187 | -2.7477 | -2.7774 |
| C_4 | Gh_A07G1241 | kif11 | Kinesin-related protein 11 | 0.1489 | 0.4684 | -0.7890 | -0.3012 |
| C_4 | Gh_A07G1246 | ALMT10 | Aluminum-activated malate transporter 10 | -0.5173 | 1.1637 | -0.2835 | -1.9035 |
| C_4 | Gh_A07G1297 | MPK4 | Mitogen-activated protein kinase 4 | 0.6752 | 0.2001 | 0.0704 | -0.1399 |
| C_4 | Gh_A07G1327 | NA | NA | -0.2845 | 0.5732 | -0.6147 | -0.6444 |
| C_4 | Gh_A07G1491 | At3g07620 | Probable glycosyltransferase | -0.2698 | 0.5328 | -1.0217 | -0.7512 |
| C_4 | Gh_A07G1589 | POX2 | Proline dehydrogenase 2, mitochondrial | 0.0043 | 0.2289 | -0.4059 | -0.2503 |
| C_4 | Gh_A07G1653 | DAO | 2-oxoglutarate-dependent dioxygenase DAO | -0.6650 | 1.0794 | -0.0327 | 0.0929 |
| C_4 | Gh_A07G1678 | NA | NA | 0.7501 | 1.3115 | -0.5872 | -2.0670 |
| C_4 | Gh_A07G1689 | NA | NA | -0.5810 | 3.1379 | -0.1141 | -0.3020 |
| C_4 | Gh_A07G1694 | NA | NA | -0.6396 | 2.5666 | -0.0200 | -0.1263 |
| C_4 | Gh_A07G1695 | NA | NA | 0.0345 | 2.7467 | 0.3424 | -0.0186 |
| C_4 | Gh_A07G1696 | NA | NA | -0.0260 | 2.7787 | 0.0178 | -0.3505 |
| C_4 | Gh_A07G1697 | NA | NA | -0.0429 | 2.9496 | -0.1281 | -0.3630 |
| C_4 | Gh_A07G1742 | FLA2 | Fasciclin-like arabinogalactan protein 2 | 0.0942 | 1.0137 | 0.0813 | 0.4656 |
| C_4 | Gh_A07G1907 | GAUT6 | Probable galacturonosyltransferase 6 | -0.5068 | 0.4063 | 0.3768 | -0.1299 |
| C_4 | Gh_A07G1989 | ABCB15 | ABC transporter B family member 15 | 0.1854 | 0.6380 | -0.2409 | -0.2250 |
| C_4 | Gh_A07G2134 | GRXC1 | Glutaredoxin-C1 | -0.5416 | 1.7663 | -0.2883 | -3.3826 |
| C_4 | Gh_A07G2151 | PEX5 | Peroxisome biogenesis protein 5 | 0.2842 | 0.7460 | -0.0764 | -0.0477 |
| C_4 | Gh_A07G2288 | At4g29190 | Zinc finger CCCH domain-containing protein 49 | -0.1220 | 0.9690 | -1.0838 | -0.6877 |
| C_4 | Gh_A07G2297 | NA | Early nodulin-93 | -0.2059 | -0.3778 | -0.1548 | -1.0923 |
| C_4 | Gh_A08G0149 | Nol8 | Nucleolar protein 8 | 0.1205 | -0.2470 | -0.6747 | -1.0169 |
| C_4 | Gh_A08G0173 | MORF9 | Multiple organellar RNA editing factor 9, chloroplastic | 0.3157 | 0.4843 | -0.4684 | -0.5466 |
| C_4 | Gh_A08G0192 | COL5 | Zinc finger protein CONSTANS-LIKE 5 | -0.3351 | 0.7245 | 0.2014 | 0.3273 |
| C_4 | Gh_A08G0193 | Os07g0190000 | Probable 1-deoxy-D-xylulose-5-phosphate synthase 2, | -0.3631 | 1.5911 | -0.5325 | -0.5340 |
|  |  |  | chloroplastic |  |  |  |  |
| C_4 | Gh_A08G0196 | CHLN | Nicotianamine synthase | 0.5302 | 0.8807 | 1.6215 | -0.2860 |
| C_4 | Gh_A08G0286 | At4g00740 | Probable methyltransferase PMT13 | -0.0111 | 0.1077 | 1.6483 | -5.9571 |
| C_4 | Gh_A08G0330 | Mgst3 | Microsomal glutathione S-transferase 3 | -0.2009 | 0.3070 | -0.0124 | 0.0280 |
| C_4 | Gh_A08G0383 | XXT2 | Xyloglucan 6-xylosyltransferase 2 | 0.2740 | 0.3611 | 0.3265 | 0.1290 |
| C_4 | Gh_A08G0400 | BPM2 | BTB/POZ and MATH domain-containing protein 2 | 0.0660 | 0.6231 | 0.2035 | 0.1015 |
| C_4 | Gh_A08G0409 | At3g63340 | Probable protein phosphatase 2C 51 | -0.4664 | -0.1423 | -0.1484 | -0.5635 |
| C_4 | Gh_A08G0547 | NA | NA | 0.4273 | 0.7935 | 0.3082 | 0.2512 |
| C_4 | Gh_A08G0678 | IDD2 | Protein indeterminate-domain 2 | 0.0179 | 0.2262 | -1.6437 | -0.9680 |
| C_4 | Gh_A08G0727 | TBC1D5 | TBC1 domain family member 5 | -0.0798 | 0.5961 | 0.0513 | -0.1459 |
| C_4 | Gh_A08G0898 | VPS26A | Vacuolar protein sorting-associated protein 26A | -0.8798 | -0.1859 | -0.3804 | -0.4300 |
| C_4 | Gh_A08G0951 | NA | NA | 1.3006 | 2.3501 | -0.2229 | 0.7093 |
| C_4 | Gh_A08G0962 | BHLH14 | Transcription factor bHLH14 | 0.9404 | 2.4594 | -3.7628 | -1.7973 |
| C_4 | Gh_A08G1005 | NA | NA | -0.0892 | 0.3739 | -2.3629 | -1.4452 |
| C_4 | Gh_A08G1082 | PALD1 | Paladin | -0.6323 | 0.5606 | -0.6690 | -0.2580 |
| C_4 | Gh_A08G1133 | NA | NA | 0.1473 | 0.6964 | 0.6301 | 0.0074 |
| C_4 | Gh_A08G1206 | NA | NA | -0.2693 | 1.0386 | 0.4466 | -0.0988 |
| C_4 | Gh_A08G1244 | ppt-1 | Palmitoyl-protein thioesterase 1 | -0.8099 | 1.2722 | -0.2104 | 0.2362 |
| C_4 | Gh_A08G1329 | ANK2 | Ankyrin-2 | 0.2227 | 0.0790 | -0.3781 | -0.6119 |
| C_4 | Gh_A08G1354 | NA | NA | -0.5445 | 0.4751 | -0.8224 | -0.2332 |
| C_4 | Gh_A08G1395 | NA | NA | -0.3249 | 0.1353 | -0.6505 | -0.7620 |
| C_4 | Gh_A08G1475 | CAT6 | Cationic amino acid transporter 6, chloroplastic | 0.3512 | -0.1030 | -0.1740 | -1.9338 |
| C_4 | Gh_A08G1616 | RBL2 | RHOMBOID-like protein 2 | -0.0149 | 1.5618 | 0.6199 | 0.8933 |
| C_4 | Gh_A08G1623 | UGE1 | Bifunctional UDP-glucose 4-epimerase and UDP-xylose | -0.0836 | 1.6893 | -0.2041 | -0.1210 |
|  |  |  | 4-epimerase 1 |  |  |  |  |
| C_4 | Gh_A08G1660 | NA | NA | 0.3770 | 2.4681 | 0.4284 | -0.4729 |
| C_4 | Gh_A08G1709 | TOM2AH1 | Tetraspanin-20 | 0.0621 | 0.8474 | -0.4899 | -0.1063 |
| C_4 | Gh_A08G1783 | SCPL48 | Serine carboxypeptidase-like 48 | -0.6438 | 1.1144 | -0.5133 | -0.1438 |
| C_4 | Gh_A08G1787 | NFD4 | Protein NUCLEAR FUSION DEFECTIVE 4 | -0.8709 | 0.2939 | -1.1468 | -1.1912 |
| C_4 | Gh_A08G1889 | GID1C | Gibberellin receptor GID1C | -0.1110 | 0.0789 | -0.2961 | -0.4737 |
| C_4 | Gh_A08G1891 | GSH3 | Probable glutamate dehydrogenase 3 | -0.8334 | 0.5966 | -0.8961 | -0.6444 |
| C_4 | Gh_A08G1944 | SYP131 | Putative syntaxin-131 | 0.0352 | 0.5932 | 0.5839 | 0.1747 |
| C_4 | Gh_A08G2080 | ATL56 | RING-H2 finger protein ATL56 | 0.5539 | 0.7593 | 0.1694 | -0.5280 |
| C_4 | Gh_A08G2112 | EBF1 | EIN3-binding F-box protein 1 | 0.5306 | 0.5409 | 0.2875 | 0.3326 |
| C_4 | Gh_A08G2192 | ABI2 | Protein phosphatase 2C 77 | -0.6360 | 0.8856 | 0.3564 | 0.5541 |
| C_4 | Gh_A08G2230 | At4g29190 | Zinc finger CCCH domain-containing protein 49 | -0.2771 | 0.7530 | -0.5432 | -0.8067 |
| C_4 | Gh_A08G2232 | NA | NA | -0.3488 | 0.7963 | 0.1958 | 0.2161 |
| C_4 | Gh_A08G2318 | ppk15 | Serine/threonine-protein kinase ppk15 | 0.3643 | 0.4980 | 0.3230 | 0.1148 |
| C_4 | Gh_A08G2329 | At5g63930 | Probable leucine-rich repeat receptor-like protein kinase | -0.4871 | 0.1248 | -0.8301 | -0.5099 |
| C_4 | Gh_A09G0126 | At5g39570 | Uncharacterized protein | 0.6974 | -0.3907 | -2.3121 | -2.3417 |
| C_4 | Gh_A09G0225 | WRKY72 | Probable WRKY transcription factor 72 | 0.2233 | 0.3457 | 0.3732 | -0.1467 |
| C_4 | Gh_A09G0334 | LHT1 | Lysine histidine transporter 1 | -0.1811 | -0.5231 | -0.5538 | -0.9056 |
| C_4 | Gh_A09G0643 | SCPL33 | Serine carboxypeptidase-like 33 | -0.7600 | -0.2018 | -0.3912 | -0.8291 |
| C_4 | Gh_A09G0655 | VCR | Varicose-related protein | 0.0407 | 0.0742 | -0.3895 | -0.5421 |
| C_4 | Gh_A09G0736 | NPR3 | Regulatory protein NPR3 | 0.0781 | 0.4233 | 0.2704 | -0.2149 |
| C_4 | Gh_A09G0776 | NA | NA | 0.1113 | 1.9307 | -0.5616 | -0.5849 |
| C_4 | Gh_A09G0864 | AXS2 | UDP-D-apiose/UDP-D-xylose synthase 2 | -0.1422 | 0.5911 | 0.0611 | -0.1136 |
| C_4 | Gh_A09G0914 | DIVARICAT | Transcription factor DIVARICATA | -0.4531 | 0.9403 | -1.0016 | -0.4713 |
|  |  | A |  |  |  |  |  |
| C_4 | Gh_A09G0972 | NA | Calmodulin | 0.3643 | 0.9692 | 0.2706 | -0.0361 |
| C_4 | Gh_A09G1328 | NA | Tropinone reductase-like 1 | -0.8618 | 1.4158 | -2.9876 | -1.5196 |
| C_4 | Gh_A09G1347 | F6'H1 | Feruloyl CoA ortho-hydroxylase 1 | 0.9983 | 1.0859 | 0.8487 | 0.2469 |
| C_4 | Gh_A09G1546 | UBC5 | Ubiquitin-conjugating enzyme E2 5 | -0.4810 | 0.2300 | -0.2717 | -0.8275 |
| C_4 | Gh_A09G1579 | NA | NA | -0.0748 | 0.4580 | 0.9818 | -0.4143 |
| C_4 | Gh_A09G1600 | BHLH126 | Transcription factor bHLH126 | 0.4243 | 1.5099 | -0.8194 | -1.1053 |
| C_4 | Gh_A09G1609 | RBL3 | RHOMBOID-like protein 3 | 0.0028 | 0.7522 | 0.1831 | 0.2489 |
| C_4 | Gh_A09G1692 | CSLG3 | Cellulose synthase-like protein G3 | -1.7662 | 0.5905 | -0.3051 | -0.8353 |
| C_4 | Gh_A09G1776 | At3g50808 | Uncharacterized protein | -0.6824 | -0.4894 | -0.8566 | -1.2444 |
| C_4 | Gh_A09G1837 | TRM32 | Protein TRM32 | -0.0047 | 0.4679 | 0.3008 | 0.0382 |
| C_4 | Gh_A09G2018 | surf6 | Surfeit locus protein 6 homolog | 0.2658 | 0.6976 | 0.2120 | -0.3142 |
| C_4 | Gh_A09G2076 | RPL19B | 60S ribosomal protein L19-2 | -0.0364 | -0.0111 | -1.2647 | -1.0879 |
| C_4 | Gh_A09G2376 | TPX2 | Protein TPX2 | -0.1279 | 0.3303 | 0.2477 | -0.0549 |
| C_4 | Gh_A09G2398 | ARR6 | Two-component response regulator ARR6 | 0.5789 | 1.4185 | 0.2035 | -0.3664 |
| C_4 | Gh_A09G2441 | IPCS1 | Phosphatidylinositol:ceramide inositolphosphotransferase 1 | 0.3054 | 1.5562 | 0.8147 | 0.3031 |
| C_4 | Gh_A09G2467 | rbcL | Ribulose bisphosphate carboxylase large chain | -0.1808 | 2.2959 | 0.7379 | -1.0012 |
| C_4 | Gh_A09G2515 | NA | NA | -0.0926 | 2.2132 | -0.0568 | -0.1354 |
| C_4 | Gh_A09G2525 | NA | NA | 0.3197 | 3.8278 | 0.3300 | -0.2360 |
| C_4 | Gh_A09G2528 | NA | NA | 1.1249 | 4.6765 | -0.6213 | 0.3800 |
| C_4 | Gh_A09G2529 | NA | NA | -0.1139 | 3.1173 | -0.0354 | 0.1261 |
| C_4 | Gh_A10G0055 | At5g44450 | Alpha N-terminal protein methyltransferase 1 | -0.0639 | 0.7146 | -0.9685 | -0.7513 |
| C_4 | Gh_A10G0074 | NA | NA | -0.2197 | 0.8701 | -0.3967 | -0.0638 |
| C_4 | Gh_A10G0101 | NA | NA | 3.3149 | 2.3200 | 0.5212 | -0.7870 |
| C_4 | Gh_A10G0212 | NA | NA | 0.0238 | 0.3889 | -0.6401 | -0.9856 |
| C_4 | Gh_A10G0238 | TSJT1 | Stem-specific protein TSJT1 | -1.0112 | 0.8599 | -1.2226 | -0.4628 |
| C_4 | Gh_A10G0254 | ERF12 | Ethylene-responsive transcription factor 12 | 0.6232 | 0.5724 | -0.4567 | -0.7592 |
| C_4 | Gh_A10G0297 | NA | Xyloglucan endotransglucosylase/hydrolase 2 | 0.1151 | 1.0276 | -0.4916 | -0.5819 |
| C_4 | Gh_A10G0299 | SRG1 | Protein SRG1 | -2.5022 | -1.0718 | -2.1152 | -1.9433 |
| C_4 | Gh_A10G0305 | STY17 | Serine/threonine-protein kinase STY17 | -0.3543 | 1.6965 | -0.2253 | 0.4075 |
| C_4 | Gh_A10G0322 | RABA1C | Ras-related protein RABA1c | 0.0757 | 0.9077 | 0.2430 | 0.3010 |
| C_4 | Gh_A10G0378 | KAT1 | Potassium channel KAT1 | 0.9791 | 2.2841 | 0.2611 | -2.0001 |
| C_4 | Gh_A10G0423 | BRG1 | BOI-related E3 ubiquitin-protein ligase 1 | 0.1540 | -0.3801 | -0.4110 | -1.2301 |
| C_4 | Gh_A10G0455 | NA | NA | 0.1812 | 1.1902 | 0.0936 | -0.1370 |
| C_4 | Gh_A10G0478 | SAUR41 | Auxin-responsive protein SAUR41 | 1.4295 | 1.2982 | 1.1670 | 0.6252 |
| C_4 | Gh_A10G0510 | PAXBP1 | PAX3- and PAX7-binding protein 1 | -0.1399 | 0.4308 | -0.1002 | -0.5468 |
| C_4 | Gh_A10G0707 | MTERF3 | Transcription termination factor 3, mitochondrial | -0.0790 | 0.4758 | -0.6477 | -0.7918 |
| C_4 | Gh_A10G0708 | bshA | N-acetyl-alpha-D-glucosaminyl L-malate synthase | -0.1015 | 0.3959 | 0.4400 | -0.0551 |
| C_4 | Gh_A10G0742 | COX2 | Cytochrome c oxidase subunit 2 | 0.0980 | 2.0840 | -0.1636 | -0.6597 |
| C_4 | Gh_A10G0803 | At2g37240 | Thioredoxin-like protein AAED1, chloroplastic | -0.5740 | 0.1022 | -3.1483 | -3.0040 |
| C_4 | Gh_A10G0858 | LAC11 | Laccase-11 | 0.1772 | 0.6039 | 0.5425 | 0.0808 |
| C_4 | Gh_A10G1134 | GAE6 | UDP-glucuronate 4-epimerase 6 | 0.0192 | 0.2742 | -0.1899 | -0.5645 |
| C_4 | Gh_A10G1188 | clpS | ATP-dependent Clp protease adapter protein ClpS | -0.0702 | 0.1644 | 0.4234 | -0.8427 |
| C_4 | Gh_A10G1359 | FER | Receptor-like protein kinase FERONIA | 0.2451 | 0.8577 | 0.3643 | 0.6169 |
| C_4 | Gh_A10G1490 | NA | NA | 1.7308 | 2.2456 | 1.0523 | -3.3958 |
| C_4 | Gh_A10G1546 | NA | NA | 1.1082 | 1.2692 | 0.2925 | 0.4431 |
| C_4 | Gh_A10G1570 | UBP12 | Ubiquitin carboxyl-terminal hydrolase 12 | 1.7730 | 1.3281 | -1.3252 | -1.2121 |
| C_4 | Gh_A10G1703 | Os01g0270100 | Cysteine proteinase inhibitor 12 | -0.0185 | -0.0678 | -0.1392 | -0.3018 |
| C_4 | Gh_A10G1715 | PERK8 | Proline-rich receptor-like protein kinase PERK8 | 0.0641 | -0.0795 | -0.3268 | -0.4316 |
| C_4 | Gh_A10G1783 | Os11g0148500 | Pyruvate kinase 1, cytosolic | 0.1221 | 0.2636 | 0.3107 | -0.0545 |
| C_4 | Gh_A10G1785 | HI_0077 | Uncharacterized protein HI_0077 | 1.1376 | 0.8038 | -0.4860 | -1.6417 |
| C_4 | Gh_A10G1864 | At3g12620 | Probable protein phosphatase 2C 38 | 0.3413 | 0.2376 | 0.1224 | -0.0474 |
| C_4 | Gh_A10G1930 | NA | NA | 2.5795 | 2.0840 | -0.3520 | -1.5825 |
| C_4 | Gh_A10G1934 | NA | NA | -0.1152 | 0.6395 | 0.1526 | 0.0937 |
| C_4 | Gh_A10G1937 | UXS6 | UDP-glucuronic acid decarboxylase 6 | -0.0988 | 0.6560 | 0.1305 | 0.4321 |
| C_4 | Gh_A10G1973 | APK1A | Protein kinase APK1A, chloroplastic | -0.1617 | 0.0551 | -0.3896 | -0.4501 |
| C_4 | Gh_A10G2085 | At3g50280 | Uncharacterized acetyltransferase | -0.2408 | 1.4776 | 0.7685 | 1.0934 |
| C_4 | Gh_A10G2175 | NA | NA | 0.3992 | 3.1030 | -0.2229 | -0.2525 |
| C_4 | Gh_A10G2185 | CKX6 | Cytokinin dehydrogenase 6 | 0.3017 | 2.0847 | -1.6320 | -0.4938 |
| C_4 | Gh_A10G2192 | NA | NA | -1.7956 | 0.5392 | -1.6418 | -0.8616 |
| C_4 | Gh_A10G2222 | At1g29660 | GDSL esterase/lipase | 2.5422 | 3.8950 | 0.2011 | 0.3212 |
| C_4 | Gh_A10G2297 | At1g04430 | Probable methyltransferase PMT8 | 0.3987 | 0.5019 | 0.4897 | 0.0665 |
| C_4 | Gh_A10G2305 | CLH2 | Chlorophyllase-2, chloroplastic | 5.0745 | 3.8487 | -0.4276 | -4.5345 |
| C_4 | Gh_A10G2346 | SYT3 | Synaptotagmin-3 | -0.3048 | 0.7156 | 0.0346 | 0.0803 |
| C_4 | Gh_A11G0010 | PANK2 | Pantothenate kinase 2 | 0.0923 | -0.2031 | -0.0121 | -0.8082 |
| C_4 | Gh_A11G0055 | Lipn | Lipase member N | -0.0867 | 0.6944 | -0.1483 | -0.3483 |
| C_4 | Gh_A11G0124 | COL5 | Zinc finger protein CONSTANS-LIKE 5 | -0.7638 | 1.5028 | -0.6623 | 0.3948 |
| C_4 | Gh_A11G0126 | CBL4 | Calcineurin B-like protein 4 | -0.2505 | 0.8397 | -0.1912 | -0.0001 |
| C_4 | Gh_A11G0203 | GA3OX4 | Gibberellin 3-beta-dioxygenase 4 | -1.2158 | 0.4648 | -1.6430 | -1.2444 |
| C_4 | Gh_A11G0223 | GSVIVT0002 | Probable polygalacturonase | 0.1384 | 1.0804 | -0.0653 | 0.1524 |
|  |  | 6920001 |  |  |  |  |  |
| C_4 | Gh_A11G0338 | KING1 | SNF1-related protein kinase regulatory subunit gamma-1 | 0.0332 | 0.9646 | 0.1752 | 0.2353 |
| C_4 | Gh_A11G0379 | NA | NA | -0.0203 | 0.3425 | -0.8063 | -0.6424 |
| C_4 | Gh_A11G0422 | MES10 | Methylesterase 10 | 1.8713 | 3.1182 | -2.0706 | -1.4248 |
| C_4 | Gh_A11G0430 | NA | Lipase | -0.8307 | 0.0919 | -0.1783 | -0.6386 |
| C_4 | Gh_A11G0459 | BHLH19 | Transcription factor bHLH19 | 0.0003 | 0.6712 | 0.9197 | -0.4584 |
| C_4 | Gh_A11G0792 | NA | Remorin | 0.4210 | 1.9527 | 0.3084 | 0.1311 |
| C_4 | Gh_A11G0802 | ASK21 | SKP1-like protein 21 | -0.3009 | 0.4640 | 0.2534 | -0.7470 |
| C_4 | Gh_A11G0835 | LEA14-A | Late embryogenesis abundant protein Lea14-A | 0.7014 | 2.8034 | 1.0458 | 0.0402 |
| C_4 | Gh_A11G0844 | At3g61590 | F-box/kelch-repeat protein | 0.5630 | 0.6642 | 0.6990 | 0.0478 |
| C_4 | Gh_A11G0870 | PYL9 | Abscisic acid receptor PYL9 | -0.4215 | -0.2012 | -1.0419 | -0.7538 |
| C_4 | Gh_A11G0931 | NAC014 | NAC domain-containing protein 14 | 0.1656 | -0.2416 | -0.2754 | -1.3098 |
| C_4 | Gh_A11G0941 | PALD1 | Paladin | -1.5521 | 0.4707 | -0.2050 | -0.4714 |
| C_4 | Gh_A11G0997 | WRKY65 | Probable WRKY transcription factor 65 | 0.1677 | 1.7607 | -0.4788 | -0.5055 |
| C_4 | Gh_A11G1298 | At3g17800 | UV-B-induced protein At3g17800, chloroplastic | -0.0340 | 0.7382 | -0.8439 | -0.5089 |
| C_4 | Gh_A11G1346 | At3g07570 | Cytochrome b561 and DOMON domain-containing | -0.1878 | 0.4343 | -0.9758 | -0.8648 |
|  |  |  | protein |  |  |  |  |
| C_4 | Gh_A11G1551 | POX2 | Proline dehydrogenase 2, mitochondrial | -1.4526 | 0.5645 | -0.2655 | 0.2094 |
| C_4 | Gh_A11G1659 | NA | NA | 0.4152 | 2.1755 | 0.0707 | -0.5191 |
| C_4 | Gh_A11G1673 | At1g43910 | AAA-ATPase | -0.2873 | 0.3626 | 0.4144 | -0.1527 |
| C_4 | Gh_A11G1679 | At2g18193 | AAA-ATPase | 0.4040 | 0.8576 | 0.8474 | 0.5128 |
| C_4 | Gh_A11G1866 | NA | NA | 0.0908 | 0.7889 | 0.4270 | -0.3269 |
| C_4 | Gh_A11G1871 | slr1673 | Uncharacterized tRNA/rRNA methyltransferase slr1673 | -0.0349 | 0.9271 | -0.5101 | -1.6049 |
| C_4 | Gh_A11G1897 | BHLH25 | Transcription factor bHLH25 | -0.2368 | 0.5603 | -1.7588 | -1.1456 |
| C_4 | Gh_A11G1967 | NA | NA | 0.0195 | 0.9994 | 0.4371 | 0.2883 |
| C_4 | Gh_A11G1983 | TTC1 | Tetratricopeptide repeat protein 1 | -0.4798 | 0.8622 | -0.6106 | -0.2429 |
| C_4 | Gh_A11G2002 | At5g24010 | Probable receptor-like protein kinase | -0.2848 | 1.4129 | -0.9921 | -0.1455 |
| C_4 | Gh_A11G2238 | JMJ16 | Putative lysine-specific demethylase JMJ16 | -0.1289 | 0.2168 | 0.1813 | -0.0627 |
| C_4 | Gh_A11G2244 | CPRF2 | Light-inducible protein CPRF2 | -0.7160 | 0.5495 | -0.3815 | -0.0074 |
| C_4 | Gh_A11G2249 | psbW | Photosystem II reaction center W protein, chloroplastic | -0.1256 | 0.6977 | -0.8668 | -0.3103 |
| C_4 | Gh_A11G2358 | At4g27190 | Disease resistance protein | -0.6787 | -0.1536 | -0.3194 | -0.3919 |
| C_4 | Gh_A11G2423 | RECQSIM | ATP-dependent DNA helicase Q-like SIM | -0.0917 | 0.5226 | 0.1641 | -0.5540 |
| C_4 | Gh_A11G2488 | NA | NA | 0.7626 | 1.1403 | -2.0736 | -2.0925 |
| C_4 | Gh_A11G2492 | OSH6 | Homeobox protein knotted-1-like 1 | 1.8780 | 2.0219 | 0.4794 | 0.2731 |
| C_4 | Gh_A11G2551 | At4g27220 | Probable disease resistance protein | 0.0421 | 0.5947 | 0.7263 | -0.2119 |
| C_4 | Gh_A11G2701 | At5g16730 | WEB family protein At5g16730, chloroplastic | 0.4518 | 0.3880 | -0.3394 | -0.3570 |
| C_4 | Gh_A11G2721 | At5g36290 | GDT1-like protein 3 | -0.0257 | 0.0975 | -0.0113 | -0.1274 |
| C_4 | Gh_A11G2782 | PLT2 | AP2-like ethylene-responsive transcription factor PLT2 | 0.1000 | 1.4533 | -0.9873 | -0.4020 |
| C_4 | Gh_A11G2791 | GK-2 | Guanylate kinase 2 | -0.1602 | -0.2819 | -0.4994 | -0.4921 |
| C_4 | Gh_A11G2820 | RF178 | Probable E3 ubiquitin-protein ligase BAH1-like | 0.4095 | 0.0609 | -0.4663 | -1.8705 |
| C_4 | Gh_A11G2855 | NA | NA | -2.7640 | 0.3277 | -0.8845 | -1.8343 |
| C_4 | Gh_A11G2861 | NA | NA | 0.0883 | 0.1735 | -0.1171 | -0.3676 |
| C_4 | Gh_A11G2862 | NA | NA | 0.6730 | 0.5881 | 0.4397 | -0.6444 |
| C_4 | Gh_A11G2935 | At3g09070 | UPF0503 protein | 0.4587 | 0.8991 | -0.1121 | 0.1027 |
| C_4 | Gh_A11G2936 | IRX12 | Laccase-4 | 0.5226 | 0.7403 | -0.2364 | 0.1054 |
| C_4 | Gh_A11G3117 | TPPJ | Probable trehalose-phosphate phosphatase J | 1.1765 | 0.9148 | 0.2278 | -0.1427 |
| C_4 | Gh_A12G0039 | NA | NA | 0.5818 | 0.6264 | -0.0696 | -0.1022 |
| C_4 | Gh_A12G0052 | GID1B | Gibberellin receptor GID1B | -0.3002 | 0.3967 | -0.0032 | 0.0320 |
| C_4 | Gh_A12G0131 | UGT85A8 | UDP-glycosyltransferase 85A8 | -0.6106 | 1.3771 | -0.6972 | 0.0203 |
| C_4 | Gh_A12G0150 | MADS27 | MADS-box transcription factor 27 | 0.3961 | 1.9528 | 0.8610 | 1.2231 |
| C_4 | Gh_A12G0237 | NA | NA | -0.8044 | 0.6784 | -0.8333 | -0.7380 |
| C_4 | Gh_A12G0252 | rplI | 50S ribosomal protein L9 | 0.7539 | 1.9038 | 1.8684 | -2.0559 |
| C_4 | Gh_A12G0260 | At3g01520 | Universal stress protein A-like protein | -0.5898 | 1.3563 | 0.2454 | -0.1749 |
| C_4 | Gh_A12G0293 | UGT80B1 | Sterol 3-beta-glucosyltransferase | -1.1163 | 1.5154 | -0.6720 | 0.0118 |
| C_4 | Gh_A12G0358 | EIF4B3 | Eukaryotic translation initiation factor 4B3 | 0.2565 | 0.2987 | 0.4106 | 0.0426 |
| C_4 | Gh_A12G0470 | CTPS1 | CTP synthase 1 | 0.4913 | 1.8081 | 1.3908 | 1.2355 |
| C_4 | Gh_A12G0499 | ntpR | Protein NtpR | -0.3635 | 0.8991 | 0.1991 | 0.3464 |
| C_4 | Gh_A12G0540 | PLDDELTA | Phospholipase D delta | -0.0666 | 0.6718 | -0.1110 | 0.2449 |
| C_4 | Gh_A12G0638 | DOF5.7 | Dof zinc finger protein DOF5.7 | -0.1157 | 0.7029 | -0.7565 | -0.1662 |
| C_4 | Gh_A12G0673 | ERF008 | Ethylene-responsive transcription factor | 0.3114 | 0.5834 | 0.2592 | 0.0351 |
| C_4 | Gh_A12G0726 | HAT22 | Homeobox-leucine zipper protein HAT22 | -0.1541 | 0.3345 | -0.8590 | -1.0135 |
| C_4 | Gh_A12G0869 | CCD7 | Carotenoid cleavage dioxygenase 7, chloroplastic | -0.3440 | -0.6488 | -0.9295 | -1.3340 |
| C_4 | Gh_A12G0941 | SBTI1.1 | Subtilisin-like protease SBT1.1 | 0.0833 | -0.0233 | -1.2026 | -1.5661 |
| C_4 | Gh_A12G1008 | NA | NA | -0.2101 | 0.9062 | -1.0341 | -0.4782 |
| C_4 | Gh_A12G1116 | WRKY23 | Probable WRKY transcription factor 23 | -0.0598 | 2.3273 | -0.0925 | -0.1656 |
| C_4 | Gh_A12G1250 | NA | NA | 0.2700 | 2.3257 | 0.1780 | -1.0761 |
| C_4 | Gh_A12G1403 | VAC14 | Protein VAC14 homolog | -0.3545 | 0.6228 | -0.1282 | -0.0946 |
| C_4 | Gh_A12G1505 | NAC029 | NAC transcription factor 29 | -1.3152 | 1.2088 | -1.4076 | -0.6155 |
| C_4 | Gh_A12G1579 | PERK10 | Proline-rich receptor-like protein kinase PERK10 | 0.2988 | 0.5090 | 0.4986 | -0.0812 |
| C_4 | Gh_A12G1811 | PIRL9 | Plant intracellular Ras-group-related LRR protein 9 | 0.2995 | 0.5025 | 0.6084 | 0.2147 |
| C_4 | Gh_A12G1840 | NA | NA | -0.7145 | -0.0027 | -1.3487 | -0.8842 |
| C_4 | Gh_A12G1960 | HAT2 | Homeobox-leucine zipper protein HAT2 | 0.0530 | 0.6120 | -0.8179 | -0.4809 |
| C_4 | Gh_A12G2012 | NA | 21 kDa protein | 0.2713 | -0.2069 | -1.2927 | -2.0106 |
| C_4 | Gh_A12G2021 | NA | NA | 0.7448 | 0.6559 | 1.0594 | -0.0669 |
| C_4 | Gh_A12G2041 | At1g12500 | Probable sugar phosphate/phosphate translocator | 0.2948 | 1.2766 | 0.3910 | 0.2592 |
| C_4 | Gh_A12G2059 | STC | Sugar carrier protein C | 0.6043 | 2.1795 | -1.0775 | -1.7308 |
| C_4 | Gh_A12G2075 | NA | Remorin | 0.1562 | 0.9734 | 0.4448 | -0.4029 |
| C_4 | Gh_A12G2078 | NA | NA | -0.9162 | 0.8682 | -1.0929 | -0.1604 |
| C_4 | Gh_A12G2079 | At5g38830 | Cysteine--tRNA ligase 2, cytoplasmic | -0.0724 | 0.2012 | 0.2824 | -0.0815 |
| C_4 | Gh_A12G2169 | CYP78A3 | Cytochrome P450 78A3 | 0.6032 | 1.3782 | 0.4615 | -0.1348 |
| C_4 | Gh_A12G2289 | SGT1B | Protein SGT1 homolog B | -0.0964 | 0.6478 | -0.7511 | -0.1636 |
| C_4 | Gh_A12G2632 | NA | NA | -0.0347 | 0.7213 | 0.3661 | -0.0180 |
| C_4 | Gh_A13G0091 | NA | NA | 1.4730 | 0.5156 | -0.7288 | -2.7907 |
| C_4 | Gh_A13G0113 | CKX1 | Cytokinin dehydrogenase 1 | -0.3599 | 0.0460 | -0.2209 | -0.6656 |
| C_4 | Gh_A13G0145 | NA | NA | -0.2147 | 0.9539 | -0.8351 | -1.5900 |
| C_4 | Gh_A13G0215 | NA | Cytochrome P450 CYP736A12 | 0.1000 | 1.4533 | -0.5689 | -1.1805 |
| C_4 | Gh_A13G0216 | LAC17 | Laccase-17 | 0.1001 | 1.4533 | 0.9916 | 0.4847 |
| C_4 | Gh_A13G0288 | RD19A | Cysteine proteinase RD19a | -0.0367 | 0.4540 | -0.3557 | -0.1032 |
| C_4 | Gh_A13G0327 | PEX4 | Pollen-specific leucine-rich repeat extensin-like protein 4 | -0.4505 | 0.3723 | -1.3711 | -0.8896 |
| C_4 | Gh_A13G0342 | CXE12 | Probable carboxylesterase 12 | -1.0575 | 1.4919 | 0.0590 | 0.5714 |
| C_4 | Gh_A13G0408 | ISPH | 4-hydroxy-3-methylbut-2-enyl diphosphate reductase, | -0.2077 | 1.0862 | 0.4355 | 0.1390 |
|  |  |  | chloroplastic |  |  |  |  |
| C_4 | Gh_A13G0448 | NPF4.6 | Protein NRT1/ PTR FAMILY 4.6 | 0.1352 | 0.3829 | 0.0263 | -0.2701 |
| C_4 | Gh_A13G0500 | XTH9 | Xyloglucan endotransglucosylase/hydrolase protein 9 | 0.1247 | 0.4478 | -0.8797 | -0.6487 |
| C_4 | Gh_A13G0618 | NA | NA | -1.9084 | -0.1784 | -0.4178 | -2.1387 |
| C_4 | Gh_A13G0667 | At3g19950 | E3 ubiquitin-protein ligase RING1-like | -0.5857 | -0.0384 | -1.3500 | -1.1796 |
| C_4 | Gh_A13G0796 | At5g07610 | F-box protein At5g07610 | -1.8111 | 0.7429 | -0.4807 | -0.2229 |
| C_4 | Gh_A13G1038 | RH10 | DEAD-box ATP-dependent RNA helicase 10 | 0.1896 | 0.3911 | 0.4968 | -0.3838 |
| C_4 | Gh_A13G1059 | OCT3 | Organic cation/carnitine transporter 3 | -1.2062 | -0.4757 | -0.6484 | -1.2307 |
| C_4 | Gh_A13G1174 | NA | NA | -1.5337 | 1.6496 | 0.2293 | 0.5445 |
| C_4 | Gh_A13G1182 | At4g26340 | F-box/FBD/LRR-repeat protein | -0.1859 | 1.0050 | -0.1939 | -0.6555 |
| C_4 | Gh_A13G1209 | BGLU41 | Putative beta-glucosidase 41 | -0.7790 | 0.5991 | -0.3402 | -0.5913 |
| C_4 | Gh_A13G1326 | CODM | Codeine O-demethylase | 0.1874 | 0.3779 | 0.3996 | -0.4599 |
| C_4 | Gh_A13G1472 | NA | NA | 2.2843 | 2.9844 | 1.2781 | 0.3516 |
| C_4 | Gh_A13G1628 | At1g67520 | G-type lectin S-receptor-like serine/threonine-protein | 1.0075 | 2.2306 | 0.7215 | 1.3336 |
|  |  |  | kinase |  |  |  |  |
| C_4 | Gh_A13G1638 | IRX9H | Probable beta-1,4-xylosyltransferase IRX9H | 1.3680 | 3.7442 | 3.6064 | -0.2525 |
| C_4 | Gh_A13G2162 | NA | NA | 0.4038 | 1.3034 | -0.7771 | -1.0899 |
| C_4 | Gh_A13G2236 | ND1 | NADH-ubiquinone oxidoreductase chain 1 | -1.1097 | 1.9959 | 0.1773 | -0.0706 |
| C_4 | Gh_A13G2237 | NA | NA | 0.0171 | 1.0194 | 0.1610 | -0.3368 |
| C_4 | Gh_A13G2340 | AOP1 | Probable 2-oxoglutarate-dependent dioxygenase AOP1 | 2.0288 | 0.0313 | 0.6965 | -2.7070 |
| C_4 | Gh_D01G0014 | STY46 | Serine/threonine-protein kinase STY46 | -1.5147 | 1.1726 | 0.2305 | -0.1654 |
| C_4 | Gh_D01G0069 | CYPRO4 | Protein CYPRO4 | 0.1371 | 0.7376 | 0.0300 | -0.0400 |
| C_4 | Gh_D01G0157 | NA | NA | -0.0867 | 0.7662 | -0.3331 | -0.2815 |
| C_4 | Gh_D01G0164 | NA | NA | -0.4140 | 1.2148 | -0.4327 | 0.2368 |
| C_4 | Gh_D01G0275 | BRG3 | Probable BOI-related E3 ubiquitin-protein ligase 3 | 0.0837 | 0.4356 | -0.5002 | -0.4014 |
| C_4 | Gh_D01G0387 | KU70 | ATP-dependent DNA helicase 2 subunit KU70 | -0.1669 | 0.2804 | -0.7057 | -0.4842 |
| C_4 | Gh_D01G0422 | VCX1 | Vacuolar calcium ion transporter | -0.2634 | 1.7197 | 0.0351 | 0.3653 |
| C_4 | Gh_D01G0591 | At1g54290 | Protein translation factor SUI1 homolog 2 | -0.3254 | 0.0696 | -0.0684 | -1.1906 |
| C_4 | Gh_D01G0641 | NA | NA | -0.3128 | -0.0425 | -0.4953 | -0.4348 |
| C_4 | Gh_D01G0648 | At5g55050 | GDSL esterase/lipase | -0.1952 | 1.8075 | -0.0378 | 0.0754 |
| C_4 | Gh_D01G0655 | ANP2 | Mitogen-activated protein kinase kinase kinase 2 | 0.7461 | 1.4191 | 1.4986 | 0.9480 |
| C_4 | Gh_D01G0672 | EHD1 | EH domain-containing protein 1 | 0.2891 | 0.7497 | 0.6040 | 0.0619 |
| C_4 | Gh_D01G0829 | ZIP1 | Zinc transporter 1 | -0.1934 | -0.1613 | -0.1132 | -0.6791 |
| C_4 | Gh_D01G0867 | Tango2 | Transport and Golgi organization 2 homolog | 0.5010 | 0.9751 | 0.4444 | 0.0162 |
| C_4 | Gh_D01G0911 | PREP | Prolyl endopeptidase | -1.6528 | 1.5382 | -1.8475 | -0.3878 |
| C_4 | Gh_D01G0978 | NA | NA | -0.6979 | -0.0913 | -1.5159 | -1.6085 |
| C_4 | Gh_D01G1031 | nlt-1 | Non-specific lipid-transfer protein-like 1 | -1.1906 | 2.7688 | -1.2403 | -1.8797 |
| C_4 | Gh_D01G1081 | VPS35B | Vacuolar protein sorting-associated protein 35B | -0.9549 | 1.0745 | -0.5636 | -1.6950 |
| C_4 | Gh_D01G1128 | CCMFC | Cytochrome c biogenesis CcmF C-terminal-like | 0.7871 | 2.0612 | 0.3037 | -0.7849 |
|  |  |  | mitochondrial protein |  |  |  |  |
| C_4 | Gh_D01G1145 | PHF1 | SEC12-like protein 1 | 0.2106 | 0.2362 | 0.1471 | -0.1598 |
| C_4 | Gh_D01G1155 | UGT94E5 | Beta-D-glucosyl crocetin beta-1,6-glucosyltransferase | -0.1438 | 1.5642 | 0.5396 | 0.8094 |
| C_4 | Gh_D01G1180 | NA | NA | 0.5687 | 2.4622 | 0.3270 | -0.4240 |
| C_4 | Gh_D01G1187 | NA | NA | -0.0431 | 1.5441 | 0.9295 | 1.2184 |
| C_4 | Gh_D01G1196 | NA | NA | -0.4877 | -0.3874 | 0.7047 | -2.4742 |
| C_4 | Gh_D01G1218 | TBR | Protein trichome birefringence | -0.1850 | 0.6638 | -0.7624 | -0.4252 |
| C_4 | Gh_D01G1293 | ZIP1 | Zinc transporter 1 | -1.9502 | 1.0100 | -1.9755 | -1.5095 |
| C_4 | Gh_D01G1303 | POLL | DNA polymerase lambda | -0.9367 | -0.3118 | -1.4044 | -1.2629 |
| C_4 | Gh_D01G1325 | ALA3 | Phospholipid-transporting ATPase 3 | -0.0082 | 0.3222 | -0.0392 | -0.4858 |
| C_4 | Gh_D01G1468 | MPK17 | Mitogen-activated protein kinase 17 | -0.3892 | 0.9168 | -0.1709 | -0.1217 |
| C_4 | Gh_D01G1550 | lip | Lipase | -0.4844 | 1.2645 | -0.7904 | -0.0275 |
| C_4 | Gh_D01G1556 | XERICO | Probable E3 ubiquitin-protein ligase XERICO | -0.6342 | 1.4419 | -0.2691 | 0.5475 |
| C_4 | Gh_D01G1663 | STY46 | Serine/threonine-protein kinase STY46 | -1.2370 | 0.0869 | -0.4179 | -0.5648 |
| C_4 | Gh_D01G1695 | At4g35230 | Probable serine/threonine-protein kinase | -0.0856 | 0.9539 | 0.2950 | 0.5468 |
| C_4 | Gh_D01G1729 | ARPN | Basic blue protein | 0.1364 | 0.7070 | -0.7730 | -0.7559 |
| C_4 | Gh_D01G1763 | POT5 | Potassium transporter 5 | 0.5986 | -0.3482 | -1.3552 | -1.4405 |
| C_4 | Gh_D01G1778 | NA | NA | 0.4677 | 0.7802 | 0.4787 | 0.4524 |
| C_4 | Gh_D01G1813 | PUR2 | Phosphoribosylamine--glycine ligase, chloroplastic | 1.0507 | 0.1651 | -0.9662 | -0.8288 |
| C_4 | Gh_D01G1970 | MED27 | Mediator of RNA polymerase II transcription subunit 27 | -0.0469 | 0.2403 | -0.3141 | -0.8875 |
| C_4 | Gh_D01G2014 | ZPR4 | Protein LITTLE ZIPPER 4 | -0.5272 | 3.1138 | 1.2208 | -1.8870 |
| C_4 | Gh_D01G2041 | NA | NA | -0.4082 | 1.7316 | 0.7163 | 1.0322 |
| C_4 | Gh_D01G2159 | NA | NA | 0.3724 | 0.6140 | -0.5525 | -0.4403 |
| C_4 | Gh_D01G2168 | SPAC24B11.0 | Uncharacterized protein C24B11.05 | -0.2894 | 0.1939 | -0.3078 | -0.5375 |
|  |  | 5 |  |  |  |  |  |
| C_4 | Gh_D01G2338 | EP1 | Epidermis-specific secreted glycoprotein EP1 | -0.1983 | 0.3932 | 0.1100 | -0.0948 |
| C_4 | Gh_D02G0029 | At3g08650 | Putative zinc transporter | 0.1870 | 0.1511 | 0.2170 | -0.0053 |
| C_4 | Gh_D02G0030 | At1g06840 | Probable LRR receptor-like serine/threonine-protein | 0.5745 | 0.6652 | 0.3497 | -0.1931 |
|  |  |  | kinase |  |  |  |  |
| C_4 | Gh_D02G0031 | At3g53590 | Putative leucine-rich repeat receptor-like serine/threonine- | 0.3219 | 0.7580 | 0.3726 | 0.0444 |
|  |  |  | protein kinase |  |  |  |  |
| C_4 | Gh_D02G0143 | CRT3 | Calreticulin-3 | 0.0248 | 0.1623 | -0.3137 | -0.1301 |
| C_4 | Gh_D02G0196 | WAKL8 | Wall-associated receptor kinase-like 8 | 0.7516 | 0.5557 | 0.1134 | -0.5144 |
| C_4 | Gh_D02G0221 | NA | NA | -0.8617 | 1.4264 | -0.0564 | -1.6375 |
| C_4 | Gh_D02G0287 | NA | Cytochrome P450 CYP749A22 | -1.1247 | 0.4224 | -0.0253 | -0.3484 |
| C_4 | Gh_D02G0292 | NA | NA | 1.8125 | 2.1506 | -0.2229 | -0.2525 |
| C_4 | Gh_D02G0297 | NA | NA | -0.6207 | 0.5020 | -1.1845 | -0.7364 |
| C_4 | Gh_D02G0387 | HMT3 | Homocysteine S-methyltransferase 3 | -0.6012 | 1.7392 | -0.1023 | 0.3500 |
| C_4 | Gh_D02G0497 | At5g18840 | Sugar transporter ERD6-like 16 | -1.7169 | 0.3664 | -1.6636 | -0.8889 |
| C_4 | Gh_D02G0561 | NA | NA | 1.7410 | 5.6687 | -2.4145 | -4.5326 |
| C_4 | Gh_D02G0563 | NA | NA | -2.3363 | 6.6790 | -3.8213 | -2.1711 |
| C_4 | Gh_D02G0564 | NA | NA | -1.2406 | 7.2031 | -1.4067 | -1.3303 |
| C_4 | Gh_D02G0568 | NA | NA | 1.7707 | 7.4008 | -3.1075 | -3.0902 |
| C_4 | Gh_D02G0613 | MYB108 | Transcription factor MYB108 | 0.2579 | 1.1497 | -0.7296 | -0.0959 |
| C_4 | Gh_D02G0712 | CYP707A3 | Abscisic acid 8'-hydroxylase 3 | -1.0026 | 1.0335 | -1.2945 | -2.1934 |
| C_4 | Gh_D02G0911 | NA | NA | -0.0978 | 3.0382 | -1.3584 | -0.6631 |
| C_4 | Gh_D02G0943 | NA | NA | -2.3143 | 4.4143 | -1.0052 | -1.7080 |
| C_4 | Gh_D02G0982 | SYT2 | Synaptotagmin-2 | 0.3234 | 0.9026 | 0.4672 | 0.0766 |
| C_4 | Gh_D02G1166 | HT1 | Serine/threonine-protein kinase HT1 | -0.1579 | 0.5651 | 0.1969 | 0.1797 |
| C_4 | Gh_D02G1255 | SBH2 | Sphinganine C4-monooxygenase 2 | -0.0451 | 0.7319 | 0.4195 | -0.1875 |
| C_4 | Gh_D02G1265 | APL | Myb family transcription factor APL | 0.1157 | 0.2606 | -0.4165 | -0.7034 |
| C_4 | Gh_D02G1276 | NA | NA | -0.0026 | 1.3184 | 0.3627 | 0.5089 |
| C_4 | Gh_D02G1314 | GEK1 | D-aminoacyl-tRNA deacylase | 0.1041 | 0.8893 | 0.4709 | 0.6023 |
| C_4 | Gh_D02G1483 | NA | NA | -0.3849 | 0.8223 | 0.1892 | -0.0355 |
| C_4 | Gh_D02G1534 | PUB6 | U-box domain-containing protein 6 | -0.0426 | 0.2572 | -0.5328 | -0.9897 |
| C_4 | Gh_D02G1572 | RIN4 | RPM1-interacting protein 4 | 0.0341 | 1.9413 | 0.5792 | -0.1410 |
| C_4 | Gh_D02G1727 | HMT1 | Homocysteine S-methyltransferase 1 | 0.3050 | 0.5094 | 0.2540 | -0.9450 |
| C_4 | Gh_D02G1736 | CSE | Caffeoylshikimate esterase | -0.3716 | 0.5879 | 0.2837 | 0.2489 |
| C_4 | Gh_D02G1780 | NA | NA | -1.3688 | 1.0356 | -0.4781 | 0.0325 |
| C_4 | Gh_D02G1829 | TPC1 | Two pore calcium channel protein 1 | -0.3833 | 1.2438 | -0.3651 | -0.0283 |
| C_4 | Gh_D02G1838 | NA | NA | -0.1463 | 1.0862 | 0.0098 | 0.4736 |
| C_4 | Gh_D02G1845 | STA1 | Protein STABILIZED1 | 0.3545 | 0.0765 | -0.1953 | -0.2005 |
| C_4 | Gh_D02G1977 | FDM1 | Factor of DNA methylation 1 | -1.0494 | 2.1479 | 0.0061 | -0.4379 |
| C_4 | Gh_D02G2124 | At4g06598 | Uncharacterized protein | 0.3029 | 0.7699 | 0.0763 | 0.0697 |
| C_4 | Gh_D02G2362 | CAT1 | Cationic amino acid transporter 1 | -0.5314 | 0.3884 | -0.8400 | -0.4061 |
| C_4 | Gh_D02G2437 | At2g41420 | Cysteine-rich and transmembrane domain-containing | 0.1627 | 1.3187 | 0.5094 | -0.1324 |
|  |  |  | protein A |  |  |  |  |
| C_4 | Gh_D02G2441 | CXE13 | Probable carboxylesterase 13 | 0.3855 | 0.3745 | -1.8726 | -1.1392 |
| C_4 | Gh_D02G2447 | At1g71900 | Probable magnesium transporter NIPA4 | -0.3652 | 0.5244 | 0.3323 | 0.1783 |
| C_4 | Gh_D03G0082 | NFD4 | Protein NUCLEAR FUSION DEFECTIVE 4 | 0.3885 | 1.2223 | 1.0720 | 0.4874 |
| C_4 | Gh_D03G0122 | At2g18193 | AAA-ATPase | 0.4799 | 0.0969 | -1.7214 | -1.2495 |
| C_4 | Gh_D03G0258 | APK1 | Adenylyl-sulfate kinase 1, chloroplastic | -1.0084 | 0.6553 | -0.4708 | -0.0765 |
| C_4 | Gh_D03G0343 | TPPJ | Probable trehalose-phosphate phosphatase J | 0.5641 | 1.4346 | 0.9835 | 0.0698 |
| C_4 | Gh_D03G0389 | PFK5 | ATP-dependent 6-phosphofructokinase 5, chloroplastic | 0.2300 | 0.3307 | 0.3156 | -0.0386 |
| C_4 | Gh_D03G0450 | ABR1 | Ethylene-responsive transcription factor ABR1 | -1.2401 | 1.6474 | 0.7326 | 0.4145 |
| C_4 | Gh_D03G0470 | NA | NA | 1.1708 | 1.3362 | -0.4068 | -0.3838 |
| C_4 | Gh_D03G0560 | LYK3 | LysM domain receptor-like kinase 3 | -0.0997 | 0.1975 | -0.1867 | -0.0529 |
| C_4 | Gh_D03G0678 | ACS1 | 1-aminocyclopropane-1-carboxylate synthase | 0.0320 | 1.5272 | -0.0355 | 0.0314 |
| C_4 | Gh_D03G0728 | NET1A | Protein NETWORKED 1A | -0.5596 | 0.5738 | -0.0379 | 0.2943 |
| C_4 | Gh_D03G0756 | NA | NA | 0.4912 | 2.0551 | -0.1571 | -0.1219 |
| C_4 | Gh_D03G0810 | At4g31240 | Probable nucleoredoxin 3 | -0.2283 | 0.9215 | -0.7704 | -0.1947 |
| C_4 | Gh_D03G0897 | Zranb3 | DNA annealing helicase and endonuclease ZRANB3 | -0.7663 | 0.1274 | -0.0984 | -0.3457 |
| C_4 | Gh_D03G1031 | NA | 21 kDa protein | 1.4837 | 1.9948 | 1.9901 | 0.6135 |
| C_4 | Gh_D03G1153 | At3g01520 | Universal stress protein A-like protein | 0.4143 | 0.5641 | -0.4133 | -0.6195 |
| C_4 | Gh_D03G1484 | DTX49 | Protein DETOXIFICATION 49 | 0.0578 | 0.7993 | -0.3924 | -0.0159 |
| C_4 | Gh_D03G1502 | slr0305 | TVP38/TMEM64 family membrane protein slr0305 | -0.1170 | 0.8674 | 0.0701 | -0.0444 |
| C_4 | Gh_D03G1504 | CYP75A2 | Flavonoid 3',5'-hydroxylase | -0.2015 | -0.2258 | 0.1915 | -1.3032 |
| C_4 | Gh_D03G1517 | NA | NA | -1.9709 | 2.0923 | -0.8742 | -0.7930 |
| C_4 | Gh_D03G1623 | NPF7.3 | Protein NRT1/ PTR FAMILY 7.3 | 0.3106 | 0.5147 | 0.5038 | -0.6711 |
| C_4 | Gh_D03G1635 | NA | NA | -0.8027 | 0.3905 | -1.7430 | -1.0953 |
| C_4 | Gh_D03G1668 | RPN9B | 26S proteasome non-ATPase regulatory subunit 13 | -0.1662 | 0.2459 | -0.2179 | -0.2092 |
|  |  |  | homolog B |  |  |  |  |
| C_4 | Gh_D03G1698 | NA | NA | 0.1674 | 0.5571 | -0.4396 | -0.4821 |
| C_4 | Gh_D03G1766 | FBPban1 | Fructose-1,6-bisphosphatase, cytosolic | -2.6967 | 1.3888 | 0.0699 | -0.2384 |
| C_4 | Gh_D03G1849 | MYC4 | Transcription factor MYC4 | -0.9422 | 0.2802 | -1.5375 | -0.7234 |
| C_4 | Gh_D03G1857 | At3g02910 | Putative gamma-glutamylcyclotransferase | -1.1788 | -0.1202 | -1.8679 | -1.4026 |
| C_4 | Gh_D04G0152 | PCMP-H8 | Pentatricopeptide repeat-containing protein | 0.1534 | 1.7106 | -1.2462 | -2.1034 |
| C_4 | Gh_D04G0388 | UBP20 | Ubiquitin carboxyl-terminal hydrolase 20 | -0.4329 | -0.0723 | -0.3850 | -0.8919 |
| C_4 | Gh_D04G0418 | WRKY3 | Probable WRKY transcription factor 3 | 0.1102 | 0.3373 | -0.2045 | -0.1514 |
| C_4 | Gh_D04G0419 | HYP1 | CSC1-like protein HYP1 | -0.7433 | -0.0766 | -0.6112 | -0.3593 |
| C_4 | Gh_D04G0481 | P4H7 | Probable prolyl 4-hydroxylase 7 | 0.2291 | 0.9010 | 0.2728 | 0.3348 |
| C_4 | Gh_D04G0572 | At3g12360 | Ankyrin repeat-containing protein | 0.2513 | 0.0226 | -0.6546 | -0.5330 |
| C_4 | Gh_D04G0655 | ANS | Leucoanthocyanidin dioxygenase | -0.3929 | 1.7949 | -0.0254 | 0.4097 |
| C_4 | Gh_D04G0719 | ACR6 | ACT domain-containing protein ACR6 | -0.4069 | 0.5502 | 0.9118 | -0.3775 |
| C_4 | Gh_D04G0911 | ACT | Vinorine synthase | -0.3750 | 1.8989 | -0.6130 | -0.3117 |
| C_4 | Gh_D04G1055 | MYBAS1 | Myb-related protein MYBAS1 | -0.8746 | 2.7747 | -0.7703 | -0.5513 |
| C_4 | Gh_D04G1202 | AKR4C9 | Aldo-keto reductase family 4 member C9 | -0.2105 | 0.8074 | -0.0075 | -0.3060 |
| C_4 | Gh_D04G1243 | IRX12 | Laccase-4 | -0.6938 | -0.0362 | -0.8307 | -0.6798 |
| C_4 | Gh_D04G1248 | RPN8B | 26S proteasome non-ATPase regulatory subunit 7 | -0.9334 | 1.1270 | -0.3553 | -2.9163 |
|  |  |  | homolog B |  |  |  |  |
| C_4 | Gh_D04G1252 | LAX2 | Auxin transporter-like protein 2 | 0.4109 | 0.1424 | 0.0909 | -0.5675 |
| C_4 | Gh_D04G1347 | ARR4 | Two-component response regulator ARR4 | 1.3543 | 1.3990 | 0.1517 | -0.0854 |
| C_4 | Gh_D04G1523 | RIBA1 | Bifunctional riboflavin biosynthesis protein RIBA 1, | -0.7544 | 0.8237 | -0.3938 | -0.0796 |
|  |  |  | chloroplastic |  |  |  |  |
| C_4 | Gh_D04G1561 | NA | NA | 0.0362 | 0.6371 | -0.3244 | -0.7446 |
| C_4 | Gh_D04G1632 | NA | Arginine decarboxylase | -0.9430 | 0.7614 | -0.8341 | -0.5066 |
| C_4 | Gh_D04G1659 | Dcaf8 | DDB1- and CUL4-associated factor 8 | -0.3761 | 0.8711 | -0.3314 | 0.2559 |
| C_4 | Gh_D04G1670 | EXLA1 | Expansin-like A1 | -0.0335 | 1.7261 | -0.9842 | -0.2382 |
| C_4 | Gh_D04G1834 | ANK1 | Ankyrin-1 | 3.9549 | 2.8974 | 3.2549 | 0.9658 |
| C_4 | Gh_D04G1994 | ATPA | ATP synthase subunit alpha, mitochondrial | 0.1066 | 1.3889 | 1.5829 | 0.0099 |
| C_4 | Gh_D04G1995 | NA | NA | 0.5569 | 1.2507 | 0.2201 | -0.4494 |
| C_4 | Gh_D04G1998 | ND1 | NADH-ubiquinone oxidoreductase chain 1 | -0.1576 | 1.6260 | 0.6825 | -0.2504 |
| C_4 | Gh_D04G1999 | NA | NA | 0.0443 | 1.6771 | 0.7834 | 0.1812 |
| C_4 | Gh_D05G0067 | NA | NA | -0.1610 | -0.0766 | 0.3347 | -1.0689 |
| C_4 | Gh_D05G0148 | EBF1 | EIN3-binding F-box protein 1 | 0.2102 | 0.4129 | -0.0322 | -0.1873 |
| C_4 | Gh_D05G0267 | NA | NA | 0.0005 | 0.9077 | 0.5419 | 0.5353 |
| C_4 | Gh_D05G0324 | CFIS1 | Pre-mRNA cleavage factor Im 25 kDa subunit 1 | -0.5803 | 0.6048 | -0.0077 | 0.3742 |
| C_4 | Gh_D05G0356 | NA | 21 kDa protein | 0.5748 | 0.5526 | -0.6187 | -0.6405 |
| C_4 | Gh_D05G0393 | MYB5 | Transcription repressor MYB5 | -0.2315 | 0.1544 | -0.7774 | -1.5105 |
| C_4 | Gh_D05G0432 | ZIP4 | Zinc transporter 4, chloroplastic | 0.1194 | 0.5486 | -1.0095 | -0.9919 |
| C_4 | Gh_D05G0436 | EDR1 | Serine/threonine-protein kinase EDR1 | 0.0254 | -0.1060 | -0.3735 | -0.6114 |
| C_4 | Gh_D05G0479 | ATHB-8 | Homeobox-leucine zipper protein ATHB-8 | 0.6353 | 1.0097 | 0.1830 | -0.0619 |
| C_4 | Gh_D05G0485 | FBXL20 | F-box/LRR-repeat protein 20 | 0.1525 | 1.4568 | 1.0897 | 0.7312 |
| C_4 | Gh_D05G0570 | APT5 | Adenine phosphoribosyltransferase 5 | 0.1873 | 1.9650 | 0.9574 | 1.0318 |
| C_4 | Gh_D05G0580 | At1g80640 | Probable receptor-like protein kinase | -0.2550 | 0.7834 | 0.0162 | -0.1485 |
| C_4 | Gh_D05G0690 | UCNL | Serine/threonine-protein kinase UCNL | 0.2327 | -0.1220 | -0.8050 | -0.6825 |
| C_4 | Gh_D05G0705 | NADK1 | NAD(H) kinase 1 | 0.1659 | 0.5637 | 0.5439 | -0.2829 |
| C_4 | Gh_D05G0815 | At2g27500 | Glucan endo-1,3-beta-glucosidase 14 | -0.4152 | 1.0395 | 0.1810 | 0.3965 |
| C_4 | Gh_D05G0816 | FD3 | Ferredoxin-3, chloroplastic | 0.3709 | 0.6502 | 0.4956 | 0.2899 |
| C_4 | Gh_D05G1001 | At5g60760 | P-loop NTPase domain-containing protein LPA1 homolog | 0.9082 | 1.7921 | 1.1783 | 0.9090 |
|  |  |  | 1 |  |  |  |  |
| C_4 | Gh_D05G1025 | LSH6 | Protein LIGHT-DEPENDENT SHORT HYPOCOTYLS 6 | -0.4435 | 0.2797 | -0.7547 | -0.6265 |
| C_4 | Gh_D05G1036 | SDR2a | Short-chain dehydrogenase reductase 2a | -0.2350 | -1.4219 | -2.2160 | -2.4284 |
| C_4 | Gh_D05G1049 | AAE14 | 2-succinylbenzoate--CoA ligase, chloroplastic/peroxisomal | -0.2743 | 1.5565 | 0.7510 | 0.2362 |
| C_4 | Gh_D05G1155 | BRAP | BRCA1-associated protein | -0.0335 | 0.6561 | -0.0825 | -0.1946 |
| C_4 | Gh_D05G1220 | mshA | D-inositol 3-phosphate glycosyltransferase | -0.4906 | 0.3603 | -0.4924 | -0.2187 |
| C_4 | Gh_D05G1238 | NA | NA | -0.3188 | 3.3207 | 1.0580 | -0.5926 |
| C_4 | Gh_D05G1343 | MYB23 | Transcription factor MYB23 | -1.5099 | 0.6286 | -0.8344 | -0.3022 |
| C_4 | Gh_D05G1353 | At3g14260 | Protein LURP-one-related 11 | -1.2018 | 2.8412 | -0.7424 | -0.2235 |
| C_4 | Gh_D05G1454 | UTR4 | UDP-galactose/UDP-glucose transporter 4 | -0.8927 | 7.2469 | -2.7960 | -1.6169 |
| C_4 | Gh_D05G1537 | NA | NA | -0.4929 | 1.6504 | -0.6362 | -0.6148 |
| C_4 | Gh_D05G1600 | CXXS1 | Thioredoxin-like protein CXXS1 | -0.5556 | 1.3139 | -0.7791 | -0.5102 |
| C_4 | Gh_D05G1651 | CSC1 | Calcium permeable stress-gated cation channel 1 | 0.4678 | 2.2973 | 0.6684 | 0.6290 |
| C_4 | Gh_D05G1652 | At1g04910 | Uncharacterized protein | 0.2121 | 1.0043 | 0.5915 | -0.0291 |
| C_4 | Gh_D05G1661 | NA | Tropinone reductase-like 3 | 0.0822 | 0.7328 | 0.2663 | -0.0778 |
| C_4 | Gh_D05G1662 | AOX4 | Ubiquinol oxidase 4, chloroplastic/chromoplastic | 0.3461 | 0.7792 | -0.0522 | 0.1523 |
| C_4 | Gh_D05G1709 | NLP4 | Protein NLP4 | 0.2979 | 1.0462 | 0.2310 | 0.1928 |
| C_4 | Gh_D05G1711 | At2g30600/At | BTB/POZ domain-containing protein | -0.3828 | 0.2608 | -0.8247 | -0.6493 |
|  |  | 2g30610 |  |  |  |  |  |
| C_4 | Gh_D05G1729 | COR47 | Dehydrin COR47 | 0.8647 | 1.4361 | 0.7813 | 0.3165 |
| C_4 | Gh_D05G1800 | BBX21 | B-box zinc finger protein 21 | 0.2775 | 0.2280 | -0.3869 | -1.1531 |
| C_4 | Gh_D05G1860 | IRE4 | Probable serine/threonine protein kinase IRE4 | 0.2056 | 0.5734 | 0.0496 | 0.2714 |
| C_4 | Gh_D05G1878 | GA17800 | Leishmanolysin-like peptidase | 0.0390 | 0.5233 | 0.1423 | -0.6617 |
| C_4 | Gh_D05G1923 | NA | NA | -0.3119 | 0.2250 | 0.3193 | -0.3029 |
| C_4 | Gh_D05G1944 | trc | Serine/threonine-protein kinase tricorner | -0.0345 | 1.8331 | 0.0131 | -0.2205 |
| C_4 | Gh_D05G1963 | RAP2-1 | Ethylene-responsive transcription factor RAP2-1 | -0.3874 | 1.4504 | -0.2626 | -0.5011 |
| C_4 | Gh_D05G2011 | ZAT10 | Zinc finger protein ZAT10 | -0.1251 | 0.9577 | 0.3495 | 0.5307 |
| C_4 | Gh_D05G2015 | IFRD1 | Interferon-related developmental regulator 1 | -0.1416 | 0.6626 | 0.0625 | -0.0409 |
| C_4 | Gh_D05G2072 | IKU2 | Receptor-like protein kinase HAIKU2 | -0.4099 | -0.1930 | -0.8995 | -1.0573 |
| C_4 | Gh_D05G2083 | NA | NA | -0.1095 | 0.9415 | 0.3614 | 0.6419 |
| C_4 | Gh_D05G2148 | ATHB-6 | Homeobox-leucine zipper protein ATHB-6 | -0.5533 | 1.3406 | -0.0267 | 0.7488 |
| C_4 | Gh_D05G2168 | TFT7 | 14-3-3 protein 7 | -0.1760 | 0.0315 | -0.3156 | -0.1652 |
| C_4 | Gh_D05G2188 | RAP2-4 | Ethylene-responsive transcription factor RAP2-4 | 0.2644 | 0.9273 | 0.0427 | 0.1491 |
| C_4 | Gh_D05G2199 | ST2 | High affinity sulfate transporter 2 | -0.8577 | -1.6174 | -3.0572 | -3.5296 |
| C_4 | Gh_D05G2215 | SOV | DIS3-like exonuclease 2 | -0.1359 | 0.6900 | 0.1720 | 0.1019 |
| C_4 | Gh_D05G2289 | ARPN | Basic blue protein | 1.1084 | 0.8249 | 0.9715 | -0.1237 |
| C_4 | Gh_D05G2297 | GLTP3 | Glycolipid transfer protein 3 | -0.2047 | 0.7837 | -1.3045 | -0.4435 |
| C_4 | Gh_D05G2340 | CK1 | Probable choline kinase 1 | -0.0344 | 1.2611 | 0.5140 | 0.7965 |
| C_4 | Gh_D05G2425 | SMT2 | 24-methylenesterol C-methyltransferase 2 | 0.3691 | 0.3690 | 0.3764 | 0.3405 |
| C_4 | Gh_D05G2450 | Os09g0520200 | Probable 1-acylglycerol-3-phosphate O-acyltransferase | 0.2100 | 0.6073 | 0.1271 | 0.1670 |
| C_4 | Gh_D05G2459 | At3g50940 | AAA-ATPase | 0.6853 | 0.6462 | -0.6719 | -0.8315 |
| C_4 | Gh_D05G2514 | ACS8 | 1-aminocyclopropane-1-carboxylate synthase 8 | 0.5085 | 1.2639 | 0.3816 | -0.9715 |
| C_4 | Gh_D05G2578 | fam188a | Protein FAM188A | -0.1603 | 0.5260 | -0.1878 | 0.1063 |
| C_4 | Gh_D05G2583 | CK1 | Probable choline kinase 1 | -0.5719 | 0.1207 | -0.3441 | -0.2190 |
| C_4 | Gh_D05G2807 | AVT1 | Vacuolar amino acid transporter 1 | -0.1259 | -0.1414 | 0.0568 | -0.4369 |
| C_4 | Gh_D05G2852 | RPPL1 | Putative disease resistance RPP13-like protein 1 | 0.4526 | 2.3634 | -2.1067 | -1.5089 |
| C_4 | Gh_D05G2920 | PYL4 | Abscisic acid receptor PYL4 | -1.2834 | 1.2218 | -0.9348 | -0.4503 |
| C_4 | Gh_D05G2998 | NAD-ME2 | NAD-dependent malic enzyme 2, mitochondrial | -1.2224 | 1.4936 | -0.2959 | -1.6165 |
| C_4 | Gh_D05G3110 | ALA3 | Phospholipid-transporting ATPase 3 | 0.6910 | 2.1689 | 0.9982 | -0.1265 |
| C_4 | Gh_D05G3130 | NA | NA | -0.1734 | 3.2649 | -0.1847 | 0.0013 |
| C_4 | Gh_D05G3133 | NA | NA | 0.4261 | 2.8099 | -0.1482 | -0.3211 |
| C_4 | Gh_D05G3134 | NA | NA | 0.5713 | 3.2789 | 0.3192 | -0.2757 |
| C_4 | Gh_D05G3136 | NA | NA | 0.0931 | 2.9151 | -0.2272 | -0.0109 |
| C_4 | Gh_D05G3371 | At4g27190 | Disease resistance protein | -1.1568 | 1.0818 | -0.1348 | -0.2769 |
| C_4 | Gh_D05G3486 | MRE11 | Double-strand break repair protein MRE11 | 0.0669 | 0.9940 | -0.8267 | -0.3239 |
| C_4 | Gh_D05G3492 | BBX32 | B-box zinc finger protein 32 | 0.5304 | 0.5679 | -0.2029 | -0.8810 |
| C_4 | Gh_D05G3499 | B120 | G-type lectin S-receptor-like serine/threonine-protein | 0.0120 | 0.0062 | 0.0695 | -0.1430 |
|  |  |  | kinase B120 |  |  |  |  |
| C_4 | Gh_D05G3517 | UBQ10 | Polyubiquitin 10 | 0.1035 | 0.6177 | -0.1406 | -0.4366 |
| C_4 | Gh_D05G3694 | RIN4 | RPM1-interacting protein 4 | 0.1643 | 0.6984 | 0.1912 | 0.1125 |
| C_4 | Gh_D05G3804 | At1g36730 | Probable eukaryotic translation initiation factor 5-1 | 0.2518 | 0.4210 | -0.6185 | -0.2832 |
| C_4 | Gh_D05G3819 | MUB6 | Membrane-anchored ubiquitin-fold protein 6 | 0.2633 | 1.3068 | 1.0692 | -0.6396 |
| C_4 | Gh_D05G3838 | TCP2 | Transcription factor TCP2 | -0.2312 | 0.4340 | -0.3208 | -0.1425 |
| C_4 | Gh_D06G0099 | CYP82A3 | Cytochrome P450 82A3 | -0.7287 | -0.3964 | -1.8628 | -1.3256 |
| C_4 | Gh_D06G0143 | WEB1 | Protein WEAK CHLOROPLAST MOVEMENT UNDER | -0.4702 | 0.0905 | -3.8148 | -2.9481 |
|  |  |  | BLUE LIGHT 1 |  |  |  |  |
| C_4 | Gh_D06G0164 | SBT1.7 | Subtilisin-like protease SBT1.7 | 0.4624 | 0.5157 | 1.0687 | -0.2966 |
| C_4 | Gh_D06G0168 | ERF008 | Ethylene-responsive transcription factor ERF008 | -0.4118 | -0.1493 | -1.5284 | -1.1239 |
| C_4 | Gh_D06G0459 | CTR1 | Serine/threonine-protein kinase CTR1 | -0.0452 | 0.8375 | 0.5227 | 0.0832 |
| C_4 | Gh_D06G0478 | NA | NA | -1.3335 | 2.9801 | 0.4200 | -0.4769 |
| C_4 | Gh_D06G0517 | NA | NA | 0.4033 | 1.7777 | 0.4046 | -0.2695 |
| C_4 | Gh_D06G0518 | ATPA | ATP synthase subunit alpha, mitochondrial | 0.1040 | 2.0875 | 0.8157 | -0.5275 |
| C_4 | Gh_D06G0611 | COV1 | Protein CONTINUOUS VASCULAR RING 1 | -0.1367 | 0.7993 | 0.2471 | 0.5640 |
| C_4 | Gh_D06G0620 | NA | NA | -0.1688 | 0.7671 | 1.0491 | -1.1171 |
| C_4 | Gh_D06G0649 | SKIP | SNW/SKI-interacting protein | 0.2211 | 0.3017 | 0.0437 | -0.1397 |
| C_4 | Gh_D06G0684 | PFK3 | ATP-dependent 6-phosphofructokinase 3 | 0.0904 | 0.5689 | 0.5908 | 0.0249 |
| C_4 | Gh_D06G0931 | NA | NA | -0.2002 | 3.1765 | -0.3691 | -0.5862 |
| C_4 | Gh_D06G1061 | TPR1 | Topless-related protein 1 | -0.0337 | 0.7477 | -0.7304 | -0.1285 |
| C_4 | Gh_D06G1093 | MGD1 | Monogalactosyldiacylglycerol synthase 1, chloroplastic | -0.2823 | 0.5756 | 0.6747 | -0.4264 |
| C_4 | Gh_D06G1229 | SS4 | Probable starch synthase 4, chloroplastic/amyloplastic | 1.0618 | 2.3887 | -0.3095 | -0.2459 |
| C_4 | Gh_D06G1285 | KOR | Endoglucanase 25 | 0.3237 | 0.1858 | 0.1735 | 0.0212 |
| C_4 | Gh_D06G1347 | NA | NA | 0.0841 | 1.0713 | 0.5081 | 0.7166 |
| C_4 | Gh_D06G1452 | NA | NA | -0.2301 | 0.0288 | -0.7682 | -0.6944 |
| C_4 | Gh_D06G1479 | SH3P3 | SH3 domain-containing protein 3 | 0.0866 | 0.5223 | 0.1980 | 0.3118 |
| C_4 | Gh_D06G1513 | AERO1 | Endoplasmic reticulum oxidoreductin-1 | -0.4836 | 1.0032 | 0.1708 | 0.5205 |
| C_4 | Gh_D06G1560 | NA | NA | 1.3087 | 1.1514 | -0.8224 | -1.0754 |
| C_4 | Gh_D06G1656 | NA | NA | 0.2131 | 1.1016 | 0.1394 | 0.1868 |
| C_4 | Gh_D06G1668 | ACR6 | ACT domain-containing protein ACR6 | -2.7144 | 1.0645 | -0.1802 | -0.1274 |
| C_4 | Gh_D06G1698 | NA | Cysteine proteinase inhibitor 1 | 0.3219 | 1.5373 | 0.5339 | 1.0740 |
| C_4 | Gh_D06G1705 | RNP1 | Heterogeneous nuclear ribonucleoprotein 1 | 0.2035 | 0.2693 | 0.3199 | 0.1809 |
| C_4 | Gh_D06G1874 | At2g19280 | Pentatricopeptide repeat-containing protein | -0.4166 | 0.6913 | -0.1668 | -0.1889 |
| C_4 | Gh_D06G2135 | HSFA4A | Heat stress transcription factor A-4a | 0.3881 | 0.3078 | -0.0382 | -0.2767 |
| C_4 | Gh_D06G2316 | BHLH93 | Transcription factor bHLH93 | -0.4623 | 0.9208 | -0.3328 | -0.0036 |
| C_4 | Gh_D06G2353 | FLS2 | LRR receptor-like serine/threonine-protein kinase FLS2 | 0.4245 | 0.5561 | 1.0838 | -0.6578 |
| C_4 | Gh_D06G2356 | PLC2 | Phosphoinositide phospholipase C 2 | -0.2566 | 1.3485 | -0.4119 | 0.0862 |
| C_4 | Gh_D06G2369 | NA | NA | 0.0710 | 0.6315 | -0.4794 | -0.3449 |
| C_4 | Gh_D06G2392 | AMC9 | Metacaspase-9 | -1.5603 | -0.1354 | -1.0879 | -1.5691 |
| C_4 | Gh_D07G0076 | At4g26100 | Casein kinase I isoform delta-like | 0.1415 | 1.1751 | -0.6300 | -0.4834 |
| C_4 | Gh_D07G0092 | NA | NA | -0.3955 | 1.2098 | -0.5831 | -0.4130 |
| C_4 | Gh_D07G0093 | NA | NA | -3.0459 | 1.8123 | 0.8131 | -1.3798 |
| C_4 | Gh_D07G0120 | TBL16 | Protein trichome birefringence-like 16 | 0.1633 | 0.6187 | 0.2800 | 0.2490 |
| C_4 | Gh_D07G0192 | RTE1 | Protein REVERSION-TO-ETHYLENE SENSITIVITY1 | -0.3680 | -0.2437 | -0.4890 | -0.6218 |
| C_4 | Gh_D07G0197 | NA | NA | -0.9619 | 1.1599 | -0.0421 | -0.4720 |
| C_4 | Gh_D07G0291 | NA | NA | -0.1495 | 0.4680 | -0.3635 | -0.1394 |
| C_4 | Gh_D07G0440 | ERF106 | Ethylene-responsive transcription factor ERF106 | 0.2906 | 1.2391 | -1.0967 | -0.9773 |
| C_4 | Gh_D07G0442 | ERF2 | Ethylene-responsive transcription factor 2 | -0.1601 | 0.4851 | -1.0225 | -0.4597 |
| C_4 | Gh_D07G0541 | HAG3 | Elongator complex protein 3 | 0.8997 | 0.3669 | -1.2492 | -5.3627 |
| C_4 | Gh_D07G0552 | NA | Peptide methionine sulfoxide reductase | -0.2229 | -0.7283 | -1.2455 | -1.6319 |
| C_4 | Gh_D07G0560 | AFC2 | Serine/threonine-protein kinase AFC2 | -0.1655 | -0.0097 | -0.2080 | -0.4928 |
| C_4 | Gh_D07G0711 | NA | NA | 0.1984 | 0.6329 | -0.2229 | -0.2525 |
| C_4 | Gh_D07G0713 | CDCA7L | Cell division cycle-associated 7-like protein | -0.3412 | 0.4973 | -0.2184 | -0.4360 |
| C_4 | Gh_D07G0738 | POT1B | Protection of telomeres protein 1b | -0.1103 | -0.2776 | -0.7387 | -0.7031 |
| C_4 | Gh_D07G0913 | GAUT8 | Galacturonosyltransferase 8 | 0.1061 | 0.7531 | 0.4701 | 0.6157 |
| C_4 | Gh_D07G0970 | CXIP4 | CAX-interacting protein 4 | 0.5467 | 0.6053 | 0.2015 | -0.2390 |
| C_4 | Gh_D07G0995 | UGT76A2 | UDP-glucose iridoid glucosyltransferase | -2.2745 | 0.1944 | -0.7093 | -1.1059 |
| C_4 | Gh_D07G1037 | NA | NA | -0.9849 | 1.4394 | -0.0835 | -1.2224 |
| C_4 | Gh_D07G1072 | SAP3 | Zinc finger A20 and AN1 domain-containing stress- | -0.3528 | 1.6649 | 0.8181 | 0.7363 |
|  |  |  | associated protein 3 |  |  |  |  |
| C_4 | Gh_D07G1309 | EXPA17 | Putative expansin-A17 | 1.5869 | 0.5594 | -0.0828 | -1.5402 |
| C_4 | Gh_D07G1355 | ALMT10 | Aluminum-activated malate transporter 10 | -0.6288 | 0.7252 | -0.8917 | -2.0989 |
| C_4 | Gh_D07G1392 | GH3.5 | Probable indole-3-acetic acid-amido synthetase GH3.5 | -0.0802 | -0.3027 | -0.9819 | -1.1008 |
| C_4 | Gh_D07G1437 | SALR | Salutaridine reductase | -0.0786 | 0.6279 | -0.5093 | -0.5124 |
| C_4 | Gh_D07G1467 | GAE3 | UDP-glucuronate 4-epimerase 3 | 0.3431 | 0.9491 | 0.9026 | 0.4929 |
| C_4 | Gh_D07G1493 | NA | NA | -0.1170 | 0.8721 | -0.2062 | -0.5421 |
| C_4 | Gh_D07G1566 | RPL13AD | 60S ribosomal protein L13a-4 | 0.2500 | 0.9458 | 0.1019 | -0.6752 |
| C_4 | Gh_D07G1777 | POX2 | Proline dehydrogenase 2, mitochondrial | -0.6896 | -0.1052 | -1.1441 | -0.8261 |
| C_4 | Gh_D07G1956 | XERICO | Probable E3 ubiquitin-protein ligase XERICO | -0.7499 | 0.7299 | -1.6687 | -1.2512 |
| C_4 | Gh_D07G2322 | NFD4 | Protein NUCLEAR FUSION DEFECTIVE 4 | -0.1913 | 1.9254 | -0.0810 | 0.3439 |
| C_4 | Gh_D07G2342 | GDPD2 | Glycerophosphodiester phosphodiesterase GDPD2 | -0.3069 | 0.4055 | 0.0251 | -0.0724 |
| C_4 | Gh_D07G2405 | ELF3 | Protein EARLY FLOWERING 3 | -0.0870 | 0.6890 | -0.5436 | -0.3525 |
| C_4 | Gh_D07G2427 | PUB4 | U-box domain-containing protein 4 | -0.2796 | 0.6220 | -0.7558 | -0.6412 |
| C_4 | Gh_D08G0045 | SPAC24B11.0 | Uncharacterized protein C24B11.05 | 1.5878 | 2.3987 | -0.1725 | -0.8690 |
|  |  | 5 |  |  |  |  |  |
| C_4 | Gh_D08G0203 | RLP12 | Receptor-like protein 12 | -2.5634 | -0.2233 | -1.1668 | -1.1965 |
| C_4 | Gh_D08G0206 | NA | NA | 3.1504 | 2.3264 | 1.7907 | 1.4724 |
| C_4 | Gh_D08G0416 | EIF2B4 | Translation initiation factor eIF-2B subunit delta | 0.3122 | 0.5021 | -0.6121 | -0.5879 |
| C_4 | Gh_D08G0495 | RANGAP1 | RAN GTPase-activating protein 1 | 4.2651 | 3.1335 | 0.7653 | 0.4034 |
| C_4 | Gh_D08G0577 | At3g63540 | Thylakoid lumenal 19 kDa protein, chloroplastic | -0.3337 | 0.6867 | -1.0338 | -0.5313 |
| C_4 | Gh_D08G0609 | NA | NA | 0.6603 | 0.9275 | -0.1261 | -0.3628 |
| C_4 | Gh_D08G0621 | Os05g0200100 | Thioredoxin-like 2, chloroplastic | -0.8849 | 0.3648 | -0.2515 | 0.0185 |
| C_4 | Gh_D08G0668 | CPRF2 | Light-inducible protein CPRF2 | 0.2071 | 0.3616 | 0.1232 | 0.1085 |
| C_4 | Gh_D08G0730 | IPT3 | Adenylate isopentenyltransferase 3, chloroplastic | 0.4705 | 1.2810 | 0.5206 | 0.6890 |
| C_4 | Gh_D08G0786 | IDD2 | Protein indeterminate-domain 2 | 0.0424 | 1.2266 | -2.9326 | -1.3052 |
| C_4 | Gh_D08G0850 | TBC1D5 | TBC1 domain family member 5 | -0.2434 | 0.6888 | -0.3618 | -0.2525 |
| C_4 | Gh_D08G0993 | NA | NA | -0.1805 | 0.3192 | -0.4531 | -0.2068 |
| C_4 | Gh_D08G0999 | CCR4-6 | Carbon catabolite repressor protein 4 homolog 6 | 0.2201 | 0.3052 | -0.9742 | -0.5497 |
| C_4 | Gh_D08G1335 | GPAT3 | Probable glycerol-3-phosphate acyltransferase 3 | -0.1044 | 1.1153 | -0.2999 | 0.2200 |
| C_4 | Gh_D08G1354 | PMP3 | Plasma membrane proteolipid 3 | 0.2129 | 0.1197 | -0.1091 | -0.5310 |
| C_4 | Gh_D08G1363 | PALD1 | Paladin | -1.1082 | -0.0294 | -1.0517 | -0.5460 |
| C_4 | Gh_D08G1475 | At3g61520 | Pentatricopeptide repeat-containing protein | 2.9397 | 4.6036 | -0.8070 | -0.8005 |
| C_4 | Gh_D08G1493 | NA | NA | 0.0764 | 0.1349 | -1.3056 | -1.1164 |
| C_4 | Gh_D08G1497 | NA | NA | 0.1255 | 0.3176 | -0.2645 | -0.2428 |
| C_4 | Gh_D08G1561 | NA | NA | 0.0114 | 1.5264 | 0.4345 | -0.7735 |
| C_4 | Gh_D08G1597 | CESA3 | Cellulose synthase A catalytic subunit 3 [UDP-forming] | -0.0576 | 0.4084 | -0.0940 | 0.1080 |
| C_4 | Gh_D08G1629 | NFD4 | Protein NUCLEAR FUSION DEFECTIVE 4 | 0.4281 | 1.4980 | 1.0018 | 0.6523 |
| C_4 | Gh_D08G1642 | NA | Glycine-rich protein A3 | 0.5906 | 0.8863 | 0.5603 | 0.1999 |
| C_4 | Gh_D08G1648 | At4g19185 | WAT1-related protein At4g19185 | 0.0116 | 0.3241 | -0.1775 | -0.4206 |
| C_4 | Gh_D08G1718 | IRL5 | Plant intracellular Ras-group-related LRR protein 5 | 0.2427 | 0.8274 | 0.0227 | -0.0471 |
| C_4 | Gh_D08G1751 | NA | NA | -0.0733 | 0.7094 | -0.3189 | 0.0707 |
| C_4 | Gh_D08G2100 | AAO | L-ascorbate oxidase | 0.2830 | 0.2311 | -0.1429 | -0.4622 |
| C_4 | Gh_D08G2102 | NA | NA | -0.3057 | 0.9179 | -0.8351 | -0.1220 |
| C_4 | Gh_D08G2116 | GATA11 | GATA transcription factor 11 | 0.6837 | 2.2348 | 0.9506 | 0.7868 |
| C_4 | Gh_D08G2133 | NFD4 | Protein NUCLEAR FUSION DEFECTIVE 4 | -1.5644 | 1.1647 | -0.9022 | -0.0663 |
| C_4 | Gh_D08G2225 | NA | Linoleate 9S-lipoxygenase | 0.6343 | 3.5160 | -1.7987 | -2.1848 |
| C_4 | Gh_D08G2273 | AKR1 | Probable aldo-keto reductase 1 | -0.1734 | 1.2188 | -0.2657 | -0.1737 |
| C_4 | Gh_D08G2324 | CCX2 | Cation/calcium exchanger 2 | -0.2428 | 0.6769 | 0.2239 | 0.4856 |
| C_4 | Gh_D08G2457 | SAUR72 | Auxin-responsive protein SAUR72 | -0.0248 | 0.1764 | 0.2256 | -0.0025 |
| C_4 | Gh_D08G2484 | EBF1 | EIN3-binding F-box protein 1 | 0.2839 | 0.3545 | -0.4263 | -0.1969 |
| C_4 | Gh_D08G2555 | NPR1 | Regulatory protein NPR1 | -0.2430 | -0.0033 | -0.0093 | -0.2701 |
| C_4 | Gh_D08G2596 | At2g19810 | Zinc finger CCCH domain-containing protein 20 | -0.1779 | 0.1111 | -1.0152 | -1.0508 |
| C_4 | Gh_D08G2601 | At3g12360 | Ankyrin repeat-containing protein | 1.1031 | 0.7059 | 1.1822 | -1.7442 |
| C_4 | Gh_D08G2732 | NA | NA | -0.4922 | 2.7461 | -0.9282 | -0.7879 |
| C_4 | Gh_D09G0076 | NA | NA | -0.3420 | 0.4881 | 0.0473 | -1.8829 |
| C_4 | Gh_D09G0128 | EXPA5 | Expansin-A5 | 2.6479 | 3.7953 | 3.0466 | 0.7959 |
| C_4 | Gh_D09G0165 | At1g35710 | Probable leucine-rich repeat receptor-like protein kinase | 0.1001 | 1.4533 | 1.1556 | 0.2971 |
| C_4 | Gh_D09G0167 | At4g08850 | Probable LRR receptor-like serine/threonine-protein | -0.2410 | 0.8550 | 0.6570 | 0.1209 |
|  |  |  | kinase |  |  |  |  |
| C_4 | Gh_D09G0175 | WAKL9 | Wall-associated receptor kinase-like 9 | 0.3662 | 1.3803 | 0.8973 | 0.8560 |
| C_4 | Gh_D09G0234 | NPF5.8 | Protein NRT1/ PTR FAMILY 5.8 | -0.1199 | -0.8610 | -0.5187 | -2.2458 |
| C_4 | Gh_D09G0300 | GLCAK1 | Glucuronokinase 1 | -0.8207 | -0.0080 | -0.2846 | -0.4806 |
| C_4 | Gh_D09G0361 | CYP94B3 | Cytochrome P450 94B3 | -2.0079 | -0.4576 | 0.0513 | -1.9170 |
| C_4 | Gh_D09G0439 | NA | NA | -0.1006 | 0.4294 | 2.3678 | -5.2664 |
| C_4 | Gh_D09G0460 | NA | NA | -0.3915 | 0.1919 | -0.5515 | -1.3200 |
| C_4 | Gh_D09G0638 | BZIP1 | Basic leucine zipper 1 | -1.2983 | 2.0433 | -0.2679 | 0.4443 |
| C_4 | Gh_D09G0776 | NA | NA | 0.2122 | 1.5952 | -0.0987 | -0.3660 |
| C_4 | Gh_D09G0777 | SKP2A | F-box protein SKP2A | 0.5369 | 0.4546 | -0.2081 | -0.4992 |
| C_4 | Gh_D09G0880 | Dcun1d4 | DCN1-like protein 4 | -0.0567 | 0.4390 | -0.0320 | -0.6571 |
| C_4 | Gh_D09G0889 | AXS2 | UDP-D-apiose/UDP-D-xylose synthase 2 | -0.2747 | 0.5701 | -0.1529 | -0.0232 |
| C_4 | Gh_D09G0902 | At4g22758 | Uncharacterized protein | -0.8686 | 0.2337 | -1.0066 | -0.3960 |
| C_4 | Gh_D09G0944 | DIVARICAT | Transcription factor DIVARICATA | -0.3397 | 1.1638 | 0.0099 | -0.0024 |
|  |  | A |  |  |  |  |  |
| C_4 | Gh_D09G0993 | NA | Calmodulin | 0.3736 | 0.4289 | 0.2392 | -0.0834 |
| C_4 | Gh_D09G1064 | NADK1 | NAD(H) kinase 1 | 0.3665 | 0.2493 | -0.0092 | -0.5577 |
| C_4 | Gh_D09G1178 | PCMP-H85 | Putative pentatricopeptide repeat-containing protein | 1.9423 | 1.4533 | 2.4124 | -1.7556 |
| C_4 | Gh_D09G1183 | BHLH130 | Transcription factor bHLH130 | 0.0142 | -0.2521 | -0.5312 | -0.9609 |
| C_4 | Gh_D09G1503 | PAH1 | Phosphatidate phosphatase PAH1 | -0.6039 | 0.4198 | -0.4430 | -0.4512 |
| C_4 | Gh_D09G1528 | IPCS1 | Phosphatidylinositol:ceramide inositolphosphotransferase1 | 0.0771 | 1.3711 | 0.8320 | 0.2974 |
| C_4 | Gh_D09G1557 | nnt1 | Protein N-methyltransferase nnt1 | 1.2569 | 0.7348 | -0.2618 | -0.1799 |
| C_4 | Gh_D09G1621 | CRK1 | Cysteine-rich receptor-like protein kinase 1 | 0.1001 | 1.4533 | 0.5451 | 0.3317 |
| C_4 | Gh_D09G1681 | NA | NA | 0.1884 | 1.3831 | -0.1456 | 0.0584 |
| C_4 | Gh_D09G1807 | NA | NA | -0.6723 | 1.5097 | 1.4137 | -0.1548 |
| C_4 | Gh_D09G1808 | ERF061 | Ethylene-responsive transcription factor ERF061 | 1.8403 | 3.2629 | 2.9493 | 1.7068 |
| C_4 | Gh_D09G1816 | NA | NA | 0.1763 | 0.7699 | -1.4354 | -0.6627 |
| C_4 | Gh_D09G1881 | UPF1 | Regulator of nonsense transcripts 1 homolog | 0.0577 | 0.3057 | 0.3097 | -0.0723 |
| C_4 | Gh_D09G1912 | At3g23880 | F-box/kelch-repeat protein | -0.7404 | 0.5364 | -0.4380 | -0.8690 |
| C_4 | Gh_D09G1921 | At5g63930 | Probable leucine-rich repeat receptor-like protein kinase | -0.1250 | 0.3567 | 0.1643 | -0.1448 |
| C_4 | Gh_D09G1979 | FKBP65 | Peptidyl-prolyl cis-trans isomerase FKBP65 | -2.9555 | -0.4953 | -4.5372 | -2.9063 |
| C_4 | Gh_D09G1986 | NA | NA | 1.0097 | 3.9298 | -0.0329 | 0.3272 |
| C_4 | Gh_D09G2041 | NA | NA | -1.2886 | -0.1788 | -1.7616 | -1.7023 |
| C_4 | Gh_D09G2131 | ACA13 | Putative calcium-transporting ATPase 13, plasma | 0.3109 | 1.6292 | 0.8938 | 0.7562 |
|  |  |  | membrane-type |  |  |  |  |
| C_4 | Gh_D09G2166 | CSLG3 | Cellulose synthase-like protein G3 | -1.3131 | -0.2064 | -1.5231 | -2.8710 |
| C_4 | Gh_D09G2239 | SAMDC4 | S-adenosylmethionine decarboxylase proenzyme 4 | 1.5000 | 2.0470 | 1.6058 | 0.6917 |
| C_4 | Gh_D09G2284 | RPL19B | 60S ribosomal protein L19-2 | 0.0364 | 0.0462 | -0.6904 | -0.8890 |
| C_4 | Gh_D09G2287 | PGA3 | Exopolygalacturonase clone GBGE184 | 2.1864 | 3.0861 | 0.1662 | -0.2436 |
| C_4 | Gh_D09G2442 | Os11g0148500 | Pyruvate kinase 1, cytosolic | -0.0357 | 0.8016 | 0.6951 | 0.2350 |
| C_4 | Gh_D10G0005 | NA | NA | -0.8710 | 0.9659 | -0.5168 | -0.0827 |
| C_4 | Gh_D10G0074 | INRPK1 | Receptor-like protein kinase | 0.1507 | 1.4101 | 0.2409 | 0.5284 |
| C_4 | Gh_D10G0192 | NA | NA | -0.6087 | 1.6698 | 0.7148 | 0.0245 |
| C_4 | Gh_D10G0217 | TSJT1 | Stem-specific protein TSJT1 | -0.9377 | 1.0285 | -1.0204 | -0.3031 |
| C_4 | Gh_D10G0288 | PDC2 | Pyruvate decarboxylase 2 | 0.6640 | 2.2705 | 1.5670 | 1.5009 |
| C_4 | Gh_D10G0310 | STY17 | Serine/threonine-protein kinase STY17 | -0.2295 | 1.5634 | -0.7903 | 0.1929 |
| C_4 | Gh_D10G0311 | GORK | Potassium channel GORK | 0.5828 | 1.7022 | -0.4760 | 0.0505 |
| C_4 | Gh_D10G0437 | BRG1 | BOI-related E3 ubiquitin-protein ligase 1 | 0.0580 | -0.0114 | -0.6437 | -0.8930 |
| C_4 | Gh_D10G0472 | NA | NA | 0.0863 | 0.8481 | 0.8797 | 0.2867 |
| C_4 | Gh_D10G0487 | ALAAT2 | Alanine aminotransferase 2, mitochondrial | -0.3549 | 0.9881 | 0.3091 | 0.5458 |
| C_4 | Gh_D10G0505 | DNAJB6 | DnaJ homolog subfamily B member 6 | -0.3155 | 0.0489 | -0.5507 | -0.8028 |
| C_4 | Gh_D10G0554 | YLS9 | Protein YLS9 | -0.3001 | 0.4287 | 0.0956 | 0.3279 |
| C_4 | Gh_D10G0577 | At1g17710 | Inorganic pyrophosphatase 2 | -0.4271 | 1.2406 | -0.7588 | -2.7569 |
| C_4 | Gh_D10G0590 | ILL4 | IAA-amino acid hydrolase ILR1-like 4 | -0.5804 | 0.7262 | 0.2374 | -0.0430 |
| C_4 | Gh_D10G0672 | NA | NA | -0.6792 | 3.8393 | -2.6983 | -1.2434 |
| C_4 | Gh_D10G0678 | bshA | N-acetyl-alpha-D-glucosaminyl L-malate synthase | -0.0960 | 0.4155 | 0.3947 | 0.0523 |
| C_4 | Gh_D10G0730 | PLD1 | Phospholipase D alpha 1 | 0.1113 | 0.7580 | 0.1152 | 0.0182 |
| C_4 | Gh_D10G1084 | PRP38 | Pre-mRNA-splicing factor 38 | 0.0814 | 0.4508 | -0.4138 | -0.2700 |
| C_4 | Gh_D10G1117 | At5g37450 | Probable LRR receptor-like serine/threonine-protein | -0.0243 | -0.3227 | -2.1187 | -1.7244 |
|  |  |  | kinase |  |  |  |  |
| C_4 | Gh_D10G1301 | gemin2 | Gem-associated protein 2 | -0.2577 | 0.9153 | -0.2159 | -0.3722 |
| C_4 | Gh_D10G1364 | GAE6 | UDP-glucuronate 4-epimerase 6 | -0.1496 | 0.4625 | -0.2249 | -0.2561 |
| C_4 | Gh_D10G1494 | yqkD | Uncharacterized protein YqkD | 0.0503 | 0.4315 | -0.2005 | -0.2744 |
| C_4 | Gh_D10G1551 | At1g04430 | Probable methyltransferase PMT8 | 0.3604 | 0.4235 | 0.4777 | -0.0849 |
| C_4 | Gh_D10G1767 | BXL1 | Beta-D-xylosidase 1 | -0.7728 | 1.3135 | 0.4026 | 0.5902 |
| C_4 | Gh_D10G1789 | NA | NA | 1.2038 | 1.5871 | 0.0201 | 0.4432 |
| C_4 | Gh_D10G2014 | CYP94A1 | Cytochrome P450 94A1 | -0.8232 | 0.5956 | -0.3771 | -0.6226 |
| C_4 | Gh_D10G2075 | ACT | Vinorine synthase | -1.1567 | 0.7823 | -0.2229 | -0.2525 |
| C_4 | Gh_D10G2301 | AHL15 | AT-hook motif nuclear-localized protein 15 | 0.3630 | 0.9364 | 0.2312 | 0.2489 |
| C_4 | Gh_D10G2354 | At3g50280 | Uncharacterized acetyltransferase At3g50280 | -0.2364 | 0.5363 | -0.0430 | 0.1824 |
| C_4 | Gh_D10G2374 | At3g47570 | Probable LRR receptor-like serine/threonine-protein | -0.0561 | -1.6138 | -3.5481 | -3.5777 |
|  |  |  | kinase |  |  |  |  |
| C_4 | Gh_D10G2433 | At4g17915 | Putative pentatricopeptide repeat-containing protein | -0.1218 | -0.0082 | -0.4335 | -2.2008 |
| C_4 | Gh_D10G2438 | AAE16 | Probable acyl-activating enzyme 16, chloroplastic | -0.2860 | -0.0534 | -0.1763 | -0.1481 |
| C_4 | Gh_D10G2454 | ANS | Leucoanthocyanidin dioxygenase | -1.1106 | 0.5213 | -0.0430 | -0.2273 |
| C_4 | Gh_D11G0009 | WER | Transcription factor WER | 1.5764 | 0.7049 | -0.8172 | -0.4670 |
| C_4 | Gh_D11G0026 | VAR3 | Zinc finger protein VAR3, chloroplastic | 0.5891 | 0.3023 | -1.8810 | -2.6442 |
| C_4 | Gh_D11G0138 | COL5 | Zinc finger protein CONSTANS-LIKE 5 | -1.0986 | 1.6636 | -1.2788 | -0.0694 |
| C_4 | Gh_D11G0140 | CBL4 | Calcineurin B-like protein 4 | -0.2672 | 0.5681 | -0.5657 | -0.2442 |
| C_4 | Gh_D11G0212 | wge | Protein winged eye | 0.0752 | 0.5620 | -0.1769 | -0.5124 |
| C_4 | Gh_D11G0236 | NA | NA | -0.6959 | 1.6955 | -0.1081 | 0.0111 |
| C_4 | Gh_D11G0237 | GSVIVT0002 | Probable polygalacturonase | -0.4494 | 0.9384 | -0.2639 | 0.3342 |
|  |  | 6920001 |  |  |  |  |  |
| C_4 | Gh_D11G0239 | ERF061 | Ethylene-responsive transcription factor ERF061 | -0.3609 | 1.6484 | 1.2568 | 0.7652 |
| C_4 | Gh_D11G0282 | NA | Lon protease homolog 2, peroxisomal | 0.0909 | 0.9545 | 0.1121 | 0.2929 |
| C_4 | Gh_D11G0288 | BOU | Mitochondrial carnitine/acylcarnitine carrier-like protein | 0.7445 | 0.2843 | 0.3781 | -0.3102 |
| C_4 | Gh_D11G0291 | AGD8 | Probable ADP-ribosylation factor GTPase-activating | 0.0623 | 0.6200 | 0.5162 | 0.2766 |
|  |  |  | protein AGD8 |  |  |  |  |
| C_4 | Gh_D11G0310 | NFD4 | Protein NUCLEAR FUSION DEFECTIVE 4 | -0.6380 | 0.3038 | -0.6438 | -0.9773 |
| C_4 | Gh_D11G0394 | KING1 | SNF1-related protein kinase regulatory subunit gamma-1 | -0.1517 | 0.5183 | -0.1461 | 0.1497 |
| C_4 | Gh_D11G0426 | ERF2 | Ethylene-responsive transcription factor 2 | -0.3697 | 1.7454 | -0.9536 | -0.8035 |
| C_4 | Gh_D11G0427 | ERF106 | Ethylene-responsive transcription factor ERF106 | -0.0470 | 3.1299 | -0.1345 | -0.4831 |
| C_4 | Gh_D11G0582 | NA | Systemin receptor SR160 | -0.1963 | 0.5463 | 0.3052 | -0.0623 |
| C_4 | Gh_D11G0714 | HISN4 | Imidazole glycerol phosphate synthase hisHF, | -0.2073 | 0.5538 | -0.0023 | -0.1144 |
|  |  |  | chloroplastic |  |  |  |  |
| C_4 | Gh_D11G0716 | At3g28050 | WAT1-related protein | -0.9039 | 0.5866 | -0.8094 | -0.6328 |
| C_4 | Gh_D11G0734 | AVT1 | Vacuolar amino acid transporter 1 | -0.4351 | 0.2072 | 0.0694 | -0.0824 |
| C_4 | Gh_D11G0834 | PVA12 | Vesicle-associated protein 1-2 | 0.3471 | 0.8717 | 0.2254 | 0.1126 |
| C_4 | Gh_D11G0923 | NA | Remorin | 0.5388 | 1.9065 | 0.1810 | 0.4115 |
| C_4 | Gh_D11G0978 | LEA14-A | Late embryogenesis abundant protein Lea14-A | 0.3985 | 2.4032 | 0.2249 | -0.4214 |
| C_4 | Gh_D11G1084 | PALD1 | Paladin | -0.8610 | 0.1737 | -0.9473 | -1.1440 |
| C_4 | Gh_D11G1141 | WRKY65 | Probable WRKY transcription factor 65 | -0.2686 | 0.5982 | -0.8746 | -1.0817 |
| C_4 | Gh_D11G1236 | VIT_07s0104g | U1 small nuclear ribonucleoprotein C | 0.5494 | 0.7285 | 0.0573 | -0.3007 |
|  |  | 01170 |  |  |  |  |  |
| C_4 | Gh_D11G1353 | H6H | Hyoscyamine 6-dioxygenase | -0.3423 | -0.3189 | -1.2476 | -0.9756 |
| C_4 | Gh_D11G1365 | EXO84A | Exocyst complex component EXO84A | -0.4701 | -0.3526 | -0.3692 | -0.5527 |
| C_4 | Gh_D11G1378 | KAM1 | Xyloglucan galactosyltransferase KATAMARI1 | 0.7486 | 1.5608 | 0.9580 | 1.0940 |
| C_4 | Gh_D11G1417 | PRT1 | E3 ubiquitin-protein ligase PRT1 | 0.1841 | 0.6291 | 0.0901 | -0.7817 |
| C_4 | Gh_D11G1446 | At3g17800 | UV-B-induced protein | 0.2227 | 0.7942 | -1.0735 | -0.9697 |
| C_4 | Gh_D11G1580 | At3g21620 | CSC1-like protein | -0.3946 | -0.1430 | -0.9690 | -2.2231 |
| C_4 | Gh_D11G1596 | ZIP5 | Zinc transporter 5 | -0.4621 | -0.0738 | -0.7693 | -0.8548 |
| C_4 | Gh_D11G1625 | COBL1 | COBRA-like protein 1 | -1.9797 | -0.6068 | -1.2775 | -1.2574 |
| C_4 | Gh_D11G1764 | NA | Thaumatin-like protein 1 | -0.8370 | 0.8288 | -0.8604 | -1.1733 |
| C_4 | Gh_D11G1985 | At5g24010 | Probable receptor-like protein kinase | 0.5010 | 1.8797 | 0.7285 | 0.9765 |
| C_4 | Gh_D11G2009 | NA | NA | -0.1574 | 1.2619 | -0.6144 | 0.0978 |
| C_4 | Gh_D11G2185 | NA | NA | 0.3078 | 1.5163 | 0.2533 | -0.7134 |
| C_4 | Gh_D11G2201 | TPPJ | Probable trehalose-phosphate phosphatase J | 0.1003 | 0.5968 | -0.9194 | -1.0763 |
| C_4 | Gh_D11G2279 | GSVIVT0002 | Probable polygalacturonase | 1.3703 | 1.8330 | 1.0018 | 0.4331 |
|  |  | 6920001 |  |  |  |  |  |
| C_4 | Gh_D11G2288 | At3g12360 | Ankyrin repeat-containing protein | 0.6719 | 2.1001 | 1.5155 | -1.6107 |
| C_4 | Gh_D11G2293 | PUB27 | U-box domain-containing protein 27 | 0.0917 | 0.0765 | -0.0135 | -0.3444 |
| C_4 | Gh_D11G2411 | TUBA3 | Tubulin alpha-3 chain | 0.9981 | 1.3863 | 0.5022 | 0.2395 |
| C_4 | Gh_D11G2561 | CPRF2 | Light-inducible protein CPRF2 | -0.4861 | 0.7674 | -0.3302 | 0.0384 |
| C_4 | Gh_D11G2761 | CXE15 | Probable carboxylesterase 15 | -0.5766 | 0.7498 | -0.7220 | -1.2033 |
| C_4 | Gh_D11G2771 | GRDP1 | Glycine-rich domain-containing protein 1 | 0.3750 | 0.0841 | 0.5188 | -0.8476 |
| C_4 | Gh_D11G2773 | PP2B15 | F-box protein PP2-B15 | 0.7961 | 1.2249 | 0.8513 | -0.3366 |
| C_4 | Gh_D11G2819 | NUP160 | Nuclear pore complex protein NUP160 | 0.1601 | 0.2859 | 0.0086 | -0.3555 |
| C_4 | Gh_D11G2903 | N | TMV resistance protein N | 0.7518 | 0.3593 | 0.4223 | -0.3698 |
| C_4 | Gh_D11G2975 | SWEET2 | Bidirectional sugar transporter SWEET2 | -0.3061 | 0.9788 | 0.1264 | -0.0016 |
| C_4 | Gh_D11G2988 | UBP15 | Ubiquitin carboxyl-terminal hydrolase 15 | 0.2433 | 0.7677 | 0.3288 | 0.0174 |
| C_4 | Gh_D11G3051 | TFCA | Tubulin-folding cofactor A | 0.1617 | 0.5777 | 0.4772 | 0.2344 |
| C_4 | Gh_D11G3112 | At4g27220 | Probable disease resistance protein | 0.2402 | 1.0165 | 0.2437 | 0.5272 |
| C_4 | Gh_D11G3115 | NA | NA | 0.5484 | 2.0247 | 0.9771 | 1.1586 |
| C_4 | Gh_D11G3171 | DIR21 | Dirigent protein 21 | 1.0526 | 0.0280 | -0.1296 | -0.6951 |
| C_4 | Gh_D11G3175 | RF178 | Probable E3 ubiquitin-protein ligase BAH1-like | -0.3422 | 0.0802 | -0.4384 | -2.0646 |
| C_4 | Gh_D11G3237 | NA | NA | -0.0609 | 0.0448 | -0.2601 | -0.1592 |
| C_4 | Gh_D11G3239 | NA | NA | 0.0703 | 0.0105 | -0.4412 | -0.4159 |
| C_4 | Gh_D11G3381 | NA | NA | -2.5747 | -2.3647 | -3.7564 | -3.6767 |
| C_4 | Gh_D12G0026 | SULTR3 | Sulfate transporter 3.1 | 0.0386 | 1.5462 | -0.5777 | 0.1363 |
| C_4 | Gh_D12G0054 | NA | NA | 0.5035 | 0.4871 | -0.1779 | -0.3644 |
| C_4 | Gh_D12G0056 | bsdc1 | BSD domain-containing protein 1 | 0.1867 | 0.2605 | 0.1905 | -0.0894 |
| C_4 | Gh_D12G0135 | At5g64080 | Non-specific lipid-transfer protein-like protein | 0.6042 | 1.1553 | 0.6721 | 0.5983 |
| C_4 | Gh_D12G0236 | NA | NA | -0.2144 | 1.3052 | -0.4988 | -0.7091 |
| C_4 | Gh_D12G0263 | At2g16365 | F-box protein At2g16365 | 0.0015 | 1.5470 | 0.5301 | 0.3490 |
| C_4 | Gh_D12G0315 | SPG20 | Spartin | 0.5930 | 1.7418 | 0.3258 | 0.0257 |
| C_4 | Gh_D12G0494 | PCMP-H43 | Pentatricopeptide repeat-containing protein | 2.0289 | 3.1194 | -0.2521 | 0.3449 |
| C_4 | Gh_D12G0561 | NIP1 | NEP1-interacting protein 1 | -0.4084 | -0.0966 | -1.2270 | -1.1583 |
| C_4 | Gh_D12G0591 | BHLH110 | Transcription factor bHLH110 | 0.3658 | 0.5456 | 0.7333 | -0.4846 |
| C_4 | Gh_D12G0607 | HBI1 | Transcription factor HBI1 | 0.6085 | 0.3668 | 0.3810 | -1.4980 |
| C_4 | Gh_D12G0645 | VRN1 | B3 domain-containing transcription factor VRN1 | 0.1350 | 0.5558 | -0.4412 | -0.2472 |
| C_4 | Gh_D12G0672 | At5g67140 | F-box protein At5g67140 | -1.2270 | 0.4837 | -0.4822 | 0.0553 |
| C_4 | Gh_D12G0676 | ERF008 | Ethylene-responsive transcription factor ERF008 | 0.1709 | 1.0076 | 0.4916 | 0.2924 |
| C_4 | Gh_D12G0905 | NA | NA | -1.0471 | 0.8867 | -0.9553 | -0.3938 |
| C_4 | Gh_D12G1002 | RPPL1 | Putative disease resistance RPP13-like protein 1 | -0.6911 | 0.8466 | -0.4440 | -0.5575 |
| C_4 | Gh_D12G1044 | PP2A13 | F-box protein PP2-A13 | -0.9213 | 2.6882 | -1.6534 | -1.0797 |
| C_4 | Gh_D12G1068 | GC4 | Golgin candidate 4 | -0.4244 | 0.5459 | -0.1271 | -0.2098 |
| C_4 | Gh_D12G1243 | WRKY23 | Probable WRKY transcription factor 23 | -0.2677 | 1.3187 | -0.5372 | -0.0508 |
| C_4 | Gh_D12G1316 | EID1 | Phytochrome A-associated F-box protein | 0.1831 | 0.7498 | 0.5055 | 0.5348 |
| C_4 | Gh_D12G1317 | BAH1 | E3 ubiquitin-protein ligase BAH1 | 0.1687 | 0.4083 | -0.3857 | -0.2438 |
| C_4 | Gh_D12G1348 | RGP1 | RAB6A-GEF complex partner protein 2 | 0.3966 | 0.4858 | 0.5318 | 0.3207 |
| C_4 | Gh_D12G1378 | NA | NA | -0.2029 | 1.8053 | 0.6905 | -0.9354 |
| C_4 | Gh_D12G1480 | RNF141 | RING finger protein 141 | -0.6900 | 0.3185 | -0.9303 | -0.9373 |
| C_4 | Gh_D12G1540 | ASB1 | Anthranilate synthase beta subunit 1, chloroplastic | -0.2618 | 1.4108 | 0.2741 | 0.2221 |
| C_4 | Gh_D12G1664 | NA | NA | -0.2139 | 0.9175 | -0.0147 | -0.2469 |
| C_4 | Gh_D12G1761 | NAC100 | NAC domain-containing protein 100 | -0.4811 | -0.3350 | 0.0219 | -1.0640 |
| C_4 | Gh_D12G1873 | EXO70B1 | Exocyst complex component EXO70B1 | -0.0252 | 0.2789 | -0.0286 | -0.0986 |
| C_4 | Gh_D12G1876 | At1g67340 | F-box protein At1g67340 | -0.0945 | 0.8687 | -1.3069 | -0.4207 |
| C_4 | Gh_D12G1889 | NA | NA | 1.1073 | 0.9710 | 1.0455 | -0.9743 |
| C_4 | Gh_D12G2110 | NA | NA | 0.6158 | 0.9097 | -0.0172 | 0.1364 |
| C_4 | Gh_D12G2188 | NA | 21 kDa protein | -0.2380 | -0.3213 | -1.2152 | -1.6003 |
| C_4 | Gh_D12G2218 | At1g12500 | Probable sugar phosphate/phosphate translocator | -0.0719 | 0.9257 | 0.5242 | 0.2920 |
| C_4 | Gh_D12G2233 | At4g22990 | SPX domain-containing membrane protein | 0.0184 | 1.8396 | 1.3209 | 0.1515 |
| C_4 | Gh_D12G2244 | UGE1 | Bifunctional UDP-glucose 4-epimerase and UDP-xylose | -0.2272 | 1.7541 | 0.0919 | 0.8640 |
|  |  |  | 4-epimerase 1 |  |  |  |  |
| C_4 | Gh_D12G2486 | PP2A13 | F-box protein PP2-A13 | -0.3975 | 0.7057 | -0.0588 | 0.0078 |
| C_4 | Gh_D12G2564 | stk11ip | Serine/threonine-protein kinase 11-interacting protein | 0.0631 | 0.2711 | 0.1345 | -0.4347 |
| C_4 | Gh_D12G2767 | NAC029 | NAC transcription factor 29 | -3.0637 | 1.2995 | -2.3972 | -1.3358 |
| C_4 | Gh_D12G2779 | NA | NA | -0.2134 | 3.2377 | -0.3100 | -0.5374 |
| C_4 | Gh_D12G2811 | NA | NA | 0.8674 | 3.2190 | -0.6698 | -0.7583 |
| C_4 | Gh_D13G0029 | ERD15 | Protein EARLY RESPONSIVE TO DEHYDRATION 15 | 0.1971 | 0.7468 | 0.6374 | 0.0512 |
| C_4 | Gh_D13G0058 | PUB62 | U-box domain-containing protein 62 | 0.1232 | -0.0373 | -0.8259 | -1.1781 |
| C_4 | Gh_D13G0129 | CKX6 | Cytokinin dehydrogenase 6 | -1.1567 | -0.0009 | -1.2518 | -1.0274 |
| C_4 | Gh_D13G0187 | Gucd1 | Protein GUCD1 | -0.5143 | -0.0662 | -0.0837 | -0.5053 |
| C_4 | Gh_D13G0192 | At4g25210 | Mediator-associated protein 1 | 1.8752 | 0.9541 | -0.0127 | -0.1449 |
| C_4 | Gh_D13G0196 | NA | NA | 0.7391 | 0.4088 | -0.1516 | -2.5207 |
| C_4 | Gh_D13G0369 | BOA | Transcription factor BOA | 0.4417 | 0.4387 | -0.8632 | -0.5568 |
| C_4 | Gh_D13G0457 | ISPH | 4-hydroxy-3-methylbut-2-enyl diphosphate reductase, | -0.2952 | 1.0107 | 0.4853 | -0.0721 |
|  |  |  | chloroplastic |  |  |  |  |
| C_4 | Gh_D13G0506 | NA | NA | -1.0806 | 0.3944 | 0.2040 | -2.7639 |
| C_4 | Gh_D13G0510 | NA | NA | -1.5498 | -1.1481 | -0.4182 | -2.2390 |
| C_4 | Gh_D13G0532 | NA | NA | -0.0081 | 0.9957 | -0.2667 | -0.1433 |
| C_4 | Gh_D13G0669 | PXMP2 | Peroxisomal membrane protein 2 | -0.5827 | 0.6635 | 0.0735 | 0.0505 |
| C_4 | Gh_D13G0782 | At3g19950 | E3 ubiquitin-protein ligase RING1-like | -0.3946 | -0.0310 | -1.8534 | -1.9161 |
| C_4 | Gh_D13G0839 | ARID2 | AT-rich interactive domain-containing protein 2 | 0.0700 | 0.5437 | -0.4032 | -0.2211 |
| C_4 | Gh_D13G1037 | At5g07610 | F-box protein | -2.1084 | 0.8469 | -3.4250 | -1.8201 |
| C_4 | Gh_D13G1152 | PP2B15 | F-box protein PP2-B15 | -1.2169 | 0.9759 | -0.2824 | -3.1331 |
| C_4 | Gh_D13G1299 | At2g44510 | Protein BCCIP homolog | 0.3540 | 0.0470 | -0.9045 | -1.2881 |
| C_4 | Gh_D13G1325 | OCT3 | Organic cation/carnitine transporter 3 | -1.3035 | 0.0105 | -0.8119 | -0.9359 |
| C_4 | Gh_D13G1505 | BGLU41 | Putative beta-glucosidase 41 | -0.3281 | 1.2311 | -0.0294 | -0.4112 |
| C_4 | Gh_D13G1530 | NA | NA | 0.6247 | 1.2712 | 0.1553 | 0.1573 |
| C_4 | Gh_D13G1631 | CODM | Codeine O-demethylase | 0.0222 | 0.4224 | 0.0487 | -0.0404 |
| C_4 | Gh_D13G1729 | SRC1 | Protein SRC1 | 0.5856 | 1.4349 | -0.0794 | -0.1462 |
| C_4 | Gh_D13G1827 | RPL2 | 60S ribosomal protein L2, mitochondrial | 0.1875 | 0.0487 | -0.2341 | -0.3569 |
| C_4 | Gh_D13G1829 | At4g32640 | Protein transport protein Sec24-like | -0.4278 | 0.0165 | -0.3088 | -0.2157 |
| C_4 | Gh_D13G1885 | NA | NA | -0.4157 | 0.2376 | 0.2351 | -0.4793 |
| C_4 | Gh_D13G1890 | CHLN | Nicotianamine synthase | -2.6919 | 0.9449 | 0.2147 | -2.0323 |
| C_4 | Gh_D13G1920 | PIRL6 | Plant intracellular Ras-group-related LRR protein 6 | -0.2964 | -0.0506 | -0.0021 | -0.2731 |
| C_4 | Gh_D13G1953 | ZFP3 | Zinc finger protein 3 | -0.0301 | 1.0370 | -0.9038 | -0.5255 |
| C_4 | Gh_D13G1966 | EBF1 | EIN3-binding F-box protein 1 | 0.8720 | 0.9318 | 0.6278 | 0.0787 |
| C_4 | Gh_D13G1979 | NA | Omega-6 fatty acid desaturase, chloroplastic | -0.0267 | 1.1553 | 0.6206 | 0.1892 |
| C_4 | Gh_D13G1982 | RTNLB21 | Reticulon-like protein B21 | -0.0973 | 1.0757 | 0.3578 | 0.2561 |
| C_4 | Gh_D13G2146 | SYP32 | Syntaxin-32 | 0.2479 | 0.2718 | 0.2018 | -0.4842 |
| C_4 | Gh_D13G2147 | NA | NA | 0.2293 | 0.5057 | 0.3951 | -0.0166 |
| C_4 | Gh_D13G2163 | AOP1.2 | Probable 2-oxoglutarate-dependent dioxygenase | 0.3501 | -0.1751 | 0.0639 | -3.1179 |
| C_4 | Gh_D13G2346 | Nudcd2 | NudC domain-containing protein 2 | 0.1125 | 0.4417 | -0.0590 | -0.0634 |
| C_4 | Gh_D13G2385 | ACR9 | ACT domain-containing protein ACR9 | -1.5340 | -0.0379 | -0.8749 | -0.9683 |
| C_4 | Gh_D13G2427 | EVN | Dolichol kinase EVAN | -0.3019 | 0.8517 | 0.2951 | -0.1133 |
| C_4 | Gh_Sca004717G15 | NA | NA | 0.0802 | -0.2383 | -1.5750 | -2.4502 |
| C_4 | Gh_Sca004807G02 | NA | NA | 0.5430 | 3.5393 | -0.6755 | -1.8935 |
| C_4 | Gh_Sca004807G04 | NA | NA | -0.2678 | 2.9914 | 0.0497 | -0.3603 |
| C_4 | Gh_Sca004811G02 | NA | NA | -0.0321 | 2.7567 | 0.1415 | 0.0515 |
| C_4 | Gh_Sca004811G04 | NA | NA | -0.0364 | 3.1360 | 0.3673 | 0.0139 |
| C_4 | Gh_Sca004815G02 | NA | NA | -0.1383 | 2.8323 | 0.1333 | -0.0275 |
| C_4 | Gh_Sca004839G01 | NA | NA | -0.1399 | 2.8040 | -0.1996 | -0.3085 |
| C_4 | Gh_Sca004839G02 | NA | NA | -0.4029 | 2.4935 | 0.4050 | 0.0084 |
| C_4 | Gh_Sca004851G03 | UXS6 | UDP-glucuronic acid decarboxylase 6 | -0.1360 | 0.6086 | -0.0677 | 0.2535 |
| C_4 | Gh_Sca004857G01 | NA | NA | -0.6119 | 0.2314 | -1.5701 | -0.9679 |
| C_4 | Gh_Sca004940G01 | NA | NA | -0.3010 | 2.9633 | -0.7470 | -0.3620 |
| C_4 | Gh_Sca004940G02 | NA | NA | -0.1663 | 2.9264 | -0.4375 | -0.9417 |
| C_4 | Gh_Sca004965G03 | UPL1 | E3 ubiquitin-protein ligase UPL1 | 0.0485 | 0.0551 | -0.0424 | -0.2161 |
| C_4 | Gh_Sca004992G03 | NA | NA | -0.3713 | 2.6349 | 0.0230 | -0.3096 |
| C_4 | Gh_Sca005000G01 | NA | NA | -0.1349 | 2.8757 | -0.0199 | -0.7343 |
| C_4 | Gh_Sca005000G03 | NA | NA | 0.3654 | 3.1658 | -0.3950 | -0.5356 |
| C_4 | Gh_Sca005093G01 | NA | NA | 0.3818 | 3.5055 | 0.1624 | -0.2584 |
| C_4 | Gh_Sca005105G01 | ZIP1 | Zinc transporter 1 | -1.7380 | 0.7921 | -2.6958 | -2.1619 |
| C_4 | Gh_Sca005148G02 | NA | NA | -0.2486 | 3.1803 | 0.0012 | -0.5057 |
| C_4 | Gh_Sca005148G03 | NA | NA | 0.0575 | 2.7440 | -0.0103 | -0.2606 |
| C_4 | Gh_Sca005163G01 | BGAL1 | Beta-galactosidase 1 | 0.3597 | 1.0142 | 0.2891 | 0.4675 |
| C_4 | Gh_Sca005165G01 | NA | Glycine-rich RNA-binding protein GRP1A | 0.0855 | -0.3492 | -1.6258 | -2.0406 |
| C_4 | Gh_Sca005244G01 | NA | Transcription factor MYB1R1 | -0.7088 | 1.7537 | -0.1036 | -0.2011 |
| C_4 | Gh_Sca005670G01 | NA | NA | -0.1276 | 0.3840 | -1.3997 | -1.1034 |
| C_4 | Gh_Sca005710G01 | NA | NA | 0.1712 | 2.8017 | -0.4841 | -0.9622 |
| C_4 | Gh_Sca005787G06 | psbA | Photosystem II protein D1 | -0.4366 | 3.7356 | 1.2409 | 0.7876 |
| C_4 | Gh_Sca005849G01 | NA | NA | -0.2581 | 2.2941 | 0.0366 | -0.5449 |
| C_4 | Gh_Sca005954G01 | ISPH | 4-hydroxy-3-methylbut-2-enyl diphosphate reductase, | 0.1001 | 1.4533 | 0.0548 | -0.2845 |
|  |  |  | chloroplastic |  |  |  |  |
| C_4 | Gh_Sca006141G01 | accD | Acetyl-coenzyme A carboxylase carboxyl transferase | -0.5133 | 1.9007 | 0.2905 | -0.3358 |
|  |  |  | subunit beta, chloroplastic |  |  |  |  |
| C_4 | Gh_Sca006566G05 | NAD7 | NADH dehydrogenase [ubiquinone] iron-sulfur protein 2 | 0.4747 | 1.5532 | 0.3100 | -0.7354 |
| C_4 | Gh_Sca006659G01 | At3g07870 | F-box protein | -0.4675 | 0.8686 | -0.5122 | -0.1935 |
| C_4 | Gh_Sca006770G02 | NA | NA | -0.2603 | 2.4236 | -0.3541 | -0.8238 |
| C_4 | Gh_Sca007315G01 | NA | NA | -0.0169 | 3.5923 | 1.7422 | 0.2886 |
| C_4 | Gh_Sca007486G01 | NA | NA | -0.3170 | 5.1216 | -1.1715 | 0.3608 |
| C_4 | Gh_Sca007622G02 | petA | Cytochrome f | -1.3722 | 1.4622 | 0.6625 | -0.1747 |
| C_4 | Gh_Sca007624G01 | NA | NA | 0.4275 | 2.8371 | -0.0517 | -0.5910 |
| C_4 | Gh_Sca007624G04 | NA | NA | 0.3591 | 3.3394 | -0.0686 | -0.6123 |
| C_4 | Gh_Sca007624G06 | NA | NA | -0.2449 | 2.9945 | 0.2480 | -0.9465 |
| C_4 | Gh_Sca008391G01 | NA | NA | -0.0943 | 3.1525 | 0.1832 | -0.1095 |
| C_4 | Gh_Sca008493G01 | At2g17570 | Dehydrodolichyl diphosphate synthase 6 | -0.9488 | -0.0093 | -0.1594 | -0.7178 |
| C_4 | Gh_Sca008593G01 | NA | NA | -0.4635 | 0.1128 | 0.3206 | -0.5497 |
| C_4 | Gh_Sca008679G03 | psbA | Photosystem II protein D1 | -0.6565 | 3.7233 | 0.5444 | -2.7386 |
| C_4 | Gh_Sca009781G01 | NA | NA | 0.0627 | 3.1584 | -0.9899 | -0.6819 |
| C_4 | Gh_Sca009781G02 | NA | NA | -0.2100 | 3.6857 | 0.0751 | -0.2895 |
| C_4 | Gh_Sca011694G01 | rbcL | Ribulose bisphosphate carboxylase large chain | 0.0265 | 2.4729 | 2.2231 | -0.3908 |
| C_4 | Gh_Sca011730G01 | rpoC1 | DNA-directed RNA polymerase subunit beta | -0.4957 | 1.1134 | 0.9914 | -0.0841 |
| C_4 | Gh_Sca016160G01 | NA | NA | -0.5836 | 2.3588 | -2.1268 | -3.0008 |
| C_4 | Gh_Sca016326G01 | NA | NA | 0.1010 | 1.2684 | 0.6631 | -0.0891 |
| C_4 | Gh_Sca017056G01 | NA | NA | 0.1971 | 0.7279 | 0.9090 | -0.5347 |
| C_4 | Gh_Sca017233G01 | NA | NA | 0.1773 | 2.7628 | 0.3945 | 0.0334 |
| C_4 | Gh_Sca017679G01 | NA | NA | 0.7994 | 2.5370 | -0.2372 | -1.5714 |
| C_4 | Gh_Sca018012G01 | psbA | Photosystem II protein D1 | -1.8002 | 3.3620 | -0.4649 | -1.9450 |
| C_4 | Gh_Sca024064G01 | OCT3 | Organic cation/carnitine transporter 3 | -1.3278 | -0.1977 | -0.7404 | -1.2718 |
| C_4 | Gh_Sca029103G01 | AtMg00660 | Uncharacterized mitochondrial protein | 0.5609 | 0.8722 | 0.4552 | -0.3735 |
| C_4 | Gh_Sca035048G01 | RPL2 | 60S ribosomal protein L2, mitochondrial | 0.4092 | 0.9319 | 0.4563 | -0.4001 |
