# Supplementary material for: Transcriptome analysis of a nematode resistant and susceptible upland cotton line at two critical stages of Meloidogyne incognita infection and development
Source: PLoS One. 2019 Sep 10;14(9):e0221328. doi: 10.1371/journal.pone.0221328 (PMC6736245; doi:10.1371/journal.pone.0221328)
Supplement: S2 Table — (DOCX) [file pone.0221328.s003.docx]

| **Gene** | **Gene name** | **Annotation** | **Primer seq 5' to 3'** | **Len** | **MW** | **% GC** | **Tm** |
| --- | --- | --- | --- | --- | --- | --- | --- |
| Gh_A11G2835 | RGA3 | Predicted: putative disease resistance protein RGA3 | F_ATCTCAAACGCACCGTCTCC | 20 | 5982 | 55 | 62 |
|  |  |  | R_TCAAGCAAGTCATCGGCATCA | 21 | 6399 | 48 | 61 |
| Gh_A11G2836 | RPPL1 | Putative disease resistance RPP13-like protein 1 | F_TGAAGGTGGATGGGTGGAAG | 20 | 6342 | 55 | 62 |
|  |  |  | R_TGAGCAAAGGACGGGAGATG | 20 | 6280 | 55 | 62 |
| Gh_A11G3090 | PUB21 | PREDICTED: U-box domain-containing protein 21-like | F_TTGGAGAAATCGCAGAGCC | 19 | 5862 | 53 | 60 |
|  |  |  | R_GAGAAATAGGACATCGGAAATGTG | 24 | 7499 | 42 | 61 |
| Gh_A11G3216 | - | PREDICTED: cytochrome P450 CYP73A100-like | F_ATTCCATTGCTACGACCCTT | 20 | 6003 | 45 | 58 |
|  |  |  | R_GCTTATCTTGTGCTTGTCCAT | 21 | 6369 | 43 | 59 |
| Gh_A11G3289 | At1g67000 | PREDICTED: rust resistance kinase Lr10-like | F_TGAAACTAGAATTACCTCCATTATCAAG | 28 | 8540 | 32 | 60 |
|  |  |  | R_CGACGGCTGAGTTTTAAGC | 19 | 5844 | 53 | 60 |
| Gh_D02G0201 | WAKL1 | PREDICTED: wall-associated receptor kinase-like 1 | F_CGGGTCTTAGTTCTTTTGTCGC | 22 | 6715 | 50 | 63 |
|  |  |  | R_TTGGTTGTTGTGTCATTGTGGTC | 23 | 7114 | 44 | 61 |
| Gh_D02G0257 | RLP12 | PREDICTED: receptor-like protein 12 | F_TGGTCTTCCAATGCTACCACC | 21 | 6317 | 52 | 63 |
|  |  |  | R_ACATCTTCTTCTATTTCTTTTCCCACAC | 28 | 8351 | 36 | 62 |
| Gh_D02G0259 | RLP12 | PREDICTED: receptor-like protein 12 | F_CTTCCAATGCCACCACTTC | 19 | 5644 | 53 | 60 |
|  |  |  | R_CAACGACCAATCCACTAGC | 19 | 5711 | 53 | 60 |
| Gh_D02G0264 | PHT1-5 | PREDICTED: probable inorganic phosphate transporter 1-5 | F_GAGAAGGTGGATAAGATAGGGAGAG | 25 | 7933 | 48 | 65 |
|  |  |  | R_CGCTTTAGGGAGCCAACCAA | 20 | 6111 | 55 | 62 |
| Gh_D02G0227 | UGT75L6 | PREDICTED: crocetin glucosyltransferase, chloroplastic-like | F_GCAGGGAAGAATTGGAACAGG | 21 | 6593 | 52 | 63 |
|  |  |  | R_TCACGAAGCAACCCAACGA | 19 | 5760 | 53 | 60 |
| Gh_D02G0229 | UGT75L6 | PREDICTED: crocetin glucosyltransferase, chloroplastic-like | F_GAATCCACCATTAAAGTTCCAGT | 23 | 6992 | 39 | 59 |
|  |  |  | R_AACAACGACAGTGCCCAA | 18 | 5471 | 50 | 58 |
| Gh_D11G3369 | N | TMV resistance protein N | F_AGGAATTTGCGGAATGGGTG | 20 | 6277 | 50 | 60 |
|  |  |  | R_AAACCTGTGGCTAATTATGGC | 21 | 6735 | 43 | 61 |
| *GhACT4* | At5g09810 | Actin gene family | F_TTGCAGACCGTATGAGCAAG | 20 | 6166 | 50 | 60 |
|  |  |  | R_ATCCTCCGATCCAGACACTG | 20 | 6022 | 55 | 62 |
