# Supplementary material for: Transcriptome analysis of a nematode resistant and susceptible upland cotton line at two critical stages of Meloidogyne incognita infection and development
Source: PLoS One. 2019 Sep 10;14(9):e0221328. doi: 10.1371/journal.pone.0221328 (PMC6736245; doi:10.1371/journal.pone.0221328)
Supplement: S3 Table — (DOCX) [file pone.0221328.s004.docx]

| **gene** | **genotype** | **time point** | **expression** | **error values** | | | **p-value** |
| --- | --- | --- | --- | --- | --- | --- | --- |
|  |  |  |  | **positive** |  | **negative** |  |
| A11G2835 | M-120 | early | 1.223 | 0.262 |  | 0.305 | 0.121 |
|  |  | late | 1.189 | 0.137 |  | 0.104 | 0.019 |
|  | C201 | early | 0.948 | 0.601 |  | 0.277 | 0.813 |
|  |  | late | 0.379 | 0.451 |  | 0.201 | 0.000 |
| A11G3216 | M-120 | early | 2.061 | 0.519 |  | 0.495 | 0.001 |
|  |  | late | 0.859 | 0.619 |  | 0.349 | 0.529 |
|  | C201 | early | 1.334 | 1.064 |  | 0.603 | 0.207 |
|  |  | late | 1.374 | 1.772 |  | 0.795 | 0.353 |
| A11G3090 | M-120 | early | 2.806 | 0.851 |  | 1.426 | 0.001 |
|  |  | late | 1.704 | 0.863 |  | 0.623 | 0.015 |
|  | C201 | early | 2.260 | 0.637 |  | 0.497 | 0.002 |
|  |  | late | 0.924 | 1.022 |  | 0.473 | 0.778 |
| A11G3289 | M-120 | early | 1.202 | 0.134 |  | 0.182 | 0.020 |
|  |  | late | 1.063 | 0.177 |  | 0.123 | 0.385 |
|  | C201 | early | 1.607 | 0.232 |  | 0.216 | 0.001 |
|  |  | late | 1.606 | 0.301 |  | 0.322 | 0.000 |
| A11G2836 | M-120 | early | 1.031 | 0.405 |  | 0.294 | 0.815 |
|  |  | late | 0.951 | 0.201 |  | 0.153 | 0.562 |
|  | C201 | early | 1.381 | 0.635 |  | 0.327 | 0.024 |
|  |  | late | 2.905 | 0.429 |  | 0.347 | 0.010 |
| D11G3369 | M-120 | early | 2.179 | 1.498 |  | 0.930 | 0.000 |
|  |  | late | 2.104 | 0.369 |  | 0.432 | 0.002 |
|  | C201 | early | 0.388 | 0.153 |  | 0.118 | 0.005 |
|  |  | late | 0.650 | 0.501 |  | 0.322 | 0.289 |
| D02G0257 | M-120 | early | 1.954 | 0.297 |  | 0.224 | 0.000 |
|  |  | late | 1.694 | 0.742 |  | 0.550 | 0.000 |
|  | C201 | early | 1.949 | 0.480 |  | 0.379 | 0.000 |
|  |  | late | 1.340 | 0.220 |  | 0.205 | 0.003 |
| D02G0259 | M-120 | early | 2.339 | 0.518 |  | 0.434 | 0.004 |
|  |  | late | 1.772 | 0.517 |  | 0.417 | 0.001 |
|  | C201 | early | 1.200 | 0.400 |  | 0.306 | 0.136 |
|  |  | late | 0.886 | 0.705 |  | 0.421 | 0.591 |
| D02G0264 | M-120 | early | 0.877 | 0.942 |  | 0.419 | 0.636 |
|  |  | late | 1.128 | 0.417 |  | 0.339 | 0.437 |
|  | C201 | early | 0.168 | 0.022 |  | 0.020 | 0.101 |
|  |  | late | 1.275 | 3.382 |  | 0.920 | 0.572 |
| D02G0201 | M-120 | early | 3.629 | 4.080 |  | 1.981 | 0.000 |
|  |  | late | 0.694 | 0.750 |  | 0.355 | 0.224 |
|  | C201 | early | 1.638 | 0.759 |  | 0.526 | 0.014 |
|  |  | late | 1.566 | 1.979 |  | 0.844 | 0.166 |
| D02G0229 | M-120 | early | 0.680 | 0.727 |  | 0.355 | 0.189 |
|  |  | late | 0.680 | 1.476 |  | 0.458 | 0.537 |
|  | C201 | early | 1.592 | 1.239 |  | 0.470 | 0.048 |
|  |  | late | 0.911 | 1.844 |  | 0.633 | 0.844 |
| D02G0227 | M-120 | early | 3.873 | 1.099 |  | 0.495 | 0.000 |
|  |  | late | 0.967 | 0.891 |  | 0.510 | 0.904 |
|  | C201 | early | 2.860 | 0.489 |  | 0.348 | 0.002 |
|  |  | late | 1.536 | 0.940 |  | 0.589 | 0.065 |
